# Clinical practice guidelines for rituximab treatment in children with steroid-sensitive nephrotic syndrome

**DOI:** 10.1007/s12519-025-00957-9

**Published:** 2025-08-13

**Authors:** Qian Shen, Zheng-Kun Xia, Jia-Lu Liu, Lei-Lin Shao, Qiu-Xia Chen, Hui-Shan Wang, Ying Shen, Jian-Hua Mao, Xiao-Yun Jiang, Cui-Hua Liu, Xiao-Shan Shao, Xiao-Wen Wang, Xia Gao, Chong-Fan Zhang, Ai-Hua Zhang, Hong Xu, the Working Group on Clinical Practice Guidelines for the Rituximab Treatment in Children with Steroid-sensitive Nephrotic Syndrome

**Affiliations:** 1 Department of Nephrology, Children’s Hospital of Fudan University, National Key Laboratory of Kidney Diseases, National Children’s Medical Center, Shanghai, 201102 China; 2 Shanghai Kidney Development and Pediatric Kidney Disease Research Center, Shanghai, 201102 China; 3 Department of Nephrology, General Hospital of Eastern Theater Command, Nanjing, China; 4 Department of Nephrology, Children’s Hospital of Nanjing Medical University, Nanjing, 210008 China; 5 Fudan University GRADE Center, Shanghai, 201102 China; 6 Department of Nephrology, Beijing Children’s Hospital of Capital Medical University, Beijing, China; 7 Department of Nephrology, Children’s Hospital of Zhejiang University School of Medicine, Hangzhou, China; 8 Department of Nephrology, The First Affiliated Hospital of Sun Yat-Sen University, Guangzhou, China; 9 Department of Nephrology and Rheumatology, Henan Children’s Hospital, Zhengzhou, China; 10 Department of Nephrology, Guiyang Children’s Hospital, Guizhou, China; 11 Department of Nephrology and Rheumatology, Wuhan Children’s Hospital of Tongji Medical College, Huazhong University of Science and Technology, Wuhan, China; 12 Department of Nephrology, Guangzhou Women’s and Children’s Medical Center, Guangzhou, China

**Keywords:** Guidelines, Pediatrics, Rituximab, Steroid-sensitive nephrotic syndrome

## Abstract

**Background:**

Steroid-sensitive nephrotic syndrome is a prevalent glomerular disease in children. The 2021 guidelines for glomerular disease management by Kidney Disease: Improving Global Outcomes and the 2023 recommendations for steroid-sensitive nephrotic syndrome management by International Pediatric Nephrology Association recommend rituximab for frequently relapsing nephrotic syndrome and steroid-dependent nephrotic syndrome in children. However, there is considerable variation in rituximab application, including administration timing, dose, frequency, concomitant medications, and follow-up schedules. In addition, rituximab use for nephrotic syndrome remains off-label in most countries.

**Data sources:**

The “Pediatric Nephrology Committee of the Chinese Medical Doctor Association”, the “Pediatric Nephrology Society of the Chinese Medical Association”, and the “Fudan University GRADE Center” collaborated to develop a clinical practice guideline for rituximab in pediatric steroid-sensitive nephrotic syndrome. Databases and starting/ending time for retrieval were as follows. Databases in English: PubMed, Embase, Cochrane, and Scopus; database in Chinese: Chinese Biomedical Literature Service provided by SinoMed. The publication dates were limited to those from 2004 to June 26, 2022.

**Results:**

Through systematic reviews and meta-analyses covering nine clinically relevant patient or population covered, intervention, comparator, and outcome questions, seven recommendations were formulated and formally graded according to these guidelines.

**Conclusions:**

This guideline aspires to serve as a pivotal resource for healthcare providers, offering guidance on administration timing, dosage, frequency, concomitant medications, and follow-up protocols.

**Supplementary Information:**

The online version contains supplementary material available at 10.1007/s12519-025-00957-9.

## Introduction

Primary nephrotic syndrome (PNS) is one of the most common glomerular diseases in children. After treatment with glucocorticoid (also interchangeably mentioned below) regimens, which are currently recognized by international communities, 80%–90% of children with PNS present with steroid-sensitive nephrotic syndrome (SSNS), but up to 50% of them progress to frequently relapsing nephrotic syndrome (FRNS)/steroid-dependent nephrotic syndrome (SDNS) [1, 2]. In addition, 15%–25% of children with FRNS/SDNS are expected to continue experiencing the disease into adulthood, requiring repeated steroid therapy plus other immunosuppressants. These include calcineurin inhibitors (CNIs), such as tacrolimus and cyclosporine (CsA), as well as cyclophosphamide (CTX) and mycophenolate mofetil (MMF). Steroids and other related immunosuppressants are associated with significant adverse reactions [3].

Rituximab (RTX), a human‒mouse chimeric anti-CD20 monoclonal antibody, has been increasingly utilized in the management of FRNS/SDNS since 2004 [4]. Serendipitously, RTX therapy, which was originally employed for idiopathic thrombocytopenic purpura in a child with FRNS/SDNS, also contributed to achieving sustained remission from proteinuria [5].

RTX is recommended for the treatment of FRNS/SDNS in children by both the 2021 guidelines for the management of glomerular diseases issued by Kidney Disease: Improving Global Outcomes (KDIGO) [6] and the 2023 recommendations for the management of children with SSNS issued by the International Pediatric Nephrology Association (IPNA) [7]. However, many differences in the use of RTX, from administration timing, dose and frequency, concomitant medications, and follow-up schedules, occur in practice. Nevertheless, no consensus or guidelines on the application of RTX alone in pediatric SSNS are available at present, and the use of RTX for treating nephrotic syndrome (NS) in some countries is still off-label. Therefore, in June 2022, the “Pediatric Nephrology Committee of the Chinese Medical Doctor Association”, the “Pediatric Nephrology Society of the Chinese Medical Association”, and the “Fudan University GRADE Center” jointly initiated the development of a clinical practice guideline for RTX in pediatric SSNS (hereinafter referred to as the Guidelines).

Through a systematic literature review covering nine clinically relevant patient or population covered, intervention, comparator, outcome (PICO) questions (Supplementary material 1), seven recommendations were formulated and formally graded in these guidelines.

## Definitions

The following definitions are used for better clarification of the recommendations in these guidelines, some of which are the terms extracted from classical textbooks and the latest authoritative guidelines or consensus, or clinical cut-offs that are set to suit related recommendations herein as not being established by any consensus before, or the ones as requirements for follow-up durations adaptable to the outcome indicators on the basis of the recommendations.

### Relevant definitions

NS: nephrotic-range proteinuria and either hypoalbuminemia (serum albumin < 30 g/L) or edema when serum albumin is not available [8]. SSNS: NS type with complete remission of proteinuria within four weeks of prednisone or prednisolone at standard doses (2 mg/kg/day or 60 mg/m^2^/day, maximum 60 mg/day) [8]. Steroid-dependent nephrotic syndrome (SDNS): frequent relapses with ≥ 2 consecutive relapses while on steroid therapy or relapses occurring within two weeks of cessation of steroid therapy [8]. FRNS: ≥ 2 relapses in the first six months following remission of the initial episode or ≥ 3 relapses in any 12 months [8].

Complete response: urine protein/creatinine (UPCR, based on first morning void or 24-hour urine sample) ≤ 20 mg/mmoL (0.2 mg/mg) or < 100 mg/m^2^/day, respectively, or negative or trace dipstick on ≥ 3 consecutive days [8]. Relapse: urine dipstick ≥ 3 + (≥ 300 mg/dL) or UPCR ≥ 200 mg/mmol (2 mg/mg) on a morning urine sample on three consecutive days in an NS child who had previously achieved complete remission [8].

Follow-up duration: the period for follow-up in terms of relapse or steroid withdrawal. Herein, literature data with a follow-up duration of ≥ 1 year were included for calculating relapse, and literature data with a follow-up duration of ≥ 3 months were used for calculating the steroid withdrawal rate. Time to first relapse: the time from the initiation of RTX intervention to the first relapse. Steroid dose/cumulative steroid dose: the cumulative steroid dosage before and/or one year after RTX intervention, expressed in mg/kg/day, mg/kg/year, or mg/m^2^/day. Time to steroid withdrawal: the time to first steroid discontinuation during the ≥ 1-year follow-up after RTX intervention. Steroid-free duration: sum of intervals with steroid discontinuation during the one-year follow-up after RTX intervention. Steroid withdrawal rate: in children followed up for ≥ 3 months after intervention with RTX, the proportion of patients with withdrawn steroids was determined. Relapse rate: in children followed up for ≥ 1 year after intervention with RTX, the proportion of patients who experienced relapse.

B-cell depletion: complete removal of peripheral blood B cells, indicated by a CD19 + /CD20 + B-cell count < 5 cells/μL or < 1% total lymphocytes. B-cell reconstitution: three circumstances of recovery after peripheral B-cell depletion, as summarized by the guidelines in the included literature: (1) peripheral CD19 + /CD20 + B cells > 5 cells/μL or > 1% total lymphocytes; (2) peripheral CD19 + /CD20 + B cells > 10 cells/μL; (3) peripheral CD19 + /CD20 + B cells > 15 cells/μL or > 3% total lymphocytes.

Adverse events: the total untoward medical occurrences reported in the included literature. Serious adverse events: the total number of serious untoward medical occurrences reported in the included literature and adverse events, including life-threatening events, leading to death, significant disability, incapacity, hospitalization, or prolongation of existing hospitalization**.** Infusion-related reactions: disorders characterized by adverse reactions to the infusion of pharmacological or biological substances [3].

Cut-offs for absolute peripheral neutrophil count: < 1.5 × 10^9^/L for neutropenia and < 0.5 × 10^9^/L for severe neutropenia [5]. Cutoff for absolute peripheral lymphopenia: < 0.8 × 10^9^/L [3].

Infection: local or systemic inflammatory reactions caused by pathogens, such as bacteria, viruses, fungi, and parasites. Hypogammaglobulinemia: disorders with a serum immunoglobulin G (IgG) value lower than the mean among the normal population of the same age range by two standard deviations [4, 6] and persistent hypogammaglobulinemia as the disorder lasting for > 1 year [9]. Serum sickness: delayed hypersensitivity reactions caused by the infusion of exogenous protein usually occur 6 to 21 days after the infusion, with clinical manifestations, such as fever, arthralgia, myalgia, and rash [3]. Detection of the serum concentration of anti-RTX antibody (ARA) usually requires commercial enzyme-linked immunosorbent assay (ELISA) or enhanced chemiluminescence (ECL) kits [10, 11].

RTX dose and RTX course: RTX dose refers to a single infusion of RTX. RTX course refers to complete set of RTX doses administered within a defined treatment period. This may consist of one or more individual RTX doses.

### Conversion, classification, and comparison

Conversion for year or month: 30 days or four weeks for a month and 365 days or one year per year. Conversion between body surface area and weight: for a child with a weight ≤ 30 kg, body surface area (m^2^) = weight (kg) × 0.035 + 0.1; for a child with a weight > 30 kg, body surface area (m^2^) = [weight (kg) − 30] × 0.02 + 1.05.

FRNS/SDNS patients: in the guidelines, the FRNS and/or SDNS cases, with or without historic administration of CNI or other immunosuppressants other than steroid(s) and levamisole before the use of RTX, are expressed as FRNS/SDNS + and FRNS/SDNS − , respectively. The cases that cannot be distinguished between FRNS/SDNS + and FRNS/SDNS − in the literature are generally referred to as FRNS/SDNS.

Steroids: whether RTX and/or other immunosuppressive agents are used to treat FRNS/SDNS, steroids are considered primary treatments. Therefore, the guidelines state that the use of RTX and other immunosuppressive agents is based on steroid treatment.

Single-dose RTX treatment: 375 mg/m^2^ when a single dose of RTX is not specifically indicated in the guidelines. Control therapy against RTX: CTX, CNI, MMF, placebo, or steroids alone.

## Aim and list of clinical topics

This policy aims to do the following: develop standard guidelines to answer the main clinical questions regarding the indications; treatments, and follow-up management for the application of RTX in SSNS children.

List of clinical topics: (1) Does RTX improve clinical outcomes in children aged 1–18 years with SSNS compared with other immunosuppressants or no treatment/placebo control? (2) What are the effects on the clinical outcomes of different steroid or immunosuppressant discontinuation regimens in children aged 1–18 years with SSNS after RTX treatment? (3) Is it feasible to use the peripheral CD19 + /CD20 + B-cell count as a monitoring indicator in children aged 1–18 years with SSNS after RTX treatment? (4) Is it feasible to use the peripheral CD19 + /CD20 + B-cell count as a predictor of relapse in children aged 1–18 years with SSNS after RTX treatment? (5) What are the effects on the clinical outcomes of maintenance therapy in children aged 1–18 years with SSNS after RTX treatment? (6) What are the effects on the clinical outcomes of repeated RTX treatment in children aged 1–18 years with SSNS relapsing after RTX treatment? (7) What is the cost-effectiveness of RTX treatment in children aged 1–18 years with SSNS? (8) What is the incidence of adverse events (including allergies, infections, hypoleukocytosis, hypogammaglobulinemia, etc.) in the RTX treatment (first use and second use) of children with SSNS? (9) What measures should be taken to prevent RTX-induced adverse reactions (first use and second use)?

## Guideline development

### Registration of the rituximab guidelines

This document was registered in both Chinese and English on the Practice Guideline REgistration for transPAREncy (PREPARE), with the registration number IPGRP-2022CN361. Starting date: June 20, 2022; finalization date: May 17, 2023 (Table 1).

**Table 1:** Schedule of guideline development

Topics	Description	Time (wk)
Planning of guideline		
Construction of guideline topics	Preliminary survey of guideline topics and development of a list of clinical questions	May 25–June 19, 2022
Establishment of guideline development group	Establishment of core expert group (Secretariat), guideline panel	~ June 20
Distribution, recovery and review of conflict-of-interest declaration	Distribution, recovery and review the conflict-of-interest form from the members of the guideline panel	June 20–24, 2022
Registration of the guidelines and planning proposal writing	Completion of registration on the platform of the Practice Guideline REgistration for transPAREncy (PREPARE)	June 24–27, 2022
Evidence retrieval	PICO construction, database retrieval, keywords, and search query	June 24–26, 2022
Literature screening	Preliminary screening by title and abstract review, and screening by full-text review	June 28–October 25, 2022
Data extraction and evidence synthesis	Extraction of data, and synthesis of evidence	~ March 27, 2023
Bias risk assessment of literature	RCT: RoB2; non-RCT, cohort study: ROBINS-I	~ April 14, 2023
Generation of GRADE evidence profile	Pooling of evidence or a body of evidence	~ April 18, 2023
Evidence-to-recommendations	GRADE EtD frameworks and Delphi rounds	April 19, 2023
Guideline writing	Guidelines drafted by the secretariat and refined by the panel of experts	~ April 25, 2023
External review	Peer review by correspondence	June 6, 2023
Finalization of guideline	Finalization of the guidelines after refining recommendations as per external review feedback	2023

*RCT* randomized-controlled trials, *RoB2* risk of bias version 2, *ROBINS-I* risk of bias in nonrandomized studies of interventions, *GRADE* Grades of Recommendation, Assessment, Development, and Evaluation, *EtD* evidence to decision, *PICO* clinically relevant patient or population covered, intervention, comparator, and outcome

### Development method for the rituximab guidelines

The development was conducted as per the WHO Handbook for Guideline Development [12], as illustrated in Supplementary material 2. The statistical analysis included meta-analyses conducted via R 4.2.2 and Stata 17.0 software. For uncontrolled binary variables, the median survival ratio (MSR) was utilized as the effect variable, with its corresponding 95% confidence interval (CI) provided. The significance level for the meta-analysis was set at *α* = 0.05. The random-effects model was employed as the preferred combined effects model, whereas the fixed-effects model was chosen in cases where there were ≤ 5 documents included or if a document’s weight exceeded 70%. Heterogeneity was deemed to be present when the *Q* test yielded a *P* value < 0.1 and the *I*^2^ test indicated *I*^2^ > 50%. For single-arm data, the analysis utilized either the generalized linear model (GLMM) method or the inverse variance (inverse) method. When only medians (including the maximum, minimum, or interquartile range) and their standard deviations were available, the Quantile Estimation (QE) method was applied. Two-arm data analysis depends on the situation of sparse data. The M–H method was preferred when the sample size of the literature was less than 100, and in situations where the incidence rate was extremely low with single zero data, the Peto method was employed. When the incidence rate included double 0 or double 1 data, the hypergeometric normal model in the GLMM method was utilized.

### Proposal and determination of clinical questions

A preliminary survey on guideline topics was conducted from May 25, 2022, to June 8, 2022. According to the preliminary survey results, the guideline topics were summarized and integrated from June 10, 2022, to June 17, 2022, to construct the PICO. A questionnaire survey for the priority of the questions was conducted among 50 clinical experts from June 16, 2022, to June 19, 2022. On the basis of the International Guideline Credentialing & Certification (INGUIDE) Program, nine clinical topics were selected from all the topics assessed (Supplementary material 3).

### Determination of primary outcomes

In the Working Group, the following primary outcomes were determined: (1) relapse-free survival (follow-up > 3 months); (2) steroid dose (follow-up > 3 months); (3) time of steroid withdrawal; (4) quality of life (follow-up > 1 year); (5) depletion of CD20 + /CD19 + B cells; and (6) elevated urine protein or elevated CD20 + /CD19 + B cells (follow-up > 3 months).

### Literature retrieval and results

Databases and starting/ending time for retrieval were as follows. Databases in English: PubMed, Embase, Cochrane, and Scopus; database in Chinese: Chinese Biomedical Literature Service provided by SinoMed. The publication dates were limited to those from 2004 to June 26, 2022.

Retrieval results: a total of 3843 articles were retrieved from English databases, including 822 from PubMed, 1729 from Embase, and 129 from Cochrane; 487 papers were from the Chinese database, 459 of which were selected after the exclusion of irrelevant or repeated articles.

### Literature screening

Preliminary screening and full-text screening were completed by Jia-Lu Liu, Qiu-Xia Chen, and Lei-Lin Shao. For preliminary screening, the titles and abstracts of the articles were independently checked. For full-text screening, each person completed 75% of all the documents, ensuring that each article was checked by two persons. Any documents with disagreement or uncertainty were finally reviewed by Shen Qian.

**Fig. 1:**
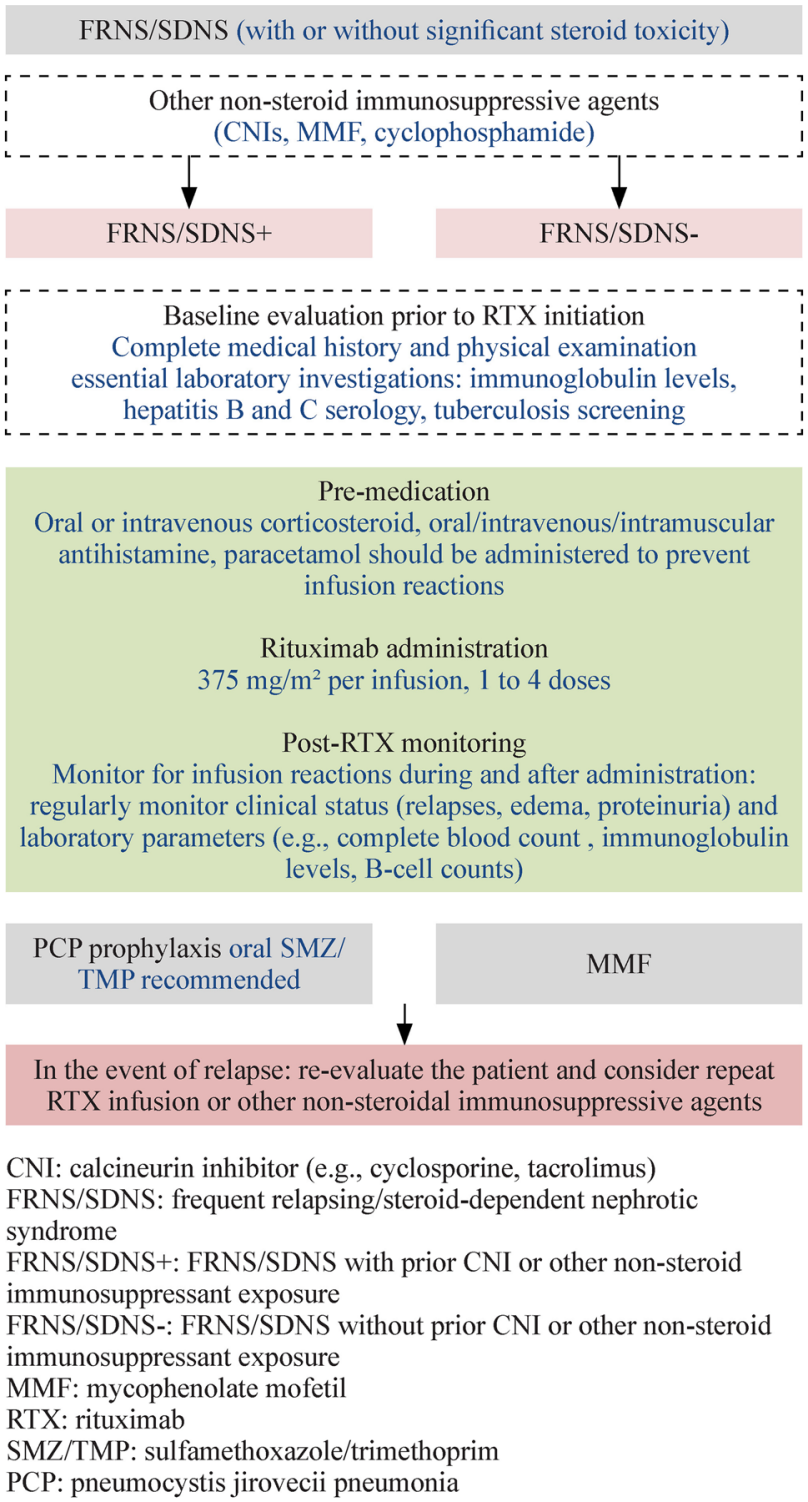
Flowchart for rituximab use in patients with steroid-sensitive nephrotic syndrome. *CNI* calcineurin inhibitor (e.g., cyclosporine, tacrolimus), *FRNS/SDNS* frequent relapsing/steroid-dependent nephrotic syndrome, *FRNS/SDNS* + FRNS/SDNS with prior CNI or other non-steroid immunosuppressant exposure, *FRNS/SDNS − *FRNS/SDNS without prior CNI or other non-steroid immunosuppressant exposure, *MMF* mycophenolate mofetil, *RTX* rituximab, *SMZ/TMP* sulfamethoxazole/trimethoprim, *PCP* pneumocystis jirovecii pneumonia

Among the 459 articles identified in the preliminary screening, 132 articles were subsequently included in the full-text screening, with the other 327 excluded for ineligibility in their title or abstract (Fig. 1). During the full-text screening, another 57 articles were excluded; 2 were included; 75 articles were extracted for data; 12 articles were further removed after discussion at recommendation meetings, and 65 articles were included.

Moreover, to prepare the guidelines, a total of 164 entries related to clinical trial registration were retrieved preliminarily (108 from the International Clinical Trials Registry Platform, 22 from clinicaltrials.gov, 30 from CHiCTR, and four randomized-controlled trials (RCTs) from the literature references of this document), and 141 of them were then removed for relevance. Within the remaining 23 entries, 15 of them had trial results officially published by the end of the external review of this document, but four of the latter met the exclusion criteria, while the other 11 were among the 65 studies included.

### Data extraction and evidence synthesis

Jia-Lu Liu, Lei-Lin Shao, and Qiu-Xia Chen were responsible for the data extraction, each completing the work for 75% of all the articles, and two persons working for the extraction of the same article. Any data with disagreement or uncertainty after verification were finally reviewed by Shen Qian. The evidence synthesis was finished with the assistance of Hui-Shan Wang, with the training provided by Chong-Fan Zhang, a member of the Methodology Team.

### Assessment and grading of evidence

Chong-Fan Zhang provided the training for this work, which was carried out by Jia-Lu Liu, Qiu-Xia Chen, and Lei-Lin Shao. For the assessment of bias risk, the risk of bias version 2 (RoB2) tool [13] was applied for RCT articles, and the risk of bias in nonrandomized studies (ROBINS) tool [14] was used for non-RCT articles. Then, the quality level of each article was discussed for any change per Grades of Recommendation, Assessment, Development, and Evaluation (GRADE) [15–17]; case series reports (CSRs) were not subject to bias risk assessment (Supplementary material 5 and 6).

### Formulating recommendations

The evidence to decision (EtD) panel consists of China-domestic clinical experts and research experts, social workers, and parents of pediatric patients. The Secretariat provided all the evidence for discussion of the proposed recommendations among the EtD group, and the Methodology Team implemented the EtD decision-making process to determine the strength and direction of each recommendation (Supplementary material 7).

### Criteria for commissioning

For the Chinese database, the search terms in Chinese related to SSNS, RTX, and the like were used; for the English databases, steroid-sensitive nephrotic syndrome, frequently relapsing nephrotic syndrome, steroid-dependent nephrotic syndrome, rituximab, etc., were used (Supplementary material 4).

The exclusion criteria for primary screening and full-text screening were as follows: (1) subjects as animals or cells; (2) infants or individuals aged > 22 years as subjects; (3) diagnoses not conforming to the SSNS; (4) no RTX intervention for at least one group; and (5) traditional reviews, case reports (each with fewer than three patients), commentaries, conference communications, and documents without full text.

## Evidence and rationale

Figure 1 illustrates the clinical practice flowchart for rituximab use in patients with steroid-sensitive nephrotic syndrome.

### Recommendation 1: rituximab is recommended for the treatment of children with FRNS/SDNS, and it can achieve better efficacy in FRNS/SDNS − than in FRNS/SDNS + (1D)

Rationale: the recommendation is justified in terms of disease relapse, steroid reduction and time to steroid withdrawal after RTX treatment in children with FRNS/SDNS.

#### Relapse rate

The one-year relapse rate of RTX treatment in children with FRNS/SDNS was lower than that of placebo (decrease of 95%) and CTX, CNI, or MMF (decrease of 68%).

A meta-analysis of 10 studies [including seven RCTs [18–24], one nonrandomized-controlled trial (NRCT) [25], and two cohort studies [26, 27]] with a one-year follow-up on the relapse rate of FRNS/SDNS treated with RTX revealed a statistically significant 82% reduction compared with the control group [odds ratio (OR) = 0.18, 95% CI 0.07–0.44] (Supplementary Fig. 1). In the FRNS/SDNS + subgroup, the relapse rate related to RTX decreased by 81% compared with that in the control group (OR = 0.19, 95% CI 0.05–0.68), and the difference was statistically significant. In the FRNS/SDNS − subgroup, the relapse rate associated with RTX was 85% lower than that in the control group (OR = 0.15, 95% CI 0.03–0.68), and the difference was statistically significant. Compared with the placebo or no treatment subgroup, the rate of relapse with RTX decreased by 94% (OR = 0.06, 95% CI 0.02–0.18), and the difference was statistically significant. Compared with those in the CTX, CNI, or MMF subgroups, the rate of relapse with RTX decreased by 68% (OR = 0.32, 95% CI 0.13–0.82), and the difference was statistically significant.

The average one-year relapse rate following RTX treatment was 41% in children with FRNS/SDNS (47% in FRNS/SDNS + and 22% in FRNS/SDNS −).

A meta-analysis of 21 studies [eight CSRs [28–35] and the RTX groups in seven RCTs [18–21, 23, 24, 36], one non-randomized studies of interventions (NRSI) [25], and five cohort studies [26, 27, 29, 37, 38]] on the relapse rate at 12 months (Supplementary Fig. 3) revealed an overall relapse rate of 41% (95% CI 31%–52%), a rate of 47% (95% CI 36%–59%) in the FRNS/SDNS + subgroup, and 22% (95% CI 9%–37%) in the FRNS/SDNS − subgroup; there was a statistically significant difference in the data between the two subgroups (*χ*^2^ = 26.926, *P* < 0.001).

The meta-analysis (Supplementary Fig. 4) of 12 studies (five CSRs [28, 30, 31, 33, 35] and the RTX groups in four RCTs [19–21, 24] and one NRSI [25] and two cohort studies [27, 29]) on FRNS/SDNS after 1–2 doses of RTX with a follow-up of 12 months revealed an overall relapse rate of 33% (95% CI 22%–45%), a rate of 14% (95% CI 7%–21%) in the FRNS/SDNS − subgroup, and 42% (95% CI 30%–55%) in the FRNS/SDNS + subgroup, with a statistically significant difference between the two subgroups (*χ*^*2*^ = 27.494, *P* < 0.001).

There was no difference in the two-year relapse rate of the FRNS/SDNS group between the RTX treatment group and the continuous immunosuppressive therapy group, but there were higher two- and three-year relapse rates in the FRNS/SDNS + group than in the FRNS/SDNS − group after RTX treatment.

Pooling the three FRNS/SDNS studies on RTX (one RCT [19], two cohort studies [27, 39]), the meta-analysis for the 24-month follow-up relapse rate (Supplementary Fig. 5) revealed no statistically significant difference between the RTX group and the steroid alone group or the CNI or CTX control subgroup (OR = 0.65, 95% CI 0.31–1.35), no such difference in the FRNS/SDNS + subgroup (CNI or CTX as a control), and no such difference in the FRNS/SDNS − subgroup (placebo as a control).

The meta-analysis of the relapse rate at the 24-month follow-up in 10 studies (five CSRs [28, 30–32, 40] and the RTX groups in two RCTs [19, 21] and three cohort studies [27, 37, 39]) for RTX treatment in FRNS/SDNS patients (Supplementary Fig. 6) revealed a rate of 72% (95% CI 61%–83%), 75% (95% CI: 63%–86%) in the FRNS/SDNS + subgroup, and 54% (95% CI 33%–75%) in the FRNS/SDNS-subgroup, with a statistically significant difference between the two subgroups (*χ*^*2*^ = 4.376, *P* = 0.036).

Another meta-analysis of the relapse rate at the 36-month follow-up in four studies (two CSRs [28, 40] and the RTX group in one RCT [21]) for RTX treatment in FRNS/SDNS (Supplementary Fig. 7) reported a rate of 85% (95% CI 76%–91%), 92% (95% CI 84%–96%) in the FRNS/SDNS + subgroup, and 47% (95% CI 21%–73%) in the FRNS/SDNS − subgroup, with a statistically significant difference between the two subgroups (exact test, *P* < 0.001).

In a multicenter RCT, 120 patients with SDNS were randomized to receive RTX or tacrolimus treatment, followed up for one year (RITURNS trial [20]) and then for another two years (RITURNS II trial [41]). Once relapse occurred, all the patients in the RTX group received the second course of RTX treatment, 44 of whom received RTX plus MMF and 15 of whom did not receive MMF. The 56 patients who relapsed in the tacrolimus group switched to RTX treatment, 52 of whom switched to RTX + MMF and four to RTX alone. The two-year relapse-free survival rates (*n* = 19) was 9% in the RTX monotherapy arm and 67% in the RTX + MMF arm (*n* = 96), respectively, with a statistically significant difference (*P* < 0.001).

#### Steroid reduction

RTX treatment significantly improved one-year cumulative steroid dose reduction in FRNS/SDNS pediatric patients compared with other immunosuppressants (RTX vs. tacrolimus, − 0.15 mg/kg/day; RTX vs. placebo, − 0.26 mg/kg/day).

Pooling the four studies (three RCTs [18, 20, 23], and one cohort study [26]), the meta-analysis for the 12-month cumulative steroid dose revealed that RTX significantly reduced the mean 12-month cumulative steroid dose by 0.16 mg/kg/day compared with that of the tacrolimus or placebo control [mean decrease (MD) =  − 0.16, 95% CI − 0.20 to − 0.12], with a statistically significant difference (Supplementary Fig. 8); decreased the cumulative steroid dose by 0.15 mg/kg/day compared with that of the tacrolimus control subgroup (MD =  − 0.15, 95% CI − 0.18 to − 0.11), with a statistically significant difference; and significantly reduced cumulative steroid dose by 0.26 mg/kg/day compared with that of the placebo subgroup (MD =  − 0.26, 95% CI − 0.36 to − 0.16), with a statistically significant difference (Supplementary Fig. 9). There was a 0.14 mg/kg/day reduction in the FRNS/SDNS + subgroup compared with tacrolimus or placebo control (MD =  − 0.14, 95% CI − 0.21 to − 0.08), with the difference statistically significant (Supplementary Fig. 8); and a 0.17 mg/kg/day in the FRNS/SDNS − subgroup, compared with tacrolimus or placebo controls (MD =  − 0.17, 95% CI − 0.28 to − 0.07), with the difference statistically significant (Supplementary Fig. 9).

For FRNS/SDNS + , the meta-analysis of three RCTs [18, 20, 23] and two cohort studies [26, 38] comparing the cumulative steroid dose before and after intervention in the RTX groups over 12 months revealed a decrease of 0.35 mg/kg/day (MD =  − 0.35, 95% CI − 0.38 to − 0.31), with a statistically significant difference (Supplementary Fig. 10).

One RCT on RTX in FRNS/SDNS − patients [22] reported no difference between the RTX and CNI control groups in terms of the median cumulative steroid dose [0.11 vs. 0.11 mg/kg/day at the 12-month follow-up (*P* = 0.15)].

One NRSI in FRNS/SDNS − patients [25] revealed a statistically significant reduction in the one-year cumulative steroid dose by 0.78 ± 0.23 mg/kg qod at the 12-month follow-up in the RTX treatment group.

A case series report on RTX treatment of FRNS/SDNS + patients with a mean follow-up period of 17 (13–21) months [42] reported a significant reduction in the steroid dose (mg/kg/day) by 63% after 12 months of RTX exposure compared with the baseline.

Among the 65 articles retrieved for this guideline, the steroid molecules used to achieve remission before rituximab was prescribed were as follows: methylprednisolone was mentioned in five articles, prednisolone in 25, and prednisone in 16. Furthermore, 19 articles provided only a general mention of “steroids”, lacking molecular specificity.

#### Steroid withdrawal and duration of steroid therapy

The meta-analysis of two studies (one RCT [43] and one NRSI [25]) with RTX therapy for FRNS/SDNS revealed that the steroid withdrawal rate in the RTX group at the three-month follow-up was 16-fold greater than that in the CNI or CTX control group (OR = 15.71, 95% CI 5.72–43.16) (Supplementary Fig. 11).

The meta-analysis of steroid withdrawal rates during the 12-month follow-up in two studies (one RCT [20] and one cohort study [39]) on RTX intervention for FRNS/SDNS revealed no statistically significant difference between RTX and CNI or CTX control (OR = 1.93, 95% CI 0.87–4.29) (Supplementary Fig. 12).

The meta-analysis of six-month steroid withdrawal rates from three studies (two CSRs [29, 34] and the RTX arm in one cohort study [37]) in FRNS/SDNS + patients revealed a rate of 63% (95% CI 51%–73%) (Supplementary Fig. 13).

In a cohort study on RTX for the treatment of FRNS/SDNS [27], no statistically significant difference in the time to steroid withdrawal was found between the RTX group and the CNI control group during follow-up for 12–24 months.

One case series report on RTX treatment (*n* = 17) in FRNS/SDNS + patients [29] reported 100% steroid withdrawal at the three-month follow-up. Another case series report on RTX intervention (*n* = 81) in FRNS/SDNS + patients [44] reported a median time to steroid withdrawal of 66 (26–409) days for 69 patients during follow-up for 13–90 months. In a case series on RTX exposure (*n* = 101) in FRNS/SDNS + patients [38], the average time to steroid withdrawal in 90 patients was 4.8 ± 2.0 months. In another case series report on RTX intervention (*n* = 37) in FRNS/SDNS + patients [30], 35 patients discontinued steroid treatment after a median of 1.3 (0.37–6) months. The RCT on RTX in the treatment of FRNS/SDNS + [23] and the comparison between RTX and placebo or no treatment control revealed a statistically significant difference in steroid-free duration one year after intervention (140.5 ± 91.3 days, RTX vs. 80.2 ± 98.5 days, controls; *P* = 0.02), although the two groups had no statistically significant difference in this parameter before intervention.

The time to steroid withdrawal in the above-mentioned studies is summarized as follows: within three months for five studies [20, 25, 29, 39, 43], within 3–6 months for three studies [23, 38, 44], and within at least 6–9 months for one study [34].

#### Median time to first relapse

The median time to first relapse was about 10 months in RTX-treated children with FRNS/SDNS + .

The meta-analysis of nine studies (six CSRs [28, 30–33, 44] and the RTX arms in three cohort studies [26, 37, 39]) on RTX treatment in FRNS/SDNS + patients for outcome follow-up at ≥ 12 months revealed that the median time to first relapse was 9.89 (95% CI 7.14–12.65) months, with *I*^*2*^ = 82.88% and *P* < 0.001 (Supplementary Figs. 14–15).

The meta-analysis of relapse-free survival at the 12-month follow-up in three studies (two RCTs [19, 20], and one NRSI [25]) on FRNS/SDNS − patients treated with 1–2 doses of RTX revealed that the median time to first relapse in the arm of 1–2 doses of RTX was 57 days later than that in the CTX/CNI or placebo control groups, with a MSR = 1.93 (95% CI 1.62–2.30), *P* < 0.001, and the difference was statistically significant, with *Q* = 112.18, *P* < 0.001 for the heterogeneity test. In the RTX subgroup compared with the CTX/CNI subgroup, the MSR = 1.303 (95% CI 1.076–1.577), *P* = 0.007; in the RTX group compared with the placebo control, the MSR = 15.333 (95% CI 9.892–23.767), *P* < 0.001 (Supplementary Fig. 16).

In one RCT on the treatment of FRNS/SDNS + with four doses of RTX [18], compared with the placebo group, the RTX group achieved a median relapse-free survival of 166 days (267 vs. 101) at 12 months, with an hazard ratio (HR) of 0.27 (95% CI 0.14–0.53), and a median time from steroid withdrawal to first relapse prolonged by 169 (211 vs. 42) days, with an HR of 0.27 (95% CI 0.14–0.53).

In a cohort study of RTX in the treatment of FRNS/SDNS + patients [26], the time to first relapse between the RTX group and the CNI control group during the 12-month follow-up was not significantly different [(8.5 ± 5.1) months, RTX vs. (9.8 ± 5.6) months, CNI] (*P* = 0.65).

In another cohort study of RTX in the treatment of FRNS/SDNS [27], the remission duration between the RTX group and the CNI control group during the 12–24-month follow-up was not significantly different [(10.1 ± 4.9) months, RTX vs. (9.1 ± 4.4) months, CNI] (*P* = 0.72).

In a cohort study of RTX at a 750 mg/m^2^ × 1 dose in the treatment of FRNS/SDNS + [39], six out of eight patients relapsed within eight months and then turned to RTX at 750 mg/m^2^ × 2 doses (about two weeks apart); the difference in the median time to first relapse at follow-up ≥ 12 months between the two dose and one dose was statistically significant [16 (1–73) months vs. 5 (1–36) months] (*P* = 0.03).

In one case series of 51 FRNS/SDNS + patients [40], the median relapse-free duration was 261 days during a follow-up of > 36 months.

### Recommendation 2: for the first course of treatment in children with FRNS/SDNS, one-to-four doses of rituximab are needed, at 375 mg/m^2^ for each dose (2D)

Rationale: in the first case series reporting that RTX in children with FRNS/SDNS + maintained remission, the initial single dose of RTX was 375/mg/m^2^ [45].

An international multicenter retrospective cohort study of RTX in children with FRNS/SDNS (*n* = 511) [46] included 191 subjects at a low dose of 375 mg/m^2^, 208 at a medium dose of 750 mg/m^2^, and 112 at high doses of 1125 to 1500 mg/m^2^, with a follow-up period of > 18 months, and revealed that 327 patients received repeated RTX due to disease relapse, B-cell repletion, adverse reactions caused by steroids and other immunosuppressants, or as planned, during the follow-up period.

In the absence of maintenance therapy with immunosuppressants, the low-dose group had a shorter relapse-free period than did the medium- and high-dose groups did (8.5 months vs. 12.7 months vs. 14.3 months). Compared with the low-dose group, the medium-dose group had a 38% lower risk of relapse (HR = 0.62, 95% CI 0.41–0.94, *P* = 0.02), whereas the high-dose group had a 50% lower risk of relapse (HR = 0.50, 95% CI 0.33–0.77, *P* = 0.002). In the presence of immunosuppressant maintenance therapy, relapse-free survival was similar among the low-, medium- and high-dose groups (14 months vs. 10.9 months vs. 12.0 months), which suggests that both the total dosage of RTX and maintenance therapy with immunosuppressants have important impacts on treatment outcomes.

The pooled results of 12-month follow-up in FRNS/SDNS + for a total dose of 1500 mg/m^2^, including two CSRs [30, 34] on RTX at 750 mg/m^2^, once every two weeks for a total of two doses, and one RCT [18] on RTX at 375 mg/m^2^ in FRNS/SDNS + , once a week for a total of four doses, revealed that the relapse rate of 750 mg/m^2^ was 37% (95% CI 28%–46%), whereas the rate of 375 mg/m^2^ was 42% (95% CI 28%–57%).

The meta-analysis of the relapse rates at the 12-month follow-up in 13 studies (six CSRs [28–31, 33, 35], three RCTs [18, 19, 24], and RTX groups in four cohort studies [26, 27, 29, 38]) on FRNS/SDNS + patients treated with one or 2–4 doses of RTX (Supplementary Fig. 17) revealed a relapse rate of 37% (95% CI 28%–46%) for the one-dose treatment group and 42% (95% CI 28%–57%) for the 2–4 dose group, with the difference not statistically significant (*χ*^2^ = 0.15, *P* = 0.67).

The meta-analysis of the relapse rates at the 12-month follow-up in four studies (RTX groups in three RCTs [20–22] and one NRSI [25]) on FRNS/SDNS − patients treated with one dose or 2–4 doses of RTX (Supplementary Fig. 18) showed a relapse rate of 13% (95% CI 2%–40%) for one dose and 16% (95% CI 9%–25%) for 2–4 doses, revealing that the difference was not statistically significant (*χ*^2^ = 0.212, *P* = 0.645).

The meta-analysis of the relapse rates at the 12-month follow-up in 11 studies (six CSRs [28–31, 33, 35], three RCTs [18, 19, 24], and two cohort studies [27, 29]) on FRNS/SDNS + patients treated with 1–2 or 3–4 doses of RTX (Supplementary Fig. 19) revealed a relapse rate of 41% (95% CI 32%–49%) for the 1–2 dose group and 50% (95% CI 10%–89%) for the 3–4 dose group, with the difference not statistically significant (*χ*^2^ = 2.204, *P* = 0.138).

The guidelines herein are based on the outcome of 12-month follow-up in 21 articles, including 19 concerning a single dose of 375 mg/m^2^ in the first course of treatment, one dose every 1–2 weeks, for a total of 1–4 doses; one article on a single dose of 100–375 mg/m^2^ in the first course of treatment, one dose every week, for a total of 1–2 doses; and the other paper concerning a single dose of 750 mg/m^2^, one dose every two weeks, for two doses in total. The Core Expert Group of the Guidelines considers that, since the comparative conclusion between a single dose of RTX at 375 mg/m^2^ and other doses for relapse, steroid reduction, and adverse reactions is not available, a regimen with a dose of 375 mg/m^2^, one dose every 1–2 weeks, for a total of 1–4 doses, is currently the most common regimen used in clinical practice.

### Recommendation 3: rituximab + MMF is recommended for the treatment of FRNS/SDNS in consideration of the delayed time to first relapse and reduced steroid dose (1D)

#### Rationale: delayed time to first relapse

In the meta-analysis of follow-up at ≥ 12 months in two studies (one RCT [47] and one self-controlled study [28]), in which MMF was added to RTX for the treatment of children with FRNS/SDNS (Supplementary Fig. 20), the time to first relapse in the combination of RTX + MMF was significantly prolonged by 2.7-fold compared with that of RTX alone [median time to first relapse ratio = 2.66 (95% CI 2.14–3.30)].

In the follow-up ≥ 12 months in one non-RCT for RTX + MMF treatment in FRNS/SDNS + patients [48], a statistically significant difference in the annual relapse rate was found between RTX + MMF (three out of nine patients) and RTX alone (six out of seven patients) (relapse frequency: 0.42 vs. 2.3) (*P* < 0.001).

In the meta-analysis (Supplementary Fig. 21) of five studies (one RCT [47], two non-RCTs [48, 49] and two self-controlled trials [28, 42]) on RTX + MMF in FRNS/SDNS + with a follow-up of ≥ 12 months, there was no statistically significant difference in the relapse rate between RTX + MMF and RTX + CsA (OR = 2.89, 95% CI 0.59–14.11), whereas the relapse rate in the RTX + MMF group was reduced by 84% compared with that in the RTX + placebo or immunosuppressant-withdrawal groups (OR = 0.16, 95% CI 0.07–0.35), with a statistically significant difference.

In an international, multicenter, retrospective cohort study on RTX in children with FRNS/SDNS (*n* = 511) [46], which included 191 patients receiving a low dose of 375 mg/m^2^, 208 receiving a medium dose of 750 mg/m^2^, and 112 receiving a high dose of 1125–1500 mg/m^2^, with a follow-up of > 18 months, similar relapse-free survival rates (14 months vs. 10.9 months vs. 12.0 months) were observed in the presence of maintenance therapy with immunosuppressants among the three groups, suggesting that both the total RTX dose and maintenance therapy with immunosuppressants have important influences on treatment outcomes.

#### Rationale: steroid dose reduction

The meta-analysis (Supplementary Fig. 22) of two studies (one RCT [47] and one non-RCT [48]) with a follow-up of ≥ 12 months for the steroid dose in the FRNS/SDNS + group revealed that the steroid dose in the RTX + MMF group was significantly lower (5.5 mg/m^2^/day) than that in the RTX + placebo or immunosuppressant-withdrawal groups (MD =  − 5.52, 95% CI −7.34 to − 3.70), equivalent to about 0.18 mg/kg/day, with a statistically significant difference.

In one non-RCT of RTX + MMF for the treatment of FRNS/SDNS + [49], there was no statistically significant difference in the steroid dose [standardized mean difference (SMD) = 0.5, 95% CI − 0.25 to 1.24] between RTX + MMF (*n* = 16, with the starting steroid dose at 0.38 mg/kg/day) and RTX + CsA (*n* = 13, with the starting steroid dose at 0.35 mg/kg/day) at follow-up ≥ 12 months.

MMF initiation timing varied across studies. Only one study [28] initiated MMF on the basis of B-cell reconstitution, specifically when CD20 + counts exceeded 10 cells/μL. One study [47] started MMF on day 29 after completing a four-dose RTX course, whereas another study [48] administered MMF to six out of nine patients after RTX, although without explicit reference to B-cell reconstitution. Other studies introduced MMF concurrently with or before RTX treatment. In one study [42], MMF was administered both before and during RTX, whereas in one study [48], three patients were already on MMF before RTX and continued it throughout and after treatment. One study [49] described MMF use being adjusted to maintain mycophenolic acid (MPA) levels post-RTX, suggesting prior MMF administration.

In one cohort study with two courses of RTX for the treatment of FRNS/SDNS + patients with a follow-up > 24 months [50], where the second course of RTX was started after B-cell reconstitution was achieved after the first course, the differences were statistically significant in both the relapse rate [93% (42/45) vs. 56% (9/16)] and the 50% relapse-free survival duration (335 days vs. 954 days), when the first course and the second course of RTX were compared.

#### Rationale: quality of life of children with FRNS/SDNS and parents

In one case series reporting on four doses of RTX (one dose every six months) + mizoribine (twice a week, 500 mg on day 1, and 550 mg on day 2) in FRNS/SDNS + patients (*n* = 22) with a follow-up of 24 months [51], 11 subjects did not relapse 12 months after the first dose of RTX, and 10 subjects did not relapse at 24 months. Statistically significant differences were shown in the frequency of relapse (one time/person/2 years vs. 5.8 times/person/2 years), Pediatric Quality of Life Inventory (PedsQL 4.0) [91.5 (95% CI 85.1–97.9) vs. 81.1 (95% CI 74.6–87.5)], and parent rated quality of life [85.2 (95% CI 78.8–91.7) vs. 74.9 (95% CI 68.5–81.3)] in the comparisons between those 24 months after RTX exposure and those before RTX.

### Recommendation 4: re-exposure to rituximab in patients with relapsing FRNS/SDNS + after rituximab intervention improves the relapse-free survival rate and prolongs relapse-free survival (1D)

Rationale: in one case series report of a second course of RTX (two doses) in the treatment of FRNS/SDNS + patients after relapse after the first course of RTX (1–2 doses) (*n* = 10), no relapse was observed at a follow-up ≥ 6 months [29]. In another case series report of RTX (1–3 courses) treatment in patients who relapsed FRNS/SDNS + (*n* = 5) after the first course of RTX (1–2 doses, median relapse time: 10 months), no relapse at the 24-month follow-up was observed [52]. A case series report of one dose of RTX for the treatment of relapsed FRNS/SDNS + patients (*n* = 39) after the first course of RTX (*n* = 46, 1–2 doses) reported a prolonged median time to relapse after immunosuppressant withdrawal between the reuse of RTX after relapse and the first course of RTX [8.5 (6.5, 11.7) months vs. 5.6 (4.3, 8.1) months] [37].

### Recommendation 5: peripheral blood CD19 + /CD20 + B-cell counts should be tested at least one month after rituximab treatment, with follow-up 5–6 months later (2D)

#### Rationale: B-cell depletion

At one week after the first dose of RTX for FRNS/SDNS, 88% of the children experienced B-cell depletion, and at one month, almost 100% of the children achieved B-cell depletion.

The pooled data (*n* = 59) from two CSRs [32, 53] and the RTX group of one RCT [19] with one dose of RTX revealed that complete B-cell depletion occurred at one month. Complete B-cell depletion also occurred at one month as per the analysis of a CSR [54] and the RTX group in one RCT [18] treated with four doses of RTX in children with FRNS/SDNS + (*n* = 39). Similarly, B-cell depletion was observed at one month in the RTX arm of an RCT on RTX treatment in FRNS/SDNS − patients (*n* = 15) [21]. In the meta-analysis of three studies on RTX in the treatment of FRNS/SDNS + patients (two CSRs [32, 55] and one cohort study [42]), B-cell depletion reached 88% (95% CI: 74%–95%) at one week after a single dose and the first of four doses of RTX (Supplementary Fig. 23).

#### Rationale: relapse rate during B-cell depletion

In the meta-analysis on the relapse rate of nine studies (three CSRs [33, 44, 56] and the RTX arms in four RCTs [20–23], one non-RCT [49], and one cohort study [42]) in FRNS/SDNS patients treated with RTX and resulting in B-cell depletion (Supplementary Fig. 24), the relapse rate (11/280) was 1% (95% CI: 0%–18%).

In two cohort studies on B lymphocyte subsets followed up in FRNS/SDNS for 12 months after RTX treatment [33, 57], between the group with relapse and the group without relapse, memory B cells presented statistically significant differences after RTX administration [(40.7 ± 16.7)/SDNS vs. (12.7 ± 4.5)/μL [57], 30 (25, 41)/μL vs. 18 (8, 27)/μL [33]], but there was no such difference before RTX administration. Th17 cells also significantly differed after RTX administration [(12.1 ± 5.2)/μL vs. (6.9 ± 2.0)/μL [57], 12 (7, 23)/μL vs. 6 (4, 10)/μL [33]).

In a cohort study on B lymphocyte subsets followed up for 12 months after RTX treatment in FRNS/SDNS [32], there are no statistically significant differences in memory B cells [2.6% (1.6%, 3.8%) vs. 2.9% (0.9%, 4.4%)], immunoglobulin M (IgM) memory B cells [1.17% (0.76%, 3.04%) vs. 1.37% (0.42%, 2.55%)], and switched memory B cells [1.31% (0.62%, 3.14%) vs. 0.98% (0.29%, 1.6%)]; however, no significant differences in memory B cells (0.6% ± 0.18%% vs. 0.32% ± 0.08%) were detected after RTX administration, but significant differences in IgM memory B cells (0.32% ± 0.05% vs. 0.16% ± 0.04%) and switched memory B cells (0.38% ± 0.1% vs. 0.08% ± 0.01%) were detected after RTX administration. Multivariable analysis revealed that the reconstitution of switched B cells was an independent risk factor for FRNS/SDNS relapse (HR = 3.45, 95% CI: 1.39–8.54).

#### Rationale: B-cell reconstitution occurs at 5–6 months after rituximab treatment in children with FRNS/SDNS

The tests for B-cell reconstitution were performed one week after RTX administration and monthly thereafter, with a follow-up period of ≥ 12 months. (1) Peripheral CD19 + /CD20 + B-cell count > 5 cells/μL or > 1% of total lymphocytes: in a meta-analysis of RTX in FRNS/SDNS + patients from five studies (two CSRs [31, 44] and the RTX arms in one RCT [19] with a single course in both groups, one non-RCT [49], and one cohort study [50]), the median time to B-cell reconstitution was 5.5 (95% CI: 5.1–5.9) months (Supplementary Figs. 25–26). In another meta-analysis of two RCTs [18, 47] on FRNS/SDNS with four doses of RTX (combined with steroids, MMF or placebo), the median time to B-cell reconstitution was 5.0 (95% CI: 4.5–5.7) months (Fig. 27 in Annex 1). (2) CD19 + /CD20 + B-cell count > 15 cells/μL or > 3% of total lymphocytes: a meta-analysis of three studies with RTX in FRNS/SDNS (one RCT [21], one non-RCT [48], and one CSR [58]) (Supplementary Figs. 28–29) reported a median time to B-cell reconstitution of 5.0 (95% CI: 4.4–5.6) months. One case series report with RTX in FRNS/SDNS + patients [53] reported the mean time to B-cell reconstitution at 4.4 months. (3) CD19 + /CD20 + B-cell count > 10 cells/μL: in one case series report of RTX in the treatment of FRNS/SDNS + patients [35], the median time to B-cell reconstitution was 5.8 (4.0–12) months.

#### Rationale: slightly prolonged duration of B-cell depletion after multiple courses of rituximab treatment

Peripheral CD19 + /CD20 + B-cell count > 5 cells/μL or > 1% of total lymphocytes: in one cohort study comparing two courses of RTX (*n* = 16) with the first course of RTX (*n* = 45) in FRNS/SDNS + patients [50], the median time of B-cell depletion after two courses of RTX was longer than that after the first course of treatment [8.0 (5.7–9.2) months vs. 5.8 (4.8–7.3) months]. In one case series reporting on repeated courses of RTX in the treatment of FRNS/SDNS + patients due to relapse or maintenance therapy [59], 346 patients (with 1149 courses of treatment) experienced a median follow-up of 5.9 (4.3, 7.7) years. Among the 632 courses monitored for B cells, 622 (98.4%) averaged the median time to B-cell reconstitution at 6.1 (95% CI 6.0–6.3) months. A total of 70% (435/622) of the patients experienced relapse with a median time of 2.6 (0.2, 10.0) months after B-cell reconstitution;

CD19 + /CD20 + B-cell count > 15 cells/μL or > 3% of total lymphocytes: in a case series report on maintenance therapy (*n* = 5) by three-dose RTX in FRNS/SDNS + after three-dose (first course) RTX treatment and reconstitution of B cells with a follow-up > 12 months [60], all B cells were depleted after the first course of treatment, with a mean depletion time of (7.0 ± 1.0) months, and all the periods of B-cell depletion lasted > 15 months after maintenance therapy.

CD19 + /CD20 + B-cell count > 10 cells/μL: in one case series report of maintenance therapy (*n* = 22) with RTX (one-to-four doses) in FRNS/SDNS + after RTX (one-to-four doses in first course) treatment and B-cell depletion with a follow-up period > 12 months [54], all B cells of the patients were depleted after the first course of RTX. In one case series on the treatment of FRNS/SDNS + patients with four courses of RTX (once every three months) [61], the B-cell depletion rate after the first course of RTX was 80% (4/5), with the B-cell depletion period of the four subjects lasting > 21 months during the follow-up, with a median of 3.2 (1.9, 3.8) years.

### Recommendation 6: rituximab treatment does not increase the incidence of serious adverse events or infections in children with SSNS (1D)

Rationale: in the meta-analysis of four studies [18, 20, 22, 23] on RTX treatment of SSNS, no statistically significant difference was observed in the incidence of serious adverse events, regardless of the comparison between the RTX group and the placebo or conventional immunosuppressant control group (14/140 vs. 9/122) (Supplementary Fig. 30), between the RTX group and the placebo control subgroup, or between the RTX group and the conventional immunosuppressant control subgroup.

In the meta-analysis of seven studies [18–20, 22, 23, 25, 26] on RTX for the treatment of SSNS, no statistically significant difference was observed in the incidence of adverse events, regardless of the comparison between the RTX group and the placebo or conventional immunosuppressant control group (130/199 vs. 107/192) (Supplementary Fig. 31), the comparison between the RTX group and the placebo control subgroup, or the RTX group and the conventional immunosuppressant control subgroup.

In one RCT [22] on SSNS treated with RTX for separate reports of drug-related adverse reactions, the difference in drug-related adverse reactions between RTX and tacrolimus was statistically significant (10/20 vs. 17/20); the difference in definite drug-related adverse reactions (9/20 vs. 7/20) was not statistically significant, but the difference in possible drug-related adverse reactions (3/20 vs. 17/20) was statistically significant.

In a multicenter RCT [18], there was a statistically significant difference in the incidence of neutropenia (grades 3–4) between the RTX group (4/24) and the placebo group (0/24) in children with SSNS (OR = 8.47, 95% CI: 1.12–64.20). In a single-center RCT [22], there was no statistically significant difference in the incidence of neutropenia between the RTX group (1/20) and the tacrolimus group (0/20) in children with SSNS (OR = 7.39, 95% CI 0.15–372.38).

The meta-analysis of the incidence of neutropenia (19/369) in 11 CSRs [11, 37, 40, 49, 62–68] of RTX-treated pediatric patients with SSNS revealed that the incidence of neutropenia was 5% (95% CI 3%–8%) (Supplementary Fig. 32).

In a multicenter RCT [18], there was no statistically significant difference in the incidence of lymphopenia between the RTX group (4/24) and the placebo group (4/24) in children with SSNS (OR = 1.00, 95% CI 0.22–4.49). In a single-center RCT [22], there was no statistically significant difference in the incidence of lymphopenia between the RTX group (0/20) and the tacrolimus group (1/20) in children with SSNS (OR = 0.14, 95% CI 0.00–6.82).

In a multicenter RCT [18], there was no statistically significant difference in the incidence of infection between the RTX group (23/24) and the placebo group (18/24) in children with SSNS (OR = 7.67, 95% CI 0.85–69.54). Among the two [22, 23] RCTs, the meta-analysis of the incidence of infections in children with SSNS revealed no statistically significant difference (OR = 2.83, 95% CI 0.69–11.62) between the RTX group (33/56) and the conventional immunosuppressant control group (23/38) (Supplementary Fig. 33).

In a meta-analysis of the incidence of hypogammaglobulinemia (104/393) reported in 11 CSRs [9, 11, 40, 42, 49, 52, 60, 67–70] in RTX-treated pediatric patients with SSNS, the mean incidence was 26% (95% CI 22%–31%) (Supplementary Fig. 34). In two of the target studies of hypogammaglobulinemia [9, 68], the incidence was 51% (56/110, 95% CI 42%–60%). In three of the studies [9, 69, 71], the incidence of persistent hypogammaglobulinemia was 13% (34/257, 95% CI 10%–18%) (Supplementary Fig. 35).

The meta-analysis of the ARA detection rate in five CSRs [10, 11, 56, 66, 72] of RTX-treated pediatric patients with SSNS revealed an ARA detection rate of 16% (15/96, 95% CI 10%–24%) (Supplementary Fig. 36). The meta-analysis of the incidence of serum sickness reported in three CSRs [37, 65, 72] of RTX-treated pediatric patients with SSNS revealed that the incidence of serum sickness was 5% (6/129, 95% CI 2%–10%) (Supplementary Fig. 37).

The meta-analysis of the incidence of infusion-related reactions reported in 21 CSRs [7, 11, 30, 34, 42, 49, 52–54, 56, 58, 60, 61, 63, 65, 73–77] in children with SSNS treated with RTX revealed that the incidence of the reaction was 31% (269/856, 95% CI 28%–35%) (Supplementary Fig. 38).

Measures to prevent RTX-induced adverse reactions in children with SSNS were taken in three periods: before, during, and after RTX infusion. Prophylaxis before RTX infusion in 843 children across 32 studies: oral administration of paracetamol (725 patients in 26 studies), oral/intravenous steroids (733 in 26 studies, including methylprednisolone for 589 patients in 19 studies, hydrocortisone for 79 in four studies, dexamethasone for 35 in two studies, and betamethasone for 30 in one study), oral/intravenous/intramuscular antihistamines (811 in 30 studies, including chlorphenamine for 488 subjects in 16 studies, diphenhydramine for 201 in nine studies, cetirizine for 70 in one study, promethazine for 35 in two studies, and cyproheptadine for 17 in two studies). Prophylactic medication during RTX infusion was conducted in 147 children in eight studies, with control of the infusion speed and drug concentration, as well as close monitoring during infusion. Post-RTX infusion prophylaxis was conducted in 247 children in 12 studies by oral medication of sulfamethoxazole and/or trimethoprim tablets to prevent *Pneumocystis carinii* pneumonia.

### Recommendation 7: we recommend considering rituximab as a cost-neutral treatment option for children with SSNS, as it does not appear to increase total medical expenditure compared with other treatment approaches (1D)

Rationale: this single-center retrospective cohort study [27] for cost-effectiveness analysis of ≥ 12-month follow-up in children aged 2–21 years with SDNS treated with RTX (*n* = 10) and CNI (*n* = 8) revealed the following: (1) No statistically significant difference of cost (USD $) in medical expenditure for remission maintenance between RTX (16,583 ± 2492) and CNI (18,036 ± 3482) (*P* = 0.31). Among the fees, there was no statistically significant difference in hospitalization cost [RTX (5052 ± 1377) vs. CNI (9420 ± 9256)] or outpatient cost [RTX (4384 ± 4939) vs. CNI (7535 ± 1107)], whereas there was a statistically significant difference in drug cost [RTX (7148 ± 1,01) vs. CNI (1082 ± 431), *P* < 0.001]. With the adjustment of the mean duration of remission maintenance to 12 months, the annual medical expenditure for remission maintenance still showed no statistically significant difference between the RTX group (9703 ± 1754) and the CNI group (23,732 ± 9835) (*P* = 0.24). (2) Neither the annual relapse frequency [RTX (0.70 ± 0.80) vs. CNI (0.83 ± 0.78); *P* > 0.05] nor the duration (months) of remission [RTX (10.10 ± 4.9) vs. CNI (9.12 ± 7.40); *P* = 0.72] indicated a statistically significant difference of efficacy between the two groups, whereas both the serum albumin (g/L) level and the urine protein (mg)-to-creatinine (mg) ratio during treatment demonstrated statistically significant differences between the two groups [RTX (37.4 ± 3.0) vs. CNI (34.2 ± 2.0), *P* = 0.03; RTX (0.23 ± 0.19) vs. CNI (0.77 ± 0.73), *P* = 0.04], respectively.

A single-center self-controlled study [78] in 30 patients with SSNS (five with onset in childhood and 25 with onset in adulthood) included the pre-RTX stage and the RTX treatment stage. For pretreatment, various immunosuppressant(s) were used for 17.8 ± 13.7 months; for the later stage, RTX treatment was used for 29.8 ± 2.6 months. (1) The average monthly medical expenditures presented no statistically significant difference in cost (USD $) between the first 24 months (2923 ± 5803) and the second 24 months (1280 ± 578) (*P* = 0.06), even in terms of hospitalization expenses (*P* = 0.05) and outpatient expenses (*P* = 0.16). (2) Between the first 24 months and the second 24 months, statistically significant differences occurred in the relapse rate (30/30 vs. 6/30), relapse frequency (4.30 ± 2.76 vs. 0.27 ± 0.52), steroid dose (mg/day) (24.1 ± 13.4 vs. 0.2 ± 0.6), and cyclosporine dose (mg/day) (89.8 ± 64.5 vs. 12.5 ± 29.1) (*P* < 0.01 for all). (3) For cost-effectiveness, both the medical expenses and the relapse frequency decreased after RTX treatment, $70,155 with relapse for 4.3 times in the first 24 months vs. $30,726 with relapse for 0.27 times in the second 24 months.

## Limitations

Most of these studies address only full relapse, as defined in the manuscript, but do not consider the time to partial relapse, for example, a proteinuria of 1 + to 2 + or a persistent urine protein:creatinine ratio > 0.02 g/mmol but less than the nephrotic range.

We included RCTs and quasi-RCTs as well as cohort studies in the meta-analysis. Perhaps, the analysis would be stronger if it was limited to RCTs and quasi-RCTs.

## Future research directions

Optimal RTX dosing and regimens: while our guidelines provide recommendations on dosing, the evidence base for optimal dosing (single-dose vs. multiple-dose, fixed-dose vs. body surface area-based dosing) remains limited, particularly concerning long-term efficacy and safety. Comparative effectiveness trials are needed to compare different RTX protocols head-to-head.

Long-term outcomes and growth: prospective, long-term follow-up studies are crucial. This includes investigating immune function (vaccination responses), infection risk (e.g., late-onset neutropenia, hypogammaglobulinemia), and potential complications associated with RTX. Additionally, growth monitoring (height, weight, growth velocity, and bone age assessment) and proactive interventions (nutritional counseling and careful steroid management) are crucial for mitigating treatment-related effects.

Biomarkers and predictors of response: identifying reliable biomarkers that can predict response to RTX would be highly valuable. This would allow for personalized treatment approaches, avoiding unnecessary RTX exposure in patients unlikely to benefit.

Concomitant medications: the optimal use of concomitant medications (e.g., steroids, CNI, and mycophenolate mofetil) in conjunction with RTX needs further clarification. Research should investigate the best strategies for tapering or discontinuing these medications after RTX administration to minimize side effects while maintaining remission.

Cost-effectiveness: formal cost-effectiveness analyses of different RTX regimens, compared with alternative maintenance therapies, are needed to inform healthcare resource allocation, especially in resource-limited settings.

## Supplementary Information

Below is the link to the electronic supplementary material.

Supplementary file1 (PDF 6590 KB)

## Data Availability

The authors confirm that the data supporting the findings of this study are available within the article and supplementary materials.
