# Supplementary material for: Clinical practice guidelines for rituximab treatment in children with steroid-sensitive nephrotic syndrome
Source: World J Pediatr. 2025 Aug 13;21(8):775–91. doi: 10.1007/s12519-025-00957-9 (PMC12380898; doi:10.1007/s12519-025-00957-9)
Supplement: Supplementary file 1 — Supplementary file1 (PDF 6590 KB) [file 12519_2025_957_MOESM1_ESM.pdf]

Supplementary material

Annex 1 Meta-analysis Figure of the RTX Guidelines

Annex 2 Planning Proposal of the Guidelines

Annex 3 Selection of Clinical Topics

Annex 4 Search Query in Chinese and English

Annex 5 Literature Bias Risk Assessment Table

Annex 6 GRADE Evidence Profile

Annex 7 Guideline EtD Table

Meta-analysis Figure of the Rituximab Guidelines

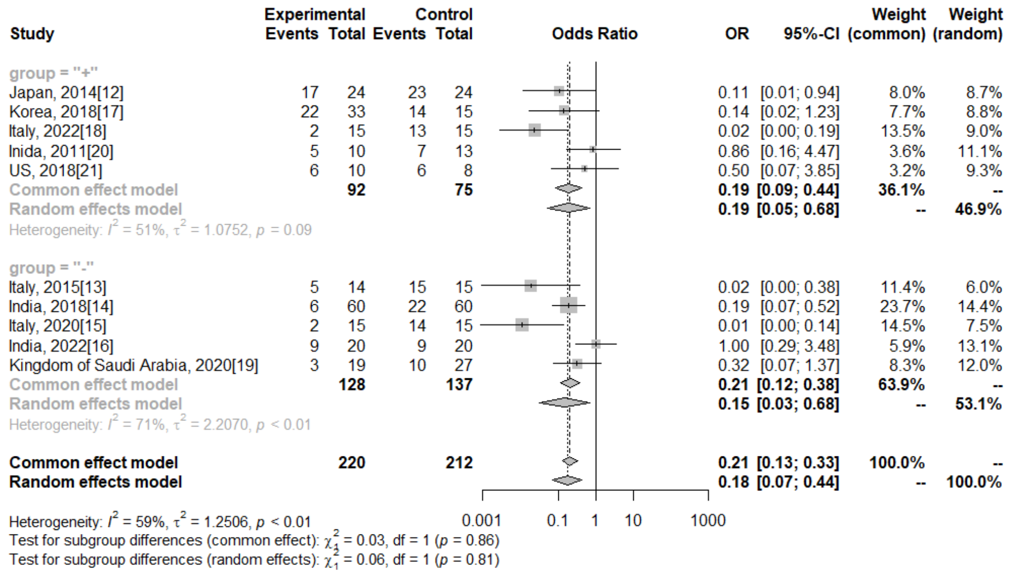

Figure 1 Meta-analysis of 1-year relapse rate of RTX treatment compared with the control group in the different FRNS/SDNS subgroups

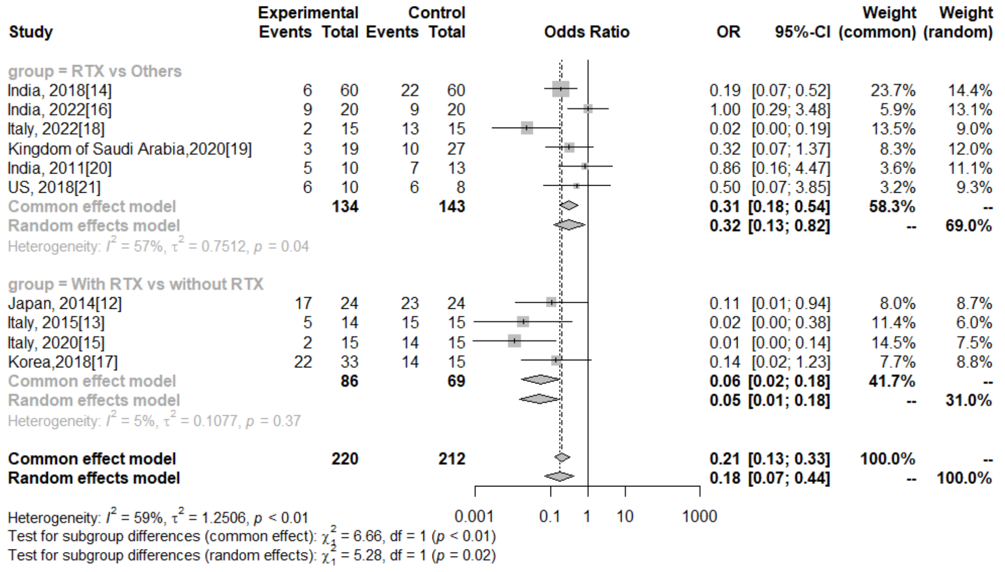

Figure 2 Meta-analysis of 1-year relapse rate of RTX treatment in FRNS/SDNS compared with the different control groups

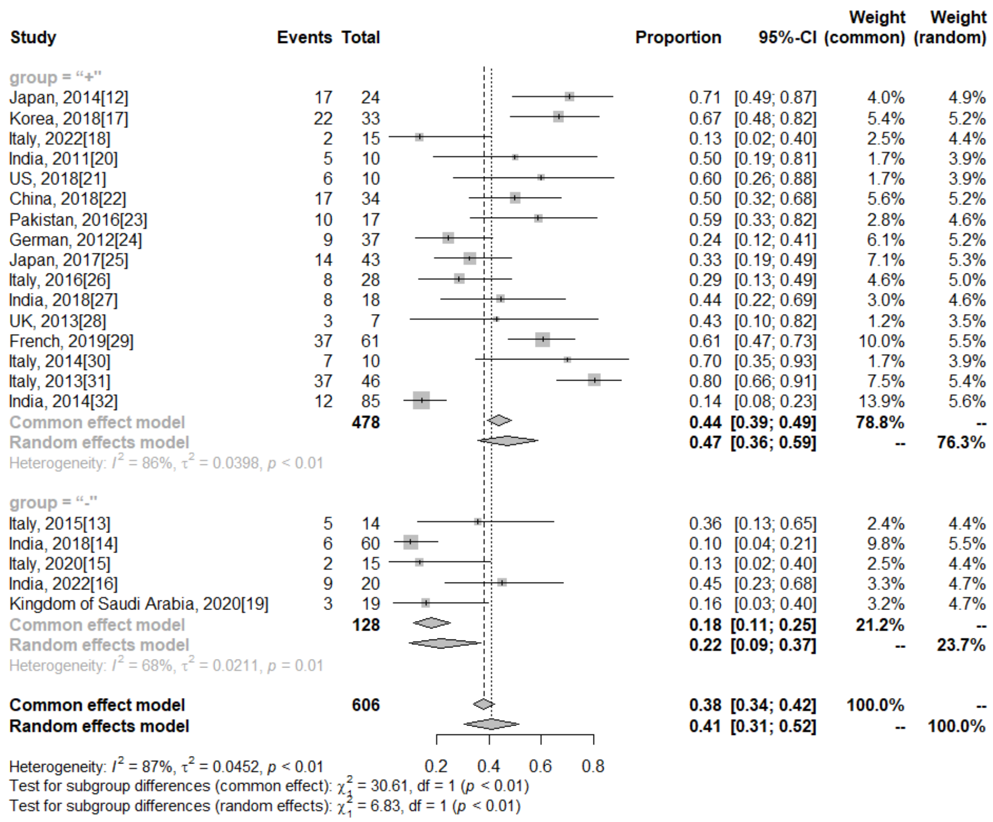

Figure 3 Meta-analysis of 1-year relapse rate of RTX treatment in the different FRNS/SDNS subgroups

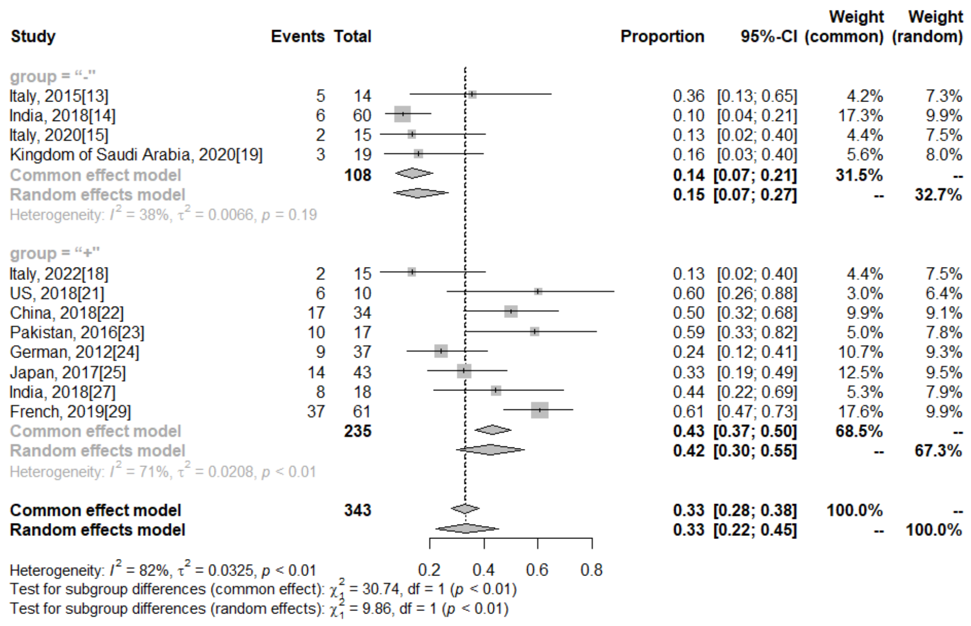

Figure 4 Meta-analysis of 1-year relapse rate of 1-2 doses of RTX treatment in the different FRNS/SDNS subgroups

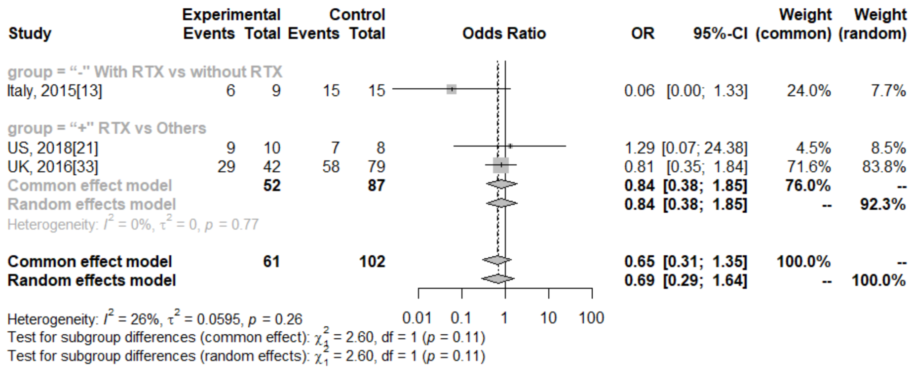

Figure 5 Meta-analysis of 2-year relapse rate of RTX treatment compared with the different control groups in the different FRNS/SDNS subgroups

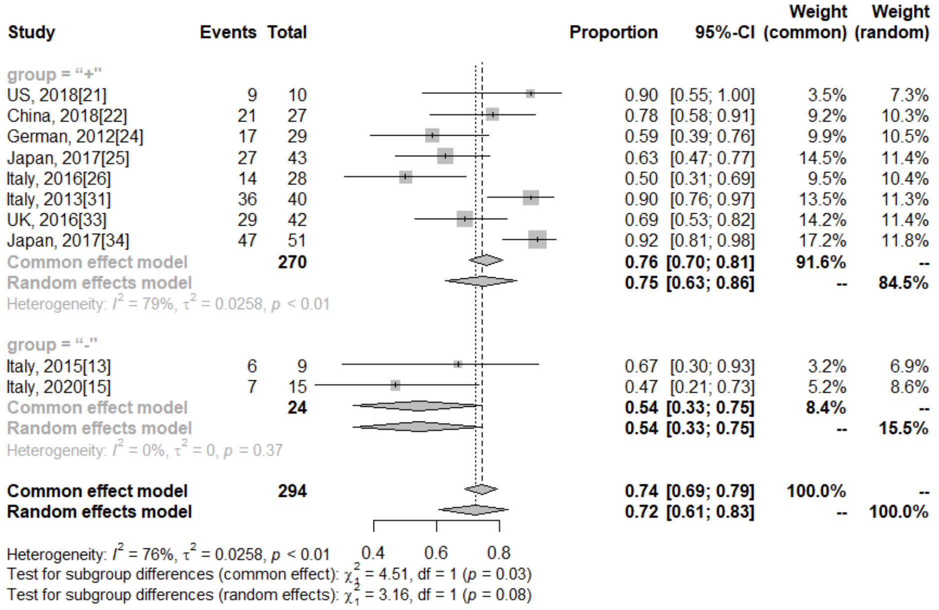

Figure 6 Meta-analysis of 2-year relapse rate of RTX treatment in the different FRNS/SDNS subgroups

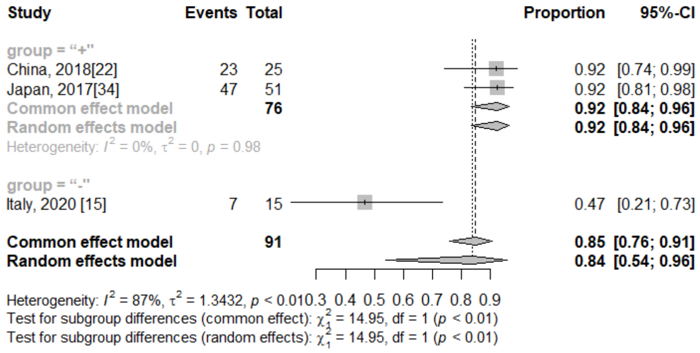

Figure 7 Meta-analysis of 3-year relapse rate of RTX treatment in the different FRNS/SDNS subgroups

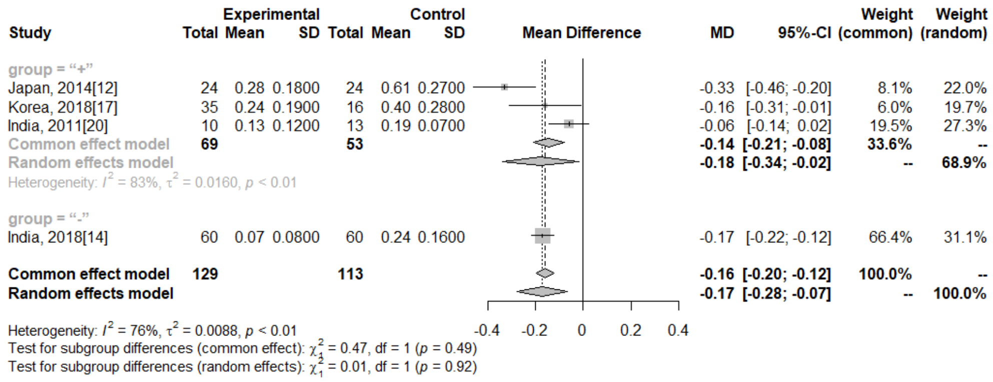

Figure 8 Meta-analysis of 1-year cumulative steroid dose of RTX treatment compared with the control group in the different FRNS/SDNS subgroups

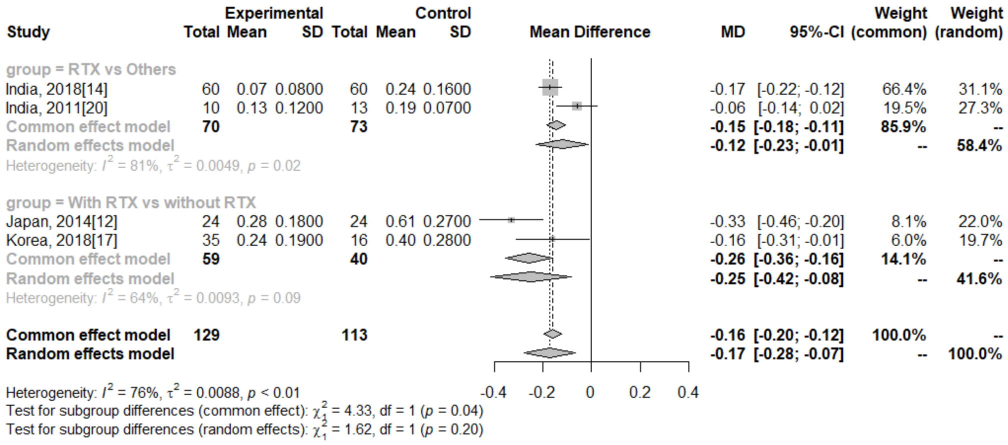

Figure 9 Meta-analysis of 1-year cumulative steroid dose of RTX treatment in FRNS/SDNS compared with the different control groups

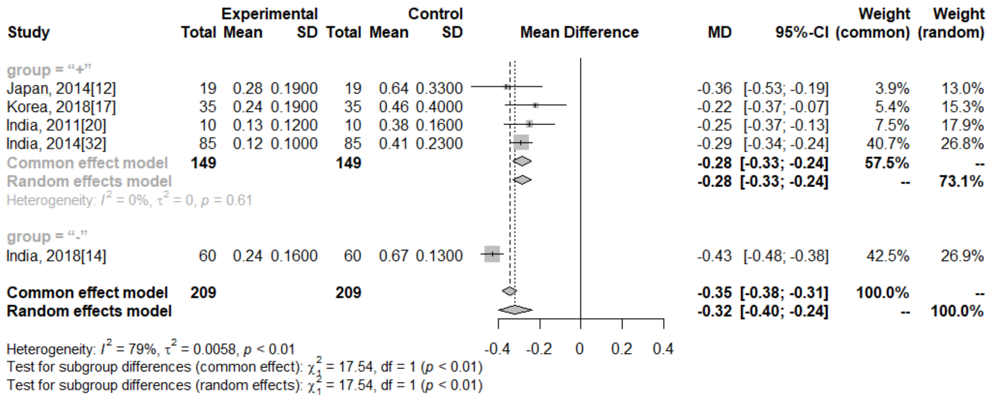

Figure 10 Meta-analysis of 1-year cumulative steroid dose of pre- and post- RTX treatment in the different FRNS/SDNS subgroups

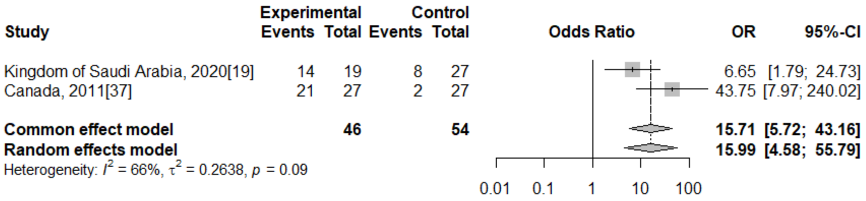

Figure 11 Meta-analysis of 3-month steroid withdrawal rate of RTX treatment compared with other immunosuppressants in FRNS/SDNS

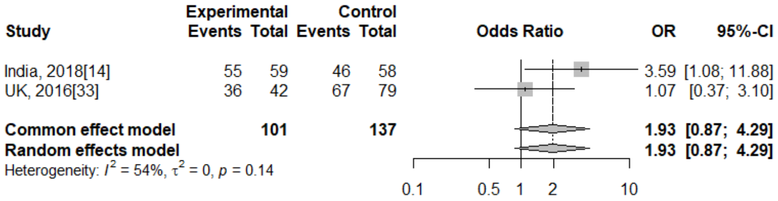

Figure 12 Meta-analysis of 1-year steroid withdrawal rate of RTX treatment compared with other immunosuppressants in FRNS/SDNS

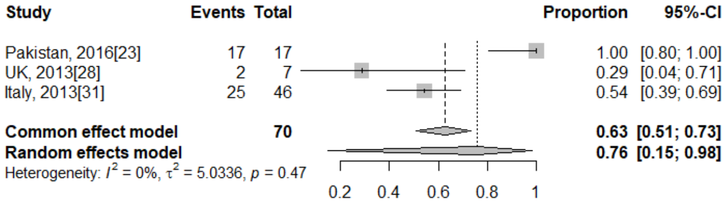

Figure 13 Meta-analysis of 6-month steroid withdrawal rate of RTX treatment in FRNS/SDNS+

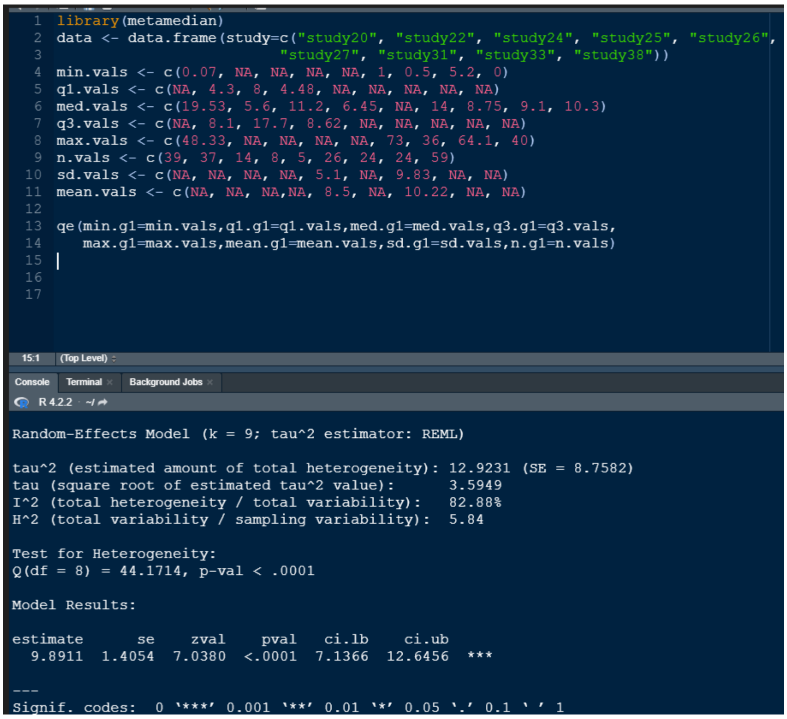

```
library(metamedian)
data <- data.frame(study=c("study20", "study22", "study24", "study25", "study26",
                          "study27", "study31", "study33", "study38"))
min.vals <- c(0.07, NA, NA, NA, NA, 1, 0.5, 5.2, 0)
q1.vals <- c(NA, 4.3, 8, 4.48, NA, NA, NA, NA, NA)
med.vals <- c(19.53, 5.6, 11.2, 6.45, NA, 14, 8.75, 9.1, 10.3)
q3.vals <- c(NA, 8.1, 17.7, 8.62, NA, NA, NA, NA, NA)
max.vals <- c(48.33, NA, NA, NA, NA, 73, 36, 64.1, 40)
n.vals <- c(39, 37, 14, 8, 5, 26, 24, 24, 59)
sd.vals <- c(NA, NA, NA, NA, 5.1, NA, 9.83, NA, NA)
mean.vals <- c(NA, NA, NA,NA, 8.5, NA, 10.22, NA, NA)

qe(min.g1=min.vals,q1.g1=q1.vals,med.g1=med.vals,q3.g1=q3.vals,
   max.g1=max.vals,mean.g1=mean.vals,sd.g1=sd.vals,n.g1=n.vals)
```

15:1 (Top Level)

Console Terminal Background Jobs

R 4.2.2 ~/

```
Random-Effects Model (k = 9; tau^2 estimator: REML)

tau^2 (estimated amount of total heterogeneity): 12.9231 (SE = 8.7582)
tau (square root of estimated tau^2 value):      3.5949
I^2 (total heterogeneity / total variability):   82.88%
H^2 (total variability / sampling variability):  5.84

Test for Heterogeneity:
Q(df = 8) = 44.1714, p-val < .0001

Model Results:

estimate      se    zval    pval   ci.lb    ci.ub
  9.8911  1.4054  7.0380  <.0001  7.1366  12.6456  ***

---
Signif. codes:  0 '***' 0.001 '**' 0.01 '*' 0.05 '.' 0.1 ' ' 1
```

Figure 14 Processes of meta-analysis of the median time to first relapse of RTX treatment in FRNS/SDNS+ with a follow-up of ≥ 12 months

Method	Pooled effect size			Heterogeneity evaluation		
	point estimate	95%CI lower bound	95%CI upper bound	τ^2	I^2	Q
QE	9.89	7.14	12.65	12.92	82.88%	44.17

Figure 15 Results of meta-analysis of the median time to first relapse of RTX treatment in FRNS/SDNS+ with a follow-up of ≥ 12 months

Studies included: 3
Participants included: Unknown

Meta-analysis pooling of aggregate data
using the fixed-effect inverse-variance model

group and study	exp(b)	[95% Conf. Interval]		% Weight
RTX vs Others				
India, 2018[14]	1.370	1.117	1.680	73.60
Kindom of Saudi Arabia, 2020[19]	0.913	0.530	1.572	10.40
Subgroup effect	1.303	1.076	1.577	84.00
With RTX vs Without RTX				
Italy, 2015[13]	15.333	9.892	23.767	16.00
Subgroup effect	15.333	9.892	23.767	16.00
Overall effect	1.933	1.622	2.303	100.00

Tests of effect size = 1:

RTX vs Others	z = 2.710	p = 0.007
With RTX vs Without RTX	z = 12.209	p = 0.000
Overall	z = 7.368	p = 0.000

Cochran Q statistics for heterogeneity

	Value	df	p-value
RTX vs Others	1.87	1	0.171
With RTX vs Without RTX	0.00	0	.
Overall	104.00	2	0.000
Between	102.13	1	0.000
Between:Within (F)	54.48	1, 1	0.086

Figure 16 Meta-analysis of the median time to first relapse of FRNS/SDNS- treated with 1-2 doses RTX compared with the different control groups at 12-month follow-up

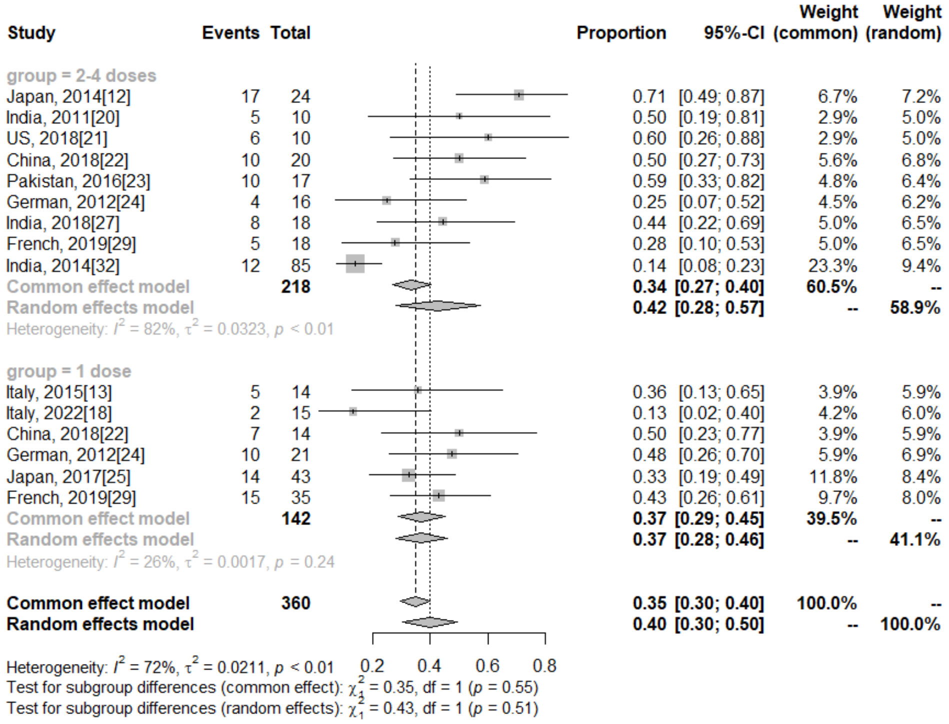

Figure 17 Meta-analysis of 1-year relapse rate of first course of RTX treatment at dose of 375 mg·m^{-2} in FRNS/SDNS+ treated with 1 or 2-4 doses

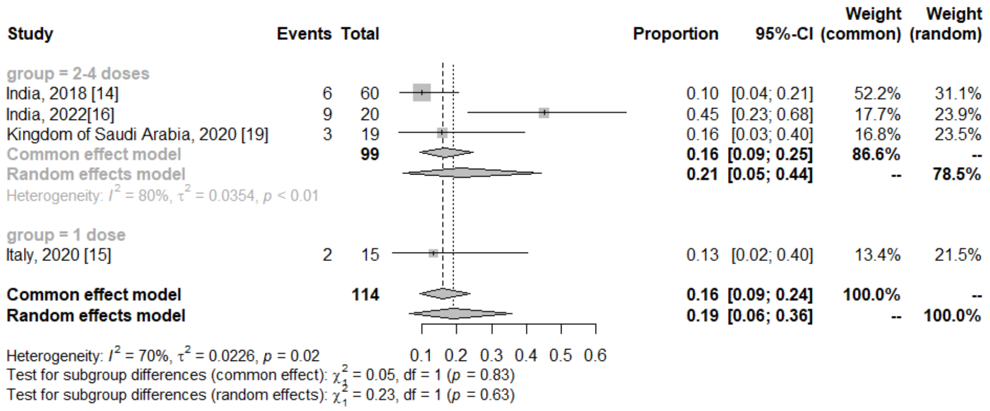

Figure 18 Meta-analysis of 1-year relapse rate of first course of RTX treatment at dose of 375 mg·m^{-2} in FRNS/SDNS- treated with 1 or 2-4 doses

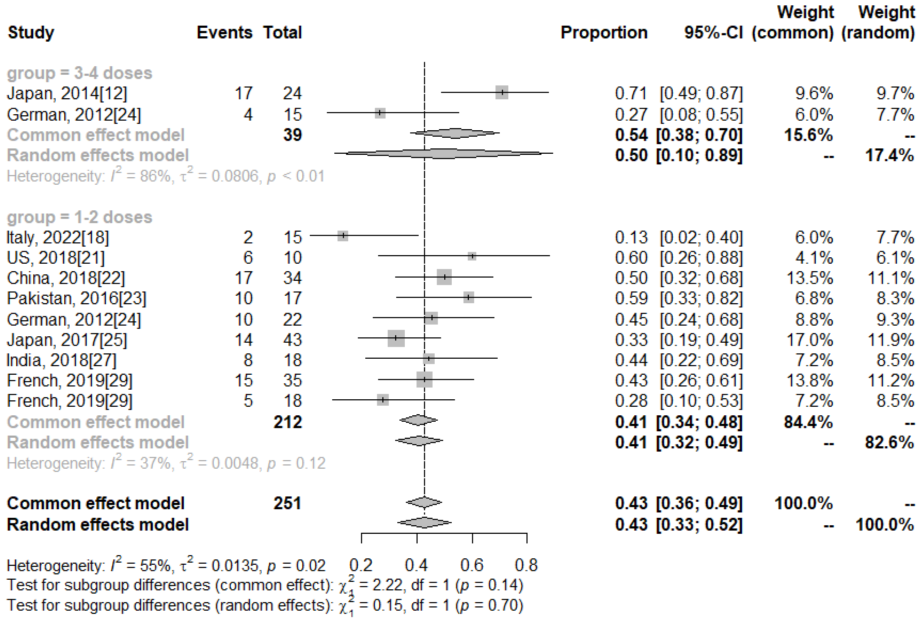

Figure 19 Meta-analysis of 1-year relapse rate of first course of RTX treatment at dose of 375 mg·m^{-2} in FRNS/SDNS+ treated with 1-2 or 3-4 doses

Studies included: 2
Participants included: Unknown

Meta-analysis pooling of aggregate data
using the fixed-effect inverse-variance model

study	exp(b)	[95% Conf.	Interval]	% Weight
China, 2018[22]	5.000	3.351	7.460	29.27
Japan, 2022[41]	2.044	1.580	2.644	70.73
Overall effect	2.656	2.139	3.297	100.00

Test of overall effect = 1: z = 8.844 p = 0.000

Heterogeneity Measures

	Value	df	p-value
Cochran's Q	13.59	1	0.000
I² (%)	92.6%		
Modified H²	12.587		
tau²	0.3707		

I² = between-study variance (tau²) as a percentage of total variance
Modified H² = ratio of tau² to typical within-study variance

.

Figure 20 Meta-analysis of the time to first relapse of RTX + MMF in FRNS/SDNS

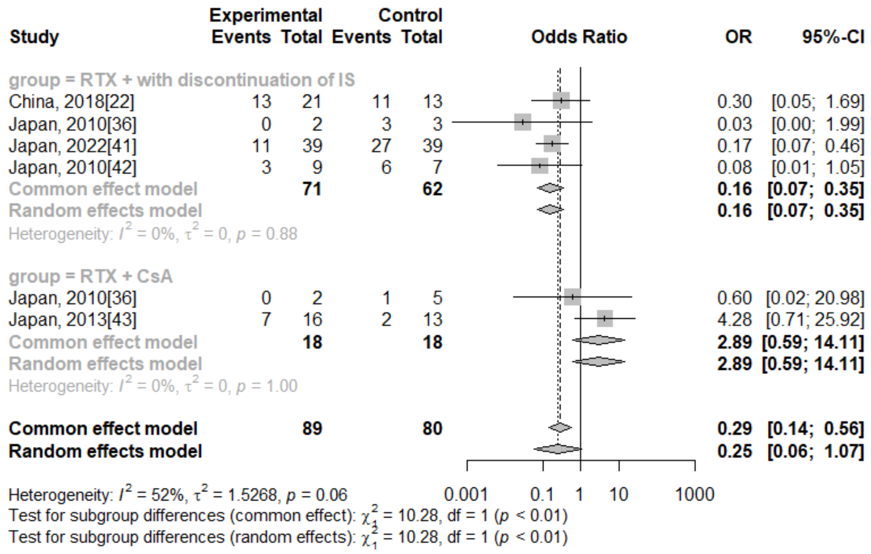

Figure 21 Meta-analysis of relapse rate of RTX + MMF compared with RTX + placebo/ immunosuppressant withdrawal or RTX + CsA in FRNS/SDNS+ with a follow-up of ≥ 12 months

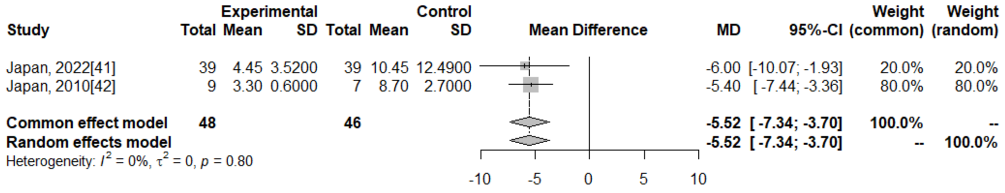

Figure 22 Meta-analysis of steroid dose of RTX + MMF compared with RTX + placebo/ immunosuppressant withdrawal in FRNS/SDNS+ with a follow-up of ≥ 12 months

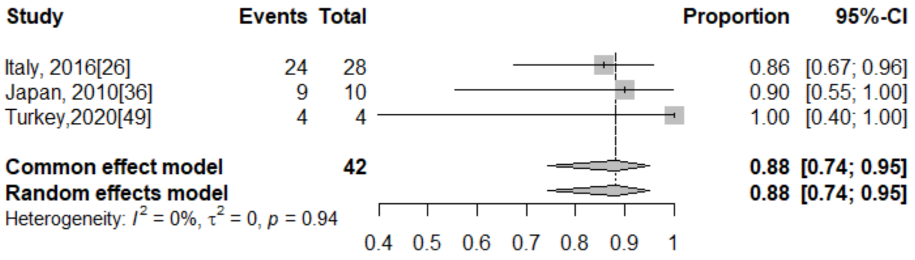

Figure 23 Meta-analysis of B-cell depletion rate at 1 week after the first dose of RTX treatment in FRNS/SDNS+

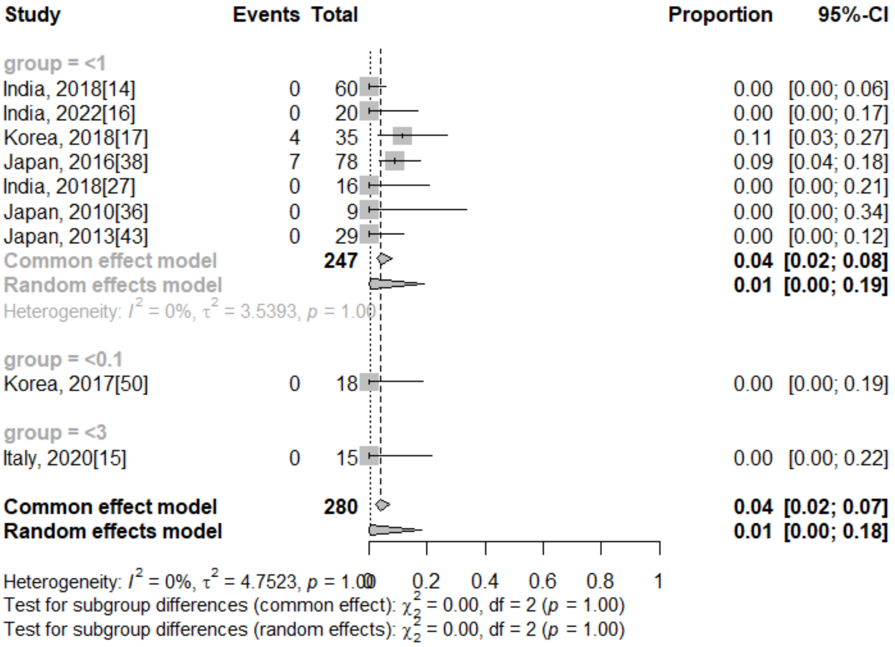

Figure 24 Meta-analysis of relapse rate of FRNS/SDNS treated with first course of RTX during B-cell depletion

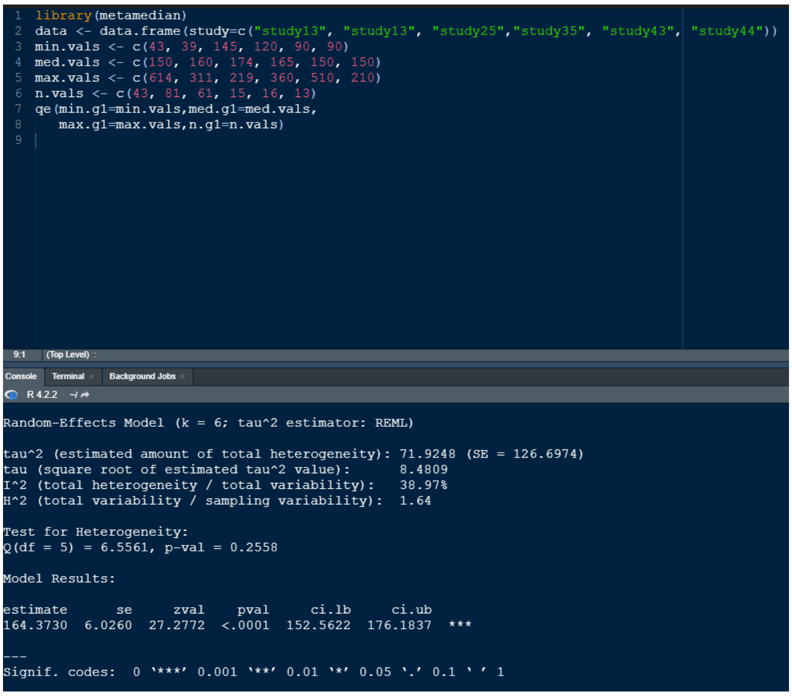
```
library(metamedian)
data <- data.frame(study=c("study13", "study13", "study25","study35", "study43", "study44"))
min.vals <- c(43, 39, 145, 120, 90, 90)
med.vals <- c(150, 160, 174, 165, 150, 150)
max.vals <- c(614, 311, 219, 360, 510, 210)
n.vals <- c(43, 81, 61, 15, 16, 13)
qe(min.g1=min.vals,med.g1=med.vals,
   max.g1=max.vals,n.g1=n.vals)

Random-Effects Model (k = 6; tau^2 estimator: REML)

tau^2 (estimated amount of total heterogeneity): 71.9248 (SE = 126.6974)
tau (square root of estimated tau^2 value):      8.4809
I^2 (total heterogeneity / total variability):   38.97%
H^2 (total variability / sampling variability):  1.64

Test for Heterogeneity:
Q(df = 5) = 6.5561, p-val = 0.2558

Model Results:

estimate      se     zval    pval     ci.lb     ci.ub
164.3730  6.0260  27.2772  <.0001  152.5622  176.1837  ***

---
Signif. codes:  0 '***' 0.001 '**' 0.01 '*' 0.05 '.' 0.1 ' ' 1
```

Figure 25 Processes of meta-analysis of the median time to B-cell reconstitution(>1%) of s single dose of RTX treatment in FRNS/SDNS+

Method	Pooled effect size			Heterogeneity evaluation		
	Point estimate	95%CI lower bound	95%CI upper bound	τ^2	I^2	Q
QE	164.37	152.56	176.18	71.92	38.97%	6.56

Figure 26 Results of meta-analysis of the median time to B-cell reconstitution(>1%) of s single dose of

RTX treatment in FRNS/SDNS+

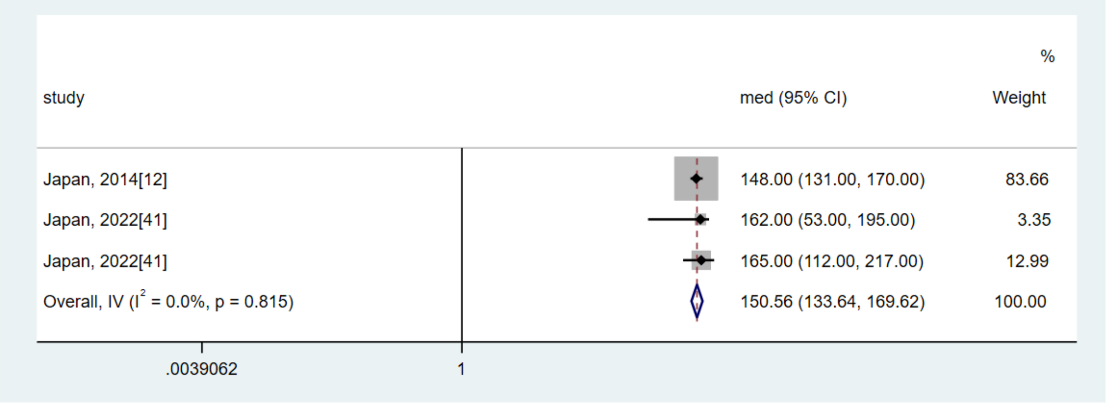

Figure 27 Meta-analysis of the median time to B-cell reconstitution(>1%) of 4 doses of RTX treatment in FRNS/SDNS

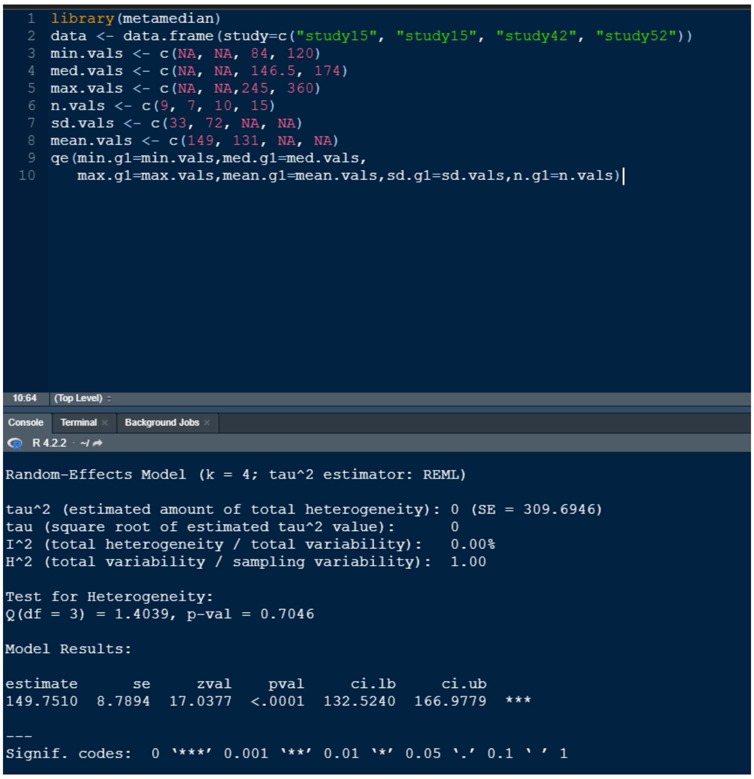

```
library(metamedian)
data <- data.frame(study=c("study15", "study15", "study42", "study52"))
min.vals <- c(NA, NA, 84, 120)
med.vals <- c(NA, NA, 146.5, 174)
max.vals <- c(NA, NA,245, 360)
n.vals <- c(9, 7, 10, 15)
sd.vals <- c(33, 72, NA, NA)
mean.vals <- c(149, 131, NA, NA)
qe(min.g1=min.vals,med.g1=med.vals,
   max.g1=max.vals,mean.g1=mean.vals,sd.g1=sd.vals,n.g1=n.vals)
```

```
10:64  (Top Level) :
Console  Terminal  Background Jobs
R 4.2.2 · ~/

Random-Effects Model (k = 4; tau^2 estimator: REML)

tau^2 (estimated amount of total heterogeneity): 0 (SE = 309.6946)
tau (square root of estimated tau^2 value):      0
I^2 (total heterogeneity / total variability):   0.00%
H^2 (total variability / sampling variability):  1.00

Test for Heterogeneity:
Q(df = 3) = 1.4039, p-val = 0.7046

Model Results:

estimate      se     zval    pval     ci.lb     ci.ub
149.7510  8.7894  17.0377  <.0001  132.5240  166.9779  ***

---
Signif. codes:  0 '***' 0.001 '**' 0.01 '*' 0.05 '.' 0.1 ' ' 1
```

Figure 28 Processes of meta-analysis of the median time to B-cell reconstitution(>3%) of s single dose of RTX treatment in FRNS/SDNS

Method	Pooled effect size			Heterogeneity evaluation		
	Point estimate	95%CI lower bound	95%CI upper bound	τ^2	I^2	Q
QE	149.75	132.52	166.98	0	0%	1.41

Figure 29 Results of meta-analysis of the median time to B-cell reconstitution(>3%) of s single dose of RTX treatment in FRNS/SDNS

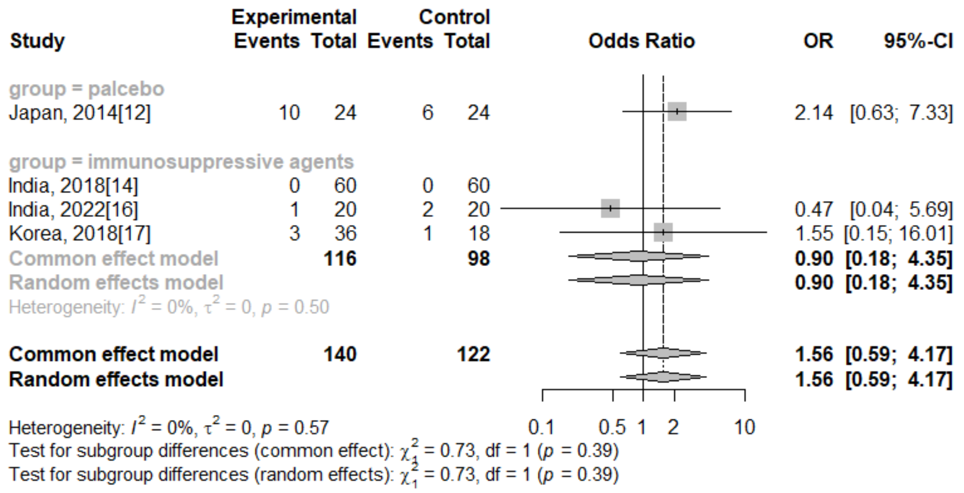

Figure 30 Meta-analysis of the incidence of serious adverse events of RTX treatment in SSNS compared with the different control groups

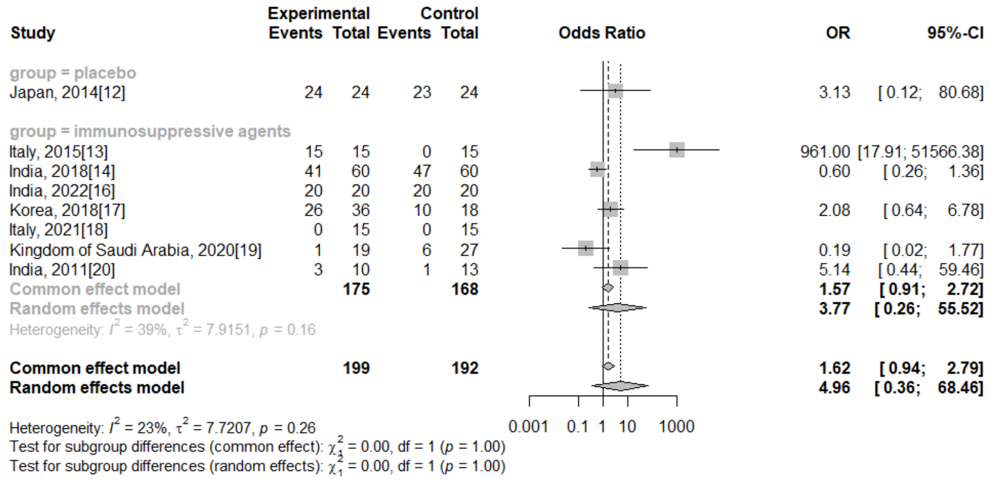

Figure 31 Meta-analysis of the incidence of adverse events of RTX treatment in SSNS compared with the different control groups

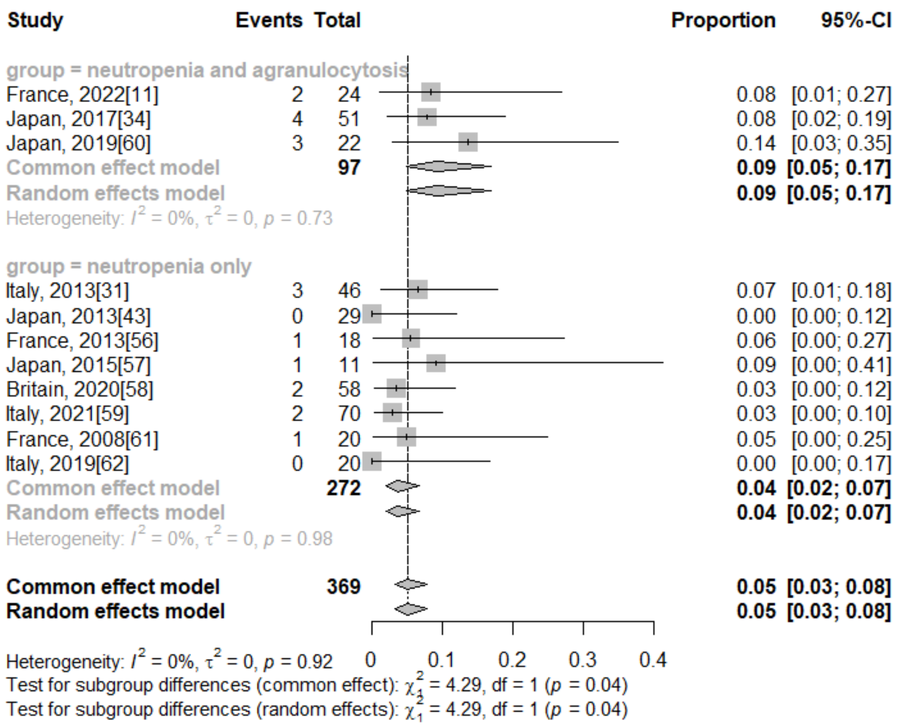

Figure 32 Meta-analysis of the incidence of neutropenia of RTX treatment in SSNS

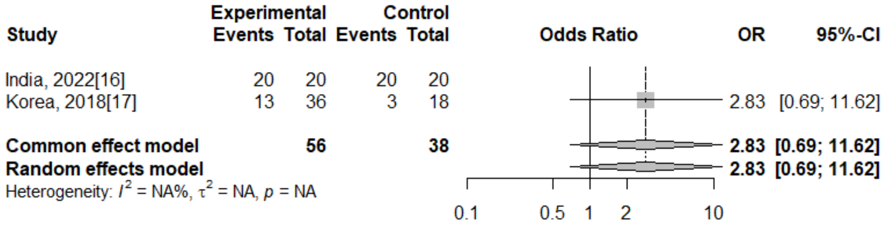

Figure 33 Meta-analysis of the incidence of infections of RTX treatment in SSNS compared with the conventional immunosuppressant control group

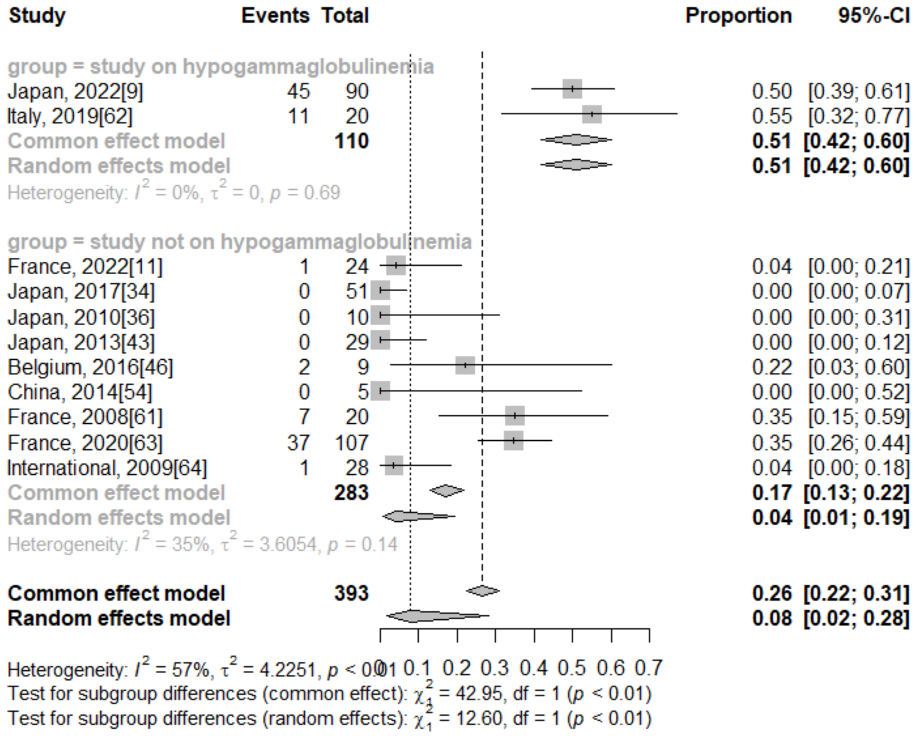

Figure 34 Meta-analysis of the incidence of hypogammaglobulinemia of RTX treatment in SSNS

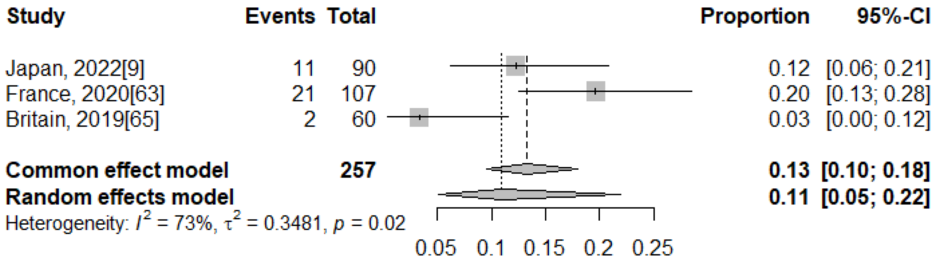

Figure 35 Meta-analysis of the incidence of persistent hypogammaglobulinemia of RTX treatment in SSNS

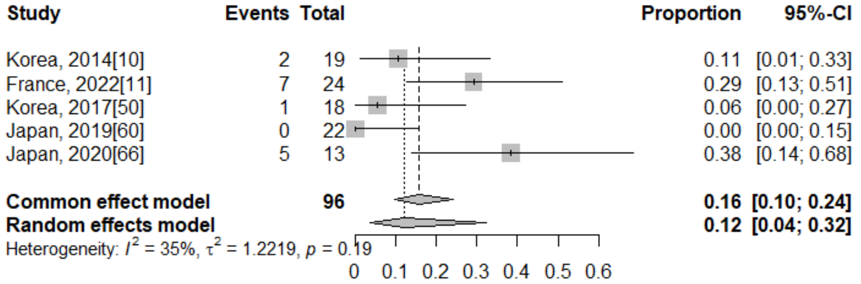

Figure 36 Meta-analysis of anti-RTX antibody (ARA) detection rate of RTX treatment in SSNS

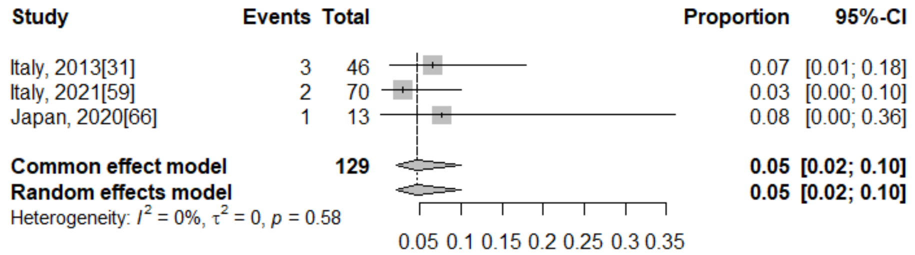

Figure 37 Meta-analysis of the incidence of serum sickness of RTX treatment in SSNS

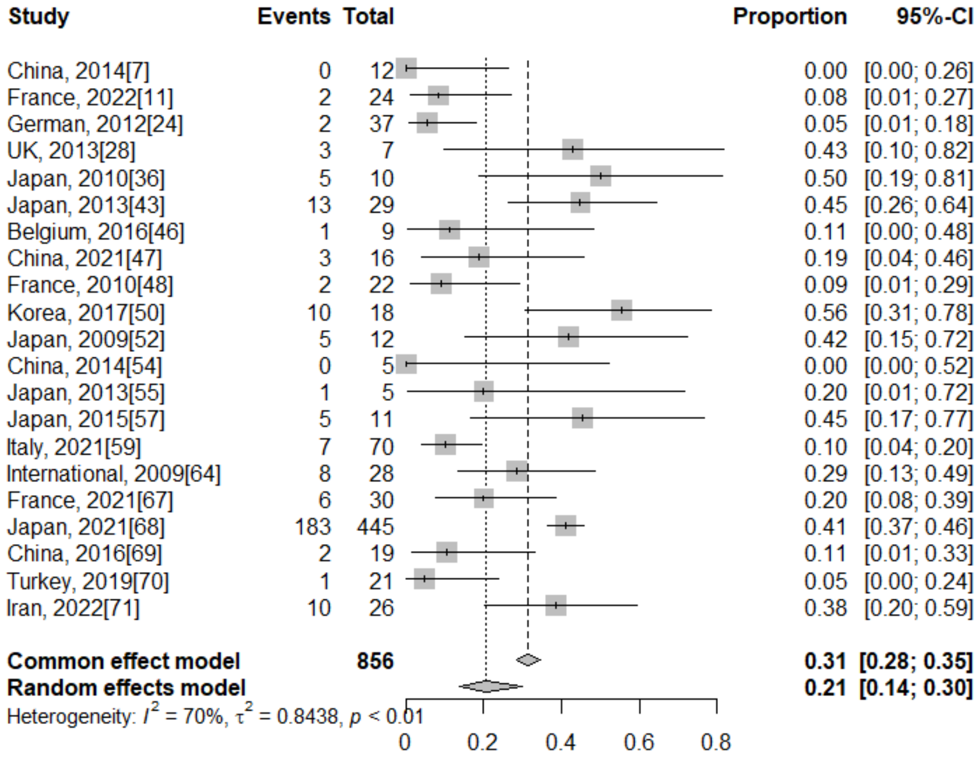

Figure 38 Meta-analysis of the incidence of infusion-related reactions of RTX treatment in SSNS

Planning Proposal of Clinical Practice Guidelines for the Rituximab Treatment in Children with Steroid-sensitive Nephrotic Syndrome

Developed by the Fudan University GRADE Center and reviewed by the Core Expert Group

1 Background

Primary nephrotic syndrome (PNS) is the most common glomerular disease in children, and 80% to 90% of pediatric patients present with steroid-sensitive nephrotic syndrome (SSNS) following glucocorticoid regimens that are currently recognized by international communities. Rituximab (RTX) is a human-mouse chimeric anti-CD20 monoclonal antibody[1]. In recent years, RTX has been gradually applied to the treatment of SSNS children with favorable efficacy. However, there are still several clinical issues such as inconsistent indications and diverse treatment and follow-up management options [2]. Moreover, there are no consensus or guidelines on RTX treatment of SSNS children in China and abroad. On the one hand, clinical practice calls for guidelines on indications, treatments, and follow-up management for the application of RTX in SSNS children; on the other hand, there are predictable challenges to the development of the guidelines, such as insufficient and low-quality literature to answer clinical questions and large gaps between the available literature and the topics of the guidelines.

2 Initiating Institutions

Jointly initiated by the “Pediatric Nephrology Group of Pediatrician Branch of Chinese Medical Doctor Association”, the "Nephrology Group of Pediatrics Branch of Chinese Medical Association" and "Fudan University GRADE Center".

3 Purpose

To develop standard guidelines to answer the main clinical questions regarding the indications, treatments, and follow-up management for the application of RTX in SSNS children.

4 Target Healthcare Professionals

Pediatric nephrologists and nurses.

5 Target Patient Populations

Clinically confirmed SSNS children aged 1 to 18 years.

6 Guideline Development Teams and the Responsibilities

6.1 Core Expert Group Clinical Experts: Professor Xu Hong (Head of Nephrology Group of Pediatrician Branch of Chinese Medical Doctor Association), Professor Zhang Aihua (Head of Nephrology Group of Pediatrics Branch of Chinese Medical Association), Professor Xia Zhengkun (General Hospital of Eastern Theater Command), and Professor Shen Qian (Department of Nephrology, Children's Hospital of Fudan University); Methodologist: Zhang Chongfan (Fudan University GRADE Center).

Responsibilities of Core Expert Group: ① Supervise, coordinate, and review the academic and methodological quality of the guidelines; ② Preside over and organize the working meetings for the guidelines; ③ Develop the work strategies for the guidelines to control the schedule; ④ Organize the discussion on the structure, purpose, target healthcare professionals, target patient populations, and planning proposal of the guidelines; ⑤ Coordinate the construction of guidelines-related clinical questions; ⑥ Review and confirm the screening, assessment, extraction, and synthesis of evidences; ⑦ Coordinate the discussion on the evidence-to-recommendations process; ⑧ Review the draft and final versions of the guidelines; ⑨ Review and manage the conflicts of interest and sign the conflict of interest form; ⑩ Supervise and coordinate the publication, distribution, and updating of the guidelines.

6.2 Methodology Team Zhang Chongfan, Wang Yingwen, and Wang Huishan (Fudan University GRADE Center).

6.3 Secretariat Academic Secretaries: Liu Jialu, Shao Leilin (Department of Nephrology, Children's Hospital of Fudan University), Wang Yingwen (Fudan University GRADE Center), and Chen Qiuxia (Children's Hospital of Nanjing Medical University); Administrative Secretary: Wang Huishan (Fudan University GRADE Center).

Responsibilities of Academic Secretaries: ① Collect guidelines-related clinical questions and construct the PICO based on significant and limited guidance questions; ② Conduct retrieval, screening, and bias risk assessment of literature, and create the summary of findings table and GRADE evidence profile; ③ Prepare systematic assessment and meta-analysis reports; ④ Draft recommendations; ⑤ Write the draft guidelines.

Responsibilities of Administrative Secretary: ① Complete the guidelines registration and draft the guidelines planning proposal; ② Collect the Interest Declaration; ③ Develop the schedule as per the progression of the guidelines, prepare working meetings for the guidelines, record and maintain meeting information, and draft meeting minutes; ④ Develop the questionnaires as per the guideline, collect and sort out the questionnaires; ⑤ Assist in the publication and dissemination of the guidelines.

6.4 Panel of Experts (Members recommended and invited by the Core Expert Group in no particular order): Xu Hong, Shen Qian (Children's Hospital of Fudan University), Zhang Aihua (Children's Hospital of Nanjing Medical University), Mao Jianhua (Children’s Hospital of Zhejiang University School of Medicine), Jiang Xiaoyun (The First Affiliated Hospital of Sun Yat-sen University), Liu Cuihua (Henan Children's Hospital), Wang Xiaowen (Wuhan Children's Hospital), Zhang Ruifeng (Xuzhou Children's Hospital), Xia Zhengkun (General Hospital of Eastern Theater Command), Wu Yubin (Shengjing Hospital of China Medical University), Bao Ying (Xi'an Children's Hospital), Shen Tong (Xiamen Maternal and Child Health Care Hospital), Xu Zhiquan (Hainan Women and Children's Medical Center), Yang Qing (The Second Affiliated Hospital of Wenzhou Medical University), Zhang Jianjiang (The First Affiliated Hospital of Zhengzhou University), Zhang Dongfeng (Hebei Children's Hospital), Feng Shipin (Chengdu Women and Children's Central Hospital), Yu Zihua (Fujian Children's Hospital), Yu Li (Guangzhou First People's Hospital), Xiao Huijie (Peking University First Hospital), Li Zhihui (Hunan Children's Hospital), Wang Mo (Children's Hospital of Chongqing Medical University), Guo Yanyan (The Affiliated Hospital of Inner Mongolia Medical University), Fu Rui (Jiangxi Children's Hospital), Sun Qing (Qingdao Women and Children's Hospital), Liu Yuling (Boai Hospital of Zhongshan), Gao Xia (Guangzhou Women and Children's Medical Center), Zhao Bo (Kunming Children's Hospital Affiliated to Kunming Medical University), Bai Haitao (The First Affiliated Hospital of Xiamen University), Shao Xiaoshan (Guiyang Maternal and Child Health Care Hospital/Guiyang Children's Hospital), Yu Lei (Urumqi Children's Hospital), Huang Chunlin (Maternity and Child Health Care of Guangxi Zhuang Autonomous Region), Zhang Bili (Tianjin Children's Hospital), Zhao Lijun (Children's Hospital of Shanxi), Liang Lijun (General Hospital of Ningxia Medical University), Xing Xuehua (Qinghai Province Women and Children's Hospital), Cai Shaojiang (Harbin Children's Hospital), and Ren Hong (Ruijin Hospital, Shanghai Jiao Tong University School of Medicine)

Responsibilities of Panel of Experts: ① Participate in the discussion of topic selection and voting on the priority of clinical questions; ② Discuss and provide suggestions for recommendations given in the guidelines based on evidences from the systematic review; ③ Participate in the discussion and voting on the expert consensus in the guidelines.

7 Guideline Registration

The document of the Guidelines is registered in both Chinese and English on the platform of the Practice Guideline REgistration for transPAREncy (PREPARE), with a registration number as IPGRP-2022CN361.

8 Declaration and Management of Conflicts of Interest

Both experts and secretaries invited to participate in the development of the guidelines should sign the conflict of interest form, and they should make a declaration and update the conflict of interest form in case any conflict of interest is identified.

The Steering Group will review any potential conflicts of interest involved in the development of the guidelines. The Steering Group will persuade stakeholders to withdraw in case of a major conflict of interest that potentially poses a higher risk of bias or compromise the credibility of the recommendations in the Guidelines. The declaration of conflict of interest, as well as any conflicts of interest involved in the development of the Guidelines and its management, will be described in the published Guidelines.

9 Methodology for Developing Guideline

The following tools will be followed and referenced: WHO Handbook for Guideline Development (2014 Edition) [3] and The Grading of Recommendations Assessment, Development and Evaluation (GRADE) [4].

10 Construction of Guideline Topics and Identification of Significant Outcomes

10.1 Construction of Guideline Topics

10.1.2 Preliminary Construction of Guideline Topics Preliminary survey on guideline topics will be conducted from May 25, 2022 to June 8, 2022. According to the preliminary survey results, the topics of the guidelines will be summarized and integrated from June 10, 2022 to June 17, 2022 to construct PICO, respectively.

Construction of overall PICOS:

P1: SSNS children aged 1 to 18 years

P2: SSNS children aged 1 to 18 years and treated with RTX

P3: SSNS children aged 1 to 18 years with disease relapse after RTX treatment

I1: RTX

I2: Glucocorticoids or immunosuppressants [mycophenolate mofetil (MMF) or calcineurin inhibitor (CNI)]

I3: Consolidation therapy (re-use of RTX or MMF or CNI)

C1: MMF

C2: CNI

C3: Blank control or placebo

O1: Relapse-free survival (follow-up > 3 months)

O2: Glucocorticoid dosage (follow-up >3 months)

O3: Time to glucocorticoid withdrawal

O4: Quality of life (follow-up >1 year)

O5: Depletion of CD20+/CD19+ B cells

O6: Increase in urine protein or repletion of CD20+/CD19+ B cells (follow-up >3 months)

S1: RCT

S2: NRSI

S3: Case series report

S4: Cost analysis method, cost-effectiveness analysis, cost-utility analysis, and cost-benefit analysis

10.1.2 Prioritization of Clinical Questions A questionnairing on the priority of 9 clinical questions (Table 1) will be conducted in the Panel of Experts, with the assessment of the priority of each clinical question based on the International Guideline Development Credentialing & Certification Program (INGUIDE). The assessors will judge the consistency of each question with their clinical practice by scoring 0 for Uncertain, 1 for Yes, and -1 for No. The clinical questions with higher scores will be given a higher priority.

Table 1 Assessment of Priority of Clinical Questions

Question	Score
①Is this question common in clinical practice?	Uncertain; Yes; No
②Is there any uncertainty about this question in clinical practice?	Uncertain; Yes; No
③Has this question not been answered before?	Uncertain; Yes; No
④This question has been asked, but there was little or poor-quality evidence at the time. Is there any new evidence to answer it now?	Uncertain; Yes; No
⑤Does this question vary widely in clinical practice (possible differences include but are not limited to different clinical settings, no recommendations, and insufficiently clear recommendations)?	Uncertain; Yes; No
⑥Does this question pose a significant impact on resource accessibility, resource utilization, and cost?	Uncertain; Yes; No

11 Literature Retrieval

11.1 Database English database: Pubmed, Embase, Cochrane Library, and Scopus; Chinese database: SinoMED.

11.2 Period January 1, 2004 to June 22, 2022.

11.3 Languages Chinese and English.

11.4 Keywords and Search Query Preliminary search prompts that there is a great risk of incomplete retrieval of literature when constructing the search query with topic PICO, while a maximum of 3,000 documents can be retrieved from 5 databases in Section 11.1 when constructing the search query with P (P1-3) and I (I1-3), ensuring sufficient retrieval of literature, and the workload of literature screening is acceptable.

11.5 Supplementary Literature Retrieval of relevant references in the systematic review/meta-analysis and duplicate checking of retrieved literature will be performed as a supplement.

12 Literature Screening

12.1 Preliminary Screening of Titles and Abstracts: The Core Expert Group will establish the inclusion and exclusion criteria for the preliminary screening of titles and abstracts by reviewing not less than 5% of the retrieved literature relevant to the topics. The formal preliminary screening will be independently performed by 2 reviewers to classify literature as "included", "excluded", or "pending". The pending literature will be discussed and decided by the Core Expert Group in a meeting.

12.2 Full-text Screening The preliminarily screened literature will proceed to the full-text screening independently performed by 2 reviewers to classify literature as "included", "excluded", or "pending". If two reviewers reach a unanimous decision, the literature will be handled as "included" or "excluded"; in case of conflicting decisions by two reviewers, the third reviewer will determine the fate of the literature; for the pending literature, it will be subject to judgment by the third reviewer for inclusion or exclusion. If the third reviewer assigned by the Core Expert Group fails to give a conclusive decision, the literature will be discussed and judged with the Steering Group.

13 Evidence Extraction

At least 5% of the included literature will be selected for preliminary extraction by the literature

extraction reviewers to create an extracted data table. Two evidence extraction reviewers will extract the literature. In case of any disagreement, the reviewer who is a member of the Core Expert Group will make a decision, otherwise, it will be submitted to the Core Expert Group for discussion and judgment.

14 Evidence Synthesis

The Methodology Team will train the Academic Secretaries on the systematic review/meta-analysis and conduct the systematic review/meta-analysis as necessary.

15 Quality Assessment of Body of Evidence

The Methodology Team will train the Academic Secretaries on the assessment of a body of evidence. Types of literature possibly involved in this guideline: In order to assess the risk of bias, RoB 2[5] will be utilized for RCTs, ROBINS-I[6] will be utilized for non-RCTs, and Quality of Health Economic Studies (QHES) [7] will be utilized for health economics studies.

16 Generation of GRADE Evidence Profile

The GRADE evidence profile will be prepared by the Academic Secretaries under the organization of the Methodology Team and will be validated by the Core Expert Group.

17 Evidence Grading and Evidence-to-Recommendations Process

The quality of evidence and strength of recommendations will be assessed as per the GRADE system. Using the GRADE Evidence-to-Decision (EtD) frameworks [8] and considering various aspects such as the priority of clinical questions, beneficial effects of evidence, adverse effects of evidence, quality of evidence, values (views of parents of pediatric patients), tradeoffs between benefits and harms, costs and the quality of evidence thereof, cost-effectiveness, fairness, acceptability, and feasibility, the Core Expert Group will implement multiple Delphi rounds to reach a consensus on the recommendations and clarify the strength of recommendations. In the process, there should be sufficient opportunity and time available for minority opinions to be expressed. If ≥ 90% of experts approve in each Delphi round, it is considered to reach a consensus. When the available evidence is deemed insufficient to answer clinical questions, recommendations can be

generated through indirect evidence or consensus of the Expert Group.

17 Funding

To be determined.

18 Guideline Writing

The Methodology Team will coordinate with the Academic Secretaries to develop the draft guideline with reference to the RIGHT items [9]. The draft will then be submitted for discussion and confirmation by the Core Expert Group.

19 External Review

The external review of the guidelines will involve the participants who are interested in it but not directly engaged in its development, such as clinical experts, public health economists, guideline development methodologists, and parents of pediatric patients. The external reviewers will assess the guidelines from various professional perspectives and put forward opinions. If there are significant doubts about one or more recommendations, the Core Expert Group should meet to discuss and resolve such questions.

20 Publication and Updating of Guideline

The final guideline is expected to be published in the *Chinese Journal of Evidence-Based Pediatrics*. Considering the update of evidence, it is planned to revise the guidelines in 3 years.

21 Dissemination, Implementation, and Assessment of Guideline

After the official publication of the guidelines, ① it will be interpreted at relevant academic conferences; ② a post-implementation effect study will be conducted as per the primary outcomes in the guidelines.

22 Schedule of Guideline Development

Table 2 Schedule of Guideline Development

Topics	Description	Time (weeks)	Responsible Person
**Planning of Guideline**		1	
Preliminary	Preliminary survey of guideline topics and initial development		Core Expert Group +

construction of guideline topics	of a list of clinical questions		Panel of Experts
Target healthcare professionals	Pediatric nephrologists and nurses		Core Expert Group + Panel of Experts
Target patient populations	Clinically confirmed SSNS children aged 1 to 18 years		Core Expert Group + Panel of Experts
Methodology	WHO Handbook for Guideline Development (2014 Edition) and RIGHT (Reporting Items for practice Guidelines in Healthcare) Checklist (Version 2.0)		Core Expert Group + Panel of Experts
**Establishment of Guideline Development Group**	Establishment of Core Expert Group (Secretariat), Panel of Experts and External Reviewer Group and definition of their responsibilities	1	Core Expert Group
**Prioritization of Clinical Questions and Retrieval of Evidence**	List of clinical questions - Important questions PICO construction, database retrieval, keywords, and search query	1	Core Expert Group + Panel of Experts + Secretariat
**Literature Screening**	Preliminary screening by title and abstract review and screening by full-text review	3	Core Expert Group + Secretariat
**Bias Risk Assessment of Literature**	Systematic review: AMSTAR 2; RCT: ROB 2; non-RCT, cohort study, case-control study: ROBINS-I; cross-sectional study: AHRQ	2	Core Expert Group + Methodology Team + Secretariat
**Generation of GRADE Evidence Profile**	Pooling of evidence or a body of evidence	1	Core Expert Group + Methodology Team + Secretariat
**Evidence-to-Recommendations**	GRADE EtD frameworks and Delphi rounds	2	Core Expert Group + Methodology Team + Secretariat
**Guideline Writing**	Guidelines drafted by the Secretariat and refined by the Panel of Experts	2	Core Expert Group + Methodology Team + Secretariat
**External Review**	Peer review by correspondence	1	External Reviewer Group
**Finalization of Guideline**	Finalization of the guidelines after refining recommendations as per external review feedback	1	Core Expert Group + Panel of Experts + Secretariat
**Total**		15	

23 References

[1]北京大学医学部肾脏病学系专家组. 利妥昔单抗在膜性肾病中应用的专家共识[J]. 中华内科杂志,2022,61(3):282-290.

[2]中华医学会肾脏病学分会专家组. 利妥昔单抗在肾小球肾炎中应用的专家共识[J]. 中华肾脏病杂志,2022,38(2):151-160.

[3]World Health Organization. WHO handbook for guideline development: WHO, 2014.

[4]Guyatt GH, Oxman AD, Schu¨nemann HJ, et al. GRADE guidelines: A new series of articles in the Journal of

Clinical Epidemiology. J Clin Epidemiol, 2011, 64(4): 382-382.

[5]Sterne J A C, Savovic J, Page M J, et al. RoB2: a revised tool for assessing risk of bias in randomised trials. BMJ, 2019, 366: l4898.

[6]Sterne J A, Hernan M A, Reeves B C, et al. ROBINS-I: a tool for assessing risk of bias in non-randomised studies of interventions. BMJ, 2016, 355: i4919.

[7] OFMAN J J, SULLIVAN S D, NEUMANN P J, et al. Examining the Value and Quality of Health Economic Analyses: Implications of Utilizing the QHES[J]. Journal of Managed Care Pharmacy, 2003, 9(1): 53–61.

[8] Alonso-Coello P, Oxman AD, Moberg J, et al. GRADE Evidence to Decision (EtD) frameworks: a systematic and transparent approach to making well informed healthcare choices. 2: Clinical practice guidelines. BMJ, 2016, 353:i2089.

[9]Chen Y, Yang K, Marušic A, et al. A reporting tool for practice guidelines in health care: the RIGHT statement. Ann Intern Med, 2017, 166(2): 128-132.

2022-6-22

Selection of Clinical Topics of the Rituximab Guidelines

List of Clinical Topics

1. Does RTX improve clinical outcomes in children aged 1-18 years with SSNS compared with other immunosuppressants or blank/placebo control?
2. What are the effects on clinical outcomes of different steroid or immunosuppressant discontinuation regimens in children aged 1-18 years with SSNS after RTX treatment?
3. Is it feasible to take peripheral CD19+/CD20+ B cell count as a monitoring indicator in children aged 1-18 years with SSNS after RTX treatment?
4. Is it feasible to take peripheral CD19+/CD20+ B cell count as a relapse predictor in children aged 1-18 years with SSNS after RTX treatment?
5. What are the effects on clinical outcomes of consolidation therapy in children aged 1-18 years with SSNS after RTX treatment?
6. What are the effects on clinical outcomes of repeated RTX treatment in children aged 1-18 years with SSNS relapsing after RTX treatment?
7. What is the cost-effectiveness of RTX treatment in children aged 1-18 years with SSNS?
8. What is the incidence of adverse events (including allergies, infections, hypoleukocytosis and hypogammaglobulinemia, etc) in RTX treatment (first use and second use) of children with SSNS?
9. What measures should be taken to prevent RTX-induced adverse reactions (first use and second use)?

Construction of Overall PICOS

P1: SSNS children aged 1 to 18 years

P2: SSNS children aged 1 to 18 years and treated with RTX

P3: SSNS children aged 1 to 18 years with disease relapse after RTX treatment

I1: RTX

I2: Glucocorticoids or immunosuppressants [mycophenolate mofetil (MMF) or calcineurin inhibitor (CNI)]

I3: Consolidation therapy (re-use of RTX or MMF or CNI)

C1: MMF

C2: CNI

C3: Blank control or placebo

O1: Relapse-free survival (follow-up > 3 months)

O2: Glucocorticoid dosage (follow-up >3 months)

O3: Time to glucocorticoid withdrawal

O4: Quality of life (follow-up >1 year)

O5: Depletion of CD20+/CD19+ B cells

O6: Increase in urine protein or repletion of CD20+/CD19+ B cells (follow-up >3 months)

S1: RCT

S2: NRSI

S3: Case series report

S4: Cost analysis method, cost-effectiveness analysis, cost-utility analysis, and cost-benefit analysis

English Search Details

Cochrane: 2022/06/24 n=141 (trials-129, Cochrane Reviews-12)

1#: MeSH descriptor: [Nephrotic Syndrome] This term only

2#: MeSH descriptor: [Nephrosis, Lipoid] This term only

3#: (("Nephrotic" and "Syndrome*") or ("Lipoid" AND "Nephrose*") or (("Minimal Change") and ("Disease*" or "Nephropath*" or "Glomerulopath*" or "Nephrotic Syndrome" or "Glomerulonephriti*")) or ("Kidney" and "Disease*"))

4#: ("steroid" or "Primary" or "corticosteroid" or "sensitive" or "dependent" or "frequently relapsing" or "refractory"):ti,ab,kw

5#: (#1 or #2 or #3) and #4

6#:MeSH descriptor: [Rituximab] Explode all trees

7#: (("CD20 Antibody" and "Rituximab") or "Mabthera" or ("IDEC-C2B8" and "Antibody") or "Rituxan" or "GP2013"):ti,ab,kw

8#: MeSH descriptor: [Steroids] This term only

9#: (("Catatoxic" and "Steroids") or "glucocorticoids" or "prednisone" or "prednisolone"):ti,ab,kw

10#: MeSH descriptor: [Immunosuppression Therapy] Explode all trees

11#: MeSH descriptor: [Immunosuppressive Agents] Explode all trees

12#: (("Immunosuppressant*" or "Immunosuppression" or "Anti-Rejection" or "Immunosuppressive" or "Antirejection" or "maintenance" or "Combination") and ("Therapy*" or "Agents")) or ("Repeated" and ("doses" or "courses" or "administration")) or ("concomitant" and ("agents" or "drugs"))

13#: #5 and (#6 or #7 or #8 or #9 or #10 or #11 or #12)

14#: MeSH descriptor: [child] This term only

15#：#13 and #14

#1	MeSH descriptor: [Nephrotic Syndrome] this term only	MeSH	383
#2	MeSH descriptor: [Nephrosis, Lipoid] this term only	MeSH	51
#3	(("Nephrotic" and "Syndrome*") or ("Lipoid" AND "Nephrose*") or (("Minimal Change") and ("Disease*" or "Nephropath*" or "Glomerulopath*" or "Nephrotic Syndrome" or "Glomerulonephriti*")) or ("Kidney" and "Disease*")):ti,ab,kw	Limits	28362
#4	("steroid" or "Primary" or "corticosteroid" or "sensitive" or "dependent" or "frequently relapsing" or "refractory"):ti,ab,kw	Limits	516867
	with Cochrane Library publication date from Jan 2004 to Jun 2022		
#5	(#1 or #2 or #3) and #4	Limits	13746
#6	MeSH descriptor: [Rituximab] explode all trees	MeSH	1446
#7	(("CD20 Antibody" and "Rituximab") or "Mabthera" or ("IDEC-C2B8" and "Antibody") or "Rituxan" or "GP2013"):ti,ab,kw	Limits	643
#8	MeSH descriptor: [Steroids] this term only	MeSH	796
#9	(("Catatoxic" and "Steroids") or "glucocorticoids" or "prednisone" or "prednisolone"):ti,ab,kw	Limits	22098
#10	MeSH descriptor: [Immunosuppression Therapy] explode all trees	MeSH	2163
#11	MeSH descriptor: [Immunosuppressive Agents] this term only	MeSH	5311
#12	((("Immunosuppressant*" or "Immunosuppression" or "Anti-Rejection" or "Immunosuppressive" or "Antirejection" or "maintenance" or "Combination") and ("Therapy*" or "Agents")) or ("Repeated" and ("doses" or "courses" or "administration")) or ("concomitant" and ("agents" or "drugs"))):ti,ab,kw	Limits	174825
#13	#5 and (#6 or #7 or #8 or #9 or #10 or #11 or #12)	Limits	3294
	with Cochrane Library publication date from Jan 2004 to Jun 2022, in Cochrane Reviews, Cochrane Protocols, Trials, Clinical Answers, Editorials and Special Collections		
#14	MeSH descriptor: [Child] explode all trees	MeSH	61335
#15	#13 and #14	Limits	141
	with Cochrane Library publication date from Jan 2004 to Jun 2022		

Embase: 2022/06/26 n=1729

('frequently relapsing nephrotic syndrome'/exp OR 'steroid sensitive nephrotic syndrome'/exp OR 'steroid dependent nephrotic syndrome'/exp OR 'refractory nephrotic syndrome'/exp OR 'minimal change disease'/exp OR 'corticosteroid dependent nephrotic syndrome' OR 'primary nephritic syndrome' OR 'nephrotic syndrome'/exp OR 'idiopathic nephrotic syndrome') AND ('child'/de OR 'infant'/de) AND ('rituximab'/exp OR 'abp 798' OR 'abp798' OR 'blitzima' OR 'ct p10' OR 'ctp10' OR 'gp 2013' OR 'gp2013' OR 'halpryza' OR 'hlx 01' OR 'hlx01' OR 'ibi 301' OR 'ibi301' OR 'idec 102' OR 'idec c2b8' OR 'idec102' OR 'idecc2b8' OR 'mabthera' OR 'mk 8808' OR 'mk8808' OR 'monoclonal antibody idec c2b8' OR 'pf 05280586' OR 'pf 5280586' OR 'pf05280586' OR 'pf5280586' OR 'r 105' OR 'r105' OR 'reditux' OR 'rg 105' OR 'rg105' OR 'riabni' OR 'ritemvia' OR 'ritucad' OR 'ritumax' OR 'rituxan' OR 'rituximab' OR 'rituximab abbs' OR 'rituximab arrx' OR 'rituximab pvvr' OR 'rituximab-abbs' OR 'rituximab-arrx' OR 'rituximab-pvvr' OR 'rituxin' OR 'rituzena' OR 'rixathon' OR 'riximyo' OR 'ro 452294' OR 'ro452294' OR 'rtxm 83' OR 'rtxm83' OR 'ruxience' OR 'truxima' OR 'tuxella' OR 'immunosuppressive treatment'/mj OR 'immune depressive therapy' OR 'immune suppression' OR 'immuno suppression' OR 'immuno suppressive treatment' OR 'immunosuppression' OR 'immunosuppression therapy' OR 'immunosuppression, active' OR 'immunosuppressive therapy' OR 'immunosuppressive treatment' OR 'transplantation reaction inhibition' OR 'immunosuppressive agent'/mj OR 'drug, immunosuppressive' OR 'immune suppressant' OR 'immuno suppressive drug' OR

'immunodepressant' OR 'immunodepressant agent' OR 'immunosuppressant' OR 'immunosuppressant agent' OR 'immunosuppressant drug' OR 'immunosuppressive agent' OR 'immunosuppressive agents' OR 'immunosuppressive drug' OR 'immunosuppressive substance' OR 'immunosuppressives' OR 'immunosuppressor' OR 'maintenance immunosuppression' OR 'maintenance therapy'/de OR 'drug maintenance' OR 'maintenance therapy' OR 'maintenance treatment' OR 'therapy, maintenance' OR 'steroid'/de OR 'cyclosteroids' OR 'steroid' OR 'steroid compound' OR 'steroid derivative' OR 'steroidal compound' OR 'steroids' OR 'steroids, brominated' OR 'steroids, chlorinated') AND [2004-2022]/py AND [embase]/lim

Pubmed: 2022/06/26 n=822

((("Nephrotic Syndrome"[Mesh] OR "Nephrosis, Lipoid"[Mesh] OR "Kidney Diseases"[Mesh]) OR ("Nephrotic"[All Fields] AND "Syndrome*"[All Fields]) OR ("Lipoid"[All Fields] AND "Nephrose*"[All Fields]) OR (("Minimal Change"[All Fields] AND ("Disease*"[All Fields] OR "Nephropath*"[All Fields] OR "Glomerulopath*"[All Fields] OR "Nephrotic Syndrome"[All Fields] OR "Glomerulonephriti*"[All Fields])) OR ("Kidney"[All Fields] AND "Disease*"[All Fields])) AND ((("steroid"[All Fields] OR "Primary"[All Fields] OR "corticosteroid"[All Fields]) AND ("sensitive"[All Fields] OR "dependent"[All Fields])) OR "frequently relapsing"[All Fields] OR "refractory"[All Fields]))) AND ((("Rituximab"[Mesh] OR ("CD20 Antibody"[All Fields] AND "Rituximab"[All Fields]) OR "Mabthera"[All Fields] OR ("IDEC-C2B8"[All Fields] AND "Antibody"[All Fields]) OR "Rituxan"[All Fields] OR "GP2013"[All Fields])) OR (("Steroids"[Mesh] OR ("Catatoxic"[All Fields] AND "Steroids"[All Fields]) OR "glucocorticoids"[All Fields] OR "prednisone"[All Fields] OR "prednisolone"[All Fields])) OR ((("Immunosuppression Therapy"[Mesh] OR "Immunosuppressive Agents"[Mesh] OR "Immunosuppressant*"[All Fields] OR ("Immunosuppression"[All Fields] OR ("Anti-Rejection"[All Fields] OR "Immunosuppressive"[All Fields] OR "Antirejection"[All Fields] OR "maintenance"[All Fields]) AND ("Therapy*"[All Fields] OR "Agents"[All Fields]))) OR ("Repeated"[All Fields] AND ("doses"[All Fields] OR "courses"[All Fields] OR "administration"[All Fields]))OR ("concomitant"[All Fields] AND ("agents"[All Fields] OR "drugs"[All Fields]))))) Filters applied: Child: birth-18 years.2004 年至今

Scopus: 2022/06/26 n=1163

TITLE-ABS-KEY(("Nephrotic Syndrome" OR "Nephrosis, Lipoid" OR "Kidney Diseases" OR ("Minimal Change" AND ("Disease*" OR "Nephropath*" OR "Glomerulopath*" OR "Nephrotic Syndrome" OR "Glomerulonephriti*"))) AND ((("corticosteroid " OR "steroid") AND ("sensitive" OR "dependent")) OR "Idiopathic" OR "Primary" OR "frequently relapsing" OR "refractory") AND ("Rituximab" OR ("CD20 Antibody" AND "Rituximab") OR "Mabthera" OR ("IDEC-C2B8" AND "Antibody") OR "Rituxan" OR "GP2013" OR "Steroids" OR "Immunosuppression Therapy" OR "Immunosuppressive Agents" OR "Immunosuppressant*" OR "Immunosuppression" OR (("Anti-Rejection" OR "Immunosuppressive" OR "Antirejection" OR "maintenance") AND ("Therapy*" OR "Agents")) OR ("Repeated" AND ("doses" OR "courses" OR "administration")) OR ("concomitant" AND ("agents" OR "drugs"))) AND ("child*" or "infant*" or "preschool*")) AND PUBYEAR AFT 2004 AND (LIMIT-TO (DOCTYPE , "ar"))

Chinese Search Details

SinoMed：2022/06/24　SinoMed=487

(((("原发"[常用字段] OR "激素敏感"[常用字段] OR "激素依赖"[常用字段] OR "频发复杂"[常用字段] OR "频复发"[常用字段] OR "难治"[常用字段]) AND ("肾病综合征"[常用字段] OR "肾病综合征"[主题词] OR "微小病变肾病"[常用字段] OR "肾病, 脂性"[主题词])) AND ("利妥昔"[常用字段] OR "美罗华"[常用字段] OR "利妥昔单抗"[常用字段] OR "利妥昔单抗 CD20 抗体"[常用字段] OR "GP2013"[常用字段] OR "IDEC-C2B8"[常用字段] OR "Rituximab"[常用字段] OR "Rituxan"[常用字段] OR "利妥昔单抗"[主题词] OR "汉利康"[常用字段] OR "巩固治疗"[常用字段] OR "免疫抑制"[常用字段] OR "重复给药"[常用字段] OR "剂量"[常用字段] OR "合并用药"[常用字段] OR "糖皮质激素"[常用字段] OR "疗程"[常用字段]))) AND ("婴儿, 新生"[特征词] OR 婴儿[特征词] OR "儿童, 学龄前"[特征词] OR 儿童[特征词] OR 青少年[特征词]) AND 人类[特征词] AND 2004-2022[日期]

Version 2 of the Cochrane risk-of-bias assessment tool for randomised trials (RoB 2.0)

Bias domain	Signalling questions*	No.17	No.14	No.12	No.16	No.13	No.41	No.59	No.15	No.37	No.18
**1 Bias arising from the the randomization process**	1.1 Was the allocation sequence random?	Y	Y	Y	Y	Y	Y	Y	Y	Y	Y
	1.2 Was the allocation sequence concealed until participants were enrolled and assigned to interventions?	NI	Y	NI	Y	Y	Y	NI	Y	Y	Y
	1.3 Did baseline differences between intervention groups suggest a problem with the randomisation process?	N	N	N	N	N	PN	N	PN	PN	PN
	**Risk-of-bias judgment**	some concerns	Low	some concerns	Low	Low	Low	some concerns	Low	Low	Low
**2 Bias due to deviations from the intended interventions (effect of adhering to intervention)**	2.1 Were participants aware of their assigned intervention during the trial?		Y		Y		N		Y	Y	Y
	2.2 Were carers and people delivering the interventions aware of participants' assigned intervention during the trial?		Y		Y		N		Y	Y	Y
	2.3 If Y/PY/NI to 2.1 or 2.2: Were important nonprotocol interventions balanced across intervention groups?		Y		Y		NA		PY	PY	PY
	2.4 If Y/PY to 2.3: Were these deviations likely to have affected the outcome?		N		N		N		PN	PN	PN
	2.5 Was there non-adherence to the assigned intervention regimen that could have affected participants’		Y		Y		Y		Y	Y	Y

	outcomes?										
	2.6 If N/PN/NI to 2.3, or Y/PY/NI to 2.4 or 2.5 Was an appropriate analysis used to estimate the effect of adhering to intervention?		NA		NA		NA		NA	NA	NA
	**Risk-of-bias judgment**		Low		Low		Low		some concerns	some concerns	some concerns
**3 Bias due to deviations from the intended interventions (effect of assignment to intervention)**	3.1 Were participants aware of their assigned intervention during the trial?	Y	Y	N	Y	Y	N	Y	Y	Y	Y
	3.2 Were carers and people delivering the interventions aware of participants' assigned intervention during the trial?	Y	Y	N	Y	Y	N	Y	Y	Y	Y
	3.3 If Y/PY/NI to 3.1 or 3.2: Were there deviations from the intended intervention that arose because of the trial context?	NI	PN	PN	PN	NI	NA	NI	NI	NI	NI
	3.4 If Y/PY to 3.3: Were these deviations likely to have affected the outcome?	/	NA	/	NA	/	NA	/	NA	NA	NA
	3.5 Were these deviations from intended intervention balanced between groups?	/	NA	/	NA	/	NA	/	NA	NA	NA
	3.6 Was an appropriate analysis used to estimate the effect of assignment to intervention?	Y	Y	Y	Y	Y	PY	PN	PY	PY	PY
	3.7 If N/PN/NI to 3.6: Was there potential for a substantial impact (on the result) of the failure to analyse participants in the	/	NA	/	NA	/	NA	PN	NA	NA	NA

	group to which they were randomized?										
	**Risk-of-bias judgment**	some concerns	Low	Low	Low	some concerns	Low	some concerns	some concerns	some concerns	some concerns
**4 Bias due to missing outcome data**	4.1 Were data for this outcome available for all, or nearly all, participants randomized?	N	Y	Y	Y	Y	Y	Y	Y	PN	PN
	4.2 If N/PN/NI to 4.1: Is there evidence that the result was not biased by missing outcome data?	N	NA	/	NA	/	NA	/	NA	PN	PN
	4.3 If N/PN to 4.2: Could missingness in the outcome depend on its true value?	NI	NA	/	NA	/	NA	/	NA	PY	PY
	4.4 If Y/PY/NI to 4.3: Is it likely that missingness in the outcome depended on its true value?	PN	NA	/	NA	/	NA	/	NA	PN	PN
	**Risk-of-bias judgment**	some concerns	Low	Low	Low	Low	Low	Low	Low	some concerns	some concerns
**5 Bias in measurement of the outcome**	5.1 Was the method of measuring the outcome inappropriate?	N	N	N	N	N	N	N	PN	PN	PN
	5.2 Could measurement or ascertainment of the outcome have differed between intervention groups?	N	N	N	N	N	PN	N	PN	PN	PN
	5.3 If N/PN/NI to 5.1 and 5.2: Were outcome assessors aware of the intervention received by study participants?	Y	Y	N	Y	Y	N	Y	Y	Y	Y
	5.4 If Y/PY/NI to 5.3: Could assessment	PN	NI	/	NI	PN	NA	PN	PN	PN	PN

	of the outcome have been influenced by knowledge of intervention received?										
	5.5 If Y/PY/NI to 5.4: Is it likely that assessment of the outcome was influenced by knowledge of intervention received?	/	PN	/	PN	/	NA	/	NA	NA	NA
	**Risk-of-bias judgment**	Low	some concerns	Low	some concerns	Low	Low	Low	some concerns	some concerns	some concerns
**6 Bias in selection of the reported result**	6.1 Were the data that produced this result analysed in accordance with a prespecified analysis plan that was finalised before unblinded outcome data were available for analysis?	Y	Y	Y	Y	Y	Y	Y	PY	PY	N
	6.2 Is the numerical result being assessed likely to have been selected, on the basis of the results, from: multiple eligible outcome measurements (eg, scales, definitions, time points) within the outcome domain?	N	N	N	N	N	N	N	N	N	Y
	6.3 Is the numerical result being assessed likely to have been selected, on the basis of the results, from: multiple eligible analyses of the data?	N	N	N	N	N	N	N	PN	PN	Y
	Risk-of-bias judgment	Low	Low	Low	Low	Low	Low	Low	Low	Low	High
**Overall bias**	**Low/High/Some concerns**	some concerns	some concerns	some concerns	some concerns	some concerns	Low	some concerns	some concerns	some concerns	High

Y=yes; PY=probably yes; PN=probably no; N=no; NA=not applicable; NI=no information.

*Signalling questions for bias due to deviations from intended interventions relate to the effect of assignment to intervention.

Assessing risk of bias in non-randomised studies of interventions (ROBINS-I)

Bias domain	Signalling questions	No.23	No.31	No.33	No.32	No.30	No.19	No.20	No.21	No.43	No.65	No.42	No.44	No.51	No.27
**1 Bias due to confounding**	1.1 Is there potential for confounding of the effect of intervention in this study? (If N/PN to 1.1: the study can be considered to be at low risk of bias due to confounding and no further signalling questions need be considered)	Y	Y	Y	Y	Y	PY	PY	PY	PY	PY	PY	PY	PY	PY
	1.2 Was the analysis based on splitting participants' follow up time according to intervention received? (If N/PN, answer questions relating to baseline confounding (1.4 to 1.6) , If Y/PY, go to question 1.3.)	N	N	N	N	N	N	N	N	N	N	N	PY	N	N
	1.3 Were intervention discontinuations or switches likely to be related to factors that are prognostic for the outcome? (If N/PN, answer questions relating to baseline confounding (1.4 to 1.6) If Y/PY, answer questions relating to both baseline and	NA	NA	NA	NA	NA	/	/	/	/	/	/	PY	/	/

	time-varying confounding (1.7 and 1.8))														
	**Questions relating to baseline confounding only**														
	1.4 Did the authors use an appropriate analysis method that controlled for all the important confounding domains?	N	N	PY	N	PY	N	N	N	N	N	N	/	N	N
	1.5 If Y/PY to 1.4: Were confounding domains that were controlled for measured validly and reliably by the variables available in this study?	NA	NA	PY	NA	PY	/	/	/	/	/	/	/	/	/
	1.6 Did the authors control for any post- intervention variables that could have been affected by the intervention?	N	N	N	N	N	N	N	N	N	N	N	/	N	PY
	**Questions relating to baseline and time-varying confounding**														
	1.7 Did the authors use an appropriate analysis method that controlled for all the important confounding domains and for time-varying confounding?	N	N	N	N	PY	N	N	N	N	N	N	PY	N	N
	1.8 If Y/PY to 1.7: Were confounding domains that were controlled for measured validly and reliably by the variables	NA	NA	NA	NA	NI	/	/	/	/	/	/	PY	/	/

	available in this study?														
	**Risk of bias judgement**	Serious	Serious	Serious	Serious	Moderate	Serious	Serious	Serious	Serious	Serious	Serious	Moderate	Serious	Serious
**2 Bias in selection of participants into the study**	2.1 Was selection of participants into the study (or into the analysis) based on participant characteristics observed after the start of intervention? (If N/PN to 2.1: go to 2.4)	N	N	N	N	N	N	N	Y	N	Y	N	Y	Y	Y
	2.2 If Y/PY to 2.1: Were the post-intervention variables that influenced selection likely to be associated with intervention?	NA	NA	NA	NA	NA	/	/	PN	/	PN	/	PN	PN	PN
	2.3 If Y/PY to 2.2: Were the postintervention variables that influenced selection likely to be influenced by the outcome or a cause of the outcome?	NA	NA	NA	NA	NA	/	/	PN	/	PN	/	/	/	/
	2.4 Do start of follow-up and start of intervention coincide for most participants?	Y	N	N	N	N	PY	PY	PY	PY	PY	PY	PY	PY	PY
	2.5 If Y/PY to 2.2 and 2.3, or N/PN to 2.4: Were adjustment techniques used that are likely to correct for the presence of selection biases?	NA	NA	NA	NA	NA	/	/	/	/	/	/	/	/	/

	**Risk of bias judgement**	Low	Moderate	Moderate	Moderate	Moderate	Low	Low	Low	Low	Low	Low	Low	Low	Low
**3 Bias in classification of interventions**	3.1 Were intervention groups clearly defined?	Y	Y	Y	Y	Y	Y	Y	Y	Y	Y	Y	Y	Y	Y
	3.2 Was the information used to define intervention groups recorded at the start of the intervention?	Y	Y	Y	Y	Y	Y	Y	Y	Y	Y	Y	PN	Y	PN
	3.3 Could classification of intervention status have been affected by knowledge of the outcome or risk of the outcome?	N	N	N	N	N	N	N	N	N	N	N	PY	N	N
	**Risk of bias judgement**	Low	Low	Low	Low	Low	Low	Low	Low	Low	Low	Low	Low	Low	Low
**4 Bias due to deviations from intended interventions**	**If your aim for this study is to assess the effect of assignment to intervention, answer questions 4.1 and 4.2**														
	4.1 Were there deviations from the intended intervention beyond what would be expected in usual practice?	N	N	N	N	N	N	N	N	N	N	N	N	N	N
	4.2 If Y/PY to 4.1: Were these deviations from intended intervention unbalanced between groups and likely to have affected the outcome?	NA	NA	NA	NA	NA	/	/	/	/	/	/	/	/	/
	**If your aim for this study is to assess the effect of starting and adhering to intervention, answer questions 4.3 to 4.6**														
	4.3 Were important co-interventions balanced across	NA	NA	NA	NA	NA	PN	PN	PY	PY	PN	PN	PN	PY	PN

	intervention groups?														
	4.4 Was the intervention implemented successfully for most participants?	NA	NA	NA	NA	NA	Y	Y	Y	Y	Y	Y	Y	Y	Y
	4.5 Did study participants adhere to the assigned intervention regimen?	NA	NA	NA	NA	NA	PY	PY	PY	PY	PY	PY	PY	PY	PY
	4.6 If N/PN to 4.3, 4.4 or 4.5: Was an appropriate analysis used to estimate the effect of starting and adhering to the intervention?	NA	NA	NA	NA	NA	PN	PN	/	/	PN	PN	/	/	PN
	**Risk of bias judgement**	Low	Low	Low	Low	Low	Moderate	Moderate	Low	Low	Moderate	Moderate	Moderate	Low	Moderate
**5 Bias due to missing data**	5.1 Were outcome data available for all, or nearly all, participants?	PY	Y	Y	Y	Y	Y	Y	Y	Y	Y	Y	Y	Y	Y
	5.2 Were participants excluded due to missing data on intervention status?	PN	N	N	N	N	N	N	N	N	N	N	N	N	N
	5.3 Were participants excluded due to missing data on other variables needed for the analysis?	PN	N	N	N	N	N	N	N	N	N	N	N	N	N
	5.4 If PN/N to 5.1, or Y/PY to 5.2 or 5.3: Are the proportion of participants and reasons for missing data similar across interventions?	NA	NA	NA	NA	NA	/	/	/	/	/	/	/	/	/

	5.5 If PN/N to 5.1, or Y/PY to 5.2 or 5.3: Is there evidence that results were robust to the presence of missing data?	NA	NA	NA	NA	NA	/	/	/	/	/	/	/	/	/
	**Risk of bias judgement**	Low	Low	Low	Low	Low	Low	Low	Low	Low	Low	Low	Low	Low	Low
**6 Bias in measurement of outcomes**	6.1 Could the outcome measure have been influenced by knowledge of the intervention received?	PN	PN	PN	PN	PN	Y	PN	PN	PN	PN	PY	PY	PN	PY
	6.2 Were outcome assessors aware of the intervention received by study participants?	Y	Y	Y	Y	Y	Y	Y	Y	Y	Y	Y	Y	Y	Y
	6.3 Were the methods of outcome assessment comparable across intervention groups?	Y	Y	Y	Y	Y	PY	PY	Y	Y	Y	PY	Y	Y	Y
	6.4 Were any systematic errors in measurement of the outcome related to intervention received?	PN	PN	PN	PN	PN	PY	PY	PN	PN	PN	PY	PY	PN	PN
	**Risk of bias judgement**	Moderate	Moderate	Moderate	Moderate	Moderate	Moderate	Moderate	Low	Low	Low	Moderate	Moderate	Low	Moderate
**7 Bias in selection of the reported result**	Is the reported effect estimate likely to be selected, on the basis of the results, from:						N	N	N	N	N	N	N	N	N
	7.1 ...multiple outcome measurements within the outcome domain?	N	N	N	N	N									

	7.2 ...multiple analyses of the intervention-outcome relationship?	N	N	N	N	N									
	7.3 ... different subgroups?	N	N	N	N	N									
	**Risk of bias judgement**	Low	Low	Low	Low	Low	Low	Low	Low	Low	Low	Low	Low	Low	Low
**Overall bias**	**Low/Moderate/Serious/Critical/ NI**	Serious	Serious	Serious	Serious	Moderate	Serious	Serious	Serious	Serious	Serious	Serious	Moderate	Serious	Serious

Y=yes; PY=probably yes; PN=probably no; N=no; NA=not applicable; NI=no information.
Low ow risk of bias: The study is judged to be at low risk of bias for all domains.
Moderate risk of bias: The study is judged to be at low or moderate risk of bias for all domains.
Serious risk of bias: The study is judged to be at serious risk of bias in at least one domain, but not at critical risk of bias in any domain.
Critical risk of bias: The study is judged to be at critical risk of bias in at least one domain.
No information: There is no clear indication that the study is at serious or critical risk of bias and there is a lack of information in one or more key domains of bias (a judgement is required for this).

GRADE Evidence Profile of the Rituximab Guidelines

Table 1 Summary of evidence for the effect of RTX treatment on clinical outcomes in SSNS aged 1-18 years based on RCTs, NRCTs and cohort studies

Outcome<br>No. of participants<br>Study Type(N)	Relative effect<br>(95% CI)	Anticipated absolute effects (n/N)			Quality of evidence<br>(GRADE)	Comments
		Comparison Group	Experimental Group	Difference		
**Relapse rate**						
**Population: FRNS/SDNS-; Intervention: RTX; Comparison: placebo/steroid/CNI/CTX; 7 RCTs[12,13,14,15,16,17,18], 2 cohort studies[20,21], 1 NRCT[19]; 432 cases**						
12-month follow-up	**invention/control**<br>**FRNS/SDNS**<br>（77/220；133/212）：<br>OR =0.18，95%CI:0.07~0.44<br>**FRNS/SDNS+**<br>（52/92；63/75）：<br>OR =0.19，95%CI:0.05~0.68<br>**FRNS/SDNS-**<br>（25/128；70/137）：<br>OR =0.15, 95%CI:0.03~0.68<br>**RTX vs placebo/blank control:**<br>（46/86；66/69）<br>OR =0.05，95%CI:0.01~0.18<br>**RTX vs CTX/CNI/MMF**：<br>（31/134；67/143）<br>OR =0.32，95%CI:0.13~0.82	23/24[12]<br>15/15[13]<br>22/60[14]<br>14/15[15]<br>9/20[16]<br>14/15[17]<br>15/15[18]<br>10/27[19]<br>7/13[20]<br>6/8[21]	17/24[12]<br>5/14[13]<br>6/60[14]<br>2/15[15]<br>9/20[16]<br>22/33[17]<br>2/15[18]<br>3/19[19]<br>5/10[20]<br>6/10[21]	**FRNS/SDNS**：<br>395 fewer per 1,000<br>(from 522 fewer to 202 fewer)<br>**FRNS/SDNS+**：<br>341 fewer per 1,000<br>(from 632 fewer to 59 fewer)<br>**FRNS/SDNS-**：<br>375 fewer per 1,000<br>(from 481 fewer to 96 fewer)<br>**RTX vs placebo/blank control:**<br>433 fewer per 1,000<br>(from 776 fewer to 158 fewer)<br>**RTX vs CTX/CNI/MMF**：<br>248 fewer per 1,000<br>(from 366 fewer to 49 fewer)	**Very low**<br>⊕○○○	**Downgrade reasons:**<br>-Risk of bias ↓ 2 levels<br>-Inconsistency ↓ 2 levels<br>*[different populations, study types, dosages of intervention and controls]*<br>-Imprecision ↓ 2 levels<br>*[clinical experience 60~80%]*<br>**Not downgrade:**<br>- Publication of bias<br>- Indirectness of evidence<br>**No upgrade reasons**
**Population: FRNS/SDNS; Intervention: RTX; Comparison: placebo/steroid/CNI/CTX; 1 RCT[13], 2 cohort studies[21,33]; 163 cases**						
24-month follow-up	**FRNS/SDNS**：<br>（44/61；80/102）	15/15[13]<br>7/8[21]	6/9[13]<br>9/10[21]	**FRNS/SDNS**：<br>82 fewer per 1,000	**Very low**<br>⊕○○○	**Downgrade reasons:**<br>-Risk of bias ↓ 2 levels

	OR=0.65，95%CI:0.31~1.35	58/79[33]	29/42[33]	(from 254 fewer to 46 more)		-Inconsistency ↓1 level<br>*[different controls]*<br>-Imprecision ↓2 levels<br>*[clinical experience 60~70%]*<br>**Not downgrade:**<br>-Indirectness of evidence<br>**Not applicable:**<br>-Publication of bias<br>**No upgrade reasons**
**The median time to first relapse (with a follow-up of ≥ 12 months)**						
**Population: FRNS/SDNS-; Intervention: RTX; Comparison: CNI/CTX; 2 RCTs[13,14], 1 NRCT [19]; 196 cases**						
the median time to first relapse	**FRNS/SDNS-:**<br>Median survival ratio(MSR) = 1.93, 95%CI:1.62~2.30, P<0·0001<br>Heterogeneity test Q=112.18，P<0·0001	1.2 months（15/15） [13]<br>7.3 months（38/60）[14]<br>6.9）months（10/27 [19]	18.4 months（6/15） [13]<br>10 months（54/60）[14]<br>6.3 months（3/19） [19]	/	**Very low**<br>⊕○○○	**Downgrade reasons:**<br>-Risk of bias↓2 levels<br>-Inconsistency↓2 levels<br>*[different interventions, controls and types]*<br>-Imprecision↓2 levels<br>*[2~3months]*<br>**Not downgrade:**<br>-Indirectness of evidence<br>**Not applicable:**<br>-Publication of bias<br>**No upgrade reasons**
**Population: FRNS/SDNS+; Intervention: RTX; Comparison: placebo; 1 RCT[12]; 48 cases**						
the median time to first relapse	/	101（95%CI：70-155)d (23/24)	267(95%CI:223-374)d (17/24)	HR=0.27(0.14-0.53) p<0·0001	**Moderate**<br>⊕⊕⊕○	**Downgrade reasons:**<br>-Risk of bias↓1 level<br>**Not downgrade:**<br>- Imprecision<br>- Indirectness of evidence<br>**Not applicable:**<br>-Inconsistency<br>-Publication of bias<br>**No upgrade reasons**
the time from steroid reduction to first relapse	/	42(95% CI 14–98)d (23/24)	211(95% CI 166–317)d (17/24)	HR=0.27, 95% CI：0.14–0.54; p<0·0001		
**Population: FRNS/SDNS+; Intervention: RTX; Comparison: CNI; 1 cohort study[20]; 23 cases**						

the median time to first relapse	/	(9.8 ± 5.6) months (7/13)	(8.5 ± 5.1) months (5/10)	Difference value 1.3 ± 2.8， P=0.65	**Very low** ⊕○○○	**Downgrade reasons:**<br>-Risk of bias ↓ 2 levels<br>-Imprecision ↓ 1 level<br>*[clinical experience 9~10 months]*<br>**Not downgrade:**<br>-Indirectness of evidence<br>**Not applicable:**<br>-Inconsistency<br>-Publication of bias<br>**No upgrade reasons**
**Population: FRNS/SDNS; Intervention: RTX; Comparison: CNI; 1 cohort study[21]; 18 cases**						
the median time to first relapse (at 24-month follow-up)	/	(10.1±4.9) months (7/8)	(9.1±4.4) months (9/10)	*P*=0.72	**Very low** ⊕○○○	**Downgrade reasons:**<br>-Risk of bias ↓ 2 levels<br>-Imprecision ↓ 1 level<br>*[clinical experience 9~10 months]*<br>**Not downgrade:**<br>-Indirectness of evidence<br>**Not applicable:**<br>-Inconsistency<br>-Publication of bias<br>**No upgrade reasons**
**Population: FRNS/SDNS+; Intervention: RTX (2 doses); Comparison: RTX (1 dose); 1 cohort study[33]; 42 cases**						
the median time to first relapse	/	5 (1~36) months (8 cases)	16(1~73) months (34 cases)	*P*=0.03	**Very low** ⊕○○○	**Downgrade reasons:**<br>-Risk of bias ↓ 2 levels<br>-Imprecision ↓ 2 levels<br>*[clinical experience 9~10 months]*<br>**Not downgrade:**<br>-Indirectness of evidence<br>**Not applicable:**<br>-Inconsistency<br>-Publication of bias<br>**No upgrade reasons**
**Steroid dose (with a follow-up of > 3 months)**						
**Population: FRNS/SDNS; Intervention: RTX; Comparison: placebo/CNI; 3 RCTs[12,14,17], 1 cohort study[20]; 242 cases**						

1-year cumulative steroid dose (mg/kg/d)	**FRNS/SDNS:**<br>MD=-0.16,<br>95%CI:-0.20~-0.12,<br>I^2=76%,P<0.01<br>**vs FK:**<br>MD=-0.15,<br>95%CI:-0.18~-0.11<br>**vs placebo/blank control:**<br>MD=-0.26,<br>95%CI:-0.36~-0.16<br>**FRNS/SDNS+:**<br>MD=-0.14,<br>95%CI:-0.21~-0.08<br>**FRNS/SDNS-:**<br>MD=-0.17,<br>95%CI:-0.28~-0.07	20.85±9.28, 24 cases[12] mg/m2/d<br>86.3±58.0, 60 cases[14] mg/kg/y<br>0.40±0.28, 16 cases[17] mg/kg/d<br>70.9 ± 26.3, 13 cases[20] mg/kg/y	9.12±5.88, 24 cases [12] mg/m2/d<br>25.8±27.8, 60 cases [14] mg/kg/y<br>0.24±0.19, 35 cases [17] mg/kg/d<br>46.1 ± 42.1, 10 cases [20] mg/kg/y	/	**Very low**<br>⊕○○○	**Downgrade reasons:**<br>-Risk of bias ↓ 2 levels<br>-Inconsistency ↓ 2 levels *[different populations and controls]*<br>-Imprecision ↓ 2 levels *[clinical experience 0.1~0.2]*<br>**Not downgrade:**<br>-Indirectness of evidence<br>**Not applicable:**<br>-Publication of bias<br>**No upgrade reasons**
**Population: FRNS/SDNS; Intervention: post-RTX; Comparison: pre-RTX; 3 RCTs[12,14,17], 2 cohort studies[20,32]; 209 cases**						
1-year cumulative steroid dose (mg/kg/d)	**FRNS/SDNS+:**<br>MD=-0.35,<br>95%CI:-0.38~-0.31,<br>I^2=79%, P<0.01	19.13±9.94, 19 cases [12] mg/m2/d<br>246.0±48.0, 60 cases [14] mg/kg/y<br>0.46±0.4, 35 cases [17] mg/kg/d<br>140.5±59.0, 10 cases [20] mg/kg/y<br>148.1±82.3, 85 cases [32] mg/kg/y	8.37±5.62, 19 cases [12] mg/m2/d<br>86.3±58.0, 60 cases [14] mg/kg/y<br>0.24±0.19, 35 cases [17] mg/kg/d<br>46.1±42.1, 10 cases [20] mg/kg/y<br>43.6±36.6, 85 cases [32] mg/kg/y	/	**low**<br>⊕⊕○○	**Downgrade reasons:**<br>-Risk of bias ↓ 2 levels<br>-Inconsistency ↓ 2 levels *[different populations and controls]*<br>-Imprecision ↓ 1 level *[clinical experience 0.3~0.5]*<br>**Not downgrade:**<br>-Indirectness of evidence<br>**Not applicable:**<br>-Publication of bias<br>**No upgrade reasons**
**Population: FRNS/SDNS-; Intervention: RTX; Comparison: CNI; 1 RCT[16]; 41 cases**						
1-year cumulative steroid dose (mg/kg/d)	/	20 cases<br>median (IQR)<br>post 0.11 (0.04, 0.19)<br>pre 0.43(0.36,0.72)	21 cases<br>median (IQR)<br>post 0.11 (0.05, 0.24)<br>pre 0.44(0.29,0.68)	Risk or mean difference: –0.08 [–0.19,0.03], p=0.15	**low**<br>⊕⊕○○	**Downgrade reasons:**<br>-Risk of bias ↓ 1levels<br>-Imprecision ↓ 1 level

						[clinical experience 0.026 (0.018~0.3)]<br>**Not applicable:**<br>-Inconsistency<br>-Indirectness of evidence<br>-Publication of bias<br>**No upgrade reasons**
**Population: FRNS/SDNS-; Intervention: RTX; Comparison: CTX; 1 NRCT[19]; 46 cases**						
steroid dose at 12-month follow-up	/	27 cases<br>pre1.02 mg/kg qod<br>post 0.36 mg/kg qod<br>difference 0.66±0.63,<br>p<0.001	19 cases<br>pre 0.86 mg/kg qod<br>post 0.08 mg/kg qod<br>difference 0.78±0.23,<br>p<0.001	/	**Very low**<br>⊕○○○	**Downgrade reasons:**<br>-Risk of bias ↓ 2 levels<br>-Inconsistency ↓ 1 level<br>*[different populations]*<br>-Imprecision ↓ 1 level<br>*[clinical experience 0.026 (0.018~0.3)]*<br>**Not downgrade:**<br>-Indirectness of evidence<br>**Not applicable:**<br>-Publication of bias<br>**No upgrade reasons**
**Population: FRNS/SDNS+; Intervention: post-RTX; Comparison: pre-RTX; 1 case series report[36]; 10 cases**						
mean steroid dose with a follow-up of mean17(range13-21) months	/	0.39 ± 0.18 mg/kg/d	0.15 ± 0.14 mg/kg/d	decrease 63% , p <0.01	**Very low**<br>⊕○○○	/
**Steroid withdrawal**						
**Population: FRNS/SDNS; Intervention: RTX; Comparison: CNI/CTX; 1 RCT[37], 1 NRCT[19]; 100 cases**						
3-month steroid withdrawal rate	**FRNS /SDNS:**<br>（35/46；10/54）<br>OR=15.71（5.72~43.16）	8/27，29.6%[19]<br>2/27，7.4%[37]	14/19，73.7%[19]<br>21/27，77.8%[37]	**FRNS /SDNS:**<br>578 more per 1,000 (from 359 more to 712 more)	**Very low**<br>⊕○○○	**Downgrade reasons:**<br>-Risk of bias ↓ 2 levels<br>-Inconsistency ↓ 2 levels<br>*[different populations and controls]*<br>-Imprecision ↓ 2 levels<br>*[clinical experience 2-4 times]*<br>**Not downgrade:**

						-Indirectness of evidence<br>**Not applicable:**<br>-Publication of bias<br>**No upgrade reasons**
**Population: FRNS/SDNS+; Intervention: RTX; Comparison: CNI; 1 RCT[17]; 51 cases**						
Steroid-free period (d/y) with a 12-month follow-up	/	16 cases<br>post 80.2±98.5，<br>pre 142.9 ± 117.7	35 cases<br>post 140.5±91.3<br>pre 99.1±111.8	p=0.12<br>p=0.17	**low**<br>⊕⊕○○	**Downgrade reasons:**<br>-Risk of bias ↓ 1 level<br>-Imprecision ↓ 2 levels<br>*[clinical experience 210 days]*<br>**Not downgrade:**<br>-Inconsistency<br>**Not applicable:**<br>-Indirectness of evidence<br>-Publication of bias<br>**No upgrade reasons**
**Population: FRNS/SDNS; Intervention: RTX; Comparison: CNI/CTX; 1 RCT[14], 1 cohort study[33]; 238 cases**						
1-year steroid withdrawal rate (range 12-90 months)	**FRNS /SDNS:**<br>（91/101； 113/137）<br>OR=1.93 (0.87~4.29)	46/58, 79.3%[14],<br>67/79, 84.8%[33]，<br><br>median time to steroid withdrawal 3 months<br>median time to first relapse from steroid withdrawal 3 months<br>relapse with steroid using 12/79[33]	55/59, 93.2%[14],<br>36/42, 85.7%[33]，<br><br>median time to steroid withdrawal 3 months<br>median time to first relapse from steroid withdrawal 12 months<br>relapse with steroid using 6/42[33]	**FRNS /SDNS:**<br>74 more per 1,000<br>(from 23 fewer to 126 more)	**Very low**<br>⊕○○○	**Downgrade reasons:**<br>-Risk of bias ↓ 2 levels<br>-Inconsistency ↓ 3 levels<br>*[different populations, controls and study types]*<br>-Imprecision ↓ 2 levels<br>*[clinical experience 1~2]*<br>**Not downgrade:**<br>-Indirectness of evidence<br>**Not applicable:**<br>-Publication of bias<br>**No upgrade reasons**
**Population: FRNS/SDNS; Intervention: RTX; Comparison: CNI; 1 cohort study[21]; 18 cases**						
Steroid-free period mean ± SD with a follow-up of ≥ 12 months	/	8 cases<br>3.20 ± 3.00 months	10 cases<br>7.30 ± 7.40 months	p=0.2	**Very low**<br>⊕○○○	**Downgrade reasons:**<br>-Risk of bias ↓ 2 levels<br>-Inconsistency ↓ 1 level<br>*[different populations]*<br>-Imprecision ↓ 1 level

						[clinical experience 210 days]<br>**Not applicable:**<br>-Indirectness of evidence<br>-Publication of bias<br>**No upgrade reasons**
**Population: FRNS/SDNS+; Intervention: RTX+MMF; Comparison: RTX+placebo; 1 RCT[41]; 57 cases**						
median time to treatment failure (d) with a follow-up of ≥ 16.8 months	/	21/29 (8 censored)<br>402(327-574)d	18/28 (10 censored)<br>754(543-997)d	HR= 0.55 (0.31-0.98)	**Moderate**<br>⊕⊕⊕○	**Downgrade reasons:**<br>-Inconsistency ↓ 1 level<br>*[different controls]*<br>**Not downgrade:**<br>-Risk of bias<br>**Not applicable:**<br>-Indirectness of evidence<br>-Imprecision<br>-Publication of bias<br>**No upgrade reasons**

Table 2 Summary of evidence for the effect of RTX treatment on clinical outcomes in SSNS aged 1-18 years based on single-arm data of RCTs, NRCTs, cohort studies and case series reports

Outcome	Study Type(N)	n/N	Proportion (95%CI)	Quality of evidence (GRADE)	Comments
**Relapse rate**					
**Population: FRNS/SDNS; 6 RCTs[12,13,14,15,17,18], 1 NRCT[19], 4 cohort studies[23,30,31,32], 8 case series reports[22,23,24,25,26,27,28,29]; 386 cases**					
12-month follow-up	6 RCTs[12,13,14,15,17,18]<br>1NRCT[19]<br>4 cohort studies[23,30,31,32],<br>8 case series reports<br>[22,23,24,25,26,27,28,29]	17/34[22]<br>10/17[23]<br>9/37[24]<br>14/43[25]<br>8/28[26]<br>8/18[27]<br>3/7[28]<br>37/61[29]<br>7/10[30]	**RTX:**<br>41%(95%CI:31%~52%)<br>**FRNS/SDNS+:**<br>47% (95%CI:36%~59%)<br>**FRNS/SDNS-:**<br>22% (95%CI:9%~37%)	**Very low**<br>⊕○○○	**Downgrade reasons:**<br>-Risk of bias ↓ 2 levels<br>-Inconsistency ↓ 3 levels<br>*[different populations, controls and study types]*<br>-Imprecision ↓ 1 level<br>*[clinical experience 35%]*<br>**Not downgrade:**<br>-Indirectness of evidence

		37/46[31]<br>12/85[32]			**Not applicable:**<br>-Publication of bias<br>**No upgrade reasons**
**Population: FRNS/SDNS; 1 RCT[15], 1 cohort study[31], 5 case series reports[22,24,25,26,34]; 294 cases**					
24-month follow-up	1 RCT[15]<br>1 cohort study[31]<br>5 case series reports[22,24,25,26,34]	7/15 [15]<br>21/27[22]<br>17/29[24]<br>27/43[25]<br>14/28[26]<br>36/40[31]<br>47/51[34]	**RTX:**<br>72%(95%CI:61% ~ 83%)<br>**FRNS/SDNS+:**<br>75% (95%CI:63%~86%)<br>**FRNS/SDNS-:**<br>54% (95%CI:33%~75%)	**Very low**<br>⊕○○○	**Downgrade reasons:**<br>-Risk of bias ↓ 2 levels<br>-Inconsistency ↓ 3 levels<br>*[different populations, controls and study types]*<br>-Imprecision ↓ 1 level<br>*[clinical experience 35%]*<br>**Not downgrade:**<br>-Indirectness of evidence<br>**Not applicable:**<br>-Publication of bias<br>**No upgrade reasons**
**Population: FRNS/SDNS; 1 RCT[15], 2 case series reports[22, 34]; 91 cases**					
36-month follow-up	1 RCT[15]<br>2 case series reports[22, 34]	7/15 人[15]<br>23/25[22]<br>47/51[34]	**FRNS/SDNS:**<br>85%(95%CI:76% ~ 91%)<br>**FRNS/SDNS+**:<br>92% (95%CI:84%~96%)<br>**FRNS/SDNS -:**<br>47% (95%CI:21%~73%)	**Very low**<br>⊕○○○	**Downgrade reasons:**<br>-Risk of bias ↓ 2 levels<br>-Inconsistency ↓ 3 levels<br>*[different populations, controls and study types]*<br>-Imprecision ↓ 1 level<br>*[clinical experience 35%]*<br>**Not downgrade:**<br>-Indirectness of evidence<br>**Not applicable:**<br>-Publication of bias<br>**No upgrade reasons**
**Population: FRNS/SDNS; 1 RCT[15]; 15 cases**					
48-month follow-up	1 RCT[15]	7/15[15]	/	**Low**<br>⊕⊕○○	**Downgrade reasons:**<br>-Risk of bias ↓ 1 level<br>-Imprecision ↓ 1 level<br>*[clinical experience 35%]*

					**Not downgrade:**<br>-Indirectness of evidence<br>**Not applicable:**<br>-Inconsistency<br>-Publication of bias<br>**No upgrade reasons**
**The time to first relapse (with a follow-up of ≥ 12 months)**					
**Population: FRNS/SDNS; 3 cohort studies[20,31,33], 6 case series reports[22,24,25,26,27,38]; 461 cases**					
The time to first relapse	3 cohort studies[20,31,33]<br>6 case series reports[22,24,25,26,27,38]	Mean±SD:<br>8.5±5.1 5/10 [21]<br>10.23±9.25 32/39 [22]<br>Median(range):<br>9.25 (0.5-36) 32/39 [22],<br>9.6(5.2-64.1) 24/37 [24],<br>19.53 (0.07-48.33) 39/43 [25]<br>14 (1-73) 26/38 [33],<br>10 (0-40) 59/81 [38]<br>Median(IQR):<br>11.2(8-17.7) 28 [26]<br>6.45(4.48-8.62) 18 [27]<br>5.6(4.3 8.1) 46 [31]	9.89 (95%CI:7.14~12.65) months	**Very low**<br>⊕○○○	**Downgrade reasons:**<br>-Risk of bias ↓ 2 levels<br>-Inconsistency ↓ 3 levels<br>*[different populations, controls and study types]*<br>-Imprecision ↓ 2 levels<br>*[clinical experience 9~10 months]*<br>**Not downgrade:**<br>-Indirectness of evidence<br>**Not applicable:**<br>-Publication of bias<br>**No upgrade reasons**
**Population: FRNS/SDNS+; 1 case series report[34]; 51 cases**					
The median time to first relapse with a follow-up of ≥ 36 months	1 case series report[34]	261d	/	**Very low**<br>⊕○○○	/
**The time to steroid withdrawal**					
**Population: FRNS/SDNS+; 1 cohort study[34]; 17 cases**					
3-month steroid withdrawal rate	1 cohort study[23]	17/17	/	**low**<br>⊕⊕○○	**Downgrade reasons:**<br>-Risk of bias ↓ 2 levels<br>**Not downgrade:**<br>-Inconsistency<br>-Indirectness of evidence<br>**Not applicable:**

					-Imprecision<br>*[clinical 3months]*<br>-Publication of bias<br>**No upgrade reasons**
**Population: FRNS/SDNS+; 2 cohort studies[23,31], 1 case series report[28]; 70 cases**					
6-month steroid withdrawal rate	2 cohort studies[23,31]<br>1 case series report[28]	17/17 (100%)[23]<br>2/7 (29%) [28]<br>25/46 (54%) [31]	63% (95%CI:51%~73%)	**Very low**<br>⊕○○○	**Downgrade reasons:**<br>-Risk of bias ↓ 2 levels<br>-Inconsistency ↓ 1 level<br>*[different study types]*<br>-Imprecision ↓ 1 level<br>*[clinical experience 3 months]*<br>**Not downgrade:**<br>-Indirectness of evidence<br>**Not applicable:**<br>-Publication of bias<br>**No upgrade reasons**
**Population: FRNS/SDNS+; 1 case series report[38]; 81 cases**					
The time to steroid withdrawal with a follow-up of 13–90 months	1 case series report[38]	69/81(85%)<br>Median 66.5(26-409) d	/	**Very low**<br>⊕○○○	/
**Population: FRNS/SDNS; 1 cohort study[32]; 101 cases**					
mean follow-up 30.6 ± 19.1(15–43) months<br>last valid follow-up 22(14–39) months	1 cohort study[32]	/	90/101 steroid withdrawal at 4.8 ± 2.0 months	**Very low**<br>⊕○○○	**Downgrade reasons:**<br>-Risk of bias ↓ 2 levels<br>-Inconsistency ↓ 1 level<br>*[different populations]*<br>-Imprecision ↓ 1 level<br>*[clinical experience 3 months]*<br>**Not downgrade:**<br>-Indirectness of evidence<br>**Not applicable:**<br>-Publication of bias<br>**No upgrade reasons**
**Population: FRNS/SDNS+; 1 case series report[24]; 37 cases**					

median follow-up 29.4 (range 9.2–92.8) months, Mean (SD) 32.7 (15.1)	1 case series report[24]	/	24/37 relapse<br>35/37 (94.5%) steroid withdrawal at 1.3(0.37-6) months	**Very low**<br>⊕○○○	/

Table 3 Summary of evidence for the effect of consolidation therapy on clinical outcomes after RTX treatment in SSNS aged 1-18 years based on RCTs, NRCTs, cohort studies and case series reports

**Outcome<br>No. of participants<br>Study Type(N)**	**Relative effect<br>(95% CI)**	**Anticipated absolute effects (n/N)**			**Quality of evidence (GRADE)**	**Comments**
		**Comparison Group**	**Experimental Group**	**Difference**		
**Relapse of RTX+MMF with a follow-up of ≥ 12 months**						
**Population: FRNS/SDNS; Intervention: RTX+MMF; Comparison: RTX+blank/placebo; RTX(375mg/m² qw×4w)[41], RTX(375mg/m² qw×1~2 doses)[22]; 1 RCT[41], 1 case series report[22]; 112 cases**						
The time to first relapse	MSR=2.66，95%CI:2.14~3.30, P=0.01,<br>Q=13.59 P<0·000	60(27,130)d 11/13[22],<br>320(266,1060)d 27/39[41]	450(332,672)d 13/21[22]<br>654(500,858)d 11/39[41]	/	**Very low**<br>⊕○○○	**Downgrade reasons:**<br>-Risk of bias ↓ 2 levels<br>-Inconsistency ↓ 1 level *[different study types]*<br>-Imprecision ↓ 2 levels<br>**Not downgrade:**<br>-Indirectness of evidence<br>**Not applicable:**<br>-Publication of bias<br>**No upgrade reasons**
**Population: FRNS/SDNS+; Intervention: RTX+MMF; Comparison: RTX; 1 NRCT[42]; 16 cases**						
Annual relapse number	/	2.3 relapses/y 6/7	0.42 relapses/y 3/9	P < 0.001	**low**<br>⊕⊕○○	**Downgrade reasons:**<br>-Risk of bias ↓ 2 levels<br>**Not downgrade:**<br>-Indirectness of evidence<br>**Not applicable:**<br>-Inconsistency<br>-Imprecision

						-Publication of bias (Not applicable)<br>**No upgrade reasons**
**Population: FRNS/SDNS+; Intervention: RTX+MMF; Comparison: RTX+placebo/Csa/IS withdrawal; 1RCT[41], 2 NRCT[42,43], 2 case series reports[22,36]; 167 cases**						
Relapse rate	**RTX+MMF vs RTX+placebo/CsA/IS withdrawl** (34/87；50/80):<br>OR=0.25，95%CI：0.06~1.07<br>**RTX+MMF vs RTX+CsA**(7/18；3/18):<br>OR=2.89，95%CI：0.59~14.11<br>**RTX+MMF vs RTX+placebo/IS withdrawl** (27/71；47/62):<br>OR=0.16，95%CI：0.07~0.35	11/13(IS withdrawl)[22]<br>3/3(IS withdrawl)[36]<br>1/5(CsA)[36]<br>27/39(placebo)[41]<br>6/7(IS withdrawl)[42]<br>2/13(CsA)[43]	13/21[22]<br>0/2[36]<br>11/39[41]<br>3/9[42]<br>7/16[43]	**RTX+MMF vs RTX+placebo/CsA/IS withdrawl:**<br>333 fewer per 1,000 (from 549 fewer to 15 more)<br>**RTX+MMF vs RTX+CsA:**<br>200 more per 1,000 (from 61 fewer to 572 more)<br>**RTX+MMF vs RTX+placebo/IS withdrawl:**<br>424 fewer per 1,000 (from 578 fewer to 235 fewer)	**Very low**<br>⊕○○○	**Downgrade reasons:**<br>-Risk of bias ↓ 2 levels<br>-Inconsistency ↓ 2 levels<br>*[different controls and study types]*<br>-Imprecision ↓ 2 levels<br>**Not downgrade:**<br>-Indirectness of evidence<br>**Not applicable:**<br>-Publication of bias<br>**No upgrade reasons**
**Steroid dose of RTX+MMF with a follow-up of ≥ 12 months**						
**Population: FRNS/SDNS+; Intervention: RTX+MMF; Comparison: RTX+blank/placebo; 1 RCT[41], 1 NRCT[42]; 94 cases**						
Steroid dose	**FRNS/SDNS+:**<br>MD=-5.52,95%CI:-7.34~-3.70	10.45±12.49 mg/m²/d[41]<br>8.7±2.7 mg/m²/d[42]	4.45±3.52 mg/m²/d[41]<br>3.3±0.6 mg/m²/d[42]	/	**Low**<br>⊕⊕○○	**Downgrade reasons:**<br>-Risk of bias ↓ 2 levels<br>-Inconsistency ↓ 1 levels<br>*[different study types]*<br>**Not downgrade:**<br>-Imprecision<br>*[clinical experience 0.01]*<br>-Indirectness of evidence<br>**Not applicable:**<br>-Publication of bias<br>**No upgrade reasons**

**Population: FRNS/SDNS+; Intervention: RTX+MMF (initial steroid dose 0.38mg/kg/d); Comparison: RTX+CsA (initial steroid dose 0.35mg/kg/d); 1 NRCT[43]; 29 cases**						
Steroid dose	SMD=0.5,95%CI: -0.25~1.24	0.057 mg/kg/d ($p < 0.01$)	0.15 mg/kg/d ($p < 0.01$)	/	**Very low**<br>⊕○○○	**Downgrade reasons:**<br>-Risk of bias ↓ 2 levels<br>-Inconsistency ↓ 1 level<br>*[different controls]*<br>-Imprecision ↓ 2 levels<br>*[clinical experience 0.01]*<br>**Not downgrade:**<br>-Indirectness of evidence<br>**Not applicable:**<br>-Publication of bias<br>**No upgrade reasons**
**Relapse of RTX+RTX with a follow-up of ≥ 12 months**						
**Population: FRNS/SDNS+; Intervention: RTX+RTX(a repeated course); Comparison: RTX; 1 cohort study[44]; 61 cases**						
50% relapse-free survival time	**FRNS/SDNS+:**<br>p = 0.001 (compared to the second dose)<br>p = 0.0005 (compared to the first dose)	42/45(93%) relapse<br>50% relapse-free survival was 335 days after RTX	9/16(56%) relapse<br>50% relapse-free survival time was 954 days after the first dose of RTX and 667 days after the second dose of RTX	/	**Moderate**<br>⊕⊕⊕○	**Downgrade reasons:**<br>-Risk of bias ↓ 1 level<br>**Not downgrade:**<br>-Inconsistency<br>-Indirectness of evidence<br>**Not applicable:**<br>-Imprecision<br>-Publication of bias<br>**No upgrade reasons**
**Quality of life**						
**Population: FRNS/SDNS+; Intervention: RTX (375mg/m², four times semiannually) followed by mizoribine (twice a week at a dose of 500 mg on the first day and 550 mg on the second day); 1 case series report[45]; 22cases**						
Quality of life scores measured by PedsQL4.0	/	Patients<br>Baseline 81.1(95%CI:74.6~87.5)<br>24-month 91.5(95%CI:85.1~97.9), *P<0.001*<br>Parents<br>Baseline 74.9(95%CI:68.5~81.3)<br>24-month 85.2(95%CI:78.8~91.7), *P<0.001*		/	**Very low**<br>⊕○○○	/

Table 4 Summary of evidence for the effect of RTX re-exposure on clinical outcomes in SSNS aged 1-18 years relapsing after RTX intervention based on RCTs, NRCTs, cohort studies and case series reports

Outcome<br>No. of participants<br>Study Type(N)	Relative effect<br>(95% CI)	Anticipated absolute effects (n/N)			Quality of evidence(GRADE)
		Comparison Group	Experimental Group	Difference	
Relapse of RTX re-exposure in relapsed FRNS/SDNS+ after RTX intervention with a follow-up of ≥ 12 months					
**Population: FRNS/SDNS+; Intervention: RTX (375 mg/m², 2 doses)+RTX (375 mg/m², 1~2 doses); 1 case series report[23]; 10 cases**					
Relapse with a follow-up of ≧ 6 months	/	/	10/10 no relapse	/	**Very low**<br>⊕○○○
**Population: FRNS/SDNS+; Intervention: RTX (375 mg/m², 1~2 doses)+RTX (1~3 courses); 1 case series report[46]; 5 cases**					
Relapse with a follow-up of 24 months	/	/	5/5 no relapse	/	**Very low**<br>⊕○○○
**Population: FRNS/SDNS+; Intervention: RTX (375 mg/m², 1~2 doses)+RTX (375 mg/m², 1 dose); 1 case series report[31]; 46 cases**					
The median time to relapse after IS withdrawal	/	39 cases<br>5.6(4.3, 8.1) months	46 cases<br>8.5(6.5, 11.7) months	/	**Very low**<br>⊕○○○

Table 5 Summary of evidence for B-cell depletion after 1 or 4 doses of RTX treatment in SSNS aged 1-18 years based on RCTs, NRCTs, cohort studies and case series reports

Outcome	Study Type(N)	n/N	Proportion (95%CI)	Quality of evidence (GRADE)	Comments
B-cell depletion (with a follow-up of ≥ 12 months)					
**Population: FRNS/SDNS+; Intervention: RTX (375mg/m², maximum 500mg); 1 case series report[26], 1 cohort study[36]; 38 cases; B lymphocytes were monitored at baseline, 2-7 days, 1, 3, 6, 9 and 12 months after RTX treatment[26], B lymphocytes were monitored at baseline, 1-week, every 1-3 months after RTX treatment[36].**					
1 week after RTX treatment	1 case series report[26]<br>1 cohort study[36]	24/28[26]<br>9/10[36]	88%(95%CI: 74%-95%)	**low**<br>⊕⊕○○	**Downgrade reasons:**<br>-Risk of bias ↓ 2 levels<br>**Not downgrade:**<br>-Inconsistency<br>-Indirectness of evidence<br>*[detection time of B cells]*

					**Not applicable:**<br>-Imprecision<br>-Publication of bias<br>**No upgrade reasons**
**Population: FRNS/SDNS；Intervention: RTX (375mg/m2，maximum 500mg); 1 RCT[13], 2 case series report[26,47]; 59 cases; B lymphocytes were monitored at baseline, 2-7 days, 1, 3, 6, 9 and 12 months after RTX treatment[26], B lymphocytes were monitored at baseline, every month after RTX treatment[13], B lymphocytes were monitored at baseline, monthly for the first six months, and every three months thereafter after RTX treatment[47].**					
1 month after RTX treatment	1 RCT[13]<br>2 case series report [26,47]	15/15[13]<br>28/28[26]<br>16/16[47]	100%	**low**<br>⊕⊕○○	**Downgrade reasons:**<br>-Risk of bias ↓ 2 levels<br>**Not downgrade:**<br>-Inconsistency<br>-Indirectness of evidence<br>*[detection time of B cells]*<br>**Not applicable:**<br>-Imprecision<br>-Publication of bias<br>**No upgrade reasons**
**Population: FRNS/SDNS-; Intervention: RTX (375mg/m2，maximum 500mg); 1 RCT[15]; 30 cases; B lymphocytes were monitored at baseline, every month after RTX treatment[15]**					
1 month after RTX treatment	1 RCT[15]	15/15	100%	**Moderate**<br>⊕⊕⊕○	**Downgrade reasons:**<br>-Risk of bias ↓ 1 level<br>**Not downgrade:**<br>-Inconsistency<br>-Indirectness of evidence<br>-Imprecision<br>**Not applicable:**<br>-Publication of bias<br>**No upgrade reasons**
**Population: FRNS/SDNS; Intervention: RTX(375mg/m^2 qw×4w); 1 RCT[12], 2 case series reports[48,49]; 43 cases; B lymphocytes were monitored at baseline, weekly for the first month, and monthly thereafter after RTX treatment[12,48,49].**					
1 week after first dose of RTX treatment [46]	1 RCT[12]<br>2 case series reports[48,49]	24/24[12]<br>15/15[48]<br>4/4[49]	100%	**Very low**<br>⊕○○○	**Downgrade reasons:**<br>-Risk of bias ↓ 2 levels<br>-Inconsistency ↓ 2 levels<br>*[different populations and study types]*

1 month after first dose of RTX treatment [12，45]					**Not downgrade:**<br>-Indirectness of evidence<br>-Imprecision<br>**Not applicable:**<br>-Publication of bias<br>**No upgrade reasons**

Table 6 Summary of evidence for B-cell reconstitution after 1 or 4 doses of RTX treatment (375mg/m2，maximum 500mg) in SSNS aged 1-18 years based on RCTs, NRCTs, and cohort studies

**Outcome No. of participants Study Type(N)**	**Relative effect (95% CI)**	**Anticipated absolute effects (n/N)**			**Quality of evidence(GRADE)**	**Comments**
		**Comparison Group**	**Experimental Group**	**Difference**		
**B-cell reconstitution: peripheral CD19+/CD20+ B cell count > 5 cells•μL-1 or > 1% of total lymphocytes (with a follow-up of ≥ 12 months)**						
**Population: FRNS/SDNS；Intervention: RTX (375mg/m2，maximum 500mg); 1 RCT[13], 2 case series reports[25,38], 1 cohort study[44], 1NRCT[43]; 229 cases; B lymphocytes were monitored before RTX, one week after RTX treatment, and monthly thereafter[25,43]. B lymphocytes were monitored at baseline and monthly after RTX treatment[13,38], B lymphocytes were monitored at baseline, every 1-2 months after RTX treatment[44].**						
The median time to B-cell reconstitution	5.5(95%CI:5.1-5.9) months	/	5.5 (range 4~12) months[13]<br>5 (range 1.4~20.5) months [25]<br>5.3 (range 3~10.4) months [38]<br>5 (range 3~7) months [43] (+CsA)<br>5 (range 3~17) months [43] (+MMF)<br>5.8 (range 4.8~7.3) months [44]	/	**Very low**<br>⊕○○○	**Downgrade reasons:**<br>-Risk of bias ↓ 2 levels<br>-Inconsistency ↓ 3 levels<br>*[different populations, study types and detection times]*<br>**Not downgrade:**<br>-Indirectness of evidence<br>-Imprecision<br>*[clinical experience 5-6 months]*<br>**Not applicable:**<br>-Publication of bias<br>**No upgrade reasons**
**Population: FRNS/SDNS；Intervention: RTX (375mg/m² qw×4w); 2 RCT[12,41]; 102 cases; B lymphocytes were monitored at baseline, weekly for the first month, and monthly thereafter after RTX treatment[12], B lymphocytes were monitored at baseline, monthly for the first five months, and every two months thereafter after RTX treatment[41].**						

The median time to B-cell reconstitution	5.0 f(95%CI: 4.5-5.7) months	/	4.9 (95% CI: 4.4-5.7) months[12]<br>5.5 (95% CI: 3.7-7.2) months[41] (+placebo)<br>5.4 (95% CI: 1.8-6.5)月[41] (+MMF)	/	**Very low** ⊕○○○	**Downgrade reasons:**<br>-Risk of bias ↓ 2 levels<br>-Inconsistency ↓ 3 levels<br>*[different populations, study types and detection times]*<br>**Not downgrade:**<br>-Indirectness of evidence<br>-Imprecision<br>*[clinical experience 5-6 months]*<br>**Not applicable:**<br>-Publication of bias<br>**No upgrade reasons**
**B-cell reconstitution: peripheral CD19+/CD20+ B cell count > 15 cells•μL-1 or > 3% of total lymphocytes (with a follow-up of ≥ 12 months)**						
**Population: FRNS/SDNS；Intervention: RTX (375mg/m2 qw×4w); 1 RCT[15], 1 NRCT[42], 1 case series report[52]; 41 cases; B lymphocytes were monitored at baseline and monthly after RTX treatment[15，42，52].**						
The time to B-cell reconstitution	5.0 (95%CI: 4.4-5.6) months	/	5.0±1.1 months[42](+MMF)<br>4.4±2.4 months[42]<br>Median 5.8 (range 4~12) months[15]<br>Median 4.9 (range 2.8~8.2) months[52]	/	**Very low** ⊕○○○	**Downgrade reasons:**<br>-Risk of bias ↓ 2 levels<br>-Inconsistency ↓ 2 levels<br>*[different populations and study types]*<br>**Not downgrade:**<br>-Indirectness of evidence<br>-Imprecision<br>**Not applicable:**<br>-Publication of bias<br>**No upgrade reasons**
**Population: FRNS/SDNS+; Intervention: RTX (375mg/m2，maximum 500mg); 1 case series report[47]; 16 cases; B lymphocytes were monitored at baseline, monthly for the first six months, and every three months thereafter after RTX treatment[47].**						
The mean time to B-cell reconstitution	/	/	4.4 months	/	**Very low** ⊕○○○	/
**B-cell reconstitution: peripheral CD19+/CD20+ B cell count > 10 cells•μL-1 (with a follow-up of ≥ 12 months)**						
**Population: FRNS/SDNS+; Intervention: RTX (375mg/m2，maximum 500mg); 1 case series report[29]; 35 cases; B lymphocytes were monitored at baseline and every month after RTX treatment[29].**						

The median time to B-cell reconstitution	/	/	5.8 (range 4~12) months	/	**Very low** ⊕○○○	/

Table 7 Summary of evidence for B-cell reconstitution after multiple courses of RTX treatment in SSNS aged 1-18 years based on cohort studies and case series reports

**Outcome No. of participants Study Type(N)**	**Relative effect (95% CI)**	**Anticipated absolute effects (n/N)**			**Quality of evidence (GRADE)**	**Comments**
		**Comparison Group**	**Experimental Group**	**Difference**		
**B-cell reconstitution: peripheral CD19+/CD20+ B cell count > 5 cells•μL-1 or > 1% of total lymphocytes (with a follow-up of ≥ 12 months)**						
**Population: FRNS/SDNS+; Intervention: RTX + 1 repeated course of RTX (when CD19+ B cell count >1%); Control: RTX; 1 cohort study[44]; 61 cases (16 cases in the experimental goup, 45 cases in the comparison goup)**						
The median time to B-cell reconstitution	/	5.8 (range 4.8–7.3) months	8.0 (range 5.7–9.2) months	/	**Moderate** ⊕⊕⊕○	**Downgrade reasons:**<br>-Risk of bias ↓ 1 level<br>**Not downgrade:**<br>-Indirectness of evidence<br>-Inconsistency<br>**Not applicable:**<br>-Imprecision<br>-Publication of bias<br>**No upgrade reasons**
**Population: FRNS/SDNS+; Intervention: RTX (375mg/m^2, 1~4 doses or 750mg/m^2, 1~2 doses) + 1 repeated course of RTX (375mg/m^2, 1~4 doses or 750mg/m^2, 1~2 doses); 1 case series report[53]; 346 cases**						
The median time to B-cell reconstitution		6.0 (95% CI: 5.7~ 6.2) months<br>B-cell depletion rate after first course of RTX 98.0% (196/200)	6.0 (95% CI: 5.6~6.5) months<br>B-cell depletion rate after second course of RTX 98.4% (182/185)	/	**Very low** ⊕○○○	/
	6.1 (95% CI: 6.0-6.3) months in 622 courses (98.4%)					
**B-cell reconstitution: peripheral CD19+/CD20+ B cell count > 15 cells•μL-1 or > 3% of total lymphocytes (with a follow-up of ≥ 12 months)**						
**Population: FRNS/SDNS+; Intervention: RTX (375mg/m^2 qw×3 doses) + 1-2 repeated courses of RTX (375mg/m^2 qw×3 doses, when depleted B cells rose to 4.4%± 1.1%); 1 case series report[54]; 5 cases**						

The time to B-cell reconstitution	/	7.0±1.0 months<br>B-cell depletion rate after first course of RTX 100% (5/5)	B-cell depletion lasted >15 months in all patients	/	**Very low**<br>⊕○○○	/
B-cell reconstitution: peripheral CD19+/CD20+ B cell count > 10 cells•μL-1 (with a follow-up of ≥ 12 months)						
**Population: FRNS/SDNS+; Intervention: RTX (375mg/m², 1~4 doses) + 1-4 repeated course of RTX (when CD19+ B cell count > 10 cells•μL-1); 1 case series report[48]; 22 cases**						
The mean time to B-cell reconstitution	/	7.9 (range 3.0~15.3) months<br>B-cell depletion rate after first course of RTX 100% (22/22)	B-cell depletion lasted >15 months in 73.7% (14/19) patients received 1-4 courses of RTX again after B-cell reconstitution	/	**Very low**<br>⊕○○○	/
**Population: FRNS/SDNS+; Intervention: RTX (375mg/m², every 3 months, 4 doses); 1 case series report[55]; 5 cases**						
The median time to B-cell reconstitution	/	B-cell depletion rate after first course of RTX 80% (4/5)	B-cell depletion lasted >21 months in 80% (4/5) patients	/	**Very low**<br>⊕○○○	/

Table 8 Summary of evidence for relapse during B cell depletion after the fisrt course of RTX treatment in SSNS aged 1-18 years based on RCTs, NRCTs, cohort studies and case series reports

Outcome	Study Type(N)	n/N	Proportion (95%CI)	Quality of evidence (GRADE)	Comments
Relapse during B cell depletion (with a follow-up of ≥ 12 months)					
**Population: FRNS/SDNS; Intervention: RTX (375mg/m2 qw×1-4 doses); 4 RCTs[14,15,16,17], 3 case series reports[27,38,50], 1 cohort study[36], 1 NRCT[43]; 280 cases**					
Relapse rate	4 RCTs[14,15,16,17]<br>3 case series report[27,38,50]<br>1 cohort study[36]<br>1 NRCT[43]	0/60[14]<br>0/15[15]<br>0/20[16]<br>4/35[17]<br>0/16[27]<br>0/9[36]<br>7/78[38]<br>0/29[43]<br>0/18[50]	1% (95%CI: 0～18%)	**Very low**<br>⊕○○○	**Downgrade reasons:**<br>-Risk of bias ↓ 2 levels<br>-Inconsistency ↓ 3 levels<br>*[different populations, research types and dosages of intervention]*<br>**Not downgrade:**<br>-Indirectness of evidence<br>-Imprecision<br>*[clinical experience 11%]*<br>**Not applicable:**<br>-Publication of bias<br>**No upgrade reasons**

Table 9 Summary of evidence for detection of B cell subgroups and related immunological molecules after the fisrt course of RTX treatment in SSNS aged 1-18 years based on cohort studies

Outcome<br>No. of participants<br>Study Type(N)	Relative effect<br>(95% CI)	Anticipated absolute effects (n/N)			Quality of evidence(GRADE)	Comments
		Comparison Group	Experimental Group	Difference		
**Detection of peripheral memory B cells with a follow-up of 12 months after RTX treatment**						
**Population: FRNS/SDNS; Intervention: RTX (375mg/m2 qw×1-2 doses); 3 cohort studies[26,27,51]; 74 cases (39 cases in the non-relapsed group, 35 cases in the relapsed group); Detection at baseline and every 1-2 months after RTX treatment[27,51], Detection at baseline, 2-7 days, 1, 3, 6, 9 and 12 months after RTX treatment[26]**						
Baseline	no statistically significant difference	2.6 (IQR: 1.6, 3.8)%[26]<br>98 (IQR: 60, 157)/μL[27]<br>94.68±25.55/μL[51]	2.9 (IQR: 0.9, 4.4)%[26]<br>98 (IQR: 60, 157)/μL[27]<br>84.0±19.64/μL[51]	/	**Very low**<br>⊕○○○	**Downgrade reasons:**<br>-Risk of bias ↓ 2 levels<br>-Inconsistency ↓ 3 levels<br>*[different populations, study types and detection times of B cells]*<br>**Not downgrade:**<br>-Indirectness of evidence<br>**Not applicable:**<br>-Imprecision<br>-Publication of bias<br>**No upgrade reasons**
12 months after RTX or the relapse time	*p*<0.001<br>*p*<0.05<br>*p*=0.15	(0.6±0.18)%[26]<br>30 (IQR: 25, 41)/μL[27]<br>40.69±16.72/μL[51]	(0.32±0.08)%[26]<br>18 (IQR: 8, 27)/μL[27]<br>12.67±4.51/μL[51]	/	**Very low**<br>⊕○○○	
**Detection of peripheral Th17 cells with a follow-up of 12 months after RTX treatment**						
**Population: FRNS/SDNS; Intervention: RTX (375mg/m2 qw×2 doses); 2 cohort studies[27,51]; 46 cases (25 cases in the non-relapsed group, 21 cases in the relapsed group); Detection at baseline and every 1-2 months after RTX treatment[27,51]**						
Baseline	no statistically significant difference	11 (IQR: 7, 17)/μL[27]<br>13.09±4.22/μL[51]	11 (IQR: 7, 17)/μL[27]<br>10.13±2.89/μL[51]	/	**Very low**<br>⊕○○○	**Downgrade reasons:**<br>-Risk of bias ↓ 2 levels<br>-Inconsistency ↓ 1 level<br>*[different populations]*<br>**Not downgrade:**<br>-Indirectness of evidence<br>**Not applicable:**<br>-Imprecision<br>-Publication of bias
12 months after RTX or the relapse time	*p*=0.018<br>*p*<0.05	12 (IQR: 7, 23)/μL[27]<br>12.08±5.16/μL[51]	6 (IQR: 4, 10)/μL[27]<br>6.86±2.03/μLl[51]	/	**Very low**<br>⊕○○○	

						**No upgrade reasons**
**Detection of peripheral IgM memory B cells with a follow-up of 12 months after RTX treatment**						
**Population: FRNS/SDNS; Intervention: RTX (375mg/m2 qw×1-2 doses); 1 cohort study[26]; 28 cases (14 cases in the non-relapsed group, 14 cases in the relapsed group); Detection at baseline, 2-7 days, 1, 3, 6, 9 and 12 months after RTX treatment[26]**						
Baseline	no statistically significant difference	1.17(IQR: 0.76, 3.04)%	1.37 (IQR:0.42 , 2.55)%	/	**Very low** ⊕○○○	**Downgrade reasons:**<br>-Risk of bias ↓ 1 levels<br>-Inconsistency ↓ 1 level<br>*[different populations]*<br>**Not downgrade:**<br>-Indirectness of evidence<br>**Not applicable:**<br>-Imprecision<br>-Publication of bias<br>**No upgrade reasons**
12 months after RTX or the relapse time	*P*=0.04	(0.32±0.05)%	(0.16±0.04)%	/	**Very low** ⊕○○○	
**Detection of peripheral switched memory B cells with a follow-up of 12 months after RTX treatment**						
**Population: FRNS/SDNS; Intervention: RTX (375mg/m2 qw×1-2 doses); 1 cohort study[26]; 28 cases (14 cases in the non-relapsed group, 14 cases in the relapsed group); Detection at baseline, 2-7 days, 1, 3, 6, 9 and 12 months after RTX treatment[26]**						
Baseline	no statistically significant difference	1.31(IQR: 0.62, 3.14)%	0.98 (IQR:0.29 , 1.6)%	/	**low** ⊕⊕○○	**Downgrade reasons:**<br>-Risk of bias ↓ 1 level<br>-Inconsistency ↓ 1 level<br>*[different populations]*<br>**Not downgrade:**<br>-Indirectness of evidence<br>**Not applicable:**<br>-Imprecision<br>-Publication of bias<br>**No upgrade reasons**
12 months after RTX or the relapse time	*P*=0.01	(0.38±0.1)%	(0.08±0.01)%	/		

Table 10 Summary of evidence for adverse events of RTX treatment in SSNS aged 1-18 years based on RCTs and NRCTs

Outcome	Relative effect (95% CI)	Anticipated absolute effects (n/N)			Quality of evidence(GRADE)	Comments
		Comparison Group	Experimental Group	Difference		
**Adverse events**						
**Population: FRNS/SDNS; Intervention: RTX 375mg/m2, 4 doses; Comparison: placebo; 1 RCT[12]; 48 cases (24 cases in the experimental goup, 24 cases in the comparison goup)**						
The incidence of adverse events	OR=3.13(0.12-80.68)	23/24[12]	24/24[12]	/	**Very low** ⊕○○○	**Downgrade reasons:**<br>-Risk of bias ↓ 2 levels<br>-Imprecision ↓ 2 levels<br>*[clinical experience 30%~40%]*<br>**Not downgrade:**<br>-Indirectness of evidence<br>-Inconsistency<br>**Not applicable:**<br>-Publication of bias<br>**No upgrade reasons**
**Population: FRNS/SDNS; Intervention: RTX 375mg/m2, 1-4 doses; Comparison: steroid [13]/CNI[14,16,17,20]/CTX[19]; 4 RCTs[13,14,16,17], 2 NRCTs[19,20]; 343 cases (175 cases in the experimental goup, 168 cases in the comparison goup)**						
The incidence of adverse events	OR=3.77(0.26-55.52)	84/168(combined)<br>0/15[13]<br>47/60[14]<br>20/20[16]<br>10/18[17]<br>0/15[18]<br>6/27[19]<br>1/13[20]	106/175(combined)<br>15/15[13]<br>41/60[14]<br>20/20[16]<br>26/36[17]<br>0/15[18]<br>1/19[19]<br>3/10[20]	272 more per 1,000 (from 309 fewer to 436 more)	**Very low** ⊕○○○	**Downgrade reasons:**<br>-Risk of bias ↓ 2 levels<br>-Inconsistency ↓ 2 levels<br>*[different populations, study types and controls]*<br>-Imprecision ↓ 2 levels<br>*[clinical experience 30%~40%]*<br>**Not downgrade:**<br>-Indirectness of evidence<br>**Not applicable:**<br>-Publication of bias<br>**No upgrade reasons**
**Population: FRNS/SDNS; Intervention: RTX 375mg/m2, 1-4 doses; Comparison: placebo[12]/steroid [13]/CNI[14,16,17,20]/CTX[19]; 5 RCTs[12,13,14,16,17], 2 NRCTs[19,20]; 391 cases (199 cases in the experimental goup, 192 cases in the comparison goup)**						

The incidence of adverse events	OR=4.96(0.36-68.46)	107/192(combined)<br>23/24[12]<br>0/15[13]<br>47/60[14]<br>20/20[16]<br>10/18[17]<br>0/15[18]<br>6/27[19]<br>1/13[20]	130/199(combined)<br>24/24[12]<br>15/15[13]<br>41/60[14]<br>20/20[16]<br>26/36[17]<br>0/15[18]<br>1/19[19]<br>3/10[20]	279 more per 1,000 (from 250 fewer to 386 more)	**Very low**<br>⊕○○○	**Downgrade reasons:**<br>-Risk of bias ↓ 2 levels<br>-Inconsistency ↓ 2 levels<br>*[different populations, study types, dosages of intervention and controls]*<br>-Imprecision ↓ 2 levels<br>*[clinical experience 30%~40%]*<br>**Not downgrade:**<br>-Indirectness of evidence<br>**Not applicable:**<br>-Publication of bias<br>**No upgrade reasons**
**Serious adverse events**						
**Population: FRNS/SDNS; Intervention: RTX 375mg/m2, 4 doses; Comparison: placebo; 1 RCT[12]; 48 cases (24 cases in the experimental goup, 24 cases in the comparison goup)**						
The incidence of serious adverse events	OR=2.14(0.63-7.33)	6/24[12]	10/24[12]	/	**low**<br>⊕⊕○○	**Downgrade reasons:**<br>-Risk of bias ↓ 1 level<br>-Imprecision ↓ 1 level<br>*[clinical experience 2%]*<br>**Not downgrade:**<br>-Indirectness of evidence<br>-Inconsistency<br>**Not applicable:**<br>-Publication of bias<br>**No upgrade reasons**
**Population: FRNS/SDNS; Intervention: RTX 375mg/m2, 1-4 doses; Comparison: CNI; 3 RCTs[14,16,17]; 214 cases (116 cases in the experimental goup, 98 cases in the comparison goup)**						
The incidence of serious adverse events	OR=0.90(0.18-4.35)	3/98(combined)<br>0/60[14]<br>2/20[16]<br>1/18[17]	4/116(combined)<br>0/60[14]<br>1/20[16]<br>3/36[17]	3 fewer per 1,000(from 25 fewer to 90 more)	**Very low**<br>⊕○○○	**Downgrade reasons:**<br>-Risk of bias ↓ 2 levels<br>-Inconsistency ↓ 1 levels<br>*[different populations]*<br>-Imprecision ↓ 2 levels<br>*[clinical experience 2%]*<br>**Not downgrade:**

						-Indirectness of evidence<br>**Not applicable:**<br>-Publication of bias<br>**No upgrade reasons**
**Population: FRNS/SDNS; Intervention: RTX 375mg/m2, 1-4 doses; Comparison: placebo[12]/CNI[14,16,17]; 4 RCTs[12,14,16,17]; 262 cases (140 cases in the experimental goup, 122 cases in the comparison goup)**						
The incidence of serious adverse events	OR=1.56(0.59-4.17)	9/122(combined)<br>6/24[12]<br>0/60[14]<br>2/20[16]<br>1/18[17]	14/140(combined)<br>10/24[12]<br>0/60[14]<br>1/20[16]<br>3/36[17]	37 more per 1,000 (from 29 fewer to 176 more)	**Very low**<br>⊕○○○	**Downgrade reasons:**<br>-Risk of bias ↓ 2 levels<br>-Inconsistency ↓ 1 levels<br>*[different populations]*<br>-Imprecision ↓ 2 levels<br>*[clinical experience 2%]*<br>**Not downgrade:**<br>-Indirectness of evidence<br>**Not applicable:**<br>-Publication of bias<br>**No upgrade reasons**
**Neutropenia**						
**Population: FRNS/SDNS; Intervention: RTX 375mg/m2, 4 doses; Comparison: placebo; 1 RCT[12]; 48 cases (24 cases in the experimental goup, 24 cases in the comparison goup)**						
The incidence of neutropenia	OR=8.47(1.12-64.20)	0/24[12]	4/24[12]	/	**Very low**<br>⊕○○○	**Downgrade reasons:**<br>-Risk of bias ↓ 1 level<br>-Imprecision ↓ 2 levels<br>*[clinical experience 30%~40%]*<br>**Not downgrade:**<br>-Indirectness of evidence<br>**Not applicable:**<br>-Inconsistency<br>-Publication of bias<br>**No upgrade reasons**
**Population: FRNS/SDNS; Intervention: RTX 375mg/m2, 2-4 doses; Comparison: FK; 1 RCT[16]; 40 cases (20 cases in the experimental goup, 20 cases in the comparison goup)**						
The incidence of neutropenia	OR=7.39(0.15-372.38)	0/20[16]	1/20[16]	/	**Very low**<br>⊕○○○	**Downgrade reasons:**<br>-Risk of bias ↓ 1 level

						-Imprecision ↓ 2 levels<br>*[clinical experience 30%~40%]*<br>**Not downgrade:**<br>-Indirectness of evidence<br>**Not applicable:**<br>-Inconsistency<br>-Publication of bias<br>**No upgrade reasons**
**lymphopenia**						
**Population: FRNS/SDNS; Intervention: RTX 375mg/m2, 4 doses; Comparison: placebo; 1 RCT[12]; 48 cases (24 cases in the experimental goup, 24 cases in the comparison goup)**						
The incidence of lymphopenia	OR=1.00(0.22-4.49)	4/24[12]	4/24[12]	/	**Very low**<br>⨁○○○	**Downgrade reasons:**<br>-Risk of bias ↓ 1 level<br>-Inconsistency ↓ 1 level<br>*[different populations]*<br>-Imprecision ↓ 1 level<br>*[clinical experience 10%]*<br>**Not downgrade:**<br>-Indirectness of evidence<br>**Not applicable:**<br>-Publication of bias<br>**No upgrade reasons**
**Population: FRNS/SDNS; Intervention: RTX 375mg/m2, 2-4 doses; Comparison: FK; 1 RCT[16]; 40 cases (20 cases in the experimental goup, 20 cases in the comparison goup)**						
The incidence of lymphopenia	OR=0.14(0.00-6.82)	1/20[16]	0/20[16]	/	**Very low**<br>⨁○○○	**Downgrade reasons:**<br>-Risk of bias ↓ 1 level<br>-Inconsistency ↓ 1 level<br>*[different populations]*<br>-Imprecision ↓ 2 levels<br>*[clinical experience 20%]*<br>**Not downgrade:**<br>-Indirectness of evidence<br>**Not applicable:**<br>-Publication of bias

						**No upgrade reasons**
Infections						
**Population: FRNS/SDNS; Intervention: RTX 375mg/m2, 4 doses; Comparison: placebo; 1 RCT[12]; 48 cases (24 cases in the experimental goup, 24 cases in the comparison goup)**						
The incidence of infections	OR=7.67(0.85-69.54)	18/24[12]	23/24[12]	/	**Very low**<br>⊕○○○	**Downgrade reasons:**<br>-Risk of bias ↓ 1 level<br>-Inconsistency ↓ 1 level<br>*[different populations]*<br>-Imprecision ↓ 2 levels<br>*[clinical experience 20%]*<br>**Not downgrade:**<br>-Indirectness of evidence<br>**Not applicable:**<br>-Publication of bias<br>**No upgrade reasons**
**Population: FRNS/SDNS; Intervention: RTX 375mg/m2, 1-4 doses; Comparison: CNI; 2 RCTs[16,17]; 94 cases (56 cases in the experimental goup, 38 cases in the comparison goup)**						
The incidence of infections	OR=2.83(0.69-11.62)	23/38(combined)<br>20/20[16]<br>3/18[17]	33/56(combined)<br>20/20[16]<br>13/36[17]	207 more per 1,000 (from 91 fewer to 342 more)	**Very low**<br>⊕○○○	**Downgrade reasons:**<br>-Risk of bias ↓ 1 level<br>-Inconsistency ↓ 1 level<br>*[different populations]*<br>-Imprecision ↓ 2 levels<br>*[clinical experience 20%]*<br>**Not downgrade:**<br>-Indirectness of evidence<br>**Not applicable:**<br>-Publication of bias<br>**No upgrade reasons**

Table 11 Summary of evidence for adverse events of RTX treatment in SSNS aged 1-18 years based on case series reports

Outcome	Study Type(N)	n/N	Proportion(95%CI)	Quality of evidence
**Infusion-related reactions**	21 case series reports [7, 11, 24, 28, 36, 43, 46, 47, 48, 50, 52, 54, 55, 57, 59, 64, 67, 68, 69, 70, 71]	269/856(combined)<br>0/12[7]<br>2/24[11]<br>2/37[24]<br>3/7[28]<br>5/10[36]<br>13/29[43]<br>1/9[46]<br>3/16[47]<br>2/22[48]<br>10/18[50]<br>5/12[52]<br>0/5[54]<br>1/5[55]<br>5/11[57]<br>7/70[59]<br>8/28[64]<br>6/30[67]<br>183/445[68]<br>2/19[69]<br>1/21[70]<br>10/26[71]	31%(28%~35%)	**Very low**<br>⊕○○○
**neutropenia**	11 case series reports [11, 31, 34, 43, 56, 57, 58, 59, 60, 61, 62]	19/369(combined)<br>2/24[11]<br>3/46[31]<br>4/51[34]<br>0/29[43]<br>1/11[57]<br>1/18[56]<br>2/58[58]<br>2/70[59]	5%(3%~8%)	**Very low**<br>⊕○○○

		3/22[60]<br>1/20[61]<br>0/20[62]		
**hypogammaglobulinemia**	11 case series reports [9, 11, 34, 36, 43, 46, 54, 61, 62, 63, 64]	104/393(combined)<br>45/90[9]<br>1/24[11]<br>0/51[34]<br>0/10[36]<br>0/29[43]<br>2/9[46]<br>0/5[54]<br>7/20[61]<br>11/20[62]<br>37/107[63]<br>1/28[64]	26%(22%~31%)	**Very low**<br>⊕○○○
Target study subgroup	2 case series reports [9, 62]	56/110(combined)<br>45/90[9]<br>11/20[62]	51%(42%~60%)	**Very low**<br>⊕○○○
Non-target study subgroup	9 case series reports [11, 34, 36, 43, 46, 54, 61, 63, 64]	48/283(combined)<br>1/24[11]<br>0/51[34]<br>0/10[36]<br>0/29[43]<br>2/9[46]<br>0/5[54]<br>7/20[61]<br>37/107[63]<br>1/28[64]	17%(13%～22%)	**Very low**<br>⊕○○○
**persistent hypogammaglobulinemia**	3 case series reports [9, 63, 65]	34/257(combined)<br>11/90[9]<br>2/60[63]<br>21/107[65]	13%(10%~18%)	**Very low**<br>⊕○○○
**anti-RTX antibody (ARA) detection**	5 case series reports [10, 11, 50, 60, 66]	15/96(combined)	16%(10%~24%)	**Very low**<br>⊕○○○

		2/19[10]<br>7/24[11]<br>1/18[50]<br>0/22[60]<br>5/13[66]		
**serum sickness**	3 case series reports [31, 59, 66]	6/129(combined)<br>3/46[31]<br>2/70[59]<br>1/13[66]	5%(2%~10%)	**Very low**<br>⊕○○○

Evidence-to-Decision Table of the Rituximab Guidelines

Summary Table of Recommendations

Recommendations	Strength of recommendations	Quality of evidence
1. One-year relapse rate following first course of RTX treatment in children with FRNS/SDNS is decreased by 82% compared with the control group (RTX vs. placebo, decreased by 95%; RTX vs. CTX/CNI/MMF, decreased by 68%).	Strong	Very low
2. Average 1-year relapse rate following first course of RTX treatment (375 mg·m-2, variable doses) is 41% in children with FRNS/SDNS (47% in FRNS/SDNS+, and 22% in FRNS/SDNS-).	Strong	Very low
3. There is no difference in 2-year relapse rate between first course of RTX treatment and continuous immunosuppressive therapy in children with FRNS/SDNS.	Weak	Very low
4. Median time to first relapse is about 10 months in children with FRNS/SDNS+ after first course of RTX treatment with a follow-up of ≥ 12 months.	Strong	Very low
5. Significant benefit of RTX treatment for children with FRNS/SDNS is achieved in terms of 1-year cumulative steroid dose reduction compared with other immunosuppressants (RTX vs. FK, lowered by 0.15 mg·kg-1·d-1; RTX vs. placebo, reduced by 0.26 mg·kg-1·d-1).	Strong	Very low
6. RTX treatment benefits on the time to steroid withdrawal in children with FRNS/SDNS compared with other immunosuppressants.	Strong	Very low
7. RTX + MMF is recommended for the treatment of children with FRNS/SDNS in consideration of delayed time to first relapse and reduced steroid dose by about 0.18 mg·kg-1·d-1.	Strong	Very low
8. Re-exposure to RTX could decrease the relapse rate in children with FRNS/SDNS after RTX treatment.	Strong	Very low
9. RTX treatment improves the quality of life in children with FRNS/SDNS.	Strong	Very low
10. Re-exposure to RTX in relapsed children with FRNS/SDNS after RTX treatment improves the relapse-free survival rate and prolongs the relapse-free survival.	Strong	Very low
11. In children with FRNS/SDNS receiving 1~4 doses of RTX treatment, B-cell depletion is achieved at one month after the first dose, B-cell reconstitution occurs at 5~6 months, and multiple courses of RTX treatment slightly prolongs the duration of B-cell depletion.	Strong	Very low

12. The relapse rate during B-cell depletion is 1%.	Strong	Very low
13. RTX treatment does not increase the incidence of serious adverse events and infections in children with SSNS.	Strong	Very low
14. RTX treatment of children with SSNS does not increase the total medical expenditure.	Strong	Very low
**Detailed judgments: A total of 18 original recommendations were discussed at the EtD meeting. The meeting decided to merge recommendation 7 and recommendation 9, delete 3 recommendations, and retain 14 recommendations. After the review comments were returned, it was revised to 7 recommended comments.**		

Recommendation 1: One-year relapse rate following first course of RTX treatment in children with FRNS/SDNS is decreased by 82% compared with the control group (RTX vs. placebo, decreased by 95%; RTX vs. CTX/CNI/MMF, decreased by 68%). (1D)

Does RTX improve clinical outcomes in children aged 1-18 years with SSNS compared with other immunosuppressants or blank/placebo control?	
STUDY TYPE	Two-arm study
POPULATION	FRNS/SDNS
INTERVENTION	First course of RTX treatment: 1~4 doses, with a single dose of 375 mg·m^{-2}, once every 1~2 weeks
COMPARISON	Blank, Placebo, CTX, CNI
MAIN OUTCOMES	12-month relapse rate of RTX treatment
PERSPECTIVE	Clinicians, social workers and parents of children with FRNS/SDNS
SETTING	Hospital
CONFLICT OF INTERESTS	No

ASSESSMENT

1. PROBLEM: Is the problem a priority?<br>Detailed judgments: The more serious or urgent a problem is, the more likely it is that an option that addresses the problem will be a priority.<br>RTX 治疗 FRNS/SDNS 患儿随访 12 月的复发率具有优先性吗?		
JUDGEMENTS	RESEARCH EVIDENCE	ADDITIONAL CONSIDERATIONS
☐ No<br>☐ Probably no<br>☐ Probably yes<br>☑ **Yes**<br>☐ Varies<br>☐ Uncertain	/	Vote Results: 4/14 chose "Probably yes", **12/14 chose "Yes"**, 1/14 chose "Uncertain".

2. Desirable effects: How substantial are the desirable anticipated effects?

Detailed judgments: How large are the desirable effects of the intervention taking into account the importance of the outcomes (how much they are valued), and the size of the effect (the likelihood of experiencing a benefit or how much of an improvement individuals would be likely to experience)?

RTX 治疗 FRNS/SDNS 患儿随访 12 月的较 CTX、CNI、安慰剂或单独使用激素 1 年复发率降低了 80%左右的有益影响有多大？根据随访 12 月的复发率结局重要性和效应值大小（获益的可能性和个体情况改善的程度）对干预措施的有益影响进行判断。

JUDGEMENTS

- ☐ Trivial
- ☐ Small
- ☐ Moderate
- ☑ **Large**
- ☐ Varies
- ☐ Uncertain

RESEARCH EVIDENCE

Outcome<br>No. of participants<br>Study Type(N)	Relative effect<br>(95% CI)	Anticipated absolute effects (n/N)			Quality of evidence<br>(GRADE)	Comments
		Comparison Group	Experimental Group	Difference		
复发人数/总人数						
**人群：FRNS/SDNS-（SDNS [12，13，14，15，16]，合并或不合并左旋咪唑[19]，SDNS 合并 CNI 依赖至少 2 年[17]. SDNS 大剂量激素依赖，或既往应用左旋咪唑，CTX 和/或 MMF. 后接受利妥昔单抗或 CNI 者[20]，部分利妥昔单抗合并或不合并 CNI，部分单独 CNI[21]）；干预组: 利妥昔单抗；对照组：CNI 或 CTX 或单独激素或安慰剂；7 项 RCT[12，13，14，15，16，17，18]，2 篇队列研究[20，21]和 1 项 NRSI[19] Meta 分析，432 例**						
随访 12 月	**干预/对照**<br>**FRNS/SDNS**<br>（77/220；133/212）：<br>OR =0.18，95%CI:0.07~0.44<br>**FRNS/SDNS+**<br>（52/92；63/75）：<br>OR =0.19，95%CI:0.05~0.68<br>**FRNS/SDNS-**<br>（25/128；70/137）：<br>OR =0.15, 95%CI:0.03~0.68<br>**RTX 较安慰剂或空白对照:**<br>（46/86；66/69）<br>OR =0.05，95%CI:0.01~0.18<br>**RTX 较 CTX、CNI 或 MMF:**<br>（31/134；67/143）<br>OR =0.32，95%CI:0.13~0.82	23/24 人[12]<br>15/15 人[13]<br>22/60 人[14]<br>14/15 人[15]<br>9/20 人[16]<br>14/15 人[17] Total 16<br>15/15 人[18]<br>10/27 人[19]<br>7/13 人[20]<br>6/8 人[21]	17/24 人[12]<br>5/14 人[13] Total 15<br>6/60 人[14]<br>2/15 人[15]<br>9/20 人[16]<br>22/33 人[17] Total 35<br>2/15 人[18]<br>3/19 人[19]<br>5/10 人[20]<br>6/10 人[21]	**FRNS/SDNS:**<br>395 fewer per 1,000<br>(from 522 fewer to 202 fewer)<br>**FRNS/SDNS+:**<br>341 fewer per 1,000<br>(from 632 fewer to 59 fewer)<br>**FRNS/SDNS-:**<br>375 fewer per 1,000<br>(from 481 fewer to 96 fewer)<br>**RTX 较安慰剂或空白对照:**<br>433 fewer per 1,000<br>(from 776 fewer to 158 fewer)<br>**RTX 较 CTX、CNI 或 MMF:**<br>248 fewer per 1,000<br>(from 366 fewer to 49 fewer)	**Very low**<br>⊕○○○	**Downgrade reasons:**<br>-Risk of bias ↓2 levels<br>-Inconsistency ↓2 levels<br>*[different population, research type; 干预组剂次不同、对照组不同干预]*<br>-Imprecision ↓2 levels<br>*[临床经验 60~80%]*<br>**Not downgrade:**<br>- Publication of bias<br>- Indirectness of evidence<br>**No upgrade reasons**

ADDITIONAL CONSIDERATIONS

Vote Results: 1/14 chose "Small", **13/14 chose "Large"**.

3. Undesirable effects: How substantial are the undesirable anticipated effects?

Detailed judgments: How large are the desirable effects of the intervention taking into account the importance of the outcomes (how much they are valued), and the size of the effect (the likelihood of experiencing a benefit or how much of an improvement individuals would be likely to experience)?

RTX 治疗 FRNS/SDNS 患儿随访 12 月较 CTX、CNI、安慰剂或单独使用激素 1 年复发率降低了 80%左右的不良影响有多大？根据随访 12 月的复发率和效应值大小（获益的可能性和个体情况改善的程度）对干预措施的不良影响进行判断

JUDGEMENTS

- ☐ Large
- ☐ Moderate
- ☑ **Small**
- ☑ **Trivial**
- ☐ Varies
- ☐ Uncertain

RESEARCH EVIDENCE

/

ADDITIONAL CONSIDERATIONS

Vote Results: 2/14 chose "Large", **6/14 chose "Small", 6/14 chose "Trivial".**

Experts voted even, but the overall trend was "small".

4. Certainty of the evidence: What is the overall certainty of the evidence of effects?

Detailed judgments: How good an indication does the research provide of the likely effects across all of the critical outcomes; i.e. the likelihood that the effects will be different enough from what the research found that it might affect a decision about the intervention?

RTX 干预后较 CTX、CNI、安慰剂或单独使用激素 1 年复发率降低了 80%左右有益影响和不良影响相关证据的总体质量？基于 RTX 干预后较对照 1 年复发率降低了 80%左右可能影响，判断 RTX 干预效果是否会对干预决策产生影响。

JUDGEMENTS	RESEARCH EVIDENCE	ADDITIONAL CONSIDERATIONS
☑ **Very low**<br>□ Low<br>□ Moderate<br>□ High<br>□ No included studies	/	Vote Results： 2/14 chose “Very low”， 4/14 chose “Low”， 5/14 chose “Moderate”， 3/14 chose “High”．

5. Values: Is there important uncertainty about or variability in how much people value the main outcomes?

Detailed judgments: How much do individuals value each of the main outcomes? Is uncertainty about how much they value each of the outcomes or variability in how much different individual value the outcomes large enough that it could lead to different decisions?

对 RTX 治疗 FRNS/SDNS 患儿较 CTX、CNI、安慰剂或单独使用激素 1 年复发率降低了 80%左右重视程度，是否因个体不同而存在不确定性和变化性？RTX 干预后较 CTX、CNI、安慰剂或单独使用激素 1 年复发率降低了 80%左右的重视程度有多大？不确定性和变化性是否会导致不同的决策？不确定性体现在对上述推荐的理解程度；变化性体现在对上述推荐重视程度的差异。

JUDGEMENTS	RESEARCH EVIDENCE	ADDITIONAL CONSIDERATIONS
□ Important uncertainty or variability<br>□ Possibly important uncertainty or variability<br>□ Probably no important uncertainty or variability<br>☑ **No important uncertainty or variability**	/	Vote Results： 2/14 chose “Probably no Important uncertainty or variability”， **12/14 chose “No Important uncertainty or variability”**．

6. Balance of effects: Does the balance between desirable and undesirable effects favour the intervention or the comparison?

Detailed judgments: What is the balance between the desirable and undesirable effects, taking into account how much individuals value the main outcome, how substantial the desirable and undesirable effects are, the certainty of those estimates, discount rates, risk aversion and risk seeking?

权衡 RTX 较 CTX、CNI、安慰剂或单独使用激素 1 年复发率降低了 80%左右利弊后，更倾向于 RTX 还是 CTX，CNI，安慰剂，单独使用激素从以下方面权衡利弊：个体对上述推荐的重视程度；利多大？；弊多大？；估计值的精确性；信心有多大？；风险多大？；可能规避风险么？

JUDGEMENTS	RESEARCH EVIDENCE	ADDITIONAL CONSIDERATIONS

□ Favours the comparison<br>□ Probably favours the comparison<br>□ Does not favour either the intervention or the comparison<br>□ Probably favours the intervention<br>☑ **Favours the intervention**<br>□ Varies<br>□ Uncertain	/	Vote Results: 5/14 chose "Probably favours the intervention", **9/14 chose "Favours the intervention"**.

7. Resources required: How large are the resource requirements (costs)?

Detailed judgments: How large is the cost of the difference in resource use between the intervention and comparison?

RTX 干预后较 CTX、CNI、安慰剂或单独使用激素 1 年复发率降低了 80%左右成本支出有多大？RTX 较对照 1 年复发率降低了 80%左右，RTX 和对照组(CTX，CNI，安慰剂，单独使用激素)的支出成本差异有多大？

JUDGEMENTS	RESEARCH EVIDENCE	ADDITIONAL CONSIDERATIONS
□ Large costs<br>□ Moderate costs<br>☑ **Negligible costs or savings**<br>□ Moderate savings<br>□ Large savings<br>□ Varies<br>□ Uncertain	Refer to recommendation 18	Vote Results: **8/14 chose "Negligible costs or savings"**, 5/14 chose "Moderate savings", 1/14 chose "Large savings".

8. Certainty of evidence of required resources: What is the certainty of the evidence of resource requirements (costs)?

Detailed judgments: How certain is the evidence of a difference for each type of resource use (eg. drugs, hospitalizations) and the cost of resources?

成本支出的证据质量如何？RTX 较 CTX、CNI、安慰剂或单独使用激素 1 年复发率下降了 80%左右成本支出（包括药物、住院等费用）相关证据的确定性。

JUDGEMENTS	RESEARCH EVIDENCE	ADDITIONAL CONSIDERATIONS
☑ **Very low**<br>□ Low<br>□ Moderate<br>□ High<br>□ No included studies	Refer to recommendation 18	Vote Results: 3/14 chose "Very low", 3/14 chose "Low", **5/14 chose "Moderate"**, 2/14 chose "High", 1/14 chose "No included studies".<br><br>Referring to recommendation 18, the quality of evidence is very low.

9. Cost-effectiveness: Does the cost-effectiveness of the intervention favour the intervention or the comparison?

Detailed judgments: Is the intervention cost-effective, taking into account uncertainty about or variability in the costs, uncertainty about or variability in the net benefit, sensitivity analyses, and the reliability and applicability of the economic evaluation?

RTX 较 CTX、CNI、安慰剂或单独使用激素 1 年复发率下降了 80%左右的成本效益分析更倾向于 RTX 还是 CTX，CNI，安慰剂，单独使用激素从以下方面分析干预的成本效益：对支出成本的不确定性或变化性；对净利润的不确定性或变化性；敏感性分析；经济评估的可靠性和适用性。

JUDGEMENTS	RESEARCH EVIDENCE	ADDITIONAL CONSIDERATIONS
□ Favours the comparison<br>□ Probably favours the comparison<br>□ Does not favour either the intervention or the comparison<br>□ Probably favours the intervention<br>☑ **Favours the intervention**<br>□ Varies<br>□ No included studies	Refer to recommendation 18	Vote Results: 4/14 chose "Probably favours the intervention", **10/14 chose "Favours the intervention"**.

10. EQUITY: What would be the impact on health equity?

Detailed judgments: Are there plausible reasons for anticipating differences in the relative effectiveness of the intervention for disadvantaged subgroups or different baseline conditions across disadvantaged subgroups that affect the absolute effectiveness of the intervention or the importance of the problem?

RTX 较 CTX、CNI、安慰剂或单独使用激素 1 年复发率降低了 80%左右的是否在弱势人群中有所降低，对此是否有合理的解释？弱势人群的不同基线水平会影响干预的绝对有效性或研究问题的重要性，对此是否有合理的解释？

JUDGEMENTS	RESEARCH EVIDENCE	ADDITIONAL CONSIDERATIONS
□ Reduced<br>□ Probably reduced<br>☑ **Probably no impact**<br>□ Probably increased<br>□ Increased<br>□ Varies<br>□ Uncertain	/	Vote Results: 4/14 chose "Probably Reduced", **5/14 chose "Probably no impact"**, 4/14 chose "Increased", 1/14 chose "Varies".

11. ACCEPTABILITY: Is the intervention acceptable to key stakeholders?

Detailed judgments: Are key stakeholders likely not to accept the distribution of the benefits, harms and costs; or the costs or undesirable effects in the short term for desirable effects (benefits) in the future? Are they likely to disagree with the values attached to the desirable or undesirable effects, or not to accept the diagnostic intervention because of ethical concerns?

患儿及其家长是否接受 RTX 较 CTX、CNI、安慰剂或单独使用激素 1 年复发率降低了 80%左右？患儿及其家长是否会接受干预带来的获益、伤害及支出，或远期获益带来的短期内的不良反应；是否会对

利弊判断背后的价值观念持反对意见；是否会出于伦理考虑拒绝诊断性治疗。		
**JUDGEMENTS**	**RESEARCH EVIDENCE**	**ADDITIONAL CONSIDERATIONS**
☐ No<br>☐ Probably no<br>☐ Probably yes<br>☑ **Yes**<br>☐ Varies<br>☐ Uncertain	/	Vote Results： 5/14 chose “Probably Yes”， **9/14 chose “Yes”** .

12. FEASABILITY: Is the option feasible to implement?

Detailed judgments: Is it feasible to sustain use of the intervention and to address potential barriers to using it?

JUDGEMENTS	RESEARCH EVIDENCE	ADDITIONAL CONSIDERATIONS
☐ No<br>☐ Probably no<br>☐ Probably yes<br>☑ **Yes**<br>☐ Varies<br>☐ Uncertain	/	Vote Results： 1/14 chose “Probably Yes”， **13/14 chose “Yes”** .

SUMMARY OF JUDGEMENTS

<table>
<tr><th>CRITERIA</th><th colspan="8">DECISION</th></tr>
<tr><td>1. PROBLEM</td><td colspan="2">No</td><td>Probably no</td><td colspan="2">Probably Yes</td><td>Yes</td><td>Varies</td><td>Don't know</td></tr>
<tr><td>2. BENEFITS</td><td colspan="2">Trivial</td><td>Small</td><td colspan="2">Moderate</td><td>Large</td><td>Varies</td><td>Don't know</td></tr>
<tr><td>3. HARMS</td><td colspan="2">Large</td><td>Moderate</td><td colspan="2">Small</td><td>Trivial</td><td>Varies</td><td>Don't know</td></tr>
<tr><td>4. QUALITY OF EVIDENCE</td><td colspan="2">Very low</td><td>Low</td><td colspan="2">Moderate</td><td>High</td><td colspan="2">No included studies</td></tr>
<tr><td>5. VALUES</td><td colspan="2">Important uncertainty or variability</td><td>Probably Important uncertainty or variability</td><td colspan="2">Probably no Important uncertainty or variability</td><td>No Important uncertainty or variability</td><td colspan="2">Varies</td></tr>
<tr><td>6. BALANCE OF EFFECTS</td><td>Favours the comparison</td><td>Probably favours the comparison</td><td colspan="2">Does not favour either the intervention or the comparison</td><td>Probably favours the intervention</td><td>Favours the intervention</td><td>Varies</td><td>Don't know</td></tr>
<tr><td>7. RESOURCES REQUIRED</td><td>Large costs</td><td>Moderate costs</td><td colspan="2">Negligible costs or savings</td><td>Large savings</td><td>Moderate savings</td><td>Varies</td><td>Don't know</td></tr>
<tr><td>8. CERTAINTY OF EVIDENCE OF REQUIRED RESOURCES</td><td>Very low</td><td>Low</td><td colspan="2">Moderate</td><td colspan="2">High</td><td colspan="2">No included studies</td></tr>
<tr><td>9. COST-EFFECTIVENESS</td><td>Favours the comparison</td><td>Probably favours the comparison</td><td colspan="2">Does not favour either the intervention or the comparison</td><td>Probably favours the intervention</td><td>Favours the intervention</td><td>Varies</td><td>Don't know</td></tr>
<tr><td>10EQUITY</td><td>Reduced</td><td>Probably Reduced</td><td colspan="2">Probably no impact</td><td>Probably Increased</td><td>Increased</td><td>Varies</td><td>Don't know</td></tr>
<tr><td>11ACCEPTABILITY</td><td>No</td><td>Probably no</td><td colspan="2">Probably Yes</td><td colspan="2">Yes</td><td>Varies</td><td>Don't know</td></tr>
<tr><td>12FEASIBILITY</td><td>No</td><td>Probably no</td><td colspan="2">Probably Yes</td><td colspan="2">Yes</td><td>Varies</td><td>Don't know</td></tr>
</table>

TYPE OF RECOMMENDATION

Strong recommendation against the intervention	Conditional recommendation against the intervention	Conditional recommendation for either the intervention or the comparison	Conditional recommendation for the intervention	Strong recommendation for the intervention
○	○	○	○	○

CONCLUSIONS

Reason for recommendation

Relapse rate is an important reference factor of RTX treatment.

Recommendation(text)

One-year relapse rate following first course of RTX treatment in children with FRNS/SDNS is decreased by 82% compared with the control group (RTX vs. placebo, decreased by 95%; RTX vs. CTX/CNI/MMF, decreased by 68%). (1D)

Subgroup considerations

1. Population subgroups: FRNS/SDNS+, FRNS/SDNS-;
2. Intervention subgroups: blank/placebo, other immunosuppressants (e.g. CTX/CNI/MMF).

Implementation considerations

1. The confidence interval of evidence merging results is too wide;
2. The quality of evidence is very low;
3. The limitation lies in the fractional intervention (first course of RTX treatment) and the isolated outcome indicator (12-month relapse rate).

Recommendation 2: Average 1-year relapse rate following first course of RTX treatment (375 mg•m-2, variable doses) is 41% in children with FRNS/SDNS (47% in FRNS/SDNS+, and 22% in FRNS/SDNS-). (1D)

Does RTX improve clinical outcomes in children aged 1-18 years with SSNS compared with other immunosuppressants or blank/placebo control?	
**STUDY TYPE**	Single-arm study and the RTX group in two-arm study
**POPULATION**	FRNS/SDNS, FRNS/SDNS+, FRNS/SDNS-
**INTERVENTION**	First course of RTX treatment: 1~4 doses, with a single dose of 375 mg·m^{-2}, once every 1~2 weeks
**COMPARISON**	No
**MAIN OUTCOMES**	12-month relapse rate of RTX treatment
**PERSPECTIVE**	Clinicians, social workers and parents of children with FRNS/SDNS
**SETTING**	Hospital
**CONFLICT OF INTERESTS**	No

ASSESSMENT

**1. PROBLEM: Is the problem a priority?**<br>Detailed judgments: The more serious or urgent a problem is, the more likely it is that an option that addresses the problem will be a priority.<br>RTX 治疗 FRNS/SDNS、FRNS/SDNS+和 FRNS/SDNS-患儿随访 12 月的复发率具有优先性吗？根据问题的严重性和紧急性程度判断其是否具有优先性。严重性和紧急性程度越高，优先性越大		
**JUDGEMENTS**	**RESEARCH EVIDENCE**	**ADDITIONAL CONSIDERATIONS**

☐ No<br>☐ Probably no<br>☐ Probably yes<br>☑ **Yes**<br>☐ Varies<br>☐ Uncertain	/	Vote Results: 1/13 chose “Probably Yes”, **12/13 chose “Yes”**.

2. Desirable effects: How substantial are the desirable anticipated effects?

Detailed judgments: How large are the desirable effects of the intervention taking into account the importance of the outcomes (how much they are valued), and the size of the effect (the likelihood of experiencing a benefit or how much of an improvement individuals would be likely to experience)?

RTX 治疗 FRNS/SDNS 患儿随访 12 月的复发率 41%、FRNS/SDNS+患儿随访 12 月的复发率 47%、FRNS/SDNS-患儿随访 12 月的复发率 22%的有益影响有多大？根据随访 12 月的复发率结局重要性和效应值大小（获益的可能性和个体情况改善的程度）对干预措施的有益影响进行判断

JUDGEMENTS

☐ Trivial
☐ Small
☐ Moderate
☑ **Large**
☐ Varies
☐ Uncertain

RESEARCH EVIDENCE

结局	研究数量和类型	事件数/总数	率（95%CI）	证据质量
**复发人数/总人数**				
**人群：FRNS/SDNS（部分仅单独激素，部分 CNI 或 MMF 其他免疫抑制剂使用后[440,21], SDNS 既往应用左旋咪唑，CTX 和/或 MMF[378], CNI 后[339], CNI 或 MMF 其他免疫抑制剂使用后 [133, 95, 69,219,230]）；不同利妥昔单抗剂量和剂次的 4 篇队列研究[339,354,379,196]和 8 篇病例系列报告[196,230,21,133,95,69,124,178]的 Meta 分析,385 例**				
随访 12 月	5 篇队列研究<br>[339,354,379,196]<br>8 篇病例系列报告<br>[21,230,133,95,69,124,178]<br>1 篇 NRSI[226]<br>7 篇 RCT<br>[354,54,340,30,193,337,496]	9/37[230]，<br>37/46[339],<br>3/7[124]，<br>7/10[354]<br>10/17[196],<br>8/28[95],<br>17/34[21]，<br>14/43[133]，<br>8/18[69],<br>37/61[178]，<br>12/85[379]	**RTX：**<br>41%(95%CI:31%~52%)<br>**FRNS/SDNS+：**<br>47% (95%CI:36%~59%)<br>**FRNS/SDNS-：**<br>22% (95%CI:9%~37%)	/

结局；患儿数量<br>研究类型和数量	相对效应（95%CI）	预期绝对效应值（事件数/例数）			证据质量	升降级说明
		对照组	干预组	风险差		
**复发人数/总人数**						
**人群：FRNS/SDNS-（仅激素治疗后 SDNS [54，340，193，275，337]，仅激素合并或不合并左旋咪唑[226]，SDNS 合并 CNI 依赖至少 2 年[30], SDNS 大剂量激素依赖，或既往应用左旋咪唑，CTX 和/或 MMF，后接受利妥昔单抗或 CNI 者[378]，部分利妥昔单抗合并或不合并 CNI，部分单独 CNI[199]）；干预组：利妥昔单抗；对照组：CNI 或 CTX 或单独激素或安慰剂；7 项 RCT[54，340，30，193，275，337，496]，2 篇队列研究[378，199]和 1 项 NRSI[226] Meta 分析，432 例**						
随访 12 月	**干预/对照**<br>**FRNS/SDNS**<br>（77/220；133/212）：<br>OR =0.18，95%CI:0.07~0.44<br>**FRNS/SDNS+**<br>（52/92；63/75）：<br>OR =0.19，95%CI:0.05~0.68<br>**FRNS/SDNS-**<br>（25/128；70/137）：<br>OR =0.15, 95%CI:0.03~0.68<br>**RTX 较安慰剂或空白对照：**<br>（46/86；66/69）<br>OR =0.05，95%CI:0.01~0.18<br>**RTX 较 CTX、CNI 或 MMF：**<br>（31/134；67/143）<br>OR =0.32，95%CI:0.13~0.82	7/13 人[378]<br>23/24 人[193]<br>22/60 人[54]<br>14/15 人[30] 总人数 16<br>6/8 人[199]<br>10/27 人[226]<br>9/20 人[275]<br>15/15 人[340]<br>14/15 人[337]<br>15/15 人[496]	5/10 人[378]<br>17/24 人[193]<br>6/60 人[54]<br>22/33 人[30] 总人数 35<br>6/10 人[199]<br>3/19 人[226]<br>9/20 人[275]<br>5/14 人[340] 总数 15<br>2/15 人[337]<br>2/15 人[496]	**FRNS/SDNS：**<br>每 1000 人减少 395 人（202~522）<br>**FRNS/SDNS+：**<br>每 1000 人减少 341 人（59~632）<br>**FRNS/SDNS-：**<br>每 1000 人减少 375 人（96~481）<br>**RTX 较安慰剂或空白对照：**<br>每 1000 人减少 433 人（158~776）<br>**RTX 较 CTX、CNI 或 MMF：**<br>每 1000 人减少 248 人（49~366）	**极低**	**降级因素：**<br>偏倚风险降 2 级<br>不一致性不降级<br>精确性降 1 级<br>发表偏倚<br>间接性不降级<br>**升级因素：无**

Refer to Meta Figure 3, Figure 4-1, Figure 4-2, Figure 5-1, Figure 5-2

ADDITIONAL CONSIDERATIONS

Vote Results: 1/13 chose “Moderate”, **12/13 chose “Large”**.

3. Undesirable effects: How substantial are the undesirable anticipated effects?

Detailed judgments: How large are the desirable effects of the intervention taking into account the importance of the outcomes (how much they are valued), and the size of the effect (the likelihood of experiencing a benefit or how much of an improvement individuals would be likely to experience)?

RTX 治疗 FRNS/SDNS 患儿随访 12 月的复发率 41%、FRNS/SDNS+患儿随访 12 月的复发率 47%、FRNS/SDNS-患儿随访 12 月的复发率 22%的不良影响有多大？根据随访 12 月的复发率和效应值大小（获益的可能性和个体情况改善的程度）对干预措施的不良影响进行判断

JUDGEMENTS	RESEARCH EVIDENCE	ADDITIONAL CONSIDERATIONS
☐ Large<br>☐ Moderate<br>☑ **Small**<br>☐ Trivial<br>☐ Varies<br>☐ Uncertain	/	Vote Results : 1/13 chose "Moderate", **7/13 chose "Small"**, 5/13 chose "Trivial" .

4. Certainty of the evidence: What is the overall certainty of the evidence of effects?

Detailed judgments: How good an indication does the research provide of the likely effects across all of the critical outcomes; i.e. the likelihood that the effects will be different enough from what the research found that it might affect a decision about the intervention?

RTX 治疗 FRNS/SDNS 患儿随访 12 月的复发率 41%、FRNS/SDNS+患儿随访 12 月的复发率 47%、FRNS/SDNS-患儿随访 12 月的复发率 22%有益影响和不良影响相关证据的总体质量？基于 RTX 治疗 FRNS/SDNS 患儿随访 12 月的复发率 41%、FRNS/SDNS+患儿随访 12 月的复发率 47%、FRNS/SDNS-患儿随访 12 月的复发率 22%的可能影响，判断 RTX 干预效果是否会对干预决策产生影响

JUDGEMENTS	RESEARCH EVIDENCE	ADDITIONAL CONSIDERATIONS
☑ **Very low**<br>☐ Low<br>☐ Moderate<br>☐ High<br>☐ No included studies	/	Vote Results: 4/13 chose "Low", **7/13 chose " Moderate "**, 2/13 chose "High" .

5. Values: Is there important uncertainty about or variability in how much people value the main outcomes?

Detailed judgments: How much do individuals value each of the main outcomes? Is uncertainty about how much they value each of the outcomes or variability in how much different individual value the outcomes large enough that it could lead to different decisions?

对 RTX 治疗 FRNS/SDNS 患儿随访 12 月的复发率 41%、FRNS/SDNS+患儿随访 12 月的复发率 47%、FRNS/SDNS-患儿随访 12 月的复发率 22%重视程度，是否因个体不同而存在不确定性和变化性？你对 RTX 治疗 FRNS/SDNS 患儿随访 12 月的复发率 41%、FRNS/SDNS+患儿随访 12 月的复发率 47%、FRNS/SDNS-患儿随访 12 月的复发率 22%的重视程度有多大？不确定性和变化性是否会导致不同的决策？不确定性体现在对上述推荐的理解程度；变化性体现在对上述推荐重视程度的差异。

JUDGEMENTS	RESEARCH EVIDENCE	ADDITIONAL CONSIDERATIONS
☐ Important uncertainty or variability	/	Vote Results : 1/13 chose

<table>
<tr><td>□ Possibly important uncertainty or variability
□ Probably no important uncertainty or variability
☑ No Important uncertainty or variability</td><td></td><td>“ Important uncertainty or variability”, 1/13 chose “Probably Important uncertainty or variability”, 5/13 chose “Probably no Important uncertainty or variability ”, 6/13 chose “ No Important uncertainty or variability”.</td></tr>
<tr><td colspan="3"><h2>6. Balance of effects: Does the balance between desirable and undesirable effects favour the intervention or the comparison?</h2>Detailed judgments: What is the balance between the desirable and undesirable effects, taking into account how much individuals value the main outcome, how substantial the desirable and undesirable effects are, the certainty of those estimates, discount rates, risk aversion and risk seeking?
权衡 RTX 治疗 FRNS/SDNS 患儿随访 12 月的复发率 41%、FRNS/SDNS+患儿随访 12 月的复发率 47%、FRNS/SDNS-患儿随访 12 月的复发率 22%的利弊从以下方面权衡利弊：个体对上述推荐意见的重视程度；利多大？；弊多大？；估计值的精确性；信心有多大？；风险多大？；可能规避风险么？</td></tr>
<tr><td>JUDGEMENTS</td><td>RESEARCH EVIDENCE</td><td>ADDITIONAL CONSIDERATIONS</td></tr>
<tr><td>□ Favours the comparison
□ Probably favours the comparison
□ Does not favour either the intervention or the comparison
□ Probably favours the intervention
☑ Favours the intervention
□ Varies
□ Uncertain</td><td>/</td><td>Vote Results：3/13 chose “Probably favours the intervention”, 10/13 chose “Favours the intervention”.</td></tr>
<tr><td colspan="3"><h2>7. Resources required: How large are the resource requirements (costs)?</h2>Detailed judgments: How large is the cost of the difference in resource use between the intervention and comparision?
RTX 治疗 FRNS/SDNS 患儿随访 12 月的复发率 41%、FRNS/SDNS+患儿随访 12 月的复发率 47%、FRNS/SDNS-患儿随访 12 月的复发率 22%的成本支出有多大？支出成本有多大？</td></tr>
<tr><td>JUDGEMENTS</td><td>RESEARCH EVIDENCE</td><td>ADDITIONAL CONSIDERATIONS</td></tr>
</table>

□ Large costs<br>□ Moderate costs<br>☑ **Negligible costs or savings**<br>□ Moderate savings<br>□ Large savings<br>□ Varies<br>□ Uncertain	Refer to recommendation 18	Vote Results : **7/13 chose "Negligible costs or savings"**, 5/13 chose " Moderate savings " , 1/13 chose "Large savings" .

8. Certainty of evidence of required resources: What is the certainty of the evidence of resource requirements (costs)?

成本支出的证据质量如何？对 RTX 治疗 FRNS/SDNS 患儿随访 12 月的复发率 41%、FRNS/SDNS+患儿随访 12 月的复发率 47%、FRNS/SDNS-患儿随访 12 月的复发率 22%的成本支出（包括药物、住院等费用）相关证据的确定性。

JUDGEMENTS	RESEARCH EVIDENCE	ADDITIONAL CONSIDERATIONS
☑ **Very low**<br>□ Low<br>□ Moderate<br>□ High<br>□ No included studies	Refer to recommendation 18	Vote Results : 2/13 chose " Very low " , **7/13 chose " Low "** , 3/13 chose " Moderate " , 1/13 chose "High" .<br><br>Referring to recommendation 18, the quality of evidence is very low.

9. Cost-effectiveness: Does the cost-effectiveness of the intervention favour the intervention or the comparison?

Detailed judgments: Is the intervention cost-effective, taking into account uncertainty about or variability in the costs, uncertainty about or variability in the net benefit, sensitivity analyses, and the reliability and applicability of the economic evaluation?

RTX 治疗 FRNS/SDNS 患儿随访 12 月的复发率 41%、FRNS/SDNS+患儿随访 12 月的复发率 47%、FRNS/SDNS-患儿随访 12 月的复发率 22%的成本效益从以下方面分析干预的成本效益：对支出成本的不确定性或变化性；对净利润的不确定性或变化性；敏感性分析；经济评估的可靠性和适用性。

JUDGEMENTS	RESEARCH EVIDENCE	ADDITIONAL CONSIDERATIONS

□ Favours the comparison<br>□ Probably favours the comparison<br>□ Does not favour either the intervention or the comparison<br>□ Probably favours the intervention<br>☑ **Favours the intervention**<br>□ Varies<br>□ No included studies	Refer to recommendation 18	Vote Results: 4/13 chose "Probably favours the intervention", **9/13 chose "Favours the intervention"**.

10. EQUITY: What would be the impact on health equity?

Detailed judgments: Are there plausible reasons for anticipating differences in the relative effectiveness of the intervention for disadvantaged subgroups or different baseline conditions across disadvantaged subgroups that affect the absolute effectiveness of the intervention or the importance of the problem?

对卫生公平性的影响？RTX 治疗 FRNS/SDNS 患儿随访 12 月的复发率 41%、FRNS/SDNS+患儿随访 12 月的复发率 47%、FRNS/SDNS-患儿随访 12 月的复发率 22%的相对有效性是否在弱势人群中有所降低，对此是否有合理的解释？弱势人群的不同基线水平会影响干预的绝对有效性或研究问题的重要性，对此是否有合理的解释？

JUDGEMENTS	RESEARCH EVIDENCE	ADDITIONAL CONSIDERATIONS
□ Reduced<br>☑ **Probably Reduced**<br>□ Probably no impact<br>□ Probably increased<br>□ Increased<br>□ Varies<br>□ Uncertain		Vote Results: 4/13 chose "Probably Reduced", 4/13 chose "Probably no impact", 2/13 chose "Probably increased", 2/13 chose "Increased", 1/13 chose "Varies".<br><br>The availability of medicines (remote areas, whether to seek medical treatment, etc.) and the comprehension of clinical knowledge may vary by different population, economy and regions. Therefore, after taking into account possible problems that disadvantaged groups (disability, poor economic conditions, etc.) may face, experts finally chose "Probably reduced" after discussion.

11. ACCEPTABILITY: Is the intervention acceptable to key stakeholders?

Detailed judgments: Are key stakeholders likely not to accept the distribution of the benefits, harms and costs; or the costs or undesirable effects in the short term for desirable effects

(benefits) in the future? Are they likely to disagree with the values attached to the desirable or undesirable effects, or not to accept the diagnostic intervention because of ethical concerns? 患儿及其家长是否接受 RTX 治疗 FRNS/SDNS 患儿随访 12 月的复发率 41%、FRNS/SDNS+患儿随访 12 月的复发率 47%、FRNS/SDNS-患儿随访 12 月的复发率 22%患儿及其家长是否会接受干预带来的获益、伤害及支出，或远期获益带来的短期内的不良反应；是否会对利弊判断背后的价值观念持反对意见；是否会出于伦理考虑拒绝诊断性治疗。		
**JUDGEMENTS**	**RESEARCH EVIDENCE**	**ADDITIONAL CONSIDERATIONS**
☐ No<br>☐ Probably no<br>☐ Probably yes<br>☑ **Yes**<br>☐ Varies<br>☐ Uncertain		Vote Results: 4/13 chose "Probably Yes", **9/13 chose "Yes"**。

12. FEASABILITY: Is the option feasible to implement?

Detailed judgments: Is it feasible to sustain use of the intervention and to address potential barriers to using it?

JUDGEMENTS	RESEARCH EVIDENCE	ADDITIONAL CONSIDERATIONS
☐ No<br>☐ Probably no<br>☐ Probably yes<br>☑ **Yes**<br>☐ Varies<br>☐ Uncertain		Vote Results: 1/13 chose "Probably Yes", **12/13 chose "Yes"**。

SUMMARY OF JUDGEMENTS (13/14)

CRITERIA	DECISION					
1. PROBLEM	No	Probably no	Probably Yes	**Yes**	Varies	Don't know
2. BENEFITS	Trivial	Small	Moderate	**Large**	Varies	Don't know
3. HARMS	Large	Moderate	**Small**	Trivial	Varies	Don't know
4. QUALITY OF EVIDENCE	**Very low**	Low	Moderate	High	No included studies	

<table>
<tr><th>CRITERIA</th><th colspan="8">DECISION</th></tr>
<tr><td>5. VALUES</td><td colspan="2">Important uncertainty or variability</td><td>Probably Important uncertainty or variability</td><td colspan="2">Probably no Important uncertainty or variability</td><td>No Important uncertainty or variability</td><td colspan="2">Varies</td></tr>
<tr><td>6. BALANCE OF EFFECTS</td><td>Favours the comparison</td><td>Probably favours the comparison</td><td colspan="2">Does not favour either the intervention or the comparison</td><td>Probably favours the intervention</td><td>Favours the intervention</td><td>Varies</td><td>Don’t know</td></tr>
<tr><td>7. RESOURCES REQUIRED</td><td>Large costs</td><td>Moderate costs</td><td colspan="2">Negligible costs or savings</td><td>Large savings</td><td>Moderate savings</td><td>Varies</td><td>Don’t know</td></tr>
<tr><td>8. CERTAINTY OF EVIDENCE OF REQUIRED RESOURCES</td><td>Very low</td><td>Low</td><td colspan="2">Moderate</td><td colspan="2">High</td><td colspan="2">No included studies</td></tr>
<tr><td>9. COST-EFFECTIVENESS</td><td>Favours the comparison</td><td>Probably favours the comparison</td><td colspan="2">Does not favour either the intervention or the comparison</td><td>Probably favours the intervention</td><td>Favours the intervention</td><td>Varies</td><td>Don’t know</td></tr>
<tr><td>10. EQUITY</td><td>Reduced</td><td>Probably Reduced</td><td colspan="2">Probably no impact</td><td>Probably Increased</td><td>Increased</td><td>Varies</td><td>Don’t know</td></tr>
<tr><td>11. ACCEPTABILITY</td><td>No</td><td>Probably no</td><td colspan="2">Probably Yes</td><td colspan="2">Yes</td><td>Varies</td><td>Don’t know</td></tr>
<tr><td>12. FEASIBILITY</td><td>No</td><td>Probably no</td><td colspan="2">Probably Yes</td><td colspan="2">Yes</td><td>Varies</td><td>Don’t know</td></tr>
</table>

TYPE OF RECOMMENDATION

**Strong recommendation against the intervention**	**Conditional recommendation against the intervention**	**Conditional recommendation for either the intervention or the comparison**	**Conditional recommendation for the intervention**	**Strong recommendation for the intervention**

○	○	○	○	○

CONCLUSIONS

Reason for recommendation

Relapse rate is an important reference factor of RTX treatment.

Recommendation(text)

Average 1-year relapse rate following first course of RTX treatment (375 mg·m-2, variable doses) is 41% in children with FRNS/SDNS (47% in FRNS/SDNS+, and 22% in FRNS/SDNS-). (1D)

Subgroup considerations

Population subgroups: FRNS/SDNS+**,** FRNS/SDNS-.

Implementation considerations

1. The quality of evidence is very low;
2. The limitation lies in the fractional intervention (first course of RTX treatment) and the isolated outcome indicator (12-month relapse rate) as well as data merging of single-arm and two-arm studies;
3. In consideration that data sources of doses use were not completely consistent and data description was not clear, no separated recommendation was generated after discussion.
4. Thirteen experts participated in this vote and one expert did not submit on time.

Recommendation 3: There is no difference in 2-year relapse rate between first course of RTX treatment and continuous immunosuppressive therapy in children with FRNS/SDNS. (2D)

Does RTX improve clinical outcomes in children aged 1-18 years with SSNS compared with other immunosuppressants or blank/placebo control?	
**STUDY TYPE**	Single-arm and two-arm study
**POPULATION**	FRNS/SDNS、FRNS/SDNS-、FRNS/SDNS+
**INTERVENTION**	RTX
**COMPARISON**	Blank, CTX, CNI
**MAIN OUTCOMES**	2~4-year relapse rate of RTX treatment
**PERSPECTIVE**	Clinicians, social workers and parents of children with FRNS/SDNS
**SETTING**	Hospital
**CONFLICT OF INTERESTS**	No

ASSESSMENT

1. PROBLEM: Is the problem a priority?<br>Detailed judgments: RTX 治疗 FRNS/SDNS、FRNS/SDNS+和 FRNS/SDNS-患儿随访 2~4 年复发率具有优先性吗？根据问题的严重性和紧急性程度判断其是否具有优先性。严重性和紧急性程度越高，优先性越大		
**JUDGEMENTS**	**RESEARCH EVIDENCE**	**ADDITIONAL CONSIDERATIONS**
☐ No<br>☐ Probably no<br>☐ Probably yes<br>☑ **Yes**<br>☐ Varies<br>☐ Uncertain	/	Vote Results： 2/14 chose “Probably no ”， 3/14 chose “Probably Yes”， **9/14 chose “Yes”**

2. Desirable effects: How substantial are the desirable anticipated effects?

Detailed judgments: How large are the desirable effects of the intervention taking into account the importance of the outcomes (how much they are valued), and the size of the effect (the likelihood of experiencing a benefit or how much of an improvement individuals would be likely to experience)?
首疗程 RTX 较对照治疗 FRNS/SDNS、FRNS/SDNS+和 FRNS/SDNS-患儿随访 2~4 年复发率无差异的有益影响有多大？根据随访 2~4 年复发率结局重要性和效应值大小（获益的可能性和个体情况改善的程度）对干预措施的有益影响进行判断

JUDGEMENTS

- ☐ Trivial
- ☑ Small
- ☐ Moderate
- ☐ Large
- ☐ Varies
- ☐ Uncertain

RESEARCH EVIDENCE

结局；患儿数量<br>研究类型和数量	相对效应（95%CI）	预期绝对效应值（事件数/例数）			证据质量	升降级说明
		对照组	干预组	风险差		
复发人数/总人数						
人群：FRNS/SDNS（仅激素治疗后 SDNS [340]，部分利妥昔单抗合并或不合并 CNI，部分单独 CNI[199]，部分仅单独激素，部分 CNI 或 MMF 其他免疫抑制剂使用后[440]）；干预组：利妥昔单抗；对照组：CNI 或 CTX 或单独激素或安慰剂；1 项 RCT[340]，2 篇队列研究[199，440] Meta 分析，163 例						
随访 24 月	FRNS/SDNS：<br>（44/61；80/102）<br>OR=0.65，95%CI:0.31~1.35	7/8 人[199]<br>15/15 人[340]<br>58/79 人[440]随访到总数不详	9/10 人[199]<br>6/9 人[340]总数 15<br>29/42 人[440]随访到总数不详	FRNS/SDNS：<br>每 1000 人减少 82 人(减少 254，增加 46)	极低	降级因素：<br>偏倚风险降 2 级<br>不一致性不降级<br>精确性降 2 级<br>发表偏倚不适用<br>间接性不降级

结局	研究数量和类型	事件数/总数	率（95%CI）	证据质量
复发人数/总人数				
人群：FRNS/SDNS（仅激素治疗后 SDNS [337]，部分仅单独激素，部分 CNI 或 MMF 或 MZR 其他免疫抑制剂使用后[21,215]，CNI 后[339]，CNI 或 MMF 其他免疫抑制剂使用后[133,95,230]），干预：利妥昔单抗，1 篇 RCT[337]，1 篇队列研究[339]和 5 篇病例系列报告[230，133，95，21,215]，294 例				
随访 24 月	1 篇 RCT[337]，<br>1 篇队列研究[339]<br>5 篇病例系列报告<br>[21,215，230,133，95]	21/27[21]，<br>47/51 [215]，<br>17/29[230]，<br>36/40[339]，<br>14/28[95]，<br>27/43[133]<br>7/15 [337]	**RTX：**<br>72%(95%CI:61% ~ 83%)<br>**FRNS/SDNS+：**<br>75% (95%CI:63%~86%)<br>**FRNS/SDNS-：**<br>54% (95%CI:33%~75%)	/
人群：FRNS/SDNS（仅激素治疗后 SDNS [337]，部分仅单独激素，部分 CNI 或 MMF 或 MZR 其他免疫抑制剂使用后[21,215]），干预：利妥昔单抗，1 篇 RCT[337]和 2 篇病例系列报告[21,215]，91 例				
人群：FRNS/SDNS，干预：利妥昔单抗，1 篇 RCT[337]，2 篇病例系列报告[21,215]，91 例				
随访 36 月	1 篇 RCT[337]<br>2 篇病例系列报告[21，215]	23/25[21]，<br>47/51[215]，<br>7/15 人[337]	**FRNS/SDNS：**<br>87%(95%CI:79% ~ 94%)<br>**FRNS/SDNS+：**<br>92% (95%CI:85%~98%)<br>**FRNS/SDNS -：**<br>47% (95%CI:21%~73%)	/
人群：FRNS/SDNS，干预：利妥昔单抗，1 篇 RCT[337]，15 例				
随访 48 月	1 篇 RCT[337]	7/15 人[337]	/	/

Refer to Meta Figure6, Figure 7 and Figure 8

ADDITIONAL CONSIDERATIONS

Vote Results： **6/14 chose "Small"**, 3/14 chose "Moderate", 5/14 chose "Large。"

After rating, the accuracy is reduced by 2 and the risk of bias is reduced by 2. Integrating expert opinions, the quality of evidence is very low.

The results of no difference in 2-year relapse rate may be out of variable study designs and control settings. With the addition of small sample size, the conclusion has limited reference significance.

3. Undesirable effects: How substantial are the undesirable anticipated effects?

Detailed judgments: How large are the desirable effects of the intervention taking into account the importance of the outcomes (how much they are valued), and the size of the effect (the likelihood of experiencing a benefit or how much of an improvement individuals would be likely to experience)?
首疗程 RTX 较对照治疗 FRNS/SDNS、FRNS/SDNS+和 FRNS/SDNS-患儿随访 2~4 年复发率无差异的不良影响有多大？根据随访 2~4 年复发率和效应值大小（获益的可能性和个体情况改善的程度）对干预措施的不良影响进行判断

JUDGEMENTS | **RESEARCH EVIDENCE** | **ADDITIONAL CONSIDERATIONS**

□ Large<br>□ Moderate<br>☑ **Small**<br>□ Trivial<br>□ Varies<br>□ Uncertain	/	Vote Results : 2/14 chose "Moderate", **8/14 chose "Small"**, 2/14 chose " Trivial ", 2/14 chose "Don't know".

4. Certainty of the evidence: What is the overall certainty of the evidence of effects?

Detailed judgments: How good an indication does the research provide of the likely effects across all of the critical outcomes; i.e. the likelihood that the effects will be different enough from what the research found that it might affect a decision about the intervention?

首疗程 RTX 较对照治疗 FRNS/SDNS、FRNS/SDNS+和 FRNS/SDNS-患儿随访 2~4 年复发率无差异的有益影响和不良影响相关证据的总体质量？基于 RTX 干预后随访 2~4 年复发率可能影响，判断 RTX 干预效果是否会对干预决策产生影响

JUDGEMENTS	RESEARCH EVIDENCE	ADDITIONAL CONSIDERATIONS
☑ **Very low**<br>□ Low<br>□ Moderate<br>□ High<br>□ No included studies	/	Vote Results : **11/14 chose "Very low"**, 3/14 chose "Low".

5. Values: Is there important uncertainty about or variability in how much people value the main outcomes?

Detailed judgments: How much do individuals value each of the main outcomes? Is uncertainty about how much they value each of the outcomes or variability in how much different individual value the outcomes large enough that it could lead to different decisions?

首疗程 RTX 较对照治疗 FRNS/SDNS、FRNS/SDNS+和 FRNS/SDNS-患儿随访 2~4 年复发率无差异的重视程度，是否因个体不同而存在不确定性和变化性？你对首疗程 RTX 较对照治疗 FRNS/SDNS、FRNS/SDNS+和 FRNS/SDNS-患儿随访 2~4 年复发率无差异重视程度有多大？不确定性和变化性是否会导致不同的决策？不确定性体现在对上述推荐的理解程度；变化性体现在对上述推荐重视程度的差异。

JUDGEMENTS	RESEARCH EVIDENCE	ADDITIONAL CONSIDERATIONS
□ Important uncertainty or variability<br>☑ **Probably Important uncertainty or variability**<br>□ Probably no important uncertainty or variability<br>□ No important uncertainty or variability	/	Vote Results: 1/14 chose "Important uncertainty or variability", **6/14 chose " Probably Important uncertainty or variability"**, 4/14 chose " Probably no Important uncertainty or variability", 3/14 chose "No Important uncertainty or variability".

6. Balance of effects: Does the balance between desirable and undesirable effects favour the intervention or the comparison?

Detailed judgments: What is the balance between the desirable and undesirable effects, taking into account how much individuals value the main outcome, how substantial the desirable and undesirable effects are, the certainty of those estimates, discount rates, risk aversion and risk seeking?

权衡首疗程 RTX 较对照治疗 FRNS/SDNS、FRNS/SDNS+和 FRNS/SDNS-患儿随访 2~4 年复发率无差异利弊从以下方面权衡利弊：个体对上述推荐的重视程度；利多大？；弊多大？；估计值的精确性；信心有多大？；风险多大？；可能规避风险么？

JUDGEMENTS	RESEARCH EVIDENCE	ADDITIONAL CONSIDERATIONS
□ Favours the comparison<br>□ Probably favours the comparison<br>☑ **Does not favour either the intervention or the comparison**<br>□ Probably favours the intervention<br>□ Favours the intervention<br>□ Varies<br>□ Uncertain	/	Vote Results: **6/14 chose "Does not favour either the intervention or the comparison"**, 3/14 chose "Probably favours the intervention", 4/14 chose "Favours the intervention", 1/14 chose "Don't know".

7. Resources required: How large are the resource requirements (costs)?

Detailed judgments: How large is the cost of the difference in resource use between the intervention and comparision?

首疗程 RTX 较对照治疗 FRNS/SDNS、FRNS/SDNS+和 FRNS/SDNS-患儿随访 2~4 年复发率无差异成本支出有多大？支出成本有多大？

JUDGEMENTS	RESEARCH EVIDENCE	ADDITIONAL CONSIDERATIONS
□ Large costs<br>□ Moderate costs<br>☑ **Negligible costs or savings**<br>□ Moderate savings<br>□ Large savings<br>□ Varies<br>□ Uncertain	Refer to recommendation 18	Vote Results: **9/14 chose "Negligible costs or savings"**, 1/14 chose "Moderate savings", 1/14 chose "Varies", 3/14 chose "Don't know".

8. Certainty of evidence of required resources: What is the certainty of the evidence of resource requirements (costs)?

Detailed judgments: How certain is the evidence of a difference for each type of resource use (eg. drugs, hospitalizations) and the cost of resources?

成本支出的证据质量如何？对首疗程 RTX 较对照治疗 FRNS/SDNS、FRNS/SDNS+和 FRNS/SDNS-患儿随访 2~4 年复发率无差异的成本支出（包括药物、住院等费用）相关证据的确定性。

JUDGEMENTS	RESEARCH EVIDENCE	ADDITIONAL CONSIDERATIONS

☑ **Very low**<br>□ Low<br>□ Moderate<br>□ High<br>□ No included studies	Refer to recommendation 18	Vote Results： **9/14 chose “Very low”**， 2/14 chose “Low”， 2/14 chose “Moderate”，1/14 chose “No included studies”．

9. Cost-effectiveness: Does the cost-effectiveness of the intervention favour the intervention or the comparison?

Detailed judgments: Is the intervention cost-effective, taking into account uncertainty about or variability in the costs, uncertainty about or variability in the net benefit, sensitivity analyses, and the reliability and applicability of the economic evaluation?

首疗程 RTX 较对照治疗 FRNS/SDNS、FRNS/SDNS+和 FRNS/SDNS-患儿随访 2~4 年复发率无差异的成本效益分析从以下方面分析干预的成本效益：对支出成本的不确定性或变化性；对净利润的不确定性或变化性；敏感性分析；经济评估的可靠性和适用性。

JUDGEMENTS	RESEARCH EVIDENCE	ADDITIONAL CONSIDERATIONS
□ Favours the comparison<br>□ Probably favours the comparison<br>□ Does not favour either the intervention or the comparison<br>□ Probably favours the intervention<br>☑ **Favours the intervention**<br>□ Varies<br>□ No included studies	Refer to recommendation 18	Vote Results：3/14 chose “Does not favour either the intervention or the comparison”，2/14 chose “Probably favours the intervention”， **4/14 chose “Favours the intervention”**，2/14 chose “Varies”，3/14 choose “No included studies”．

10. EQUITY: What would be the impact on health equity?

Detailed judgments: Are there plausible reasons for anticipating differences in the relative effectiveness of the intervention for disadvantaged subgroups or different baseline conditions across disadvantaged subgroups that affect the absolute effectiveness of the intervention or the importance of the problem?

对卫生公平性的影响？首疗程 RTX 较对照治疗 FRNS/SDNS、FRNS/SDNS+和 FRNS/SDNS-患儿随访 2~4 年复发率无差异的相对有效性是否在弱势人群中会更差，对此是否有合理的解释？弱势人群的不同基线水平会影响干预的绝对有效性或研究问题的重要性，对此是否有合理的解释？

JUDGEMENTS	RESEARCH EVIDENCE	ADDITIONAL CONSIDERATIONS
□ Reduced<br>☑ **Probably Reduced**<br>□ Probably no impact<br>□ Probably increased<br>□ Increased<br>□ Varies<br>□ Uncertain	/	Vote Results：**8/14 chose “Probably Reduced”**，2/14 chose “Probably no impact”，1/14 chose “Increased”，3/14 chose “Don’t know”．

11. ACCEPTABILITY: Is the intervention acceptable to key stakeholders?

Detailed judgments: Are key stakeholders likely not to accept the distribution of the benefits, harms and costs; or the costs or undesirable effects in the short term for desirable effects (benefits) in the future? Are they likely to disagree with the values attached to the desirable or undesirable effects, or not to accept the diagnostic intervention because of ethical concerns? 患儿及其家长是否接受首疗程 RTX 较对照治疗 FRNS/SDNS、FRNS/SDNS+和 FRNS/SDNS-患儿随访 2~4 年复发率无差异？患儿及其家长是否会接受干预带来的获益、伤害及支出，或远期获益带来的短期内的不良反应；是否会对利弊判断背后的价值观念持反对意见；是否会出于伦理考虑拒绝诊断性治疗。

JUDGEMENTS	RESEARCH EVIDENCE	ADDITIONAL CONSIDERATIONS
□ No<br>□ Probably no<br>☑ **Probably yes**<br>☑ **Yes**<br>□ Varies<br>□ Uncertain	/	Vote Results： 2/14 chose “Probably no ”， **6/14 chose “Probably Yes”, 6/14 chose “Yes” .**<br><br>Experts voted even, but the overall trend was “yes” .

12. FEASABILITY: Is the option feasible to implement?

Detailed judgments: Is it feasible to sustain use of the intervention and to address potential barriers to using it?

JUDGEMENTS	RESEARCH EVIDENCE	ADDITIONAL CONSIDERATIONS
□ No<br>□ Probably no<br>□ Probably yes<br>☑ **Yes**<br>□ Varies<br>□ Uncertain	/	Vote Results： 5/14 chose “Probably Yes”， **9/14 chose “Yes” .**

SUMMARY OF JUDGEMENTS

CRITERIA	DECISION							
**1. PROBLEM**	No		Probably no	Probably Yes		**Yes**	Varies	Don’t know
**2. BENEFITS**	Trivial		**Small**	Moderate		Large	Varies	Don’t know
**3. HARMS**	Large		Moderate	**Small**		Trivial	Varies	Don’t know
**4. QUALITY OF EVIDENCE**	**Very low**		Low	Moderate		High	No included studies	
**5. VALUES**	Important uncertainty or variability		**Probably Important uncertainty or variability**	Probably no Important uncertainty or variability		No Important uncertainty or variability	Varies	
**6. BALANCE OF EFFECTS**	Favours the comparison	Probably favours the comparison	**Does not favour either the intervention or the comparison**		Probably favours the intervention	Favours the intervention	Varies	Don’t know
**7. RESOURCES REQUIRED**	Large costs	Moderate costs	**Negligible costs or savings**		Large savings	Moderate savings	Varies	Don’t know
**8. CERTAINTY OF EVIDENCE OF REQUIRED RESOURCES**	**Very low**	Low	Moderate		High		No included studies	
**9. COST-EFFECTIVENESS**	Favours the comparison	Probably favours the comparison	Does not favour either the intervention or the comparison		Probably favours the intervention	**Favours the intervention**	Varies	Don’t know
**10 EQUITY**	Reduced	**Probably Reduced**	Probably no impact		Probably Increased	Increased	Varies	Don’t know
**11 ACCEPTABILITY**	No	Probably no	**Probably Yes**		**Yes**		Varies	Don’t know
**12 FEASIBILITY**	No	Probably no	Probably Yes		**Yes**		Varies	Don’t know

TYPE OF RECOMMENDATION

Strong recommendation against the intervention	Conditional recommendation against the intervention	Conditional recommendation for either the intervention or the comparison	Conditional recommendation for the intervention	Strong recommendation for the intervention
○	○	○	○	○

CONCLUSIONS

Reason for recommendation

Relapse rate is an important reference factor of RTX treatment.

Recommendation(text)

There is no difference in 2-year relapse rate between first course of RTX treatment and continuous immunosuppressive therapy in children with FRNS/SDNS. (2D)

Subgroup considerations

Population subgroups: FRNS/SDNS+, FRNS/SDNS-.

Implementation considerations

1. The results of no difference in 2-year relapse rate may be out of variable study designs and control settings.
2. Because of few studies and small samples, the conclusion has limited reference significance.

Research priorities

Population subgroups: FRNS/SDNS+, FRNS/SDNS-.

Recommendation 4: Median time to first relapse is about 10 months in children with FRNS/SDNS+ after first course of RTX treatment with a follow-up of ≥ 12 months. (1D)

Does RTX improve clinical outcomes in children aged 1-18 years of age with SSNS compared with other immunosuppressants or blank/placebo control?	
**STUDY TYPE**	Single-arm study
**POPULATION**	FRNS/SDNS+
**INTERVENTION**	RTX
**COMPARISON**	Blank, CTX, CNI
**MAIN OUTCOMES**	Median time to first relapse with a follow-up of ≥ 12 months
**PERSPECTIVE**	Clinicians, social workers and parents of children with FRNS/SDNS
**SETTING**	Hospital
**CONFLICT OF INTERESTS**	No

ASSESSMENT

1. PROBLEM: Is the problem a priority?

Detailed judgments: The more serious or urgent a problem is, the more likely it is that an option that addresses the problem will be a priority.
RTX 治疗 FRNS/SDNS+患儿随访 12 月以上首次复发时间具有优先性吗？根据问题的严重性和紧急性程度判断其是否具有优先性。严重性和紧急性程度越高，优先性越大

JUDGEMENTS	RESEARCH EVIDENCE	ADDITIONAL CONSIDERATIONS
□ No<br>□ Probably no<br>□ Probably yes<br>☑ **Yes**<br>□ Varies<br>□ Uncertain		Vote Results： 1/14 chose “Probably Yes”， **13/14 chose “Yes”．**

2. Desirable effects: How substantial are the desirable anticipated effects?

Detailed judgments: How large are the desirable effects of the intervention taking into account the importance of the outcomes (how much they are valued), and the size of the effect (the likelihood of experiencing a benefit or how much of an improvement individuals would be likely to experience)?

RTX 治疗 FRNS/SDNS+患儿随访 12 月以上首次复发时间 10 个月的有益影响有多大？根据随访 12 月以上首次复发时间结局重要性和效应值大小（获益的可能性和个体情况改善的程度）对干预措施的有益影响进行判断

JUDGEMENTS

- ☐ Trivial
- ☐ Small
- ☐ Moderate
- ☑ **Large**
- ☐ Varies
- ☐ Uncertain

RESEARCH EVIDENCE

结局	研究数量和类型	事件数/总数	率（95%CI）	证据质量
随访≥12 个月的首次复发时间				
**人群：FRNS/SDNS（部分仅单独激素，部分 CNI 或 MMF 其他免疫抑制剂使用后[440,21], SDNS 既往应用左旋咪唑，CTX 和/或 MMF[378], CNI 后[339], CNI 或 MMF 其他免疫抑制剂使用后［133, 95, 69,219,230]）；不同利妥昔单抗剂量和剂次的 3 篇队列研究[440，378，339]和 6 篇病例系列报告[21,219,230,133，95，69]的 Meta 分析, 461 例**				
首次复发时间	3 篇队列研究<br>[440，378，339]<br>6 篇病例系列报告<br>[21,219,230,133,95,69]	均数±标准差：<br>8.5±5.1 5/10 人[378]<br>10.23±9.25 32/39 人[21]<br>中位数（范围）：<br>19.53（0.07-48.33）39/43 人［133]，<br>14（1-73）26/38 人［440],<br>9.6(5.2-64.1) 24/37 人[230]，<br>9.25（0.5-36）32/39 人[21]，<br>10（0-40）59/81 人[219]<br>中位数（IQR）：<br>5.6(4.3 8.1)46 人［339],<br>11.2(8-17.7) 28 人［95],<br>6.45(4.48-8.62)18 人［69]	9.89（95%CI:7.14~12.65)月，其中[378] qw2-3 剂和[69] qw 2 剂未明确定义 d0。以给药后 45 天内减停所有其他免疫抑制剂后算 d0[339]	/
**人群：FRNS/SDNS+（部分仅单独激素，部分 CSA 或 MZR 其他免疫抑制剂使用后）,干预：利妥昔单抗, 1 篇病例系列报告[215]**				
中位数无复发时间<br>随访至少 36 个月	1 篇病例系列报告[215]	261 天	/	/

Refer to Meta Figure 9, Figure 10, Figure 11

ADDITIONAL CONSIDERATIONS

Vote Results：1/14 chose “Small”, 1/14 chose “Moderate”, **11/14 chose “Large”.**

3. Undesirable effects: How substantial are the undesirable anticipated effects?

Detailed judgments: How large are the desirable effects of the intervention taking into account the importance of the outcomes (how much they are valued), and the size of the effect (the likelihood of experiencing a benefit or how much of an improvement individuals would be likely to experience)?

RTX 治疗 FRNS/SDNS+患儿随访 12 月以上首次复发时间 10 个月的不良影响有多大？根据随访 12 月以上首次复发时间 10 个月和效应值大小（获益的可能性和个体情况改善的程度）对干预措施的不良影响进行判断

JUDGEMENTS

- ☐ Large
- ☐ Moderate
- ☑ **Small**
- ☐ Trivial
- ☐ Varies
- ☐ Uncertain

RESEARCH EVIDENCE

/

ADDITIONAL CONSIDERATIONS

Vote Results：2/14 chose “Large”, 1/14 chose “Moderate”, **8/14 chose “Small”**, 3/14 chose “Trivial”.

4. Certainty of the evidence: What is the overall certainty of the evidence of effects?

Detailed judgments: How good an indication does the research provide of the likely effects across all of the critical outcomes; i.e. the likelihood that the effects will be different enough from what the research found that it might affect a decision about the intervention?

RTX 干预后随访 12 月以上复发时间 10 个月的有益影响和不良影响相关证据的总体质量？基于 RTX 干预后随访 12 月以上复发时间 10 个月的可能影响，判断 RTX 干预效果是否会对干预决策产生影响		
**JUDGEMENTS**	**RESEARCH EVIDENCE**	**ADDITIONAL CONSIDERATIONS**
☑ **Very low**<br>□ Low<br>□ Moderate<br>□ High<br>□ No included studies	/	Vote Results： **7/14 chose “Very low”**，5/14 chose “Low”，2/14 chose “Moderate”.<br><br>All included were single-arm studies.

5. Values: Is there important uncertainty about or variability in how much people value the main outcomes?

Detailed judgments: How much do individuals value each of the main outcomes? Is uncertainty about how much they value each of the outcomes or variability in how much different individual value the outcomes large enough that it could lead to different decisions?

对 RTX 治疗 FRNS/SDNS+患儿随访 12 月以上复发时间 10 个月重视程度，是否因个体不同而存在不确定性和变化性？对 RTX 治疗 FRNS/SDNS+患儿随访 12 月以上复发时间 10 个月的重视程度有多大？不确定性和变化性是否会导致不同的决策？不确定性体现在对上述推荐的理解程度；变化性体现在对上述推荐重视程度的差异。

**JUDGEMENTS**	**RESEARCH EVIDENCE**	**ADDITIONAL CONSIDERATIONS**
□ Important uncertainty or variability<br>□ Possibly important uncertainty or variability<br>□ Probably no important uncertainty or variability<br>☑ **No Important uncertainty or variability**	/	Vote Results：5/14 chose “Probably Important uncertainty or variability”，4/14 chose “Probably no Important uncertainty or variability”，**5/14 chose “No Important uncertainty or variability”**.<br><br>After discussion and voting by experts, it was believed that there was no uncertainty and variability due to different individuals.

6. Balance of effects: Does the balance between desirable and undesirable effects favour the intervention or the comparison?

Detailed judgments: What is the balance between the desirable and undesirable effects, taking into account how much individuals value the main outcome, how substantial the desirable and undesirable effects are, the certainty of those estimates, discount rates, risk aversion and risk seeking?

权衡 RTX 治疗随访 12 月以上复发时间 10 个月的利弊。从以下方面权衡利弊：个体对上述推荐的重视程度；利多大？；弊多大？；估计值的精确性；信心有多大？；风险多大？；可能规避风险么？

**JUDGEMENTS**	**RESEARCH EVIDENCE**	**ADDITIONAL CONSIDERATIONS**

□ Favours the comparison<br>□ Probably favours the comparison<br>□ Does not favour either the intervention or the comparison<br>☑ **Probably favours the intervention**<br>□ Favours the intervention<br>□ Varies<br>□ Uncertain	/	Vote Results: **8/14 chose "Probably favours the intervention"**, 6/14 chose "Favours the intervention".<br><br>In the absence of control, only the advantages and disadvantages weighed here.

7. Resources required: How large are the resource requirements (costs)?

Detailed judgments: How large is the cost of the difference in resource use between the intervention and comparision?

RTX 治疗 FRNS/SDNS+患儿随访 12 月以上复发时间 10 个月成本支出有多大？支出成本有多大？

JUDGEMENTS	RESEARCH EVIDENCE	ADDITIONAL CONSIDERATIONS
□ Large costs<br>□ Moderate costs<br>☑ **Negligible costs or savings**<br>□ Moderate savings<br>□ Large savings<br>□ Varies<br>□ Uncertain	Refer to recommendation 18	Vote Results: **10/14 chose "Negligible costs or savings"**, 1/14 chose "Moderate savings", 1/14 chose "Large savings", 1/14 chose "Varies", 1/14 chose "Don't know".

8. Certainty of evidence of required resources: What is the certainty of the evidence of resource requirements (costs)?

How certain is the evidence of a difference for each type of resource use (eg. drugs, hospitalizations) and the cost of resources?

成本支出的证据质量如何？对 RTX 治疗随访 12 月以上复发时间 10 个月成本支出（包括药物、住院等费用）相关证据的确定性。

JUDGEMENTS	RESEARCH EVIDENCE	ADDITIONAL CONSIDERATIONS
☑ **Very low**<br>□ Low<br>□ Moderate<br>□ High<br>□ No included studies	Refer to recommendation 18	Vote Results: **8/14 chose "Very low"**, 5/14 chose "Low", 1/14 chose "Moderate".

9. Cost-effectiveness: Does the cost-effectiveness of the intervention favour the intervention or the comparison?

Detailed judgments: Is the intervention cost-effective, taking into account uncertainty about or variability in the costs, uncertainty about or variability in the net benefit, sensitivity analyses, and the reliability and applicability of the economic evaluation?
RTX 治疗随访 12 月以上复发时间 10 个月的成本效益。从以下方面分析干预的成本效益：对支出成本的不确定性或变化性；对净利润的不确定性或变化性；敏感性分析；经济评估的可靠性和适用性。

JUDGEMENTS	RESEARCH EVIDENCE	ADDITIONAL CONSIDERATIONS
□ Favours the comparison<br>□ Probably favours the comparison<br>□ Does not favour either the intervention or the comparison<br>□ Probably favours the intervention<br>☑ **Favours the intervention**<br>□ Varies<br>□ No included studies	Refer to recommendation 18	Vote Results：1/14 chose “Does not favour either the intervention or the comparison”，5/14 chose “Probably favours the intervention”，**7/14 chose “Favours the intervention”,** 1/14 chose “No included studies”.

10. EQUITY: What would be the impact on health equity?

Detailed judgments: Are there plausible reasons for anticipating differences in the relative effectiveness of the intervention for disadvantaged subgroups or different baseline conditions across disadvantaged subgroups that affect the absolute effectiveness of the intervention or the importance of the problem?
对卫生公平性的影响？RTX 治疗随访 12 月以上首次复发时间 10 个月的相对有效性是否在弱势人群中有所降低，对此是否有合理的解释？弱势人群的不同基线水平会影响干预的绝对有效性或研究问题的重要性，对此是否有合理的解释？

JUDGEMENTS	RESEARCH EVIDENCE	ADDITIONAL CONSIDERATIONS
□ Reduced<br>☑ **Probably Reduced**<br>□ Probably no impact<br>□ Probably increased<br>□ Increased<br>□ Varies<br>□ Uncertain	/	Vote Results：**8/14 chose “Probably Reduced”**，3/14 chose “Probably no impact”，2/14 chose ”Probably increased”，1/14 chose “Increased”.

11. ACCEPTABILITY: Is the intervention acceptable to key stakeholders?

Detailed judgments: Are key stakeholders likely not to accept the distribution of the benefits, harms and costs; or the costs or undesirable effects in the short term for desirable effects (benefits) in the future? Are they likely to disagree with the values attached to the desirable or undesirable effects, or not to accept the diagnostic intervention because of ethical concerns?
患儿及其家长是否接受 RTX 治疗 FRNS/SDNS+患儿随访 12 月以上复发时间 10 个月。患儿及其家长是否会接受干预带来的获益、伤害及支出，或远期获益带来的短期内的不良反应；是否会对利弊判断背后的价值观念持反对意见；是否会出于伦理考虑拒绝诊断性治疗。

JUDGEMENTS	RESEARCH EVIDENCE	ADDITIONAL CONSIDERATIONS
□ No	/	Vote Results：5/14 chose “Probably

☐ Probably no<br>☐ Probably yes<br>☑ **Yes**<br>☐ Varies<br>☐ Uncertain		Yes”, **8/14 chose “Yes”,** 1/14 chose “Don’ t know” .

12. FEASABILITY: Is the option feasible to implement?

Detailed judgments: Is it feasible to sustain use of the intervention and to address potential barriers to using it?

JUDGEMENTS	RESEARCH EVIDENCE	ADDITIONAL CONSIDERATIONS
☐ No<br>☐ Probably no<br>☐ Probably yes<br>☑ **Yes**<br>☐ Varies<br>☐ Uncertain	/	Vote Results: 3/14 chose “Probably Yes”, **11/14 chose “Yes” .**

SUMMARY OF JUDGEMENTS

<table>
<tr><th>CRITERIA</th><th colspan="8">DECISION</th></tr>
<tr><td>1. PROBLEM</td><td colspan="2">No</td><td>Probably no</td><td colspan="2">Probably Yes</td><td>Yes</td><td>Varies</td><td>Don’t know</td></tr>
<tr><td>2. BENEFITS</td><td colspan="2">Trivial</td><td>Small</td><td colspan="2">Moderate</td><td>Large</td><td>Varies</td><td>Don’t know</td></tr>
<tr><td>3. HARMS</td><td colspan="2">Large</td><td>Moderate</td><td colspan="2">Small</td><td>Trivial</td><td>Varies</td><td>Don’t know</td></tr>
<tr><td>4. QUALITY OF EVIDENCE</td><td colspan="2">Very low</td><td>Low</td><td colspan="2">Moderate</td><td>High</td><td colspan="2">No included studies</td></tr>
<tr><td>5. VALUES</td><td colspan="2">Important uncertainty or variability</td><td>Probably Important uncertainty or variability</td><td colspan="2">Probably no Important uncertainty or variability</td><td>No Important uncertainty or variability</td><td colspan="2">Varies</td></tr>
<tr><td>6. BALANCE OF EFFECTS</td><td>Favours the comparison</td><td>Probably favours the comparison</td><td colspan="2">Does not favour either the intervention or the comparison</td><td>Probably favours the intervention</td><td>Favours the intervention</td><td>Varies</td><td>Don’t know</td></tr>
<tr><td>7. RESOURCES REQUIRED</td><td>Large costs</td><td>Moderate costs</td><td colspan="2">Negligible costs or savings</td><td>Large savings</td><td>Moderate savings</td><td>Varies</td><td>Don’t know</td></tr>
<tr><td>8. CERTAINTY OF EVIDENCE OF REQUIRED RESOURCES</td><td>Very low</td><td>Low</td><td colspan="2">Moderate</td><td colspan="2">High</td><td colspan="2">No included studies</td></tr>
<tr><td>9. COST-EFFECTIVENESS</td><td>Favours the comparison</td><td>Probably favours the comparison</td><td colspan="2">Does not favour either the intervention or the comparison</td><td>Probably favours the intervention</td><td>Favours the intervention</td><td>Varies</td><td>Don’t know</td></tr>
<tr><td>10. EQUITY</td><td>Reduced</td><td>Probably Reduced</td><td colspan="2">Probably no impact</td><td>Probably Increased</td><td>Increased</td><td>Varies</td><td>Don’t know</td></tr>
<tr><td>11. ACCEPTABILITY</td><td>No</td><td>Probably no</td><td colspan="2">Probably Yes</td><td colspan="2">Yes</td><td>Varies</td><td>Don’t know</td></tr>
<tr><td>12. FEASIBILITY</td><td>No</td><td>Probably no</td><td colspan="2">Probably Yes</td><td colspan="2">Yes</td><td>Varies</td><td>Don’t know</td></tr>
</table>

TYPE OF RECOMMENDATION

Strong recommendation against the intervention	Conditional recommendation against the intervention	Conditional recommendation for either the intervention or the comparison	Conditional recommendation for the intervention	Strong recommendation for the intervention
○	○	○	○	○

CONCLUSIONS

Reason for recommendation

Median time to first relapse after first course of RTX treatment is an important factor to consider.

Recommendation(text)

Median time to first relapse is about 10 months in children with FRNS/SDNS+ after first course of RTX treatment with a follow-up of ≥ 12 months. (1D)

Recommendation 5: Significant benefit of RTX treatment for children with FRNS/SDNS is achieved in terms of 1-year cumulative steroid dose reduction compared with other immunosuppressants (RTX vs. FK, lowered by 0.15 mg•kg-1•d-1; RTX vs. placebo, reduced by 0.26 mg•kg-1•d-1). (1D)

Does RTX have an effect on cumulative steroid dose in children aged 1-18 years with SSNS?	
**STUDY TYPE**	Two-arm study
**POPULATION**	FRNS/SDNS
**INTERVENTION**	RTX
**COMPARISON**	Placebo, FK
**MAIN OUTCOMES**	1-year cumulative steroid dose reduction ($mg{\cdot}kg^{-1}{\cdot}d^{-1}$)
**PERSPECTIVE**	Clinicians, social workers and parents of children with FRNS/SDNS
**SETTING**	Hospital
**CONFLICT OF INTERESTS**	No

ASSESSMENT

1. PROBLEM: Is the problem a priority?

Detailed judgments: Detailed judgments: The more serious or urgent a problem is, the more likely it is that an option that addresses the problem will be a priority.
FRNS/SDNS 患儿接受 RTX 治疗平均 1 年激素累积剂量减少问题具有优先性么？根据问题的严重性和紧急性程度判断其是否具有优先性。严重性和紧急性程度越高，优先性越大

JUDGEMENTS	RESEARCH EVIDENCE	ADDITIONAL CONSIDERATIONS
☐ No<br>☐ Probably no<br>☐ Probably yes<br>☑ **Yes**<br>☐ Varies<br>☐ Uncertain	/	Vote Results : 1/14 chose " Probably Yes " , **13/14 chose "Yes" .**

2. Desirable effects: How substantial are the desirable anticipated effects?

Detailed judgments: How large are the desirable effects of the intervention taking into account the importance of the outcomes (how much they are valued), and the size of the effect (the likelihood of experiencing a benefit or how much of an improvement individuals would be likely to experience)?

FRNS/SDNS 患儿接受 RTX 治疗平均 1 年激素累积剂量（$mg\cdot kg^{-1}\cdot d^{-1}$）减少 0.16，较 FK 减少 0.15，较空白对照减少 0.26 的有益影响有多大？根据结局重要性（受重视的程度）和效应值大小（获益的可能性和个体情况改善的程度）对干预措施的有益影响进行判断

JUDGEMENTS

- ☐ Trivial
- ☐ Small
- ☐ Moderate
- ☑ **Large**
- ☐ Varies
- ☐ Uncertain

RESEARCH EVIDENCE

结局；患儿数量<br>研究类型和数量	相对效应（95%CI）	预期绝对效应值（事件数/例数）			证据质量	升降级说明
		对照组	干预组	风险差		
激素用量（随访时间＞3 个月）						
**患者分组：FRNS/SDNS（仅激素治疗后 SDNS [54,193]，SDNS 合并 CNI 依赖至少 2 年[30],SDNS 既往应用左旋咪唑，CTX 和/或 MMF[378]）；干预组：利妥昔单抗；对照组：CNI 或安慰剂；3 项 RCT[54,30,193]和 1 项队列研究[378],242 例**						
12 月累积激素剂量 mg/kg/d	**FRNS/SDNS：**<br>MD=-0.16，<br>95%CI:-0.20~-0.12，<br>I^2=76%,P<0.01<br>**较对照组 FK：**<br>MD=-0.15，<br>95%CI:-0.18~-0.11<br>**较对照组安慰剂和空白对照：**<br>MD=-0.26，<br>95%CI:-0.36~-0.16<br>**FRNS/SDNS+：**<br>MD=-0.14，<br>95%CI:-0.21~-0.08<br>**FRNS/SDNS-：**<br>MD=-0.17，<br>95%CI:-0.28~-0.07	70.9 ± 26.3，13 人[378] mg/kg/y<br>20.85±9.28，24 人[193] mg/m2/d<br>86.3±58.0，60 人[54] mg/kg/y<br>0.40±0.28，16 人[30] mg/kg/d	46.1 ± 42.1，10 人[378] mg/kg/y<br>9.12±5.88，24 人[193] mg/m2/d<br>25.8±27.8，60 人[54] mg/kg/y<br>0.24±0.19，35 人[30] mg/kg/d	/	**极低**	**降级因素：**<br>偏倚风险降 2 级<br>不一致性不降级<br>精确性降 1 级<br>发表偏倚不适用<br>间接性不降级

结局；患儿数量<br>研究类型和数量	相对效应（95%CI）	预期绝对效应值（事件数/例数）			证据质量	升降级说明
		对照组	干预组	风险差		
激素用量（随访时间＞3 个月）						
**患者分组：FRNS/SDNS（仅激素治疗后 SDNS [54,193]，SDNS 合并 CNI 依赖至少 2 年[30],SDNS 既往应用左旋咪唑，CTX 和/或 MMF 和或 CNI[378，379]）；自身前后 12 月累积激素剂量，干预组：利妥昔单抗；3 项 RCT[54,30,193]和 2 项队列研究[378,379],209 例**						
自身前后 12 月累积激素剂量 mg/kg/d	**FRNS/SDNS+：**<br>MD=-0.35，<br>95%CI:-0.38~-0.31，<br>I^2=79%，P<0.01	140.5±59.0，10 人[378] mg/kg/y<br>148.1±82.3，85 人[379] mg/kg/y<br>0.46±0.4，35 人[30] mg/kg/d<br>246.0±48.0，60 人[54] mg/kg/y<br>19.13±9.94，19 人[193] mg/m2/d	46.1±42.1，10 人[378] mg/kg/y<br>43.6±36.6，85 人[379] mg/kg/y<br>0.24±0.19，35 人[30] mg/kg/d<br>86.3±58.0，60 人[54] mg/kg/y<br>8.37±5.62，19 人[193] mg/m2/d	/	**低**	**降级因素：**<br>偏倚风险降 2 级<br>不一致性不降级<br>精确性不降级<br>发表偏倚不适用<br>间接性不降级
**患者分组：仅激素治疗后 SDNS/FRNS；干预：利妥昔单抗-；对照：CNI，1 篇 RCT[275], 41 例**						
12 月累积激素剂量	/	20 人，mg/kg/d.<br>median (IQR)0.11 (0.04, 0.19)<br>分组前 0.43(0.36,0.72)	21 人，mg/kg/d.<br>median (IQR) 0.11 (0.05, 0.24)<br>分组前 0.44(0.29,0.68)	Risk or mean difference:<br>−0.08 [−0.19,0.03], p=0.15	**低**	**降级因素：**<br>偏倚风险降 1 级<br>不一致性不适用<br>精确性降 1 级<br>发表偏倚不适用<br>间接性不降级
**患者分组：仅激素治疗后 SDNS/FRNS；干预：利妥昔单抗-；对照：CTX，1 篇 NRSI 研究[226],46 例**						
随访 12 月激素剂量	/	27 人，激素剂量自治疗前 1.02 减少至 0.36mg/kg qod，前后差异 0.66±0.63, p<0.001	19 人，激素剂量自治疗前 0.86 减少至 0.08mg/kg qod ，前后差异 0.78±0.23, p<0.001	/	**极低**	**降级因素：**<br>偏倚风险降 2 级<br>不一致性不适用<br>精确性降 1 级<br>发表偏倚不适用<br>间接性不降级
**患者分组：FRNS/SDNS+；干预：利妥昔单抗+；自身给药 1 篇病例系列报道[135],10 例**						
激素平均剂量，随访平均 17(范围 13–21)月	/	0.39 ± 0.18 mg/kg/d	0.15 ± 0.14 mg/kg/d	减少 63%，p <0.01	**极低**	/

Refer to Meta Figure12, Figure 13, Figure 14

ADDITIONAL CONSIDERATIONS

Vote Results : 2/14 chose “Moderate”, **12/14 chose “Large”.**

3. Undesirable effects: How substantial are the undesirable anticipated effects?

Detailed judgments: How large are the desirable effects of the intervention taking into account the importance of the outcomes (how much they are valued), and the size of the effect (the likelihood of experiencing a benefit or how much of an improvement individuals would be likely to experience)?<br>FRNS/SDNS 患儿接受 RTX 治疗平均 1 年激素累积剂量（mg·kg^{-1}·d^{-1}）减少 0.16，较 FK 减少 0.15，较空白对照减少 0.26 的不良影响有多大？根据结局重要性（受重视的程度）和效应值大小（获益的可能性和个体情况改善的程度）对干预措施的不良影响进行判断		
**JUDGEMENTS**	**RESEARCH EVIDENCE**	**ADDITIONAL CONSIDERATIONS**
☐ Large<br>☐ Moderate<br>☐ small<br>☑ **Trivial**<br>☐ Varies<br>☐ Uncertain	/	Vote Results : 1/14 chose " Large " , 3/14 chose " Moderate " , 3/14 chose "Small", **7/14 chose "Trivial"** .

4. Certainty of the evidence: What is the overall certainty of the evidence of effects?

Detailed judgments: How good an indication does the research provide of the likely effects across all of the critical outcomes; i.e. the likelihood that the effects will be different enough from what the research found that it might affect a decision about the intervention?<br>FRNS/SDNS 患儿接受 RTX 治疗平均 1 年激素累积剂量（mg·kg^{-1}·d^{-1}）减少 0.16，较 FK 减少 0.15，较空白对照减少 0.26 的有益影响和不良影响相关证据的总体质量？ 基于 FRNS/SDNS 患儿接受 RTX 治疗平均 1 年激素累积剂量（mg·kg^{-1}·d^{-1}）减少 0.16，较 FK 减少 0.15，较空白对照减少 0.26 的可能影响，判断干预效果是否会对干预决策产生影响		
**JUDGEMENTS**	**RESEARCH EVIDENCE**	**ADDITIONAL CONSIDERATIONS**
☑ **Very low**<br>☐ Low<br>☐ Moderate<br>☐ High<br>☐ No included studies	/	Vote Results : 1/14 chose "Very low", **11/14 chose "Low",** 2/14 chose "Moderate" .<br><br>Considering multiple integrated evidence including evidence of very low quality, the overall estimation is very low.

5. Values: Is there important uncertainty about or variability in how much people value the main outcomes?

Detailed judgments: How much do individuals value each of the main outcomes? Is uncertainty about how much they value each of the outcomes or variability in how much different individual value the outcomes large enough that it could lead to different decisions?<br>对 FRNS/SDNS 患儿接受 RTX 治疗平均 1 年激素累积剂量（mg·kg^{-1}·d^{-1}）减少 0.16，较 FK 减少 0.15，较空白对照减少 0.26 的重视程度，是否因个体不同而存在不确定性和变化性？个体对 FRNS/SDNS 患儿接受 RTX 治疗平均 1 年激素累积剂量（mg·kg^{-1}·d^{-1}）减少 0.16，较 FK 减少 0.15，较空白对照减少 0.26 的重视程度有多大？不确定性和变化性是否会导致不同的决策？不确定性体现在对上述推荐的理解程度；变化性体现在对上述推荐重视程度的差异。		
**JUDGEMENTS**	**RESEARCH EVIDENCE**	**ADDITIONAL CONSIDERATIONS**

□ Important uncertainty or variability<br>□ Possibly important uncertainty or variability<br>□ Probably no important uncertainty or variability<br>☑ **No Important uncertainty or variability**	/	Vote Results : 2/14 chose "Probably no Important uncertainty or variability", **12/14 chose "No Important uncertainty or variability".**

6. Balance of effects: Does the balance between desirable and undesirable effects favour the intervention or the comparison?

Detailed judgments: What is the balance between the desirable and undesirable effects, taking into account how much individuals value the main outcome, how substantial the desirable and undesirable effects are, the certainty of those estimates, discount rates, risk aversion and risk seeking?

权衡 FRNS/SDNS 患儿接受 RTX 治疗平均 1 年激素累积剂量（mg·kg-1·d-1）减少 0.16，较 FK 减少 0.15，较空白对照减少 0.26 的利弊后更倾向干预组还是对照组？从以下方面权衡利弊：个体对上述推荐的重视程度；利多大？；弊多大？；估计值的精确性；信心有多大？；风险多大？；可能规避风险么？

JUDGEMENTS	RESEARCH EVIDENCE	ADDITIONAL CONSIDERATIONS
□ Favours the comparison<br>□ Probably favours the comparison<br>□ Does not favour either the intervention or the comparison<br>□ Probably favours the intervention<br>☑ **Favours the intervention**<br>□ Varies<br>□ Uncertain	/	Vote Results : 3/14 chose "Probably favours the intervention", **11/14 chose "Favours the intervention".**

7. Resources required: How large are the resource requirements (costs)?

Detailed judgments: How large is the cost of the difference in resource use between the intervention and comparision?

FRNS/SDNS 患儿接受 RTX 治疗平均 1 年激素累积剂量（mg·kg^{-1}·d^{-1}）减少 0.16，较 FK 减少 0.15，较空白对照减少 0.26 的的支出成本差异有多大?

JUDGEMENTS	RESEARCH EVIDENCE	ADDITIONAL CONSIDERATIONS

□ Large costs<br>□ Moderate costs<br>☑ **Negligible costs or savings**<br>□ Moderate savings<br>□ Large savings<br>□ Varies<br>□ Uncertain	Refer to recommendation 18	Vote Results : **8/14 chose "Negligible costs or savings",** 2/14 chose "Moderate savings", 1/14 chose "Large savings", 3/14 chose "Don' t know" .

8. Certainty of evidence of required resources: What is the certainty of the evidence of resource requirements (costs)?

Detailed judgments: How certain is the evidence of a difference for each type of resource use (eg. drugs, hospitalizations) and the cost of resources?

成本支出的证据质量如何？对 FRNS/SDNS 患儿接受 RTX 治疗平均 1 年激素累积剂量（$mg{\cdot}kg^{-1}{\cdot}d^{-1}$）减少 0.16，较 FK 减少 0.15，较空白对照减少 0.26 的成本支出（包括药物、住院等费用）相关证据的确定性。

JUDGEMENTS	RESEARCH EVIDENCE	ADDITIONAL CONSIDERATIONS
☑ **Very low**<br>□ Low<br>□ Moderate<br>□ High<br>□ No included studies	Refer to recommendation 18	Vote Results : 1/14 chose " Very low" , **8/14 chose " Low" ,** 2/14 chose " Moderate " , 3/14 chose "No included studies" .<br><br>Referring to recommendation 18, the quality of evidence is very low.

9. Cost-effectiveness: Does the cost-effectiveness of the intervention favour the intervention or the comparison?

Detailed judgments: Is the intervention cost-effective, taking into account uncertainty about or variability in the costs, uncertainty about or variability in the net benefit, sensitivity analyses, and the reliability and applicability of the economic evaluation?

对 FRNS/SDNS 患儿接受 RTX 治疗平均 1 年激素累积剂量（$mg{\cdot}kg^{-1}{\cdot}d^{-1}$）减少 0.16，较 FK 减少 0.15，较空白对照减少 0.26 的干预的成本效益分析更倾向干预组还是对照组？从以下方面分析干预的成本效益：对支出成本的不确定性或变化性；对净利润的不确定性或变化性；敏感性分析；经济评估的可靠性和适用性。

JUDGEMENTS	RESEARCH EVIDENCE	ADDITIONAL CONSIDERATIONS

☐ Favours the comparison<br>☐ Probably favours the comparison<br>☐ Does not favour either the intervention or the comparison<br>☐ Probably favours the intervention<br>☑ **Favours the intervention**<br>☐ Varies<br>☐ No included studies	Refer to recommendation 18	Vote Results: 1/14 chose "Probably favours the comparison", 3/14 chose "Probably favours the intervention", **10/14 chose "Favours the intervention".**

10. EQUITY: What would be the impact on health equity?

Detailed judgments: Are there plausible reasons for anticipating differences in the relative effectiveness of the intervention for disadvantaged subgroups or different baseline conditions across disadvantaged subgroups that affect the absolute effectiveness of the intervention or the importance of the problem?

对卫生公平性的影响？FRNS/SDNS 患儿接受 RTX 治疗平均 1 年激素累积剂量（mg·kg^{-1}·d^{-1}）减少 0.16，较 FK 减少 0.15，较空白对照减少 0.26 的相对有效性是否在弱势人群中有所减少，对此是否有合理的解释？弱势人群的不同基线水平会影响干预的绝对有效性或研究问题的重要性，对此是否有合理的解释？

JUDGEMENTS	RESEARCH EVIDENCE	ADDITIONAL CONSIDERATIONS
☐ Reduced<br>☑ **Probably Reduced**<br>☐ Probably no impact<br>☐ Probably increased<br>☐ Increased<br>☐ Varies<br>☐ Uncertain	/	Vote Results: **9/14 chose "Probably Reduced",** 3/14 chose "Probably no impact", 1/14 chose "Increased", 1/14 chose "Don't know".

11. ACCEPTABILITY: Is the intervention acceptable to key stakeholders?

Detailed judgments: Are key stakeholders likely not to accept the distribution of the benefits, harms and costs; or the costs or undesirable effects in the short term for desirable effects (benefits) in the future? Are they likely to disagree with the values attached to the desirable or undesirable effects, or not to accept the diagnostic intervention because of ethical concerns?

患儿及其家长是否接受 FRNS/SDNS 患儿接受 RTX 治疗平均 1 年激素累积剂量（mg·kg-1·d-1）减少 0.16，较 FK 减少 0.15，较空白对照减少 0.26，患儿及其家长是否会接受干预带来的获益、伤害及支出，或远期获益带来的短期内的不良反应；是否会对利弊判断背后的价值观念持反对意见；是否会出于伦理考虑拒绝诊断性治疗。

JUDGEMENTS	RESEARCH EVIDENCE	ADDITIONAL CONSIDERATIONS
☐ No<br>☐ Probably no<br>☐ Probably yes<br>☑ **Yes**<br>☐ Varies<br>☐ Uncertain	/	Vote Results: 3/14 chose "Probably Yes", **11/14 chose "Yes".**

12. FEASABILITY: Is the option feasible to implement?

Detailed judgments: Is it feasible to sustain use of the intervention and to address potential barriers to using it?

JUDGEMENTS	RESEARCH EVIDENCE	ADDITIONAL CONSIDERATIONS
☐ No<br>☐ Probably no<br>☐ Probably yes<br>☑ **Yes**<br>☐ Varies<br>☐ Uncertain	/	Vote Results： **14/14 chose "Yes".**

SUMMARY OF JUDGEMENTS

<table>
<tr><th>CRITERIA</th><th colspan="8">DECISION</th></tr>
<tr><td>1. PROBLEM</td><td colspan="2">No</td><td>Probably no</td><td colspan="2">Probably Yes</td><td>Yes</td><td>Varies</td><td>Don’ t know</td></tr>
<tr><td>2. BENEFITS</td><td colspan="2">Trivial</td><td>Small</td><td colspan="2">Moderate</td><td>Large</td><td>Varies</td><td>Don’ t know</td></tr>
<tr><td>3. HARMS</td><td colspan="2">Large</td><td>Moderate</td><td colspan="2">Small</td><td>Trivial</td><td>Varies</td><td>Don’ t know</td></tr>
<tr><td>4. QUALITY OF EVIDENCE</td><td colspan="2">Very low</td><td>Low</td><td colspan="2">Moderate</td><td>High</td><td colspan="2">No included studies</td></tr>
<tr><td>5. VALUES</td><td colspan="2">Important uncertainty or variability</td><td>Probably Important uncertainty or variability</td><td colspan="2">Probably no Important uncertainty or variability</td><td>No Important uncertainty or variability</td><td colspan="2">Varies</td></tr>
<tr><td>6. BALANCE OF EFFECTS</td><td>Favours the comparison</td><td>Probably favours the comparison</td><td colspan="2">Does not favour either the intervention or the comparison</td><td>Probably favours the intervention</td><td>Favours the intervention</td><td>Varies</td><td>Don’ t know</td></tr>
<tr><td>7. RESOURCES REQUIRED</td><td>Large costs</td><td>Moderate costs</td><td colspan="2">Negligible costs or savings</td><td>Large savings</td><td>Moderate savings</td><td>Varies</td><td>Don’ t know</td></tr>
<tr><td>8. CERTAINTY OF EVIDENCE OF REQUIRED RESOURCES</td><td>Very low</td><td>Low</td><td colspan="2">Moderate</td><td colspan="2">High</td><td colspan="2">No included studies</td></tr>
<tr><td>9. COST-EFFECTIVENESS</td><td>Favours the comparison</td><td>Probably favours the comparison</td><td colspan="2">Does not favour either the intervention or the comparison</td><td>Probably favours the intervention</td><td>Favours the intervention</td><td>Varies</td><td>Don’ t know</td></tr>
<tr><td>10. EQUITY</td><td>Reduced</td><td>Probably Reduced</td><td colspan="2">Probably no impact</td><td>Probably Increased</td><td>Increased</td><td>Varies</td><td>Don’ t know</td></tr>
<tr><td>11. ACCEPTABILITY</td><td>No</td><td>Probably no</td><td colspan="2">Probably Yes</td><td colspan="2">Yes</td><td>Varies</td><td>Don’ t know</td></tr>
<tr><td>12. FEASIBILITY</td><td>No</td><td>Probably no</td><td colspan="2">Probably Yes</td><td colspan="2">Yes</td><td>Varies</td><td>Don’ t know</td></tr>
</table>

TYPE OF RECOMMENDATION

Strong recommendation against the intervention	Conditional recommendation against the intervention	Conditional recommendation for either the intervention or the comparison	Conditional recommendation for the intervention	Strong recommendation for the intervention
○	○	○	○	○

CONCLUSIONS

Reason for recommendation

Steroid dose is an important factor to consider.

Recommendation(text)

Significant benefit of RTX treatment for children with FRNS/SDNS is achieved in terms of 1-year cumulative steroid dose reduction compared with other immunosuppressants (RTX vs. FK, lowered by 0.15 mg·kg-1·d-1; RTX vs. placebo, reduced by 0.26 mg·kg-1·d-1). (1D)

Implementation considerations

The price of steroid

Recommendation 6: RTX treatment benefits on the time to steroid withdrawal in children with FRNS/SDNS compared with other immunosuppressants. (1D)

Does RTX have an effect on steroid withdrawal in children aged 1-18 years with SSNS?	
**STUDY TYPE**	Two-arm study
**POPULATION**	FRNS/SDNS、FRNS/SDNS+
**INTERVENTION**	RTX
**COMPARISON**	CNI, CTX
**MAIN OUTCOMES**	3-month or 12-month steroid withdrawal rate
**PERSPECTIVE**	Clinicians, social workers and parents of children with FRNS/SDNS
**SETTING**	Hospital
**CONFLICT OF INTERETS**	No

ASSESSMENT

1. PROBLEM: Is the problem a priority?

Detailed judgments: The more serious or urgent a problem is, the more likely it is that an option that addresses the problem will be a priority.

与其他免疫抑制剂相比 RTX 治疗 FRNS/SDNS 随访 3 和 12 个月激素停用问题具有优先性么？根据问题的严重性和紧急性程度判断其是否具有优先性。严重性和紧急性程度越高，优先性越大

JUDGEMENTS	RESEARCH EVIDENCE	ADDITIONAL CONSIDERATIONS
☐ No<br>☐ Probably no<br>☐ Probably yes<br>☑ **Yes**<br>☐ Varies<br>☐ Uncertain	/	Vote Results： **14/14 chose "Yes"**.

2. Desirable effects: How substantial are the desirable anticipated effects?

Detailed judgments: How large are the desirable effects of the intervention taking into account the importance of the outcomes (how much they are valued), and the size of the effect (the

likelihood of experiencing a benefit or how much of an improvement individuals would be likely to experience)?
与其他免疫抑制剂相比 RTX 治疗 FRNS/SDNS 随访 3 和 12 个月激素停用获益的有益影响有多大？根据结局重要性（受重视的程度）和效应值大小（获益的可能性和个体情况改善的程度）对干预措施的有益影响进行判断

JUDGEMENTS

- ☐ Tivial
- ☐ Sall
- ☐ Moderate
- ☑ **Large**
- ☐ Varies
- ☐ Uncertain

RESEARCH EVIDENCE

结局；患儿数量<br>研究类型和数量	相对效应（95%CI）	预期绝对效应值（事件数/例数）			证据质量	升降级说明
		对照组	干预组	风险差		
激素停用的时间						
**患者分组：FRNS/SDNS(SDNS 合并 CNI 依赖至少 1 年[338]，仅激素合并或不合并左旋咪唑[226])；干预：利妥昔单抗；对照：CNI 和 CTX，1 篇 RCT 研究[338]和 1 篇 NRSI 研究[226],100 例**						
随访 3 月停用率（总体随访 12 月，3 月后部分对照改为 RTX，338)	**FRNS /SDNS：**<br>（35/46；10/54）<br>OR：14.2（5.25~38.40）	2/27，7.4%停用[338]，<br>8/27，29.6%停用[226]	21/27，77.8%停用[338]，<br>14/19，73.7%停用[226]	**FRNS /SDNS：**<br>每 1000 人增加 578 人<br>（359~712）	**极低**	**降级因素：**<br>偏倚风险降 2 级<br>不一致性不降级<br>精确性降 2 级<br>发表偏倚不适用<br>间接性不降级
**患者分组：FRNS/SDNS+(SDNS 合并 CNI 依赖至少 2 年)；干预：利妥昔单抗；对照：CNI，1 篇 RCT 研究[30],51 例**						
随访 12 月<br>无激素时长 Drug-free period, d/y	/	16 人，80.2±98.5，<br>入组前一年 142.9 ± 117.7	35 人（1 人无资料），<br>140.5±91.3<br>入组前一年 99.1±111.8	p=0.12<br>p=0.17	**极低**	**降级因素：**<br>偏倚风险降 1 级<br>不一致性不适用<br>精确性降 2 级<br>发表偏倚不适用<br>间接性不降级
**人群：FRNS/SDNS(仅激素治疗后 SDNS [54],部分仅单独激素，部分 CNI 或 MMF 其他免疫抑制剂使用后[440])； 干预组措施：利妥昔单抗；对照组措施：CTX 或 CNI, 1 篇 RCT[54], 1 篇队列研究[440],238 例**						
随访 12 月停用率，其中范围 12-90 月	**FRNS /SDNS：**<br>（91/101；113/137）<br>OR：1.88 (0.86~4.11)	46/58, 79.3%停用[54],67/79 (84.8%)可停用，停用中位时间为 3 月，停用后首次复发中位时间为 3 个月，12/79 人复发时仍激素使用中[440]	55/59, 93.2%停用[54],36/42 (85.7%)可停用，停用中位时间为 3 月，停用后首次复发中位时间为 12 个月，6/42 人复发时仍激素使用中[440]	**FRNS /SDNS：**<br>每 1000 人增加 74 人（减少 23，增加 126）	**极低**	**降级因素：**<br>偏倚风险降 2 级<br>不一致性不降级<br>精确性降 2 级<br>发表偏倚不适用<br>间接性不降级
**人群：FRNS/SDNS（部分利妥昔单抗合并或不合并 CNI，部分单独 CNI）；干预组：利妥昔单抗合并或不合并 CNI；对照组：CNI；1 篇队列研究[199]，18 例**						
至少 12 月，部分 24 月<br>无激素时长 mean ± SD	/	8 人，3.20 ± 3.00 月	10 人，7.30 ± 7.40 月	p=0.2	**极低**	**降级因素：**<br>偏倚风险降 2 级<br>不一致性不适用<br>精确性降 2 级<br>发表偏倚不适用<br>间接性不降级
**人群：FRNS/SDNS+；干预组：利妥昔单抗后 MMF；对照组：RTX 后加用 MMF 安慰剂；1 篇 RCT[194]，57 例**						
随访至少 16.8 个月中位数非治疗失败（天）	/	21/29 治疗失败，删失 8，中位数非治疗失败时间 402（327-574）d	18/28 治疗失败，删失 10，中位数非治疗失败时间 754（543-997）d	HR 0.55 (0.31-0.98)	**中**	**降级因素：**<br>偏倚风险不降级<br>不一致性不适用<br>精确性降 1 级<br>发表偏倚不适用<br>间接性不降级

ADDITIONAL CONSIDERATIONS

Vote Results : **14/14 chose “Large”.**

结局	研究数量和类型	事件数/总数	率（95%CI）	证据质量
激素停用的时间				
**患者分组：FRNS/SDNS+；干预：利妥昔单抗+； 1 篇队列研究[196]，17 例**				
3 个月停用激素比例	1 篇队列研究[196]	17/17	/	/
**患者分组：FRNS/SDNS+；干预：利妥昔单抗+； 2 篇队列研究[339，196]和 2 篇病例系列报告[124]，70 例**				
6 个月停用激素比例	2 篇队列研究[339，196]<br>2 篇病例系列报告[124]	25/46 人(54%) [339]<br>2/7（29%）[124]<br>17/17 (100%)[196]	68%（95%CI:56~79）	
**患者分组：FRNS/SDNS+；干预：利妥昔单抗+； 1 篇病例系列报告[219]，81 例**				
停用激素时间随访 13–90 月	1 篇病例系列报告[219]	69/81（85%）<br>中位 66.5（26-409）天	/	/
**人群：FRNS/SDNS（SDNSke 合并左旋咪唑，CTX 和/或 MMF 和或 CNI）；干预：利妥昔单抗+，1 项队列研究[379]，101 例**				
随访平均 30.6 ± 19.1 (15–43)月，末次有效随访时间 22 (14–39)月	1 篇队列研究[379]	/	90/101 在 4.8 ± 2.0 月后停用	/
**患者分组：FRNS/SDNS+；干预：利妥昔单抗 1 项病例报道[230]，37 例**				
随访中位 29.4 (范围 9.2–92.8)月，Mean (SD) 32.7 (15.1)	1 篇病例报道[230]	/	24/37 复发，35/37 (94.5%) 在 1.3 (0.37-6) 月停用	/

Refer to Meta Figure15, Figure 16, Figure17

3. Undesirable effects: How substantial are the undesirable anticipated effects?

Detailed judgments: How large are the desirable effects of the intervention taking into account the importance of the outcomes (how much they are valued), and the size of the effect (the likelihood of experiencing a benefit or how much of an improvement individuals would be likely to experience)?

与其他免疫抑制剂相比 RTX 治疗 FRNS/SDNS 随访 3 和 12 个月激素停用获益的不良影响有多大？根据结局重要性（受重视的程度）和效应值大小（获益的可能性和个体情况改善的程度）对干预措施的不良影响进行判断

JUDGEMENTS	RESEARCH EVIDENCE	ADDITIONAL CONSIDERATIONS
□ Large<br>□ Moderate<br>☑ **Small**<br>□ trivial<br>□ Varies<br>□ Uncertain	/	Vote Results：1/14 chose "Large", **8/14 chose "Small"**, 5/14 chose "Trivial".

4. Certainty of the evidence: What is the overall certainty of the evidence of effects?

Detailed judgments: How good an indication does the research provide of the likely effects across all of the critical outcomes; i.e. the likelihood that the effects will be different enough from what the research found that it might affect a decision about the intervention?

与其他免疫抑制剂相比 RTX 治疗 FRNS/SDNS 随访 3 和 12 个月激素停用获益有益影响和不良影响相关证据的总体质量？基于与其他免疫抑制剂相比 RTX 治疗 FRNS/SDNS 随访 3 和 12 个月激素停用获益可能影响，判断干预效果是否会对干预决策产生影响

JUDGEMENTS	RESEARCH EVIDENCE	ADDITIONAL CONSIDERATIONS
☑ **Very low**<br>□ Low<br>□ Moderate	/	Vote Results：**7/14 chose "Very low"**, 6/14 chose "Low", 1/14 chose "Moderate".

□ High<br>□ No included studies		

5. Values: Is there important uncertainty about or variability in how much people value the main outcomes?

Detailed judgments: How much do individuals value each of the main outcomes? Is uncertainty about how much they value each of the outcomes or variability in how much different individual value the outcomes large enough that it could lead to different decisions?
对与其他免疫抑制剂相比 RTX 治疗 FRNS/SDNS 随访 3 和 12 个月激素停用获益的重视程度，是否因个体不同而存在不确定性和变化性？个体对与其他免疫抑制剂相比 RTX 治疗 FRNS/SDNS 随访 3 和 12 个月激素停用获益的重视程度有多大？不确定性和变化性是否会导致不同的决策？不确定性体现在对上述推荐的理解程度；变化性体现在对上述推荐重视程度的差异。

JUDGEMENTS	RESEARCH EVIDENCE	ADDITIONAL CONSIDERATIONS
□ Important uncertainty or variability<br>□ Possibly important uncertainty or variability<br>□ Probably no important uncertainty or variability<br>☑ **No Important uncertainty or variability**	/	Vote Results : 1/14 chose "Probably no Important uncertainty or variability", **13/14 chose "No Important uncertainty or variability".**

6. Balance of effects: Does the balance between desirable and undesirable effects favour the intervention or the comparison?

Detailed judgments: What is the balance between the desirable and undesirable effects, taking into account how much individuals value the main outcome, how substantial the desirable and undesirable effects are, the certainty of those estimates, discount rates, risk aversion and risk seeking?
权衡与其他免疫抑制剂相比 RTX 治疗 FRNS/SDNS 随访 3 和 12 个月激素停用获益利弊后更倾向于干预组还是对照组？从以下方面权衡利弊：个体对上述推荐的重视程度；利多大？；弊多大？；估计值的精确性；信心有多大？；风险多大？；可能规避风险么？

JUDGEMENTS	RESEARCH EVIDENCE	ADDITIONAL CONSIDERATIONS
□ Favours the comparison<br>□ Probably favours the comparison<br>□ Does not favour either the intervention or the comparison<br>□ Probably favours the intervention<br>☑ **Favours the intervention**<br>□ Varies<br>□ Uncertain	/	Vote Results : 4/14 chose "Probably favours the intervention", **10/14 chose "Favours the intervention".**

7. Resources required: How large are the resource requirements (costs)?

Detailed judgments: How large is the cost of the difference in resource use between the intervention and comparision?

与其他免疫抑制剂相比 RTX 治疗 FRNS/SDNS 随访 3 和 12 个月激素停用获益成本支出有多大？与其他免疫抑制剂相比 RTX 治疗 FRNS/SDNS 随访 3 和 12 个月激素停用获益的支出成本差异有多大？

JUDGEMENTS	RESEARCH EVIDENCE	ADDITIONAL CONSIDERATIONS
□ Large costs<br>□ Moderate costs<br>□ Negligible costs or savings<br>□ Moderate savings<br>□ Large savings<br>□ Varies<br>☑ **Don' t know**	Refer to recommendation 18	Vote Results : 6/14 chose " Negligible costs or savings " , 1/14 chose "Large savings", **7/14 chose "Don' t know" .**

8. Certainty of evidence of required resources: What is the certainty of the evidence of resource requirements (costs)?

Detailed judgments: How certain is the evidence of a difference for each type of resource use (eg. drugs, hospitalizations) and the cost of resources?

成本支出的证据质量如何？对与其他免疫抑制剂相比 RTX 治疗 FRNS/SDNS 随访 3 和 12 个月激素停用获益成本支出（包括药物、住院等费用）相关证据的确定性。

JUDGEMENTS	RESEARCH EVIDENCE	ADDITIONAL CONSIDERATIONS
☑ **Very low**<br>□ Low<br>□ Moderate<br>□ High<br>□ No included studies	Refer to recommendation 18	Vote Results : 3/14 chose "Very low" , **5/14 chose " Low " ,** 1/14 chose " Moderate " , **5/14 chose "No included studies" .**<br><br>Referring to recommendation 18, the quality of evidence is very low。

9. Cost-effectiveness: Does the cost-effectiveness of the intervention favour the intervention or the comparison?

Detailed judgments: Is the intervention cost-effective, taking into account uncertainty about or variability in the costs, uncertainty about or variability in the net benefit, sensitivity analyses, and the reliability and applicability of the economic evaluation?

对与其他免疫抑制剂相比 RTX 治疗 FRNS/SDNS 随访 3 和 12 个月激素停用获益干预的成本效益分析更倾向于干预组还是对照组？从以下方面分析干预的成本效益：对支出成本的不确定性或变化性；对净利润的不确定性或变化性；敏感性分析；经济评估的可靠性和适用性。

JUDGEMENTS	RESEARCH EVIDENCE	ADDITIONAL CONSIDERATIONS

□ Favours the comparison<br>□ Probably favours the comparison<br>□ Does not favour either the intervention or the comparison<br>□ Probably favours the intervention<br>☑ **Favours the intervention**<br>□ Varies<br>□ No included studies	Refer to recommendation 18	Vote Results : 1/14 chose " Probably favours the intervention " , **12/14 chose "Favours the intervention",** 1/14 chose "No included studies" .

10. EQUITY: What would be the impact on health equity?

Detailed judgments: Are there plausible reasons for anticipating differences in the relative effectiveness of the intervention for disadvantaged subgroups or different baseline conditions across disadvantaged subgroups that affect the absolute effectiveness of the intervention or the importance of the problem?

对卫生公平性的影响？与其他免疫抑制剂相比 RTX 治疗 FRNS/SDNS 随访 3 和 12 个月激素停用获益的相对有效性是否在弱势人群中有所降低，对此是否有合理的解释？弱势人群的不同基线水平会影响干预的绝对有效性或研究问题的重要性，对此是否有合理的解释？

JUDGEMENTS	RESEARCH EVIDENCE	ADDITIONAL CONSIDERATIONS
□ Reduced<br>☑ **Probably Reduced**<br>□ Probably no impact<br>□ Probably increased<br>□ Increased<br>□ Varies<br>□ Uncertain	/	Vote Results : **9/14 chose "Probably Reduced"**, 3/14 chose " Probably no impact " , 1/14 chose " Increased " , 1/14 chose "Varies" .

11. ACCEPTABILITY: Is the intervention acceptable to key stakeholders?

Detailed judgments: Are key stakeholders likely not to accept the distribution of the benefits, harms and costs; or the costs or undesirable effects in the short term for desirable effects (benefits) in the future? Are they likely to disagree with the values attached to the desirable or undesirable effects, or not to accept the diagnostic intervention because of ethical concerns?

患儿及其家长是否接受与其他免疫抑制剂相比 RTX 治疗 FRNS/SDNS 随访 3 和 12 个月激素停用获益？患儿及其家长是否会接受干预带来的获益、伤害及支出，或远期获益带来的短期内的不良反应；是否会对利弊判断背后的价值观念持反对意见；是否会出于伦理考虑拒绝诊断性治疗。

JUDGEMENTS	RESEARCH EVIDENCE	ADDITIONAL CONSIDERATIONS
□ No<br>□ Probably no<br>□ Probably yes<br>☑ **Yes**	/	Vote Results : 4/14 chose " Probably Yes " , **10/14 chose "Yes" .**

☐ Varies<br>☐ Uncertain		

12. FEASABILITY: Is the option feasible to implement?

Detailed judgments: Is it feasible to sustain use of the intervention and to address potential barriers to using it?

JUDGEMENTS	RESEARCH EVIDENCE	ADDITIONAL CONSIDERATIONS
☐ No<br>☐ Probably no<br>☐ Probably yes<br>☑ **Yes**<br>☐ Varies<br>☐ Uncertain	/	Vote Results: **14/14 chose "Yes".**

SUMMARY OF JUDGEMENTS

CRITERIA	DECISION							
1. PROBLEM	No		Probably no	Probably Yes		**Yes**	Varies	Don’t know
2. BENEFITS	Trivial		Small	Moderate		**Large**	Varies	Don’t know
3. HARMS	Large		Moderate	**Small**		Trivial	Varies	Don’t know
4. QUALITY OF EVIDENCE	**Very low**		Low	Moderate		High	No included studies	
5. VALUES	Important uncertainty or variability		Probably Important uncertainty or variability	Probably no Important uncertainty or variability		**No Important uncertainty or variability**	Varies	
6. BALANCE OF EFFECTS	Favours the comparison	Probably favours the comparison	Does not favour either the intervention or the comparison		Probably favours the intervention	**Favours the intervention**	Varies	Don’t know
7. RESOURCES REQUIRED	Large costs	Moderate costs	Negligible costs or savings		Large savings	Moderate savings	Varies	**Don’t know**
8. CERTAINTY OF EVIDENCE OF REQUIRED RESOURCES	**Very low**	Low	Moderate		High		No included studies	
9. COST-EFFECTIVENESS	Favours the comparison	Probably favours the comparison	Does not favour either the intervention or the comparison		Probably favours the intervention	**Favours the intervention**	Varies	Don’t know
10. EQUITY	Reduced	**Probably Reduced**	Probably no impact		Probably Increased	Increased	Varies	Don’t know
11. ACCEPTABILITY	No	Probably no	Probably Yes		**Yes**		Varies	Don’t know
12. FEASIBILITY	No	Probably no	Probably Yes		**Yes**		Varies	Don’t know

TYPE OF RECOMMENDATION

Strong recommendation against the intervention	Conditional recommendation against the intervention	Conditional recommendation for either the intervention or the comparison	Conditional recommendation for the intervention	Strong recommendation for the intervention
○	○	○	○	○

CONCLUSIONS

Reason for recommendation

Steroid withdrawal is an important factor to consider.

Recommendation(text)

RTX treatment benefits on the time to steroid withdrawal in children with FRNS/SDNS compared with other immunosuppressants. (1D)

Implementation considerations

1. There is no evidence on 1-month and 6-month steroid withdrawal rate;
2. It is uncertain that steroid withdrawal is out of study protocol or actual situation.

Recommendation 7: RTX + MMF is recommended for the treatment of children with FRNS/SDNS in consideration of delayed time to first relapse and reduced steroid dose by about 0.18 mg•kg-1•d-1. (1D)

Does consolidation therapy improve clinical outcomes in children aged 1-18 years with SSNS after RTX treatment?	
**STUDY TYPE**	Two-arm study
**POPULATION**	FRNS/SDNS、FRNS/SDNS+
**INTERVENTION**	RTX+MMF
**COMPARISON**	RTX, RTX+CsA, RTX+placebo
**MAIN OUTCOMES**	The time to first relapse
**PERSPECTIVE**	Clinicians, social workers and parents of children with FRNS/SDNS
**SETTING**	Hospital
**CONFLICT OF INTERESTS**	No

ASSESSMENT

1. PROBLEM: Is the problem a priority?

Detailed judgments: The more serious or urgent a problem is, the more likely it is that an option that addresses the problem will be a priority.
FRNS/SDNS 患儿利妥昔单抗后加 MMF 能从延长首次复发时间中获益问题具有优先性么？根据问题的严重性和紧急性程度判断其是否具有优先性。严重性和紧急性程度越高，优先性越大

JUDGEMENTS	RESEARCH EVIDENCE	ADDITIONAL CONSIDERATIONS
☐ No<br>☐ Probably no<br>☐ Probably yes<br>☑ **Yes**<br>☐ Varies<br>☐ Uncertain		Vote Results : 2/14 chose "Probably Yes", **12/14 chose "Yes"** .

2. Desirable effects: How substantial are the desirable anticipated effects?

Detailed judgments: How large are the desirable effects of the intervention taking into account the importance of the outcomes (how much they are valued), and the size of the effect (the likelihood of experiencing a benefit or how much of an improvement individuals would be likely to experience)?

FRNS/SDNS 患儿利妥昔单抗后加 MMF 能从延长首次复发时间中获益的有益影响有多大？根据结局重要性（受重视的程度）和效应值大小（获益的可能性和个体情况改善的程度）对干预措施的有益影响进行判断

JUDGEMENTS

- ☐ trivial
- ☐ small
- ☐ Moderate
- ☑ Large
- ☐ Varies
- ☐ Uncertain

RESEARCH EVIDENCE

结局；患儿数量<br>研究类型和数量	相对效应（95%CI）	预期绝对效应值（事件数/例数）<br>对照组	干预组	风险差	证据质量	升降级说明
利妥昔单抗后加 MMF 随访时间≥12 个月复发或缓解						
**人群：FRNS/SDNS；干预组：利妥昔单抗+MMF；对照组：利妥昔单抗和/或安慰剂；利妥昔单抗（375mg/m² qw×4w）[194]，利妥昔单抗（375mg/m² qw×1~2w）[21]；1 篇 RCT[194]，1 篇病例系列报道[21]；干预组 60 例，对照组 52 例**						
首次复发时间	首次中位复发时间比（MSR）=3.15，95%CI:1.31~7.58，P=0.01，异性检验 Q=13.59 P<0·000	27/39 例复发[194]，首次复发时间 320 (266，1060)d<br>11/13 例复发[21]，首次复发时间 60（27，130）d	11/39 复发[194]，首次复发时间 654 (500，858)d<br>13/21 例复发[21]，首次复发时间 450（332，672）d	/	极低	**降级因素：**<br>偏倚风险降 2 级<br>不一致性不降级<br>精确性降 1 级<br>发表偏倚不适用<br>间接性不降级<br>**升级因素：无**
**人群：FRNS/SDNS+；干预组：利妥昔单抗（375mg/m²）+MMF；对照组：利妥昔单抗，1 篇非 RCT[201]，干预组 9 例，对照组 7 例**						
年复发次数	/	6/7 例复发，2.3 次/年	3/9 例复发，0.42 次/年	P＜0.001	极低	**降级因素：**<br>偏倚风险降 2 级<br>不一致性不适用<br>精确性降 1 级<br>发表偏倚不适用<br>间接性不降级<br>**升级因素：无**
**人群：FRNS/SDNS+；干预组：利妥昔单抗+MMF，对照组：利妥昔单抗+CsA 或安慰剂（停免疫抑制剂）；1 篇 RCT[194]，2 篇非 RCT[135,142]，1 篇队列研究[135]和 1 篇病例系列报道[21]，干预组 87 例，对照组 80 例**						
复发率	**RTX+CsA 或 RTX+安慰剂（停免疫抑制剂）**（34/87；50/77）：<br>OR=0.25，95%CI：0.06~1.07<br>**RTX+CsA**<br>（7/18；3/18）：<br>OR=2.89，95%CI：0.59~14.11<br>**RTX+安慰剂或停免疫抑制剂：**<br>（27/71；47/62）<br>OR=0.16，95%CI：0.07~0.35	27/39 例复发（安慰剂）[194]<br>6/7 例复发（空白）[201]<br>11/13 例复发（空白）[21]，<br>2/13 例复发（CsA）[142]，<br>3/3 例复发（空白）[135]<br>1/5 例复发（CsA）[135]	11/39 复发[194]<br>3/9 例复发[201]<br>13/21 复发[21]<br>7/16 复发[142]<br>0/2 例复发[135]	**RTX+CsA 或 RTX+安慰剂（停免疫抑制剂）：**<br>每 1000 人减少 333 人（减少 549，增加 15）<br>**RTX+CsA：**<br>每 1000 人增加 200 人（减少 61，增加 572）<br>**RTX+安慰剂或停免疫抑制剂：**	极低	**降级因素：**<br>偏倚风险降 2 级<br>不一致性不降级<br>精确性降 2 级<br>发表偏倚不适用<br>间接性不降级<br>**升级因素：无**
				每 1000 人减少 424 人（235，578）		

Refer to Meta Figure 18, Figure 19

ADDITIONAL CONSIDERATIONS

Vote Results : 1/14 chose " Moderate " , **13/14 chose "Large"** .

3. Undesirable effects: How substantial are the undesirable anticipated effects?

Detailed judgments: How large are the desirable effects of the intervention taking into account the importance of the outcomes (how much they are valued), and the size of the effect (the likelihood of experiencing a benefit or how much of an improvement individuals would be likely to experience)?

FRNS/SDNS 患儿利妥昔单抗后加 MMF 能从延长首次复发时间中获益的不良影响有多大？根据结局重要性（受重视的程度）和效应值大小（获益的可能性和个体情况改善的程度）对干预措施的不良影响进行判断

JUDGEMENTS	RESEARCH EVIDENCE	ADDITIONAL CONSIDERATIONS
□ Large<br>□ Moderate<br>☑ **Small**<br>☑ **Trivial**<br>□ Varies<br>□ Uncertain		Vote Results : **7/14 chose "Small", 7/14 chose "Trivial" .**<br><br>Experts voted even, but the overall trend was "small" .

4. Certainty of the evidence: What is the overall certainty of the evidence of effects?

Detailed judgments: How good an indication does the research provide of the likely effects across all of the critical outcomes; i.e. the likelihood that the effects will be different enough from what the research found that it might affect a decision about the intervention?

FRNS/SDNS 患儿利妥昔单抗后加 MMF 能从延长首次复发时间中获益有益影响和不良影响相关证据的总体质量？基于 FRNS/SDNS 患儿利妥昔单抗后加 MMF 能从延长首次复发时间中获益的可能影响，判断干预效果是否会对干预决策产生影响

JUDGEMENTS	RESEARCH EVIDENCE	ADDITIONAL CONSIDERATIONS
☑ **Very low**<br>□ Low<br>□ Moderate<br>□ High<br>□ No included studies		Vote Results: **11/14 chose "Very low",** 3/14 chose "Low" .

5. Values: Is there important uncertainty about or variability in how much people value the main outcomes?

Detailed judgments: How much do individuals value each of the main outcomes? Is uncertainty about how much they value each of the outcomes or variability in how much different individual value the outcomes large enough that it could lead to different decisions?

对 FRNS/SDNS 患儿利妥昔单抗后加 MMF 能从延长首次复发时间中获益的重视程度，是否因个体不同而存在不确定性和变化性？个体对 FRNS/SDNS 患儿利妥昔单抗后加 MMF 能从延长首次复发时间中获益的重视程度有多大？不确定性和变化性是否会导致不同的决策？不确定性体现在对上述推荐的理解程度；变化性体现在对上述推荐重视程度的差异。

JUDGEMENTS	RESEARCH EVIDENCE	ADDITIONAL CONSIDERATIONS
□ Important uncertainty or variability<br>□ Possibly important uncertainty or variability<br>□ Probably no important uncertainty or variability<br>☑ **No Important uncertainty or variability**		Vote Results : 1/14 chose " Probably no Important uncertainty or variability", **13/14 chose " No Important uncertainty or variability" .**

6. Balance of effects: Does the balance between desirable and undesirable effects favour the intervention or the comparison?

Detailed judgments: What is the balance between the desirable and undesirable effects, taking into account how much individuals value the main outcome, how substantial the desirable and undesirable effects are, the certainty of those estimates, discount rates, risk aversion and risk seeking?

权衡 FRNS/SDNS 患儿利妥昔单抗后加 MMF 能从延长首次复发时间中获益利弊后更倾向于干预组还是对照组？从以下方面权衡利弊：个体对上述推荐的重视程度；利多大？；弊多大？；估计值的精确性；信心有多大？；风险多大？；可能规避风险么？

JUDGEMENTS	RESEARCH EVIDENCE	ADDITIONAL CONSIDERATIONS
□ Favours the comparison<br>□ Probably favours the comparison<br>□ Does not favour either the intervention or the comparison<br>□ Probably favours the intervention<br>☑ **Favours the intervention**<br>□ Varies<br>□ Uncertain		Vote Results : 4/14 chose "Probably favours the intervention", **10/14 chose "Favours the intervention"**.

7. Resources required: How large are the resource requirements (costs)?

Detailed judgments: How large is the cost of the difference in resource use between the intervention and comparision?

FRNS/SDNS 患儿利妥昔单抗后加 MMF 能从延长首次复发时间中获益成本支出有多大？干预组和对照组的支出成本差异有多大？

JUDGEMENTS	RESEARCH EVIDENCE	ADDITIONAL CONSIDERATIONS
□ Large costs<br>□ Moderate costs<br>☑ **Negligible costs or savings**<br>□ Moderate savings<br>□ Large savings<br>□ Varies<br>□ Uncertain	Refer to recommendation 18	Vote Results : **8/14 chose "Negligible costs or savings"**, 2/14 chose "Moderate savings", 4/14 chose "Don't know".

8. Certainty of evidence of required resources: What is the certainty of the evidence of resource requirements (costs)?

Detailed judgments: How certain is the evidence of a difference for each type of resource use (eg. drugs, hospitalizations) and the cost of resources?<br>成本支出的证据质量如何？对 FRNS/SDNS 患儿利妥昔单抗后加 MMF 能从延长首次复发时间中获益成本支出（包括药物、住院等费用）相关证据的确定性。		
**JUDGEMENTS**	**RESEARCH EVIDENCE**	**ADDITIONAL CONSIDERATIONS**
□ Very low<br>□ Low<br>□ Moderate<br>□ High<br>☑ **No included studies**	Refer to recommendation 18	Vote Results： 4/14 chose “Very low”， 4/14 chose “Low”， 1/14 chose “Moderate”， **5/14 chose “No included studies”．**

9. Cost-effectiveness: Does the cost-effectiveness of the intervention favour the intervention or the comparison?

Detailed judgments: Is the intervention cost-effective, taking into account uncertainty about or variability in the costs, uncertainty about or variability in the net benefit, sensitivity analyses, and the reliability and applicability of the economic evaluation?

对 FRNS/SDNS 患儿利妥昔单抗后加 MMF 能从延长首次复发时间中获益干预的成本效益分析更倾向于干预组还是对照组？从以下方面分析干预的成本效益：对支出成本的不确定性或变化性；对净利润的不确定性或变化性；敏感性分析；经济评估的可靠性和适用性。

**JUDGEMENTS**	**RESEARCH EVIDENCE**	**ADDITIONAL CONSIDERATIONS**
□ Favours the comparison<br>□ Probably favours the comparison<br>□ Does not favour either the intervention or the comparison<br>□ Probably favours the intervention<br>☑ **Favours the intervention**<br>□ Varies<br>□ No included studies	Refer to recommendation 18	Vote Results： 2/14 chose “Probably favours the intervention”， **10/14 chose “Favours the intervention”，** 2/14 chose “No included studies”．

10. EQUITY: What would be the impact on health equity?

Detailed judgments: Are there plausible reasons for anticipating differences in the relative effectiveness of the intervention for disadvantaged subgroups or different baseline conditions across disadvantaged subgroups that affect the absolute effectiveness of the intervention or the importance of the problem?

对卫生公平性的影响？FRNS/SDNS 患儿利妥昔单抗后加 MMF 能从延长首次复发时间中获益的相对有效性是否在弱势人群中有所降低，对此是否有合理的解释？弱势人群的不同基线水平会影响干预的绝对有效性或研究问题的重要性，对此是否有合理的解释？

**JUDGEMENTS**	**RESEARCH EVIDENCE**	**ADDITIONAL CONSIDERATIONS**

☐ Reduced<br>☑ **Probably Reduced**<br>☐ Probably no impact<br>☐ Probably increased<br>☐ Increased<br>☐ Varies<br>☐ Uncertain	/	Vote Results : **9/14 chose "Probably Reduced",** 3/14 chose "Probably no impact", 1/14 chose "Probably increased", 1/14 chose "Don't know".

11. ACCEPTABILITY: Is the intervention acceptable to key stakeholders?

Detailed judgments: Are key stakeholders likely not to accept the distribution of the benefits, harms and costs; or the costs or undesirable effects in the short term for desirable effects (benefits) in the future? Are they likely to disagree with the values attached to the desirable or undesirable effects, or not to accept the diagnostic intervention because of ethical concerns? 患儿及其家长是否接受 FRNS/SDNS 患儿利妥昔单抗后加 MMF 能从延长首次复发时间中获益？患儿及其家长者是否会接受干预带来的获益、伤害及支出，或远期获益带来的短期内的不良反应；是否会对利弊判断背后的价值观念持反对意见；是否会出于伦理考虑拒绝诊断性治疗。

JUDGEMENTS	RESEARCH EVIDENCE	ADDITIONAL CONSIDERATIONS
☐ No<br>☐ Probably no<br>☐ Probably yes<br>☑ **Yes**<br>☐ Varies<br>☐ Uncertain	/	Vote Results : 3/14 chose "Probably Yes", **11/14 chose "Yes".**

12. FEASABILITY: Is the option feasible to implement?

Detailed judgments: Is it feasible to sustain use of the intervention and to address potential barriers to using it?

JUDGEMENTS	RESEARCH EVIDENCE	ADDITIONAL CONSIDERATIONS
☐ No<br>☐ Probably no<br>☐ Probably yes<br>☑ **Yes**<br>☐ Varies<br>☐ Uncertain	/	Vote Results : 1/14 chose "Probably Yes", **13/14 chose "Yes".**

SUMMARY OF JUDGEMENTS

<table>
<tr><th>CRITERIA</th><th colspan="8">DECISION</th></tr>
<tr><td>1. PROBLEM</td><td colspan="2">No</td><td>Probably no</td><td colspan="2">Probably Yes</td><td>Yes</td><td>Varies</td><td>Don’t know</td></tr>
<tr><td>2. BENEFITS</td><td colspan="2">Trivial</td><td>Small</td><td colspan="2">Moderate</td><td>Large</td><td>Varies</td><td>Don’t know</td></tr>
<tr><td>3. HARMS</td><td colspan="2">Large</td><td>Moderate</td><td colspan="2">Small</td><td>Trivial</td><td>Varies</td><td>Don’t know</td></tr>
<tr><td>4. QUALITY OF EVIDENCE</td><td colspan="2">Very low</td><td>Low</td><td colspan="2">Moderate</td><td>High</td><td colspan="2">No included studies</td></tr>
<tr><td>5. VALUES</td><td colspan="2">Important uncertainty or variability</td><td>Probably Important uncertainty or variability</td><td colspan="2">Probably no Important uncertainty or variability</td><td>No Important uncertainty or variability</td><td colspan="2">Varies</td></tr>
<tr><td>6. BALANCE OF EFFECTS</td><td>Favours the comparison</td><td>Probably favours the comparison</td><td colspan="2">Does not favour either the intervention or the comparison</td><td>Probably favours the intervention</td><td>Favours the intervention</td><td>Varies</td><td>Don’t know</td></tr>
<tr><td>7. RESOURCES REQUIRED</td><td>Large costs</td><td>Moderate costs</td><td colspan="2">Negligible costs or savings</td><td>Large savings</td><td>Moderate savings</td><td>Varies</td><td>Don’t know</td></tr>
<tr><td>8. CERTAINTY OF EVIDENCE OF REQUIRED RESOURCES</td><td>Very low</td><td>Low</td><td colspan="2">Moderate</td><td colspan="2">High</td><td colspan="2">No included studies</td></tr>
<tr><td>9. COST-EFFECTIVENESS</td><td>Favours the comparison</td><td>Probably favours the comparison</td><td colspan="2">Does not favour either the intervention or the comparison</td><td>Probably favours the intervention</td><td>Favours the intervention</td><td>Varies</td><td>Don’t know</td></tr>
<tr><td>10. EQUITY</td><td>Reduced</td><td>Probably Reduced</td><td colspan="2">Probably no impact</td><td>Probably Increased</td><td>Increased</td><td>Varies</td><td>Don’t know</td></tr>
<tr><td>11. ACCEPTABILITY</td><td>No</td><td>Probably no</td><td colspan="2">Probably Yes</td><td colspan="2">Yes</td><td>Varies</td><td>Don’t know</td></tr>
<tr><td>12. FEASIBILITY</td><td>No</td><td>Probably no</td><td colspan="2">Probably Yes</td><td colspan="2">Yes</td><td>Varies</td><td>Don’t know</td></tr>
</table>

TYPE OF RECOMMENDATION

Strong recommendation against the intervention	Conditional recommendation against the intervention	Conditional recommendation for either the intervention or the comparison	Conditional recommendation for the intervention	Strong recommendation for the intervention
○	○	○	○	○

CONCLUSIONS

Recommendation(text)

RTX + MMF is recommended for the treatment of children with FRNS/SDNS in consideration of delayed time to first relapse and reduced steroid dose by about 0.18 mg·kg-1·d-1. (1D)

Implementation considerations

Limited sample size.

Research priorities

Study on specific prolonged time.

Recommendation 8: Re-exposure to RTX could decrease the relapse rate in children with FRNS/SDNS after RTX treatment. (1D)

Does consolidation therapy improve clinical outcomes in children aged 1-18 years with SSNS after RTX treatment?	
STUDY TYPE	RCT
POPULATION	FRNS/SDNS+
INTERVENTION	Second course of RTX treatment given at B cell reconstitution (peripheral CD19+B cells ＞ 1% of total lymphocytes)
COMPARISON	Single course of RTX treatment
MAIN OUTCOMES	50% relapse-free survival time
PERSPECTIVE	Clinicians, social workers and parents of children with FRNS/SDNS
SETTING	Hospital
CONFLICT OF INTERESTS	No

ASSESSMENT

1. PROBLEM: Is the problem a priority?<br>Detailed judgments: The more serious or urgent a problem is, the more likely it is that an option that addresses the problem will be a priority.<br>FRNS/SDNS+患儿利妥昔单抗后再予利妥昔巩固治疗 Probably 从无复发率中获益问题具有优先性么？根据问题的严重性和紧急性程度判断其是否具有优先性。严重性和紧急性程度越高，优先性越大		
**JUDGEMENTS**	**RESEARCH EVIDENCE**	**ADDITIONAL CONSIDERATIONS**
□ No<br>□ Probably no<br>□ Probably yes<br>☑ **Yes**<br>□ Varies<br>□ Uncertain		Vote Results : 2/14 chose " Moderate " , **12/14 chose "Yes" .**

2. Desirable effects: How substantial are the desirable anticipated effects?<br>Detailed judgments: How large are the desirable effects of the intervention taking into account the importance of the outcomes (how much they are valued), and the size of the effect (the

likelihood of experiencing a benefit or how much of an improvement individuals would be likely to experience)?
FRNS/SDNS+患儿利妥昔单抗后再予利妥昔巩固治疗 Probably 从无复发率中获益的有益影响有多大？根据结局重要性（受重视的程度）和效应值大小（获益的可能性和个体情况改善的程度）对干预措施的有益影响进行判断

JUDGEMENTS

- ☐ trivial
- ☐ small
- ☐ Moderate
- ☑ **Large**
- ☐ Varies
- ☐ Uncertain

RESEARCH EVIDENCE

结局；患儿数量 研究类型和数量	相对效应（95%CI）	预期绝对效应值（事件数/例数）			证据质量	升降级说明
		对照组	干预组	风险差		
利妥昔单抗后加 RTX 巩固治疗随访时间≥12 个月复发或缓解						
人群：FRNS/SDNS+；干预组：利妥昔单抗（375mg/m2）+重复 1 疗程 RTX（基线、RTX 后每 1~2 月检测 B 细胞，CD19+B 淋巴细胞>1%）；对照组：利妥昔单抗，1 项 RCT[309]，干预组 16 例，对照组 45 例，伴 IS						
随访>24 月	FRNS/SDNS+：<br>p = 0.001<br>p = 0.0005	42/45（93%）复发<br>50%无复发生存时间为 RTX 后 335 天	9/16（56%）复发<br>50%无复发生存时间为首剂后 954 天，第 2 剂后 667 天	/	/	/

ADDITIONAL CONSIDERATIONS

Vote Results：3/14 chose “Moderate”，**10/14 chose “Large”**，1/14 chose “Don’t know”.

3. Undesirable effects: How substantial are the undesirable anticipated effects?

Detailed judgments: How large are the desirable effects of the intervention taking into account the importance of the outcomes (how much they are valued), and the size of the effect (the likelihood of experiencing a benefit or how much of an improvement individuals would be likely to experience)?
FRNS/SDNS+患儿利妥昔单抗后再予利妥昔巩固治疗可能从无复发率中获益的不良影响有多大？根据结局重要性（受重视的程度）和效应值大小（获益的可能性和个体情况改善的程度）对干预措施的不良影响进行判断

JUDGEMENTS

- ☐ Large
- ☐ Moderate
- ☐ small
- ☑ **Trivial**
- ☐ Varies
- ☐ Uncertain

RESEARCH EVIDENCE

/

ADDITIONAL CONSIDERATIONS

Vote Results：1/14 chose “Moderate”，6/14 chose “Small”，**7/14 chose “Trivial”.**

4. Certainty of the evidence: What is the overall certainty of the evidence of effects?

Detailed judgments: How good an indication does the research provide of the likely effects across all of the critical outcomes; i.e. the likelihood that the effects will be different enough from what the research found that it might affect a decision about the intervention?
FRNS/SDNS+患儿利妥昔单抗后再予利妥昔巩固治疗可能从无复发率中获益有益影响和不良影响相关证据的总体质量？基于 FRNS/SDNS+患儿利妥昔单抗后再予利妥昔巩固治疗可能从无复发率中获益可能影响，判断干预效果是否会对干预决策产生影响

JUDGEMENTS

- ☑ **Very low**
- ☐ Low
- ☐ Moderate

RESEARCH EVIDENCE

/

ADDITIONAL CONSIDERATIONS

Vote Results：**13/14 chose “Very low”**，1/14 chose “Moderate”.

□ High<br>□ No included studies		

5. Values: Is there important uncertainty about or variability in how much people value the main outcomes?

Detailed judgments: How much do individuals value each of the main outcomes? Is uncertainty about how much they value each of the outcomes or variability in how much different individual value the outcomes large enough that it could lead to different decisions?

对 FRNS/SDNS+患儿利妥昔单抗后再予利妥昔巩固治疗可能从无复发率中获益的重视程度，是否因个体不同而存在不确定性和变化性？个体对 FRNS/SDNS+患儿利妥昔单抗后再予利妥昔巩固治疗可能从无复发率中获益的重视程度有多大？不确定性和变化性是否会导致不同的决策？不确定性体现在对上述推荐的理解程度；变化性体现在对上述推荐重视程度的差异。

JUDGEMENTS	RESEARCH EVIDENCE	ADDITIONAL CONSIDERATIONS
□ Important uncertainty or variability<br>□ Possibly important uncertainty or variability<br>□ Probably no important uncertainty or variability<br>☑ **No Important uncertainty or variability**	/	Vote Results : 1/14 chose " Probably no Important uncertainty or variability", **13/14 chose " No Important uncertainty or variability" .**

6. Balance of effects: Does the balance between desirable and undesirable effects favour the intervention or the comparison?

Detailed judgments: What is the balance between the desirable and undesirable effects, taking into account how much individuals value the main outcome, how substantial the desirable and undesirable effects are, the certainty of those estimates, discount rates, risk aversion and risk seeking?

权衡 FRNS/SDNS+患儿利妥昔单抗后再予利妥昔巩固治疗可能从无复发率中获益利弊后更倾向于还是对照组？从以下方面权衡利弊：个体对上述推荐的重视程度；利多大？；弊多大？；估计值的精确性；信心有多大？；风险多大？；可能规避风险么？

JUDGEMENTS	RESEARCH EVIDENCE	ADDITIONAL CONSIDERATIONS
□ Favours the comparison<br>□ Probably favours the comparison<br>□ Does not favour either the intervention or the comparison<br>□ Probably favours the intervention<br>☑ **Favours the intervention**<br>□ Varies<br>□ Uncertain	/	Vote Results : 4/14 chose " Probably favours the intervention " , **10/14 chose "Favours the intervention" .**

7. Resources required: How large are the resource requirements (costs)?

Detailed judgments: How large is the cost of the difference in resource use between the intervention and comparision?

FRNS/SDNS+患儿利妥昔单抗后再予利妥昔巩固治疗可能从无复发率中获益成本支出有多大？干预组和对照组的支出成本差异有多大？

JUDGEMENTS	RESEARCH EVIDENCE	ADDITIONAL CONSIDERATIONS
□ Large costs<br>□ Moderate costs<br>☑ **Negligible costs or savings**<br>□ Moderate savings<br>□ Large savings<br>□ Varies<br>□ Uncertain	Refer to recommendation 18	Vote Results : **8/14 chose "Negligible costs or savings"**, 1/14 chose "Moderate savings"。1/14 chose "Large savings", 4/14 chose "Don' t know" .

8. Certainty of evidence of required resources: What is the certainty of the evidence of resource requirements (costs)?

Detailed judgments: How certain is the evidence of a difference for each type of resource use (eg. drugs, hospitalizations) and the cost of resources?

成本支出的证据质量如何？对 FRNS/SDNS+患儿利妥昔单抗后再予利妥昔巩固治疗可能从无复发率中获益成本支出（包括药物、住院等费用）相关证据的确定性。

JUDGEMENTS	RESEARCH EVIDENCE	ADDITIONAL CONSIDERATIONS
☑ **Very low**<br>□ Low<br>□ Moderate<br>□ High<br>□ No included studies	Refer to recommendation 18	Vote Results: **6/14 chose "Very low"**, 3/14 chose "Low", 1/14 chose "Moderate", 4/14 chose "No included studies" .

9. Cost-effectiveness: Does the cost-effectiveness of the intervention favour the intervention or the comparison?

Detailed judgments: Is the intervention cost-effective, taking into account uncertainty about or variability in the costs, uncertainty about or variability in the net benefit, sensitivity analyses, and the reliability and applicability of the economic evaluation?

对 FRNS/SDNS+患儿利妥昔单抗后再予利妥昔巩固治疗可能从无复发率中获益干预的成本效益分析更倾向于干预组还是对照组？从以下方面分析干预的成本效益：对支出成本的不确定性或变化性；对净利润的不确定性或变化性；敏感性分析；经济评估的可靠性和适用性。

JUDGEMENTS	RESEARCH EVIDENCE	ADDITIONAL CONSIDERATIONS

□ Favours the comparison<br>□ Probably favours the comparison<br>□ Does not favour either the intervention or the comparison<br>□ Probably favours the intervention<br>☑ **Favours the intervention**<br>□ Varies<br>□ No included studies	Refer to recommendation 18	Vote Results : 2/14 chose "Probably favours the intervention", **11/14 chose "Favours the intervention"**, 1/14 chose "No included studies".

10. EQUITY: What would be the impact on health equity?

Detailed judgments: Are there plausible reasons for anticipating differences in the relative effectiveness of the intervention for disadvantaged subgroups or different baseline conditions across disadvantaged subgroups that affect the absolute effectiveness of the intervention or the importance of the problem?

对卫生公平性的影响？FRNS/SDNS+患儿利妥昔单抗后再予利妥昔巩固治疗可能从无复发率中获益的相对有效性在弱势人群中是否有所降低，对此是否有合理的解释？弱势人群的不同基线水平会影响干预的绝对有效性或研究问题的重要性，对此是否有合理的解释？

JUDGEMENTS	RESEARCH EVIDENCE	ADDITIONAL CONSIDERATIONS
□ Reduced<br>☑ **Probably Reduced**<br>□ Probably no impact<br>□ Probably increased<br>□ Increased<br>□ Varies<br>□ Uncertain	/	Vote Results : **10/14 chose "Probably Reduced"**, 1/14 chose "Probably no impact", 1/14 chose "Probably increased", 2/14 chose "Don't know".<br>Probably 会有部分人因各种原因无法使用上激素。

11. ACCEPTABILITY: Is the intervention acceptable to key stakeholders?

Detailed judgments: Are key stakeholders likely not to accept the distribution of the benefits, harms and costs; or the costs or undesirable effects in the short term for desirable effects (benefits) in the future? Are they likely to disagree with the values attached to the desirable or undesirable effects, or not to accept the diagnostic intervention because of ethical concerns?

患儿及其家长是否接受 FRNS/SDNS+患儿利妥昔单抗后再予利妥昔巩固治疗可能从无复发率中获益患儿及其家长者是否会接受干预带来的获益、伤害及支出，或远期获益带来的短期内的不良反应；是否会对利弊判断背后的价值观念持反对意见；是否会出于伦理考虑拒绝诊断性治疗。

JUDGEMENTS	RESEARCH EVIDENCE	ADDITIONAL CONSIDERATIONS
□ No<br>□ Probably no<br>□ Probably yes	/	Vote Results : 5/14 chose "Probably Yes", **9/14 chose "Yes"**.

☑ **Yes**<br>☐ Varies<br>☐ Uncertain		

12. FEASABILITY: Is the option feasible to implement?<br>Detailed judgments: Is it feasible to sustain use of the intervention and to address potential barriers to using it?		
**JUDGEMENTS**	**RESEARCH EVIDENCE**	**ADDITIONAL CONSIDERATIONS**
☐ No<br>☐ Probably no<br>☐ Probably yes<br>☑ **Yes**<br>☐ Varies<br>☐ Uncertain		Vote Results : 2/14 chose "Probably Yes", **12/14 chose "Yes".**

SUMMARY OF JUDGEMENTS

CRITERIA	DECISION							
**1. PROBLEM**	No		Probably no	Probably Yes		**Yes**	Varies	Don’ t know
**2. BENEFITS**	Trivial		Small	Moderate		**Large**	Varies	Don’ t know
**3. HARMS**	Large		Moderate	Small		**Trivial**	Varies	Don’ t know
**4. QUALITY OF EVIDENCE**	**Very low**		Low	Moderate		High	No included studies	
**5. VALUES**	Important uncertainty or variability		Probably Important uncertainty or variability	Probably no Important uncertainty or variability		**No Important uncertainty or variability**	Varies	
**6. BALANCE OF EFFECTS**	Favours the comparison	Probably favours the comparison	Does not favour either the intervention or the comparison		Probably favours the intervention	**Favours the intervention**	Varies	Don’ t know
**7. RESOURCES REQUIRED**	Large costs	Moderate costs	**Negligible costs or savings**		Large savings	Moderate savings	Varies	Don’ t know
**8. CERTAINTY OF EVIDENCE OF REQUIRED RESOURCES**	**Very low**	Low	Moderate		High		No included studies	
**9. COST-EFFECTIVENESS**	Favours the comparison	Probably favours the comparison	Does not favour either the intervention or the comparison		Probably favours the intervention	**Favours the intervention**	Varies	Don’ t know
**10. EQUITY**	Reduced	**Probably Reduced**	Probably no impact		Probably Increased	Increased	Varies	Don’ t know
**11. ACCEPTABILITY**	No	Probably no	Probably Yes		**Yes**		Varies	Don’ t know
**12. FEASIBILITY**	No	Probably no	Probably Yes		**Yes**		Varies	Don’ t know

TYPE OF RECOMMENDATION

Strong recommendation against the intervention	Conditional recommendation against the intervention	Conditional recommendation for either the intervention or the comparison	Conditional recommendation for the intervention	Strong recommendation for the intervention
○	○	○	○	○

CONCLUSIONS

Reason for recommendation

Consolidation therapy is an important factor to consider.

Recommendation(text)

Re-exposure to RTX could decrease the relapse rate in children with FRNS/SDNS after RTX treatment. (1D)

Implementation considerations

1. Included references were few and sample size was small;
2. RTX re-exposure after 6 months and recombination with other preparations do reduce the relapse rate;
3. There may be cases where clinical data is not published.

Research priorities

1. The perspective of study design could be better, considering the end point;
2. Clinical data could be collected from the view of publication.

Recommendation 9 (merged into recommendation 7): RTX + MMF reduces steroid dose by about 0.18 mg•kg-1•d-1 in children with FRNS/SDNS.

Does consolidation therapy improve clinical outcomes in children aged 1-18 years with SSNS after RTX treatment?	
**STUDY TYPE**	Two-arm study
**POPULATION**	FRNS/SDNS+
**INTERVENTION**	RTX+MMF
**COMPARISON**	RTX, RTX+placebo；RTX+CsA
**MAIN OUTCOMES**	Steroid dose reduction
**PERSPECTIVE**	Clinicians, social workers and parents of children with FRNS/SDNS
**SETTING**	Hospital
**CONFLICT OF INTERESTS**	No

ASSESSMENT

1. PROBLEM: Is the problem a priority?

Detailed judgments: The more serious or urgent a problem is, the more likely it is that an option that addresses the problem will be a priority.
FRNS/SDNS 患儿利妥昔单抗治疗基础上加 MMF 维持可使激素用量减少问题具有优先性么？根据问题的严重性和紧急性程度判断其是否具有优先性。严重性和紧急性程度越高，优先性越大

JUDGEMENTS	RESEARCH EVIDENCE	ADDITIONAL CONSIDERATIONS
□ No<br>□ Probably no<br>□ Probably yes<br>□ Yes<br>□ Varies<br>□ Uncertain	/	

2. Desirable effects: How substantial are the desirable anticipated effects?

Detailed judgments: How large are the desirable effects of the intervention taking into account the importance of the outcomes (how much they are valued), and the size of the effect (the

likelihood of experiencing a benefit or how much of an improvement individuals would be likely to experience)?<br>FRNS/SDNS 患儿利妥昔单抗治疗基础上加 MMF 维持可使激素用量减少约 0.18mg/kg/d 的有益影响有多大？根据结局重要性（受重视的程度）和效应值大小（获益的可能性和个体情况改善的程度）对干预措施的有益影响进行判断		
**JUDGEMENTS**	**RESEARCH EVIDENCE**	**ADDITIONAL CONSIDERATIONS**
□ Trivial<br>□ Small<br>□ Moderate<br>□ Large<br>□ Varies<br>□ Uncertain	**Refer to Meta Figure 20**	

3. Undesirable effects: How substantial are the undesirable anticipated effects?

Detailed judgments: How large are the desirable effects of the intervention taking into account the importance of the outcomes (how much they are valued), and the size of the effect (the likelihood of experiencing a benefit or how much of an improvement individuals would be likely to experience)?

FRNS/SDNS 患儿利妥昔单抗治疗基础上加 MMF 维持可使激素用量减少约 0.18mg/kg/d 的不良影响有多大？根据结局重要性（受重视的程度）和效应值大小（获益的可能性和个体情况改善的程度）对干预措施的不良影响进行判断

JUDGEMENTS	RESEARCH EVIDENCE	ADDITIONAL CONSIDERATIONS
□ Large<br>□ Moderate<br>□ Small<br>□ Trivial<br>□ Varies<br>□ Uncertain	/	

4. Certainty of the evidence: What is the overall certainty of the evidence of effects?

Detailed judgments: How good an indication does the research provide of the likely effects across all of the critical outcomes; i.e. the likelihood that the effects will be different enough from what the research found that it might affect a decision about the intervention?

FRNS/SDNS 患儿利妥昔单抗治疗基础上加 MMF 维持可使激素用量减少约 0.18mg/kg/d 有益影响和不良影响相关证据的总体质量？基于 FRNS/SDNS 患儿利妥昔单抗治疗基础上加 MMF 维持可使激素用量减少约 0.18mg/kg/d 的可能影响，判断干预效果是否会对干预决策产生影响

JUDGEMENTS	RESEARCH EVIDENCE	ADDITIONAL CONSIDERATIONS
□ Very low<br>□ Low<br>□ Moderate<br>□ High	/	

□ No included studies		

5. Values: Is there important uncertainty about or variability in how much people value the main outcomes?

Detailed judgments: How much do individuals value each of the main outcomes? Is uncertainty about how much they value each of the outcomes or variability in how much different individual value the outcomes large enough that it could lead to different decisions?
对 FRNS/SDNS 患儿利妥昔单抗治疗基础上加 MMF 维持可使激素用量减少约 0.18mg/kg/d 的重视程度，是否因个体不同而存在不确定性和变化性？个体对 FRNS/SDNS 患儿利妥昔单抗治疗基础上加 MMF 维持可使激素用量减少约 0.18mg/kg/d 的重视程度有多大？不确定性和变化性是否会导致不同的决策？不确定性体现在对上述推荐的理解程度；变化性体现在对上述推荐重视程度的差异。

JUDGEMENTS	RESEARCH EVIDENCE	ADDITIONAL CONSIDERATIONS
□ Important uncertainty or variability<br>□ Possibly important uncertainty or variability<br>□ Probably no important uncertainty or variability<br>□ No important uncertainty or variability	/	

6. Balance of effects: Does the balance between desirable and undesirable effects favour the intervention or the comparison?

Detailed judgments: What is the balance between the desirable and undesirable effects, taking into account how much individuals value the main outcome, how substantial the desirable and undesirable effects are, the certainty of those estimates, discount rates, risk aversion and risk seeking?
权衡 FRNS/SDNS 患儿利妥昔单抗治疗基础上加 MMF 维持可使激素用量减少约 0.18mg/kg/d 利弊后更倾向于干预组还是对照组？从以下方面权衡利弊：个体对上述推荐的重视程度；利多大？弊多大？估计值的精确性；信心有多大？风险多大？可能规避风险么？

JUDGEMENTS	RESEARCH EVIDENCE	ADDITIONAL CONSIDERATIONS
□ Favours the comparison<br>□ Probably favours the comparison<br>□ Does not favour either the intervention or the comparison<br>□ Probably favours the intervention<br>□ Favours the intervention<br>□ Varies<br>□ Uncertain	/	

7. Resources required: How large are the resource requirements (costs)?

Detailed judgments: How large is the cost of the difference in resource use between the intervention and comparision?

FRNS/SDNS 患儿利妥昔单抗治疗基础上加 MMF 维持可使激素用量减少约 0.18mg/kg/d 成本支出有多大？干预组和对照组的支出成本差异有多大？		
**JUDGEMENTS**	**RESEARCH EVIDENCE**	**ADDITIONAL CONSIDERATIONS**
□ Large costs<br>□ Moderate costs<br>□ Negligible costs or savings<br>□ Moderate savings<br>□ Large savings<br>□ Varies<br>□ Uncertain	/	

8. Certainty of evidence of required resources: What is the certainty of the evidence of resource requirements (costs)?

Detailed judgments: How certain is the evidence of a difference for each type of resource use (eg. drugs, hospitalizations) and the cost of resources?

成本支出的证据质量如何？对 FRNS/SDNS 患儿利妥昔单抗治疗基础上加 MMF 维持可使激素用量减少约 0.18mg/kg/d 成本支出（包括药物、住院等费用）相关证据的确定性。

JUDGEMENTS	RESEARCH EVIDENCE	ADDITIONAL CONSIDERATIONS
□ Very low<br>□ Low<br>□ Moderate<br>□ High<br>□ No included studies	/	

9. Cost-effectiveness: Does the cost-effectiveness of the intervention favour the intervention or the comparison?

Detailed judgments: Is the intervention cost-effective, taking into account uncertainty about or variability in the costs, uncertainty about or variability in the net benefit, sensitivity analyses, and the reliability and applicability of the economic evaluation?

对 FRNS/SDNS 患儿利妥昔单抗治疗基础上加 MMF 维持可使激素用量减少约 0.18mg/kg/d 干预的成本效益分析更倾向于干预组还是对照组？从以下方面分析干预的成本效益：对支出成本的不确定性或变化性；对净利润的不确定性或变化性；敏感性分析；经济评估的可靠性和适用性。

JUDGEMENTS	RESEARCH EVIDENCE	ADDITIONAL CONSIDERATIONS

□ Favours the comparison<br>□ Probably favours the comparison<br>□ Does not favour either the intervention or the comparison<br>□ Probably favours the intervention<br>□ Favours the intervention<br>□ Varies<br>□ No included studies	/	

10. EQUITY: What would be the impact on health equity?

Detailed judgments: Are there plausible reasons for anticipating differences in the relative effectiveness of the intervention for disadvantaged subgroups or different baseline conditions across disadvantaged subgroups that affect the absolute effectiveness of the intervention or the importance of the problem?

对卫生公平性的影响？FRNS/SDNS 患儿利妥昔单抗治疗基础上加 MMF 维持可使激素用量减少约 0.18mg/kg/d 的相对有效性是否在弱势人群中有所减少得更少，对此是否有合理的解释？弱势人群的不同基线水平会影响干预的绝对有效性或研究问题的重要性，对此是否有合理的解释？

JUDGEMENTS	RESEARCH EVIDENCE	ADDITIONAL CONSIDERATIONS
□ Reduced<br>□ Probably reduced<br>□ Probably no impact<br>□ Probably increased<br>□ Increased<br>□ Varies<br>□ Uncertain	/	

11. ACCEPTABILITY: Is the intervention acceptable to key stakeholders?

Detailed judgments: Are key stakeholders likely not to accept the distribution of the benefits, harms and costs; or the costs or undesirable effects in the short term for desirable effects (benefits) in the future? Are they likely to disagree with the values attached to the desirable or undesirable effects, or not to accept the diagnostic intervention because of ethical concerns?

患儿及其家长是否接受 FRNS/SDNS 患儿利妥昔单抗治疗基础上加 MMF 维持可使激素用量减少约 0.18mg/kg/d 患儿及其家长是否会接受干预带来的获益、伤害及支出，或远期获益带来的短期内的不良反应；是否会对利弊判断背后的价值观念持反对意见；是否会出于伦理考虑拒绝诊断性治疗。

JUDGEMENTS	RESEARCH EVIDENCE	ADDITIONAL CONSIDERATIONS
□ No<br>□ Probably no<br>□ Probably yes<br>□ Yes<br>□ Varies<br>□ Uncertain	/	

12. FEASABILITY: Is the option feasible to implement?

Detailed judgments: Is it feasible to sustain use of the intervention and to address potential barriers to using it?

JUDGEMENTS	RESEARCH EVIDENCE	ADDITIONAL CONSIDERATIONS
◻ No<br>◻ Probably no<br>◻ Probably yes<br>◻ Yes<br>◻ Varies<br>◻ Uncertain	/	

SUMMARY OF JUDGEMENTS

<table>
<tr><th>CRITERIA</th><th colspan="8">DECISION</th></tr>
<tr><td>1. PROBLEM</td><td colspan="2">No</td><td>Probably no</td><td colspan="2">Probably Yes</td><td>Yes</td><td>Varies</td><td>Don’ t know</td></tr>
<tr><td>2. BENEFITS</td><td colspan="2">Trivial</td><td>Small</td><td colspan="2">Moderate</td><td>Large</td><td>Varies</td><td>Don’ t know</td></tr>
<tr><td>3. HARMS</td><td colspan="2">Large</td><td>Moderate</td><td colspan="2">Small</td><td>Trivial</td><td>Varies</td><td>Don’ t know</td></tr>
<tr><td>4. QUALITY OF EVIDENCE</td><td colspan="2">Very low</td><td>Low</td><td colspan="2">Moderate</td><td>High</td><td colspan="2">No included studies</td></tr>
<tr><td>5. VALUES</td><td colspan="2">Important uncertainty or variability</td><td>Probably Important uncertainty or variability</td><td colspan="2">Probably no Important uncertainty or variability</td><td>No Important uncertainty or variability</td><td colspan="2">Varies</td></tr>
<tr><td>6. BALANCE OF EFFECTS</td><td>Favours the comparison</td><td>Probably favours the comparison</td><td colspan="2">Does not favour either the intervention or the comparison</td><td>Probably favours the intervention</td><td>Favours the intervention</td><td>Varies</td><td>Don’ t know</td></tr>
<tr><td>7. RESOURCES REQUIRED</td><td>Large costs</td><td>Moderate costs</td><td colspan="2">Negligible costs or savings</td><td>Large savings</td><td>Moderate savings</td><td>Varies</td><td>Don’ t know</td></tr>
<tr><td>8. CERTAINTY OF EVIDENCE OF REQUIRED RESOURCES</td><td>Very low</td><td>Low</td><td colspan="2">Moderate</td><td colspan="2">High</td><td colspan="2">No included studies</td></tr>
<tr><td>9. COST-EFFECTIVENESS</td><td>Favours the comparison</td><td>Probably favours the comparison</td><td colspan="2">Does not favour either the intervention or the comparison</td><td>Probably favours the intervention</td><td>Favours the intervention</td><td>Varies</td><td>Don’ t know</td></tr>
<tr><td>10. EQUITY</td><td>Reduced</td><td>Probably Reduced</td><td colspan="2">Probably no impact</td><td>Probably Increased</td><td>Increased</td><td>Varies</td><td>Don’ t know</td></tr>
<tr><td>11. ACCEPTABILITY</td><td>No</td><td>Probably no</td><td colspan="2">Probably Yes</td><td colspan="2">Yes</td><td>Varies</td><td>Don’ t know</td></tr>
<tr><td>12. FEASIBILITY</td><td>No</td><td>Probably no</td><td colspan="2">Probably Yes</td><td colspan="2">Yes</td><td>Varies</td><td>Don’ t know</td></tr>
</table>

TYPE OF RECOMMENDATION

Strong recommendation against the intervention	Conditional recommendation against the intervention	Conditional recommendation for either the intervention or the comparison	Conditional recommendation for the intervention	Strong recommendation for the intervention
○	○	○	○	○

CONCLUSIONS

Reason for recommendation

This recommendation is related to steroid dose reduction, and after expert discussion it is merged with recommendation 7.

Recommendation 10: RTX treatment improves the quality of life in children with FRNS/SDNS. (1D)

Does consolidation therapy improve clinical outcomes in children aged 1-18 years with SSNS after RTX treatment?	
STUDY TYPE	Self-control study
POPULATION	FRNS/SDNS+
INTERVENTION	RTX (375mg/m2, four times semiannually) followed by mizoribine (twice a week at a dose of 500 mg on the first day and 550 mg on the second day)
COMPARISON	No
MAIN OUTCOMES	Quality of life (with a follow-up of ≥ 12 months)
PERSPECTIVE	Clinicians, social workers and parents of children with FRNS/SDNS
SETTING	Hospital
CONFLICT OF INTERETS	No

ASSESSMENT

1. PROBLEM: Is the problem a priority?

Detailed judgments: The more serious or urgent a problem is, the more likely it is that an option that addresses the problem will be a priority.
FRNS/SDNS 患儿应用利妥昔单抗后生存质量改善问题具有优先性么？根据问题的严重性和紧急性程度判断其是否具有优先性。严重性和紧急性程度越高，优先性越大

JUDGEMENTS	RESEARCH EVIDENCE	ADDITIONAL CONSIDERATIONS
□ No<br>□ Probably no<br>□ Probably yes<br>☑ **Yes**<br>□ Varies<br>□ Uncertain	/	Vote Results : 5/14 chose " Probably Yes " , **9/14 chose "Yes" .**

2. Desirable effects: How substantial are the desirable anticipated effects?

Detailed judgments: How large are the desirable effects of the intervention taking into account the importance of the outcomes (how much they are valued), and the size of the effect (the likelihood of experiencing a benefit or how much of an improvement individuals would be likely to experience)?
FRNS/SDNS 患儿应用利妥昔单抗后生存质量有所改善的有益影响有多大？根据结局重要性（受重视的程度）和效应值大小（获益的可能性和个体情况改善的程度）对干预措施的有益影响进行判断

<table>
<tr><th>JUDGEMENTS</th><th>RESEARCH EVIDENCE</th><th>ADDITIONAL CONSIDERATIONS</th></tr>
<tr>
<td>□ Trivial
□ Small
□ Moderate
☑ **Large**
□ Varies
□ Uncertain</td>
<td>
<table>
<tr><th rowspan="2">结局；患儿数量
研究类型和数量</th><th rowspan="2">相对效应（95%CI）</th><th colspan="3">预期绝对效应值（事件数/例数）</th><th rowspan="2">证据质量</th><th rowspan="2">升降级说明</th></tr>
<tr><th>对照组</th><th>干预组</th><th>风险差</th></tr>
<tr><td colspan="7">生活质量</td></tr>
<tr><td colspan="7">人群：FRNS/SDNS+；干预组: RTX（375mg/m2，半年一次×4 次）+咪唑立宾（每周 2 次，第 1 天 500 mg，第 2 天 550mg），1 项病例报道[400]，22 例</td></tr>
<tr><td>基于 PedsQL4.0 量表随访 24 个月生活质量评分</td><td>/</td><td colspan="2">患者基线：81.1(95%CI:74.6~87.5)
24 个月：91.5(95%CI:85.1~97.9)，$P<0.001$
父母基线：74.9（95%CI:68.5~81.3）,24 个月：85.2（95%CI:78.8~91.7），$P<0.001$</td><td>/</td><td>极低</td><td>/</td></tr>
</table>
</td>
<td>Vote Results：3/14 chose “Moderate”，**11/14 chose “Large”**.</td>
</tr>
</table>

3. Undesirable effects: How substantial are the undesirable anticipated effects?

Detailed judgments: How large are the desirable effects of the intervention taking into account the importance of the outcomes (how much they are valued), and the size of the effect (the likelihood of experiencing a benefit or how much of an improvement individuals would be likely to experience)?

FRNS/SDNS 患儿应用利妥昔单抗后生存质量有所改善的不良影响有多大？根据结局重要性（受重视的程度）和效应值大小（获益的可能性和个体情况改善的程度）对干预措施的不良影响进行判断

JUDGEMENTS	RESEARCH EVIDENCE	ADDITIONAL CONSIDERATIONS
□ Large<br>□ Moderate<br>□ small<br>☑ **Tivial**<br>□ Varies<br>□ Uncertain	/	Vote Results：6/14 chose “Small”，**8/14 chose “Trivial”**.

4. Certainty of the evidence: What is the overall certainty of the evidence of effects?

Detailed judgments: How good an indication does the research provide of the likely effects across all of the critical outcomes; i.e. the likelihood that the effects will be different enough from what the research found that it might affect a decision about the intervention?

FRNS/SDNS 患儿应用利妥昔单抗后生存质量有所改善有益影响和不良影响相关证据的总体质量？基于干预对所有关键结局的可能影响，判断干预效果是否会对干预决策产生影响

JUDGEMENTS	RESEARCH EVIDENCE	ADDITIONAL CONSIDERATIONS
☑ **Very low**<br>□ Low<br>□ Moderate<br>□ High<br>□ No included studies	/	Vote Results：**12/14 chose “Very low”**，2/14 chose “Low”.

5. Values: Is there important uncertainty about or variability in how much people value the main outcomes?

Detailed judgments: How much do individuals value each of the main outcomes? Is uncertainty about how much they value each of the outcomes or variability in how much different individual value the outcomes large enough that it could lead to different decisions?

5. 价值观念：对 FRNS/SDNS 患儿应用利妥昔单抗后生存质量有所改善的重视程度，是否因个体不同而存在不确定性和变化性?

Detailed judgments: 个体对 FRNS/SDNS 患儿应用利妥昔单抗后生存质量有所改善的重视程度有多大？不确定性和变化性是否会导致不同的决策？不确定性体现在对上述推荐的理解程度；变化性体现在对上述推荐重视程度的差异。

JUDGEMENTS	RESEARCH EVIDENCE	ADDITIONAL CONSIDERATIONS
□ Important uncertainty or variability<br>□ Possibly important uncertainty or variability<br>□ Probably no important uncertainty or variability<br>☑ **No Important uncertainty or variability**	/	Vote Results : 2/14 chose “ Probably no Important uncertainty or variability”, **12/14 chose “No Important uncertainty or variability”.**

6. Balance of effects: Does the balance between desirable and undesirable effects favour the intervention or the comparison?

Detailed judgments: What is the balance between the desirable and undesirable effects, taking into account how much individuals value the main outcome, how substantial the desirable and undesirable effects are, the certainty of those estimates, discount rates, risk aversion and risk seeking?

6. 利弊权衡：权衡 FRNS/SDNS 患儿应用利妥昔单抗后生存质量有所改善利弊

Detailed judgments: 从以下方面权衡利弊：个体对上述推荐的重视程度；利多大？；弊多大？；估计值的精确性；信心有多大？；风险多大？；可能规避风险么？

JUDGEMENTS	RESEARCH EVIDENCE	ADDITIONAL CONSIDERATIONS
□ Favours the comparison<br>□ Probably favours the comparison **(弊 Large 于利)**<br>□ Does not favour either the intervention or the comparison **(弊利相当)**<br>□ Probably favours the intervention **(利 Large 于弊)**<br>☑ **Favours the intervention**<br>□ Varies<br>□ Uncertain	/	Vote Results : 6/14 chose “ Probably favours the intervention ” , **8/14 chose “Favours the intervention”.**

7. Resources required: How large are the resource requirements (costs)?

Detailed judgments: How large is the cost of the difference in resource use between the intervention and comparision?

7. 成本支出：FRNS/SDNS 患儿应用利妥昔单抗后生存质量有所改善成本支出有多大?

Detailed judgments: 支出成本有多大?

JUDGEMENTS	RESEARCH EVIDENCE	ADDITIONAL CONSIDERATIONS
☐ Large costs<br>☐ Moderate costs<br>☑ **Negligible costs or savings**<br>☐ Moderate savings<br>☐ Large savings<br>☐ Varies<br>☐ Uncertain	Refer to recommendation 18	Vote Results : 1/14 chose "Moderate costs", **7/14 chose "Negligible costs or savings",** 1/14 chose "Moderate savings", 5/14 chose "Don't know".

8. Certainty of evidence of required resources: What is the certainty of the evidence of resource requirements (costs)?

Detailed judgments: How certain is the evidence of a difference for each type of resource use (eg. drugs, hospitalizations) and the cost of resources?

8. 成本支出的证据质量：FRNS/SDNS 患儿应用利妥昔单抗后生存质量有所改善成本支出的证据质量如何?

Detailed judgments: 对 FRNS/SDNS 患儿应用利妥昔单抗后生存质量有所改善成本支出（包括药物、住院等费用）相关证据的确定性。

How certain is the evidence of a difference for each type of resource use (eg. drugs, hospitalizations) and the cost of resources?

JUDGEMENTS	RESEARCH EVIDENCE	ADDITIONAL CONSIDERATIONS
☑ **Very low**<br>☐ Low<br>☐ Moderate<br>☐ High<br>☐ No included studies	Refer to recommendation 18	Vote Results: **6/14 chose "Very low"**, 4/14 chose "Low", 1/14 chose "Moderate", 3/14 chose "No included studies".

9. Cost-effectiveness: Does the cost-effectiveness of the intervention favour the intervention or the comparison?

Detailed judgments: Is the intervention cost-effective, taking into account uncertainty about or variability in the costs, uncertainty about or variability in the net benefit, sensitivity analyses, and the reliability and applicability of the economic evaluation?

9. 成本效益：对 FRNS/SDNS 患儿应用利妥昔单抗后生存质量有所改善干预的成本效益分析

Detailed judgments: 从以下方面分析干预的成本效益：对支出成本的不确定性或变化性；对净利润的不确定性或变化性；敏感性分析；经济评估的可靠性和适用性。		
**JUDGEMENTS**	**RESEARCH EVIDENCE**	**ADDITIONAL CONSIDERATIONS**
□ Favours the comparison<br>□ Probably favours the comparison<br>□ Does not favour either the intervention or the comparison<br>□ Probably favours the intervention<br>☑ **Favours the intervention**<br>□ Varies<br>□ No included studies	Refer to recommendation 18	Vote Results : 4/14 chose "Probably favours the intervention", **9/14 chose "Favours the intervention"**, 1/14 chose "No included studies".

10. EQUITY: What would be the impact on health equity?

Detailed judgments: Are there plausible reasons for anticipating differences in the relative effectiveness of the intervention for disadvantaged subgroups or different baseline conditions across disadvantaged subgroups that affect the absolute effectiveness of the intervention or the importance of the problem?

10. EQUITY: 对卫生公平性的影响?

Detailed judgments: FRNS/SDNS 患儿应用利妥昔单抗后生存质量有所改善的相对有效性在弱势人群中是否有所降低，对此是否有合理的解释？弱势人群的不同基线水平会影响干预的绝对有效性或研究问题的重要性，对此是否有合理的解释?

**JUDGEMENTS**	**RESEARCH EVIDENCE**	**ADDITIONAL CONSIDERATIONS**
□ Reduced<br>☑ **Probably Reduced**<br>□ Probably no impact<br>□ Probably increased<br>□ Increased<br>□ Varies<br>□ Uncertain	/	Vote Results : **10/14 chose "Probably Reduced"**, 2/14 chose "Increased", 2/14 chose "Don't know".

11. ACCEPTABILITY: Is the intervention acceptable to key stakeholders?

Detailed judgments: Are key stakeholders likely not to accept the distribution of the benefits, harms and costs; or the costs or undesirable effects in the short term for desirable effects (benefits) in the future? Are they likely to disagree with the values attached to the desirable or undesirable effects, or not to accept the diagnostic intervention because of ethical concerns?

11. ACCEPTABILITY: 患儿及其家长是否接受 FRNS/SDNS 患儿应用利妥昔单抗后生存质量有所改善

Detailed judgments: 患儿及其家长是否会接受干预带来的获益、伤害及支出，或远期获益带来的短期内的不良反应；是否会对利弊判断背后的价值观念持反对意见；是否会出于伦理考虑拒绝诊断性治疗。

**JUDGEMENTS**	**RESEARCH EVIDENCE**	**ADDITIONAL CONSIDERATIONS**

☐ No<br>☐ Probably no<br>☐ Probably yes<br>☑ **Yes**<br>☐ Varies<br>☐ Uncertain	/	Vote Results : 4/14 chose " Probably Yes " , **10/14 chose "Yes" .**

12. FEASABILITY: Is the option feasible to implement?

Detailed judgments: Is it feasible to sustain use of the intervention and to address potential barriers to using it?

JUDGEMENTS	RESEARCH EVIDENCE	ADDITIONAL CONSIDERATIONS
☐ No<br>☐ Probably no<br>☐ Probably yes<br>☑ **Yes**<br>☐ Varies<br>☐ Uncertain	/	Vote Results : 2/14 chose " Probably Yes " , **12/14 chose "Yes" .**

SUMMARY OF JUDGEMENTS

<table>
<tr><th>CRITERIA</th><th colspan="8">DECISION</th></tr>
<tr><td>1. PROBLEM</td><td colspan="2">No</td><td>Probably no</td><td colspan="2">Probably Yes</td><td>Yes</td><td>Varies</td><td>Don’ t know</td></tr>
<tr><td>2. BENEFITS</td><td colspan="2">Trivial</td><td>Small</td><td colspan="2">Moderate</td><td>Large</td><td>Varies</td><td>Don’ t know</td></tr>
<tr><td>3. HARMS</td><td colspan="2">Large</td><td>Moderate</td><td colspan="2">Small</td><td>Trivial</td><td>Varies</td><td>Don’ t know</td></tr>
<tr><td>4. QUALITY OF EVIDENCE</td><td colspan="2">Very low</td><td>Low</td><td colspan="2">Moderate</td><td>High</td><td colspan="2">No included studies</td></tr>
<tr><td>5. VALUES</td><td colspan="2">Important uncertainty or variability</td><td>Probably Important uncertainty or variability</td><td colspan="2">Probably no Important uncertainty or variability</td><td>No Important uncertainty or variability</td><td colspan="2">Varies</td></tr>
<tr><td>6. BALANCE OF EFFECTS</td><td>Favours the comparison</td><td>Probably favours the comparison</td><td colspan="2">Does not favour either the intervention or the comparison</td><td>Probably favours the intervention</td><td>Favours the intervention</td><td>Varies</td><td>Don’ t know</td></tr>
<tr><td>7. RESOURCES REQUIRED</td><td>Large costs</td><td>Moderate costs</td><td colspan="2">Negligible costs or savings</td><td>Large savings</td><td>Moderate savings</td><td>Varies</td><td>Don’ t know</td></tr>
<tr><td>8. CERTAINTY OF EVIDENCE OF REQUIRED RESOURCES</td><td>Very low</td><td>Low</td><td colspan="2">Moderate</td><td colspan="2">High</td><td colspan="2">No included studies</td></tr>
<tr><td>9. COST-EFFECTIVENESS</td><td>Favours the comparison</td><td>Probably favours the comparison</td><td colspan="2">Does not favour either the intervention or the comparison</td><td>Probably favours the intervention</td><td>Favours the intervention</td><td>Varies</td><td>Don’ t know</td></tr>
<tr><td>10. EQUITY</td><td>Reduced</td><td>Probably Reduced</td><td colspan="2">Probably no impact</td><td>Probably Increased</td><td>Increased</td><td>Varies</td><td>Don’ t know</td></tr>
<tr><td>11. ACCEPTABILITY</td><td>No</td><td>Probably no</td><td colspan="2">Probably Yes</td><td colspan="2">Yes</td><td>Varies</td><td>Don’ t</td></tr>
</table>

CRITERIA	DECISION					
						know
12. FEASIBILITY	No	Probably no	Probably Yes	**Yes**	Varies	Don' t know

TYPE OF RECOMMENDATION

Strong recommendation against the intervention	Conditional recommendation against the intervention	Conditional recommendation for either the intervention or the comparison	Conditional recommendation for the intervention	Strong recommendation for the intervention
○	○	○	○	○

CONCLUSIONS

Reason for recommendation

1. Quality of life has reference significance;
2. The outcome indicators were statistically significant.

Recommendation(text)

RTX treatment improves the quality of life in children with FRNS/SDNS. (1D)

Implementation considerations

Few references, small sample size, very low quality of evidence

Recommendation 11: Re-exposure to RTX in relapsed children with FRNS/SDNS after RTX treatment improves the relapse-free survival rate and prolongs the relapse-free survival. (1D)

Does re-exposure to RTX in relapsed children with FRNS/SDNS after RTX treatment improve clinical outcomes in children aged 1-18 years with SSNS after RTX treatment?	
**STUDY TYPE**	Self-control study
**POPULATION**	FRNS/SDNS+
**INTERVENTION**	Before: RTX 375 mg/m2 qw×1-2w
**COMPARISON**	After: RTX 375 mg/m2 qw×1-2w
**MAIN OUTCOMES**	24-month relapse-free survival rate
**PERSPECTIVE**	Clinicians, social workers and parents of children with FRNS/SDNS
**SETTING**	Hospital
**CONFLICT OF INTERESTS**	No

ASSESSMENT

1. PROBLEM: Is the problem a priority?

Detailed judgments: Detailed judgments: The more serious or urgent a problem is, the more likely it is that an option that addresses the problem will be a priority.
FRNS/SDNS 接受 RTX 治疗复发后再 RTX 无复发生存率和无复发生存时间问题具有优先性么？根据问题的严重性和紧急性程度判断其是否具有优先性。严重性和紧急性程度越高，优先性越大

JUDGEMENTS	RESEARCH EVIDENCE	ADDITIONAL CONSIDERATIONS
□ No<br>□ Probably no<br>□ Probably yes<br>☑ **Yes**<br>□ Varies<br>□ Uncertain	/	Vote Results : 2/14 chose " Probably Yes ", **12/14 chose "Yes"** .

2. Desirable effects: How substantial are the desirable anticipated effects?

Detailed judgments: How large are the desirable effects of the intervention taking into account the importance of the outcomes (how much they are valued), and the size of the effect (the likelihood of experiencing a benefit or how much of an improvement individuals would be likely to experience)?
FRNS/SDNS 接受 RTX 治疗复发后再 RTX 无复发生存率和无复发生存时间获益的有益影响有多大？根据结局重要性（受重视的程度）和效应值大小（获益的可能性和个体情况改善的程度）对干预措施的有益影响进行判断

JUDGEMENTS

- ☐ Trivial
- ☐ Small
- ☐ Moderate
- ☑ **Large**
- ☐ Varies
- ☐ Uncertain

RESEARCH EVIDENCE

结局；患儿数量；研究类型和数量	相对效应 (95%CI)	预期绝对效应值（事件数/例数）			证据质量	升降级说明
		对照组	干预组	风险差		
利妥昔单抗后复发加 RTX 随访时间≥12 个月复发或缓解						
**人群：FRNS/SDNS+；干预组：利妥昔单抗（375 mg/m² 2 剂）+RTX（375 mg/m²1~2 剂）；1 篇队列研究[196]，10 例，伴 IS**						
随访≥6 个月	/	/	10/10 缓解	/	极低	**降级因素：**<br>偏倚风险降 2 级<br>不一致性不适用<br>精确性降 1 级<br>发表偏倚不适用<br>间接性不降级<br>**升级因素：无**
**人群：FRNS/SDNS+；干预组：利妥昔单抗（375 mg/m² 1~2 剂）+RTX（1~3 疗程）；1 篇队列研究[418]，5 例，伴 IS**						
随访 24 个月		首疗程中位时间 10 个月	多疗程后 5/5 无复发>24 个月	/	/	
**人群：FRNS/SDNS+；干预组：利妥昔单抗（375mg/m² 1~2 剂）+重复 1~4 疗程 RTX（375mg/m²）（基线及之后每月检测 B 细胞）；1 项队列研究[339]，46 例，IS 减停**						
		停激素+CNIs 后中位无复发时间 5.6（四分位：4.3–8.1）月	停激素+CNIs 后中位无复发时间 8.5（四分位：6.5–11.7）月	/	极低	**降级因素：**<br>偏倚风险降 2 级<br>不一致性不降级<br>精确性降 1 级<br>发表偏倚不适用<br>间接性不降级<br>**升级因素：无**

ADDITIONAL CONSIDERATIONS

Vote Results : **14/14 chose "Large".**

3. Undesirable effects: How substantial are the undesirable anticipated effects?

Detailed judgments: How large are the desirable effects of the intervention taking into account the importance of the outcomes (how much they are valued), and the size of the effect (the likelihood of experiencing a benefit or how much of an improvement individuals would be likely to experience)?
FRNS/SDNS 接受 RTX 治疗复发后再 RTX 无复发生存率和无复发生存时间获益的不良影响有多大？根据结局重要性（受重视的程度）和效应值大小（获益的可能性和个体情况改善的程度）对干预措施的不良影响进行判断

JUDGEMENTS

- ☐ Large
- ☐ Moderate
- ☐ Small
- ☑ **Trivial**
- ☐ Varies
- ☐ Uncertain

RESEARCH EVIDENCE

/

ADDITIONAL CONSIDERATIONS

Vote Results : 1/14 chose " Moderate ", 4/14 chose "Small", **9/14 chose "Trivial".**

**4. Certainty of the evidence: What is the overall certainty of the evidence of effects?**<br>Detailed judgments: How good an indication does the research provide of the likely effects across all of the critical outcomes; i.e. the likelihood that the effects will be different enough from what the research found that it might affect a decision about the intervention?<br>FRNS/SDNS 接受 RTX 治疗复发后再 RTX 无复发生存率和无复发生存时间获益有益影响和不良影响相关证据的总体质量？基于 FRNS/SDNS 接受 RTX 治疗复发后再 RTX 无复发生存率和无复发生存时间获益的可能影响，判断干预效果是否会对干预决策产生影响		
**JUDGEMENTS**	**RESEARCH EVIDENCE**	**ADDITIONAL CONSIDERATIONS**
☑ **Very low**<br>□ Low<br>□ Moderate<br>□ High<br>□ No included studies	/	Vote Results: **11/14 chose "Very low"**, 2/14 chose "Low", 1/14 chose "No included studies".
**5. Values: Is there important uncertainty about or variability in how much people value the main outcomes?**<br>Detailed judgments: How much do individuals value each of the main outcomes? Is uncertainty about how much they value each of the outcomes or variability in how much different individual value the outcomes large enough that it could lead to different decisions?<br>对 FRNS/SDNS 接受 RTX 治疗复发后再 RTX 无复发生存率和无复发生存时间获益的重视程度，是否因个体不同而存在不确定性和变化性？个体对 FRNS/SDNS 接受 RTX 治疗复发后再 RTX 无复发生存率和无复发生存时间获益的重视程度有多大？不确定性和变化性是否会导致不同的决策？不确定性体现在对上述推荐的理解程度；变化性体现在对上述推荐重视程度的差异。		
**JUDGEMENTS**	**RESEARCH EVIDENCE**	**ADDITIONAL CONSIDERATIONS**
□ Important uncertainty or variability<br>□ Possibly important uncertainty or variability<br>□ Probably no important uncertainty or variability<br>☑ **No Important uncertainty or variability**	/	Vote Results: 1/14 chose "Probably no Important uncertainty or variability", **13/14 chose "No Important uncertainty or variability"**.
**6. Balance of effects: Does the balance between desirable and undesirable effects favour the intervention or the comparison?**<br>Detailed judgments: What is the balance between the desirable and undesirable effects, taking into account how much individuals value the main outcome, how substantial the desirable and undesirable effects are, the certainty of those estimates, discount rates, risk aversion and risk seeking?<br>权衡 FRNS/SDNS 接受 RTX 治疗复发后再 RTX 无复发生存率和无复发生存时间获益利弊。从以下方面权衡利弊：个体对上述推荐的重视程度；利多大？；弊多大？；估计值的精确性；信心有多大？；风险多大？；可能规避风险么？		
**JUDGEMENTS**	**RESEARCH EVIDENCE**	**ADDITIONAL CONSIDERATIONS**

□ Favours the comparison<br>□ Probably favours the comparison<br>□ Does not favour either the intervention or the comparison<br>□ Probably favours the intervention<br>☑ **Favours the intervention**<br>□ Varies<br>□ Uncertain	/	Vote Results : 4/14 chose "Probably favours the intervention", **10/14 chose "Favours the intervention"**.

7. Resources required: How large are the resource requirements (costs)?

Detailed judgments: How large is the cost of the difference in resource use between the intervention and comparision?

FRNS/SDNS 接受 RTX 治疗复发后再 RTX 无复发生存率和无复发生存时间获益成本支出有多大？支出成本有多大？

JUDGEMENTS	RESEARCH EVIDENCE	ADDITIONAL CONSIDERATIONS
□ Large costs<br>□ Moderate costs<br>☑ **Negligible costs or savings**<br>□ Moderate savings<br>□ Large savings<br>□ Varies<br>□ Uncertain	/	Vote Results : 1/14 chose "Moderate costs", **7/14 chose "Negligible costs or savings"**, 1/14 chose "Moderate savings", 5/14 chose "Don't know".

8. Certainty of evidence of required resources: What is the certainty of the evidence of resource requirements (costs)?

Detailed judgments: How certain is the evidence of a difference for each type of resource use (eg. drugs, hospitalizations) and the cost of resources?

成本支出的证据质量如何？对 FRNS/SDNS 接受 RTX 治疗复发后再 RTX 无复发生存率和无复发生存时间获益成本支出（包括药物、住院等费用）相关证据的确定性。

JUDGEMENTS	RESEARCH EVIDENCE	ADDITIONAL CONSIDERATIONS
□ Very low<br>☑ **Low**<br>□ Moderate<br>□ High<br>□ No included studies	Refer to recommendation 18	Vote Results : 5/14 chose "Very low", **6/14 chose "Low"**, 3/14 chose "No included studies".

9. Cost-effectiveness: Does the cost-effectiveness of the intervention favour the intervention or the comparison?

Detailed judgments: Is the intervention cost-effective, taking into account uncertainty about or variability in the costs, uncertainty about or variability in the net benefit, sensitivity analyses, and the reliability and applicability of the economic evaluation?

对 FRNS/SDNS 接受 RTX 治疗复发后再 RTX 无复发生存率和无复发生存时间获益干预的成本效益分析更倾向于再干预？从以下方面分析干预的成本效益：对支出成本的不确定性或变化性；对净利润的不确定性或变化性；敏感性分析；经济评估的可靠性和适用性。

JUDGEMENTS	RESEARCH EVIDENCE	ADDITIONAL CONSIDERATIONS
□ Favours the comparison<br>□ Probably favours the comparison<br>□ Does not favour either the intervention or the comparison<br>□ Probably favours the intervention<br>☑ **Favours the intervention**<br>□ Varies<br>□ No included studies	Refer to recommendation 18	Vote Results : 2/14 chose "Probably favours the intervention", **11/14 chose "Favours the intervention"**, 1/14 chose "No included studies".

10. EQUITY: What would be the impact on health equity?

Detailed judgments: Are there plausible reasons for anticipating differences in the relative effectiveness of the intervention for disadvantaged subgroups or different baseline conditions across disadvantaged subgroups that affect the absolute effectiveness of the intervention or the importance of the problem?

对卫生公平性的影响？FRNS/SDNS 接受 RTX 治疗复发后再 RTX 无复发生存率和无复发生存时间获益的相对有效性是否在弱势人群中有所降低，对此是否有合理的解释？弱势人群的不同基线水平会影响干预的绝对有效性或研究问题的重要性，对此是否有合理的解释？

JUDGEMENTS	RESEARCH EVIDENCE	ADDITIONAL CONSIDERATIONS
□ Reduced<br>☑ **Probably Reduced**<br>□ Probably no impact<br>□ Probably increased<br>□ Increased<br>□ Varies<br>□ Uncertain	Refer to recommendation 18	Vote Results : **9/14 chose "Probably Reduced"**, 2/14 chose "Probably no impact", 1/14 chose "Probably increased", 2/14 chose "Don't know".

11. ACCEPTABILITY: Is the intervention acceptable to key stakeholders?

Detailed judgments: Are key stakeholders likely not to accept the distribution of the benefits, harms and costs; or the costs or undesirable effects in the short term for desirable effects (benefits) in the future? Are they likely to disagree with the values attached to the desirable or undesirable effects, or not to accept the diagnostic intervention because of ethical concerns?

患儿及其家长者是否接受 FRNS/SDNS 接受 RTX 治疗复发后再 RTX 无复发生存率和无复发生存时间获益。患儿及其家长是否会接受再干预带来的获益、伤害及支出，或远期获益带来的短期内的不良反应；是否

会对利弊判断背后的价值观念持反对意见；是否会出于伦理考虑拒绝诊断性治疗。		
**JUDGEMENTS**	**RESEARCH EVIDENCE**	**ADDITIONAL CONSIDERATIONS**
☐ No<br>☐ Probably no<br>☐ Probably yes<br>☑ **Yes**<br>☐ Varies<br>☐ Uncertain	/	Vote Results : 2/14 chose " Probably Yes " , **12/14 chose "Yes" .**

12. FEASABILITY: Is the option feasible to implement?

Detailed judgments: Is it feasible to sustain use of the intervention and to address potential barriers to using it?

**JUDGEMENTS**	**RESEARCH EVIDENCE**	**ADDITIONAL CONSIDERATIONS**
☐ No<br>☐ Probably no<br>☐ Probably yes<br>☑ **Yes**<br>☐ Varies<br>☐ Uncertain	/	Vote Results : **14/14 chose "Yes" .**

SUMMARY OF JUDGEMENTS

<table>
<tr><th>CRITERIA</th><th colspan="8">DECISION</th></tr>
<tr><td>1. PROBLEM</td><td colspan="2">No</td><td>Probably no</td><td colspan="2">Probably Yes</td><td>Yes</td><td>Varies</td><td>Don’ t know</td></tr>
<tr><td>2. BENEFITS</td><td colspan="2">Trivial</td><td>Small</td><td colspan="2">Moderate</td><td>Large</td><td>Varies</td><td>Don’ t know</td></tr>
<tr><td>3. HARMS</td><td colspan="2">Large</td><td>Moderate</td><td colspan="2">Small</td><td>Trivial</td><td>Varies</td><td>Don’ t know</td></tr>
<tr><td>4. QUALITY OF EVIDENCE</td><td colspan="2">Very low</td><td>Low</td><td colspan="2">Moderate</td><td>High</td><td colspan="2">No included studies</td></tr>
<tr><td>5. VALUES</td><td colspan="2">Important uncertainty or variability</td><td>Probably Important uncertainty or variability</td><td colspan="2">Probably no Important uncertainty or variability</td><td>No Important uncertainty or variability</td><td colspan="2">Varies</td></tr>
<tr><td>6. BALANCE OF EFFECTS</td><td>Favours the comparison</td><td>Probably favours the comparison</td><td colspan="2">Does not favour either the intervention or the comparison</td><td>Probably favours the intervention</td><td>Favours the intervention</td><td>Varies</td><td>Don’ t know</td></tr>
<tr><td>7. RESOURCES REQUIRED</td><td>Large costs</td><td>Moderate costs</td><td colspan="2">Negligible costs or savings</td><td>Large savings</td><td>Moderate savings</td><td>Varies</td><td>Don’ t know</td></tr>
<tr><td>8. CERTAINTY OF EVIDENCE OF REQUIRED RESOURCES</td><td>Very low</td><td>Low</td><td colspan="2">Moderate</td><td colspan="2">High</td><td colspan="2">No included studies</td></tr>
<tr><td>9. COST-EFFECTIVENESS</td><td>Favours the comparison</td><td>Probably favours the comparison</td><td colspan="2">Does not favour either the intervention or the comparison</td><td>Probably favours the intervention</td><td>Favours the intervention</td><td>Varies</td><td>Don’ t know</td></tr>
<tr><td>10. EQUITY</td><td>Reduced</td><td>Probably Reduced</td><td colspan="2">Probably no impact</td><td>Probably Increased</td><td>Increased</td><td>Varies</td><td>Don’ t know</td></tr>
<tr><td>11. ACCEPTABILITY</td><td>No</td><td>Probably no</td><td colspan="2">Probably Yes</td><td colspan="2">Yes</td><td>Varies</td><td>Don’ t know</td></tr>
<tr><td>12. FEASIBILITY</td><td>No</td><td>Probably no</td><td colspan="2">Probably Yes</td><td colspan="2">Yes</td><td>Varies</td><td>Don’ t know</td></tr>
</table>

TYPE OF RECOMMENDATION

Strong recommendation against the intervention	Conditional recommendation against the intervention	Conditional recommendation for either the intervention or the comparison	Conditional recommendation for the intervention	Strong recommendation for the intervention
○	○	○	○	○

CONCLUSIONS

Recommendation(text)

Re-exposure to RTX in relapsed children with FRNS/SDNS after RTX treatment improves the relapse-free survival rate and prolongs the relapse-free survival. (1D)

Implementation considerations

Included references are all case series reports and the quality of evidence is very low.

Recommendation 12: In children with FRNS/SDNS receiving 1~4 doses of RTX treatment, B-cell depletion is achieved at one month after the first dose, B-cell reconstitution occurs at 5~6 months, and multiple courses of RTX treatment slightly prolongs the duration of B-cell depletion. (1D)

Is it feasible to take peripheral CD19+/CD20+ B cell count as a monitoring indicator in children aged 1-18 years with SSNS after RTX treatment?	
**STUDY TYPE**	Single-arm study
**POPULATION**	FRNS/SDNS+, FRNS/SDNS-
**INTERVENTION**	RTX 1~4 doses
**COMPARISON**	No
**MAIN OUTCOMES**	1-month B cell depletion rate, the time to B cell reconstitution, B cell sustained depletion time after multiple courses of RTX treatment
**PERSPECTIVE**	Clinicians, social workers and parents of children with FRNS/SDNS
**SETTING**	Hospital
**CONFLICT OF INTERESTS**	No

ASSESSMENT

1. PROBLEM: Is the problem a priority?<br>Detailed judgments: The more serious or urgent a problem is, the more likely it is that an option that addresses the problem will be a priority.<br>FRNS/SDNS 予 RTX 1~4 剂治疗首剂输注 1 月后 B 细胞耗竭率、B 细胞重建时间，多疗程后 B 细胞耗竭持续时间问题具有优先性么？根据问题的严重性和紧急性程度判断其是否具有优先性。严重性和紧急性程度越高，优先性越大		
**JUDGEMENTS**	**RESEARCH EVIDENCE**	**ADDITIONAL CONSIDERATIONS**

□ No<br>□ Probably no<br>□ Probably yes<br>☑ **Yes**<br>□ Varies<br>□ Uncertain	/	Vote Results : 2/14 chose "Probably Yes", **12/14 chose "Yes"**.

2. Desirable effects: How substantial are the desirable anticipated effects?

Detailed judgments: How large are the desirable effects of the intervention taking into account the importance of the outcomes (how much they are valued), and the size of the effect (the likelihood of experiencing a benefit or how much of an improvement individuals would be likely to experience)?

FRNS/SDNS 予 RTX 1~4 剂治疗首剂输注 1 月后 B 细胞耗竭达＜1%， 5～6 个月 B 细胞重建，多疗程后 B 细胞耗竭持续时间延长的有益影响有多大？根据结局重要性（受重视的程度）和效应值大小（获益的可能性和个体情况改善的程度）对干预措施的有益影响进行判断

JUDGEMENTS	RESEARCH EVIDENCE	ADDITIONAL CONSIDERATIONS
□ Trivial<br>□ Small<br>□ Moderate<br>☑ **Large**<br>□ Varies<br>□ Uncertain	(see table below)	Vote Results : 3/14 chose " Moderate ", **11/14 chose "Large"**.

结局	研究数量和类型	事件数/总数	率（95%CI）	证据质量
B 细胞耗竭（随访≥12 个月）				
人群：FRNS/SDNS+[95,135]；干预：利妥昔单抗（单剂 RTX 375mg/m^2，最大 500mg）；1 项病例系列报道[95]，1 项队列研究[135]；38 例；基线、治疗后 2–7 天及 1、3、6、9、12 月监测 B 淋巴细胞[95]，基线、治疗后 1 周及之后的每 1-3 月监测 B 淋巴细胞[135]；伴有 IS 巩固治疗[95]，部分伴有 IS 巩固治疗[135]				
RTX 后 1 周	1 篇病例系列报道[95]<br>1 篇队列研究[135]	24/28[95]<br>9/10[135]	/	极低
人群：FRNS/SDNS（部分 CNI 或 MMF 其他免疫抑制剂使用后）[340]，FRNS/SDNS+[95,431];干预：利妥昔单抗（单剂 RTX 375mg/m^2，最大 500mg）；1 项 RCT 研究[340]，2 项病例系列报道[95,431]；43 例；基线、治疗后 2–7 天及 1、3、6、9、12 月监测 B 淋巴细胞[95]，基线、每月监测 B 淋巴细胞[340]，基线、前半年每月、继之每 3 个月监测 B 淋巴细胞[431]；伴有 IS 巩固治疗[95，431]，不伴有 IS 巩固治疗[340]				
RTX 后 1 月	1 篇 RCT[340]，<br>2 篇病例系列报道[95,431]	28/28[95]<br>15/15[340]<br>16/16[431]	/	极低
人群：FRNS/SDNS-[337]；干预：利妥昔单抗（单剂 RTX 375mg/m^2，最大 500mg）+激素，对照：激素；1 项 RCT 研究；干预组 15 例，对照组 15 例；基线及之后的每月监测 B 淋巴细胞；不伴有 IS 巩固治疗				
RTX 后 1 月	1 篇 RCT[337]	15/15	/	极低
人群：FRNS/SDNS+[151,372];FRNS/SDNS（部分 CNI 或 MMF 其他免疫抑制剂使用后）[193]；干预：利妥昔单抗（4 剂 RTX 375mg/m^2 每周一次×4 次）；1 项 RCT[193]，2 项病例系列报道[151，372]；B 细胞监测频率：基线，前四周每周一次，之后每月一次；CsA3 月内减停,其余 IS 直接停[193]，部分伴有 IS 巩固治疗[372]，未说明是否伴巩固治疗[151]				
第一剂 RTX 后 1 周[151]<br>第一剂 RTX 后 1 月[193，372]	1 篇 RCT[193]，<br>2 篇病例系列报道[151，372]	4/4[151]<br>24/24[193]<br>15/15[372]	/	极低

结局，患儿数量<br>研究类型和数量	相对效应（95%CI）	预期绝对效应值（事件数/例数）对照组	干预组	风险差	证据质量	升降级说明
B 细胞耗竭持续时间（B 细胞重建定义为：外周血 CD19+和（或）CD20+ B 淋巴细胞/总 B 细胞>1% 或者>5cells/mm³）（随访≥12 个月）						
人群：FRNS/SDNS+[133，219,309]，FRNS/SDNS[340],1 项 RCT[340]，2 项病例系列报道[133,219]，1 项非 RCT[309]；干预组：利妥昔（单剂 375mg/m²，最大 500mg）；200 例；RTX 前、RTX 后一周及之后的每月监测 B 淋巴细胞[133]；基线、每月监测 B 淋巴细胞[219,340]；基线、每 1~2 月监测 B 淋巴细胞[309]；伴有 IS 巩固治疗[133,219,309]；不伴有 IS 巩固治疗[340]						
B 细胞耗竭持续时间（随访≥12 个月）	**B 细胞重建的中位时间：**<br>5.5（95%CI：5.1-5.9）月	/	中位 150（范围：43~614）天[133]<br>中位 160（范围：39~311）天[219]<br>中位 174（范围：145~219）天[309]<br>中位 165（范围：120~360）天[340]	/	**极低**	/
人群：FRNS/SDNS+[142]，1 项非 RCT，干预组：利妥昔单抗(单剂 375mg/m²，最大 500mg)+MMF，对照组：利妥昔单抗（单剂 375mg/m²，最大 500mg）+CsA，干预组 16 例，对照组 13 例；RTX 前、RTX 后一周及之后的每月监测 B 淋巴细胞						
B 细胞耗竭持续时间（随访≥12 个月）	/	中位 150 (范围：90–210)天	中位 150 (范围：90–510)天[142]	/	**极低**	/
人群：FRNS/SDNS+[193]，1 项 RCT，干预组：利妥昔单抗（4 剂 375mg/m² 每周一次×4 次），对照组：安慰剂，干预组 24 例，对照组 24 例；B 淋巴细胞监测频率：基线、前 4 周每周一次、之后每月一次；后期巩固治疗：CsA3 个月减停,其它 IS 直接停用						
B 细胞耗竭持续时间（随访≥12 个月）	**B 细胞重建中位时间：**<br>5.0（95%CI：4.5-5.7）月		148(95% CI： 131-170)天	/	**极低**	/
人群：FRNS/SDNS[194]，1 项 RCT，干预组：利妥昔单抗（4 剂 375mg/m² 每周一次×4 次）+MMF，对照组：利妥昔单抗（4 剂 375mg/m² 每周一次×4 次）+安慰剂，干预组 39 例，对照组 39 例；B 淋巴细胞监测频率：基线、前 4 周每周一次、之后 5 个月每月一次、继之每 2 月一次；后期巩固治疗：CsA3 个月减停,其它 IS 直接停用						
B 细胞耗竭持续时间（随访≥12 个月）	/	165.0 (95% CI： 112-217)天	162.0 (95% CI： 53-195)天	/	**极低**	/
B 细胞耗竭持续时间（B 细胞重建定义为：外周血 CD19+和（或）CD20+ B 淋巴细胞/总 B 细胞>3% 或者>15cells/mm³）（随访≥12 个月）						
人群：FRNS/SDNS+[201]，1 项非 RCT，干预组：利妥昔单抗（单剂 375mg/m²，最大 500mg）+MMF，对照组：利妥昔单抗（单剂 375mg/m²，最大 500mg），干预组 9 例，对照组 7 例；在基线及之后的每月监测 B 淋巴细胞						
B 细胞耗竭持续时间（随访≥12 个月）	/	131±72 天	149±33 天	P>0.05	**极低**	/
人群：FRNS/SDNS+[217，431]，FRNS/SDNS-[337]；1 项 RCT[337]，2 项病例系列报道[217,431]，干预组：利妥昔（单剂 375mg/m²，最大 500mg）；41 例；基线、每月监测 B 淋巴细胞[217,337]；基线、前半年每月、继之每 3 月监测 B 淋巴细胞[431]；部分伴有 IS 巩固治疗[217,431]；不伴有 IS 巩固治疗[337]						
B 细胞耗竭持续时间（随访≥12 个月）	/	/	中位 146.5(范围：84~245)天[217]<br>中位 174(范围：120-360)天[337]<br>平均 132.6 天[431]	/	**极低**	/
B 细胞耗竭持续时间（B 细胞重建定义为：外周血 CD19+或 CD20+ B 淋巴细胞>10cells/mm³）（随访≥12 个月）						
人群：FRNS/SDNS+[178]，干预组：利妥昔（单剂 375mg/m²，最大 500mg）；1 项队列研究；35 例；基线、每月监测 B 淋巴细胞；部分伴有 IS						
B 细胞耗竭持续时间（随访≥12 个月）	/	/	中位 174（范围：120~360）天	/	**极低**	/

结局；患儿数量<br>研究类型和数量	相对效应<br>(95%CI)	预期绝对效应值（事件数/例数）			证据质量	升降级说明
		对照组	干预组	风险差		
**B 细胞耗竭持续时间（巩固治疗）**						
**人群：FRNS/SDNS+；干预组：RTX（375mg/m² qw×3 剂）+重复 1 疗程 RTX（375mg/m² qw×3 剂）（基线、RTX 后每 2 月、B 细胞重建后每月检测 B 细胞，CD19+B 淋巴细胞回升至 4.4%±1.1%，再予 1~2 个疗程）；1 项病例系列报道[13]，5 例；不伴 IS**						
随访>12 月	/	首疗程 B 细胞耗竭率 100%（5/5）<br>耗竭时间（7.0±1.0 月）	B 细胞耗竭均持续>15 月	/	极低	/
**人群：FRNS/SDNS+；干预组：RTX（375mg/m²，3 月 1 次×4 次）；1 项病例系列报道[235]，5 例；基线、RTX 后每 3 月检测 B 细胞（CD19+B 淋巴细胞>10 个细胞/mm³ 为重建），不伴 IS**						
随访中位时间 3.2（范围：1.9–3.8）年	/	首疗程 B 细胞耗竭率 80%（4/5）	4 例 B 细胞耗竭持续>21 月	/	极低	/
**人群：FRNS/SDNS+；干预组：利妥昔单抗（375mg/m²）+重复 1 疗程 RTX（基线、RTX 后每 1~2 月检测 B 细胞，CD19+B 淋巴细胞>1%重复 RTX）；对照组：利妥昔单抗，1 项 RCT[309]，干预组 16 例，对照组 45 例，伴 IS**						
随访>24 月	/	首疗程后 B 细胞耗竭率 100%（61/61）<br>中位 174 (范围: 145–219)天	中位时间 239 (范围:171–276)天	/	极低	/
**人群：FRNS/SDNS+；干预组：利妥昔单抗（375mg/m² 1~4 剂）+重复 1~4 疗程 RTX（375mg/m²）（基线及之后每月检测 B 细胞，CD19+B 淋巴细胞>10 个细胞/mm³ 重复 RTX）；1 项队列研究[372]，22 例，伴 IS**						
随访>12 月	/	首疗程后 B 细胞耗竭率 100%（22/22），<br>平均耗竭时间 7.9 月（范围：3.0~15.3 月）	73.7%（14/19）患者再予 1~4 疗程 RTX 后 B 细胞耗竭持续>15 月	/		
**B 细胞耗竭持续时间（复发后再予 RTX）**						
**人群：FRNS/SDNS+；干预组：利妥昔单抗（375mg/m² 1~2 剂）+重复 1~4 疗程 RTX（375mg/m²）（基线及之后每月检测 B 细胞）；1 项队列研究[339]，46 例，IS 减停**						
随访≥12 月	/	46 例首疗程后 B 细胞耗竭时间（195±90）天	多个疗程后 B 细胞耗竭时间（219±142）天	/	极低	**降级因素：**<br>偏倚风险降 2 级<br>不一致性不降级<br>精确性降 1 级<br>发表偏倚不适用<br>间接性不降级<br>**升级因素：无**
**B 细胞耗竭持续时间（B 细胞重建或复发后再予 RTX）**						
**人群：FRNS/SDNS+；干预组：利妥昔单抗（375mg/m² 1~4 剂或 750mg/m² 1~2 剂）+重复 1 疗程 RTX（375mg/m² 1~4 剂或 750mg/m² 1~2 剂）；1 项病例系列报道[83]，346 例，伴 IS**						
随访中位时间 5.9 (四分位：4.3–7.7)年	/	首疗程 RTX 后 B 细胞耗竭率 98.0%（196/200），中位耗竭时间 180 (95% CI：171~ 186)天	第 2 疗程 RTX 后 B 细胞耗竭率 98.4%（182/185），中位耗竭时间 180(95% CI：168~195)天	/	极低	/

Refer to Meta Figure 21, Figure 22, Figure 23, Figure 24, Figure 25, Figure 26

3. Undesirable effects: How substantial are the undesirable anticipated effects?

Detailed judgments: How large are the desirable effects of the intervention taking into account the importance of the outcomes (how much they are valued), and the size of the effect (the likelihood of experiencing a benefit or how much of an improvement individuals would be likely to experience)?

FRNS/SDNS 予 RTX 1~4 剂治疗首剂输注 1 月后 B 细胞耗竭达 < 1%， 5 ~ 6 个月 B 细胞重建，多疗程后 B 细胞耗竭持续时间延长的不良影响有多大？根据结局重要性（受重视的程度）和效应值大小（获益的可能性和个体情况改善的程度）对干预措施的不良影响进行判断

JUDGEMENTS	RESEARCH EVIDENCE	ADDITIONAL CONSIDERATIONS

□ Large<br>□ Moderate<br>☑ **Small**<br>□ Trivial<br>□ Varies<br>□ Uncertain	/	Vote Results : **9/14 chose " Small "** , 5/14 chose "Trivial" .

4. Certainty of the evidence: What is the overall certainty of the evidence of effects?

Detailed judgments: How good an indication does the research provide of the likely effects across all of the critical outcomes; i.e. the likelihood that the effects will be different enough from what the research found that it might affect a decision about the intervention?

FRNS/SDNS 予 RTX 1~4 剂治疗首剂输注 1 月后 B 细胞耗竭达 < 1%， 5 ~ 6 个月 B 细胞重建，多疗程后 B 细胞耗竭持续时间延长有益影响和不良影响相关证据的总体质量？基于 FRNS/SDNS 予 RTX 1~4 剂治疗首剂输注 1 月后 B 细胞耗竭达 < 1%， 5 ~ 6 个月 B 细胞重建，多疗程后 B 细胞耗竭持续时间延长的可能影响，判断干预效果是否会对干预决策产生影响

JUDGEMENTS	RESEARCH EVIDENCE	ADDITIONAL CONSIDERATIONS
☑ **Very low**<br>□ Low<br>□ Moderate<br>□ High<br>□ No included studies	/	Vote Results : **10/14 chose " Very low "** , 2/14 chose " Low " , 2/14 chose "Moderate" .

5. Values: Is there important uncertainty about or variability in how much people value the main outcomes?

Detailed judgments: How much do individuals value each of the main outcomes? Is uncertainty about how much they value each of the outcomes or variability in how much different individual value the outcomes large enough that it could lead to different decisions?

对 FRNS/SDNS 予 RTX 1~4 剂治疗首剂输注 1 月后 B 细胞耗竭达 < 1%， 5 ~ 6 个月 B 细胞重建，多疗程后 B 细胞耗竭持续时间延长重视程度，是否因个体不同而存在不确定性和变化性？个体对 FRNS/SDNS 予 RTX 1~4 剂治疗首剂输注 1 月后 B 细胞耗竭达 < 1%， 5 ~ 6 个月 B 细胞重建，多疗程后 B 细胞耗竭持续时间延长的重视程度有多大？不确定性和变化性是否会导致不同的决策？不确定性体现在对上述推荐的理解程度；变化性体现在对上述推荐重视程度的差异。

JUDGEMENTS	RESEARCH EVIDENCE	ADDITIONAL CONSIDERATIONS
□ Important uncertainty or variability<br>□ Possibly important uncertainty or variability<br>□ Probably no important uncertainty or variability<br>☑ **No Important**	/	Vote Results : 1/14 chose " Probably no Important uncertainty or variability " , **13/14 chose " No Important uncertainty or variability" .**

uncertainty or variability		

6. Balance of effects: Does the balance between desirable and undesirable effects favour the intervention or the comparison?

Detailed judgments: What is the balance between the desirable and undesirable effects, taking into account how much individuals value the main outcome, how substantial the desirable and undesirable effects are, the certainty of those estimates, discount rates, risk aversion and risk seeking?
权衡 FRNS/SDNS 予 RTX 1~4 剂治疗首剂输注 1 月后 B 细胞耗竭达 < 1%， 5 ~ 6 个月 B 细胞重建，多疗程后 B 细胞耗竭持续时间延长利弊。从以下方面权衡利弊：个体对 FRNS/SDNS 予 RTX 1~4 剂治疗首剂输注 1 月后 B 细胞耗竭达 < 1%， 5 ~ 6 个月 B 细胞重建，多疗程后 B 细胞耗竭持续时间延长重视程度；利多大？；弊多大？；估计值的精确性；信心有多大？；风险多大？；可能规避风险么？

JUDGEMENTS	RESEARCH EVIDENCE	ADDITIONAL CONSIDERATIONS
□ Favours the comparison<br>□ Probably favours the comparison **(disadvantages outweigh advantages)**<br>□ Does not favour either the intervention or the comparison **(advantages equal disadvantages)**<br>□ Probably favours the intervention **(advantages outweigh disadvantages)**<br>☑ **Favours the intervention**<br>□ Varies<br>□ Uncertain	/	Vote Results： 3/14 chose "Probably favours the intervention", **11/14 chose "Favours the intervention".**

7. Resources required: How large are the resource requirements (costs)?

Detailed judgments: How large is the cost of the difference in resource use between the intervention and comparision?
FRNS/SDNS 予 RTX 1~4 剂治疗首剂输注 1 月后 B 细胞耗竭达 < 1%， 5 ~ 6 个月 B 细胞重建，多疗程后 B 细胞耗竭持续时间延长成本支出有多大？支出成本有多大？

JUDGEMENTS	RESEARCH EVIDENCE	ADDITIONAL CONSIDERATIONS

□ Large costs<br>□ Moderate costs<br>☑ **Negligible costs or savings**<br>□ Moderate savings<br>□ Large savings<br>□ Varies<br>□ Uncertain	Refer to recommendation 18	Vote Results: 1/14 chose "Large costs", 1/14 chose "Moderate costs", **8/14 chose "Negligible costs or savings"**, 4/14 chose "Don't know".

8. Certainty of evidence of required resources: What is the certainty of the evidence of resource requirements (costs)?

Detailed judgments: How certain is the evidence of a difference for each type of resource use (eg. drugs, hospitalizations) and the cost of resources?

成本支出的证据质量如何？对 FRNS/SDNS 予 RTX 1~4 剂治疗首剂输注 1 月后 B 细胞耗竭达 < 1%， 5～6 个月 B 细胞重建，多疗程后 B 细胞耗竭持续时间延长成本支出（包括药物、住院等费用）相关证据的确定性。

JUDGEMENTS	RESEARCH EVIDENCE	ADDITIONAL CONSIDERATIONS
□ Very low<br>□ Low<br>□ Moderate<br>□ High<br>☑ **No included studies**	Refer to recommendation 18	Vote Results: **5/14 chose "Very low"**, 4/14 chose "Low", 2/14 chose "Moderate", 3/14 chose "No included studies".<br><br>No literature was actually included.

9. Cost-effectiveness: Does the cost-effectiveness of the intervention favour the intervention or the comparison?

Detailed judgments: Is the intervention cost-effective, taking into account uncertainty about or variability in the costs, uncertainty about or variability in the net benefit, sensitivity analyses, and the reliability and applicability of the economic evaluation?

对 FRNS/SDNS 予 RTX 1~4 剂治疗首剂输注 1 月后 B 细胞耗竭达 < 1%， 5～6 个月 B 细胞重建，多疗程后 B 细胞耗竭持续时间延长干预的成本效益。从以下方面分析干预的成本效益：对支出成本的不确定性或变化性；对净利润的不确定性或变化性；敏感性分析；经济评估的可靠性和适用性。

JUDGEMENTS	RESEARCH EVIDENCE	ADDITIONAL CONSIDERATIONS

□ Favours the comparison<br>□ Probably favours the comparison<br>□ Does not favour either the intervention or the comparison<br>□ Probably favours the intervention<br>☑ **Favours the intervention**<br>□ Varies<br>□ No included studies	Refer to recommendation 18	Vote Results : 3/14 chose " Probably favours the intervention " , **11/14 chose "Favours the intervention" .**

10. EQUITY: What would be the impact on health equity?

Detailed judgments: Are there plausible reasons for anticipating differences in the relative effectiveness of the intervention for disadvantaged subgroups or different baseline conditions across disadvantaged subgroups that affect the absolute effectiveness of the intervention or the importance of the problem?
对卫生公平性的影响？FRNS/SDNS 予 RTX 1~4 剂治疗首剂输注 1 月后 B 细胞耗竭达＜1%， 5～6 个月 B 细胞重建，多疗程后 B 细胞耗竭持续时间延长干预的相对有效性在弱势人群中有所降低，对此是否有合理的解释？弱势人群的不同基线水平会影响干预的绝对有效性或研究问题的重要性，对此是否有合理的解释？

JUDGEMENTS	RESEARCH EVIDENCE	ADDITIONAL CONSIDERATIONS
□ Reduced<br>☑ **Probably Reduced**<br>□ Probably no impact<br>□ Probably increased<br>□ Increased<br>□ Varies<br>□ Uncertain	/	Vote Results : **9/14 chose " Probably Reduced "** , 1/14 chose "Probably no impact", 4/14 chose "Don' t know" .

11. ACCEPTABILITY: Is the intervention acceptable to key stakeholders?

Detailed judgments: Are key stakeholders likely not to accept the distribution of the benefits, harms and costs; or the costs or undesirable effects in the short term for desirable effects (benefits) in the future? Are they likely to disagree with the values attached to the desirable or undesirable effects, or not to accept the diagnostic intervention because of ethical concerns?
患儿及其家长是否接受 FRNS/SDNS 予 RTX 1~4 剂治疗首剂输注 1 月后 B 细胞耗竭达＜1%， 5～6 个月 B 细胞重建，多疗程后 B 细胞耗竭持续时间延长患儿及其家长是否会接受干预带来的获益、伤害及支出，或远期获益带来的短期内的不良反应；是否会对利弊判断背后的价值观念持反对意见；是否会出于伦理考虑拒绝诊断性治疗。

JUDGEMENTS	RESEARCH EVIDENCE	ADDITIONAL CONSIDERATIONS

☐ No<br>☐ Probably no<br>☐ Probably yes<br>☑ **Yes**<br>☐ Varies<br>☐ Uncertain	/	Vote Results : 4/14 chose "Probably Yes", **10/14 chose "Yes"**.

12. FEASABILITY: Is the option feasible to implement?

Detailed judgments: Is it feasible to sustain use of the intervention and to address potential barriers to using it?

JUDGEMENTS	RESEARCH EVIDENCE	ADDITIONAL CONSIDERATIONS
☐ No<br>☐ Probably no<br>☐ Probably yes<br>☑ **Yes**<br>☐ Varies<br>☐ Uncertain	/	Vote Results : 2/14 chose "Probably Yes", **12/14 chose "Yes"**.

SUMMARY OF JUDGEMENTS

<table>
<tr><th>CRITERIA</th><th colspan="8">DECISION</th></tr>
<tr><td>1. PROBLEM</td><td colspan="2">No</td><td>Probably no</td><td colspan="2">Probably Yes</td><td>Yes</td><td>Varies</td><td>Don’ t know</td></tr>
<tr><td>2. BENEFITS</td><td colspan="2">Trivial</td><td>Small</td><td colspan="2">Moderate</td><td>Large</td><td>Varies</td><td>Don’ t know</td></tr>
<tr><td>3. HARMS</td><td colspan="2">Large</td><td>Moderate</td><td colspan="2">Small</td><td>Trivial</td><td>Varies</td><td>Don’ t know</td></tr>
<tr><td>4. QUALITY OF EVIDENCE</td><td colspan="2">Very low</td><td>Low</td><td colspan="2">Moderate</td><td>High</td><td colspan="2">No included studies</td></tr>
<tr><td>5. VALUES</td><td colspan="2">Important uncertainty or variability</td><td>Probably Important uncertainty or variability</td><td colspan="2">Probably no Important uncertainty or variability</td><td>No Important uncertainty or variability</td><td colspan="2">Varies</td></tr>
<tr><td>6. BALANCE OF EFFECTS</td><td>Favours the comparison</td><td>Probably favours the comparison</td><td colspan="2">Does not favour either the intervention or the comparison</td><td>Probably favours the intervention</td><td>Favours the intervention</td><td>Varies</td><td>Don’ t know</td></tr>
<tr><td>7. RESOURCES REQUIRED</td><td>Large costs</td><td>Moderate costs</td><td colspan="2">Negligible costs or savings</td><td>Large savings</td><td>Moderate savings</td><td>Varies</td><td>Don’ t know</td></tr>
<tr><td>8. CERTAINTY OF EVIDENCE OF REQUIRED RESOURCES</td><td>Very low</td><td>Low</td><td colspan="2">Moderate</td><td colspan="2">High</td><td colspan="2">No included studies</td></tr>
<tr><td>9. COST-EFFECTIVENESS</td><td>Favours the comparison</td><td>Probably favours the comparison</td><td colspan="2">Does not favour either the intervention or the comparison</td><td>Probably favours the intervention</td><td>Favours the intervention</td><td>Varies</td><td>Don’ t know</td></tr>
<tr><td>10. EQUITY</td><td>Reduced</td><td>Probably Reduced</td><td colspan="2">Probably no impact</td><td>Probably Increased</td><td>Increased</td><td>Varies</td><td>Don’ t know</td></tr>
<tr><td>11. ACCEPTABILITY</td><td>No</td><td>Probably no</td><td colspan="2">Probably Yes</td><td colspan="2">Yes</td><td>Varies</td><td>Don’ t know</td></tr>
<tr><td>12. FEASIBILITY</td><td>No</td><td>Probably no</td><td colspan="2">Probably Yes</td><td colspan="2">Yes</td><td>Varies</td><td>Don’ t know</td></tr>
</table>

TYPE OF RECOMMENDATION

Strong recommendation against the intervention	Conditional recommendation against the intervention	Conditional recommendation for either the intervention or the comparison	Conditional recommendation for the intervention	Strong recommendation for the intervention
○	○	○	○	○

CONCLUSIONS

Reason for recommendation

B cell depletion and reconstitution are important reference indicators.

Recommendation(text)

In children with FRNS/SDNS receiving 1~4 doses of RTX treatment, B-cell depletion is achieved at one month after the first dose, B-cell reconstitution occurs at 5~6 months, and multiple courses of RTX treatment slightly prolongs the duration of B-cell depletion. (1D)

Implementation considerations

Multiple courses include cases of relapse.

Recommendation 13 (deleted): Reconstitution of memory B cells and detection of Th17 cells could be used as a reference index to predict relapse.

Is it feasible to take peripheral CD19+/CD20+ B cell count as a relapse predicting indicator in children aged 1-18 years with SSNS after RTX treatment?	
**STUDY TYPE**	cohort
**POPULATION**	FRNS/SDNS
**RELAPSE GROUP**	Relapse within 12 months after the first course of RTX treatment (375mg/m2, qw×2w)
**NON-RELAPSED GROUP**	No relapse within 12 months after the first course of RTX treatment (375mg/m2, qw×2w)
**MAIN OUTCOMES**	Which B cell subgroup predicts relapse
**PERSPECTIVE**	Clinicians, social workers and parents of children with FRNS/SDNS
**SETTING**	Hospital
**CONFLICT OF INTERESTS**	No

ASSESSMENT

1. PROBLEM: Is the problem a priority?<br>Detailed judgments: Detailed judgments: Detailed judgments: The more serious or urgent a problem is, the more likely it is that an option that addresses the problem will be a priority.<br>外周血 CD20+/CD19+B 细胞数量作为预测疾病复发的问题具有优先性么？根据问题的严重性和紧急性程度判断其是否具有优先性。严重性和紧急性程度越高，优先性越大		
**JUDGEMENTS**	**RESEARCH EVIDENCE**	**ADDITIONAL CONSIDERATIONS**
□ No<br>□ Probably no<br>□ Probably yes<br>□ Yes<br>□ Varies<br>□ Uncertain	/	
**2. Desirable effects: How substantial are the desirable anticipated effects?**		

Detailed judgments: How large are the desirable effects of the intervention taking into account the importance of the outcomes (how much they are valued), and the size of the effect (the likelihood of experiencing a benefit or how much of an improvement individuals would be likely to experience)?<br>记忆 B 细胞或 Th17 作为预测疾病复发的有益影响有多大？根据结局重要性（受重视的程度）和效应值大小（获益的可能性和个体情况改善的程度）对预测的有益影响进行判断		
**JUDGEMENTS**	**RESEARCH EVIDENCE**	**ADDITIONAL CONSIDERATIONS**
□ Trivial<br>□ Small<br>□ Moderate<br>□ Large<br>□ Varies<br>□ Uncertain		

3. Undesirable effects: How substantial are the undesirable anticipated effects?

Detailed judgments: How large are the desirable effects of the intervention taking into account the importance of the outcomes (how much they are valued), and the size of the effect (the likelihood of experiencing a benefit or how much of an improvement individuals would be likely to experience)?<br>记忆 B 细胞或 Th17 作为预测疾病复发的不良影响有多大？根据结局重要性（受重视的程度）和效应值大小（获益的可能性和个体情况改善的程度）对预测的不良影响进行判断		
**JUDGEMENTS**	**RESEARCH EVIDENCE**	**ADDITIONAL CONSIDERATIONS**
□ Large<br>□ Moderate<br>□ Small<br>□ Trivial<br>□ Varies<br>□ Uncertain	/	

4. Certainty of the evidence: What is the overall certainty of the evidence of effects?

Detailed judgments: How good an indication does the research provide of the likely effects across all of the critical outcomes; i.e. the likelihood that the effects will be different enough from what the research found that it might affect a decision about the intervention?<br>记忆 B 细胞或 Th17 作为预测疾病复发有益影响和不良影响相关证据的总体质量？基于记忆 B 细胞或 Th17 作为预测疾病复发指标的可能影响，判断预测效果是否会对预测决策产生影响		
**JUDGEMENTS**	**RESEARCH EVIDENCE**	**ADDITIONAL CONSIDERATIONS**
□ Very low<br>□ Low<br>□ Moderate<br>□ High<br>□ No included studies	/	

5. Values: Is there important uncertainty about or variability in how much people value the main outcomes?

Detailed judgments: How much do individuals value each of the main outcomes? Is uncertainty about how much they value each of the outcomes or variability in how much different individual value the outcomes large enough that it could lead to different decisions?

对记忆 B 细胞或 Th17 作为预测疾病复发的重视程度，是否因个体不同而存在不确定性和变化性？个体对记忆 B 细胞或 Th17 作为预测疾病复发的重视程度有多大？不确定性和变化性是否会导致不同的决策？不确定性体现在对上述推荐的理解程度；变化性体现在对上述推荐重视程度的差异。

JUDGEMENTS	RESEARCH EVIDENCE	ADDITIONAL CONSIDERATIONS
□ Important uncertainty or variability<br>□ Possibly important uncertainty or variability<br>□ Probably no important uncertainty or variability<br>□ No important uncertainty or variability	/	

6. Balance of effects: Does the balance between desirable and undesirable effects favour the intervention or the comparison?

Detailed judgments: What is the balance between the desirable and undesirable effects, taking into account how much individuals value the main outcome, how substantial the desirable and undesirable effects are, the certainty of those estimates, discount rates, risk aversion and risk seeking?

权衡记忆 B 细胞或 Th17 作为预测疾病复发利弊。从以下方面权衡利弊：个体对记忆 B 细胞或 Th17 作为预测疾病复发的重视程度；利多大？；弊多大？；估计值的精确性；信心有多大？；风险多大？；可能规避风险么？

JUDGEMENTS	RESEARCH EVIDENCE	ADDITIONAL CONSIDERATIONS
□ Favours the comparison<br>□ Probably favours the comparison **(disadvantages outweigh advantages)**<br>□ Does not favour either the intervention or the comparison **(advantages equal disadvantages)**<br>□ Probably favours the intervention **(advantages outweigh disadvantages)**<br><br>□ Favours the intervention	/	

◻ Varies<br>◻ Uncertain		

7. Resources required: How large are the resource requirements (costs)?

Detailed judgments: How large is the cost of the difference in resource use between the intervention and comparision?

记忆 B 细胞或 Th17 作为预测疾病复发成本支出有多大？支出成本有多大？

JUDGEMENTS	RESEARCH EVIDENCE	ADDITIONAL CONSIDERATIONS
◻ Large costs<br>◻ Moderate costs<br>◻ Negligible costs or savings<br>◻ Moderate savings<br>◻ Large savings<br>◻ Varies<br>◻ Uncertain	/	

8. Certainty of evidence of required resources: What is the certainty of the evidence of resource requirements (costs)?

Detailed judgments: How certain is the evidence of a difference for each type of resource use (eg. drugs, hospitalizations) and the cost of resources?

成本支出的证据质量如何？对记忆 B 细胞或 Th17 作为预测疾病复发成本支出（包括药物、住院等费用）相关证据的确定性。

JUDGEMENTS	RESEARCH EVIDENCE	ADDITIONAL CONSIDERATIONS
◻ Very low<br>◻ Low<br>◻ Moderate<br>◻ High<br>◻ No included studies	/	

9. Cost-effectiveness: Does the cost-effectiveness of the intervention favour the intervention or the comparison?

Detailed judgments: Is the intervention cost-effective, taking into account uncertainty about or variability in the costs, uncertainty about or variability in the net benefit, sensitivity analyses, and the reliability and applicability of the economic evaluation?<br>成本效益分析更倾向于记忆 B 细胞或 Th17 作为预测疾病复发指标。从以下方面分析预测的成本效益：对支出成本的不确定性或变化性；对净利润的不确定性或变化性；敏感性分析；经济评估的可靠性和适用性。		
**JUDGEMENTS**	**RESEARCH EVIDENCE**	**ADDITIONAL CONSIDERATIONS**
□ Favours the comparison<br>□ Probably favours the comparison<br>□ Does not favour either the intervention or the comparison<br>□ Probably favours the intervention<br>□ Favours the intervention<br>□ Varies<br>□ No included studies	/	

10. EQUITY: What would be the impact on health equity?

Detailed judgments: Are there plausible reasons for anticipating differences in the relative effectiveness of the intervention for disadvantaged subgroups or different baseline conditions across disadvantaged subgroups that affect the absolute effectiveness of the intervention or the importance of the problem?
对卫生公平性的影响？记忆 B 细胞或 Th17 作为预测疾病复发是否在弱势人群中有所降低，对此是否有合理的解释？弱势人群的不同基线水平会影响预测的绝对有效性或研究问题的重要性，对此是否有合理的解释？

**JUDGEMENTS**	**RESEARCH EVIDENCE**	**ADDITIONAL CONSIDERATIONS**
□ Reduced<br>□ Probably reduced<br>□ Probably no impact<br>□ Probably increased<br>□ Increased<br>□ Varies<br>□ Uncertain	/	

11. ACCEPTABILITY: Is the intervention acceptable to key stakeholders?

Detailed judgments: Are key stakeholders likely not to accept the distribution of the benefits, harms and costs; or the costs or undesirable effects in the short term for desirable effects (benefits) in the future? Are they likely to disagree with the values attached to the desirable or undesirable effects, or not to accept the diagnostic intervention because of ethical concerns?
患儿及其家长是否接受记忆 B 细胞或 Th17 作为预测疾病复发的指标。患儿及其家长是否会接受干预带来的获益、伤害及支出，或远期获益带来的短期内的不良反应；是否会对利弊判断背后的价值观念持反对意见；是否会出于伦理考虑拒绝诊断性治疗。

**JUDGEMENTS**	**RESEARCH EVIDENCE**	**ADDITIONAL CONSIDERATIONS**

☐ No<br>☐ Probably no<br>☐ Probably yes<br>☐ Yes<br>☐ Varies<br>☐ Uncertain	/	

12. FEASABILITY: Is the option feasible to implement?

Detailed judgments: Is it feasible to sustain use of the intervention and to address potential barriers to using it?

JUDGEMENTS	RESEARCH EVIDENCE	ADDITIONAL CONSIDERATIONS
☐ No<br>☐ Probably no<br>☐ Probably yes<br>☐ Yes<br>☐ Varies<br>☐ Uncertain	/	

SUMMARY OF JUDGEMENTS

CRITERIA				DECISION				
1. PROBLEM	No		Probably no	Probably Yes		Yes	Varies	Don’t know
2. BENEFITS	Trivial		Small	Moderate		Large	Varies	Don’t know
3. HARMS	Large		Moderate	Small		Trivial	Varies	Don’t know
4. QUALITY OF EVIDENCE	Very low		Low	Moderate		High	No included studies	
5. VALUES	Important uncertainty or variability		Probably Important uncertainty or variability	Probably no Important uncertainty or variability		No Important uncertainty or variability	Varies	
6. BALANCE OF EFFECTS	Favours the comparison	Probably favours the comparison	Does not favour either the intervention or the comparison		Probably favours the intervention	Favours the intervention	Varies	Don’t know
7. RESOURCES REQUIRED	Large costs	Moderate costs	Negligible costs or savings		Large savings	Moderate savings	Varies	Don’t know
8. CERTAINTY OF EVIDENCE OF REQUIRED RESOURCES	Very low	Low	Moderate		High		No included studies	
9. COST-EFFECTIVENESS	Favours the comparison	Probably favours the comparison	Does not favour either the intervention or the comparison		Probably favours the intervention	Favours the intervention	Varies	Don’t know
10. EQUITY	Reduced	Probably Reduced	Probably no impact		Probably Increased	Increased	Varies	Don’t know
11. ACCEPTABILITY	No	Probably no	Probably Yes		Yes		Varies	Don’t know
12. FEASIBILITY	No	Probably no	Probably Yes		Yes		Varies	Don’t know

TYPE OF RECOMMENDATION

Strong recommendation against the intervention	Conditional recommendation against the intervention	Conditional recommendation for either the intervention or the comparison	Conditional recommendation for the intervention	Strong recommendation for the intervention
○	○	○	○	○

CONCLUSIONS

Reason for recommendation

After discussion by experts, the evidence for B cells as a predictor is insufficient and it is hard to answer the question of the specific time point, so this recommendation is deleted, and the evidence for reference is put in the recommendation description of recommendation 14.

Recommendation 14: The relapse rate during B-cell depletion is 1%. (1D)

Is it feasible to take peripheral CD19+/CD20+ B cell count as a relapse predicting indicator in children aged 1-18 years with SSNS after RTX treatment?	
**STUDY TYPE**	Case series report
**POPULATION**	FRNS/SDNS, FRNS/SDNS+, FRNS/SDNS-
**INTERVENTION**	First course of RTX treatment (375mg/m2, qw×(1~4)w)
**COMPARISON**	No
**MAIN OUTCOMES**	Relapse rate during B cell depletion (with a follow-up of ≥ 12 months)
**PERSPECTIVE**	Clinicians, social workers and parents of children with FRNS/SDNS
**SETTING**	Hospital
**CONFLICT OF INTERESTS**	No

ASSESSMENT

1. PROBLEM: Is the problem a priority?

Detailed judgments: Detailed judgments: Detailed judgments: The more serious or urgent a problem is, the more likely it is that an option that addresses the problem will be a priority.
B 细胞耗竭期间复发率问题具有优先性么？根据问题的严重性和紧急性程度判断其是否具有优先性。严重性和紧急性程度越高，优先性越大

JUDGEMENTS	RESEARCH EVIDENCE	ADDITIONAL CONSIDERATIONS
□ No<br>□ Probably no<br>□ Probably yes<br>☑ **Yes**<br>□ Varies<br>□ Uncertain	/	Vote Results : 2/14 chose " Probably Yes ", **12/14 chose "Yes" .**

2. Desirable effects: How substantial are the desirable anticipated effects?

Detailed judgments: How large are the desirable effects of the intervention taking into account the importance of the outcomes (how much they are valued), and the size of the effect (the

likelihood of experiencing a benefit or how much of an improvement individuals would be likely to experience)?
B 细胞耗竭期间复发率 1%的有益影响有多大？根据结局重要性（受重视的程度）和效应值大小（获益的可能性和个体情况改善的程度）对干预措施的有益影响进行判断

JUDGEMENTS

- ☐ Trivial
- ☐ Small
- ☐ Moderate
- ☑ **Large**
- ☐ Varies
- ☐ Uncertain

RESEARCH EVIDENCE

结局	研究数量和类型	事件数/总数	率（95%CI）	证据质量
**B 细胞耗竭期间复发（随访≥12 个月）**				
**人群：FRNS/SDNS+[69,135,142,219,234，275]；FRNS/SDNS[30]；FRNS/SDNS-[54，337]；干预：利妥昔单抗（单剂 RTX 375mg/m²）[135,142,219,337]，利妥昔单抗（RTX 375mg/m², qw×(1-2)w）[30]，利妥昔单抗（RTX 375mg/m², qw×2w）[54,69]，利妥昔单抗（RTX 375mg/m², qw×(2-4)w）[275]，利妥昔单抗（单剂 RTX 375mg/m²，qw×(1-4)w）[234]；4 项 RCT[30,54,275,337]，1 项非 RCT[142]；1 项队列研究[135]；3 项病例系列报道[69,219,234]；280 例；伴有 IS 巩固治疗[142,219]，部分伴有 IS 巩固治疗[135,234]，IS 逐渐减停[30]，仅伴有激素[69,275]，不伴 IS 或激素[54,337]**				
B 细胞耗竭期间复发（随访≥12 个月）	4 项 RCT[30,54,275,337]；<br>1 项非 RCT[142]；<br>1 项队列研究[135]；<br>3 项病例系列报道[69,219,234]	4/35[30]<br>0/60[54]<br>0/16[69]<br>0/9[135]<br>0/29[142]<br>7/78[219]<br>0/20[275]<br>0/18[234]<br>0/15[337]	复发率：<br>1%（95%CI：0～18%）<br>**CD19+/CD20+B 淋巴细胞<1%或 5 个细胞/mm³耗竭率复发：**<br>1%(95%CI：0～19%)	/

Refer to Meta Figure 27

ADDITIONAL CONSIDERATIONS

Vote Results：1/14 chose “Moderate”，**10/14 chose “Large”，** 3/14 chose “Don’t know”.

3. Undesirable effects: How substantial are the undesirable anticipated effects?

Detailed judgments: How large are the desirable effects of the intervention taking into account the importance of the outcomes (how much they are valued), and the size of the effect (the likelihood of experiencing a benefit or how much of an improvement individuals would be likely to experience)?
B 细胞耗竭期间复发率 1%的不良影响有多大？根据结局重要性（受重视的程度）和效应值大小（获益的可能性和个体情况改善的程度）对干预措施的不良影响进行判断

JUDGEMENTS

- ☐ Large
- ☐ Moderate
- ☑ **Small**
- ☐ Trivial
- ☐ Varies
- ☐ Uncertain

RESEARCH EVIDENCE

/

ADDITIONAL CONSIDERATIONS

Vote Results：**6/14 chose “Small”**，5/14 chose “Trivial”，3/14 chose “Don’t know”.

4. Certainty of the evidence: What is the overall certainty of the evidence of effects?

Detailed judgments: How good an indication does the research provide of the likely effects across all of the critical outcomes; i.e. the likelihood that the effects will be different enough from what the research found that it might affect a decision about the intervention?
B 细胞耗竭期间复发率 1%有益影响和不良影响相关证据的总体质量？基于 B 细胞耗竭期间复发率 1%的可能影响，判断预测效果是否会对预测决策产生影响

JUDGEMENTS

- ☑ **Very low**

RESEARCH EVIDENCE

/

ADDITIONAL CONSIDERATIONS

Vote Results：**12/14 chose “Very**

□ Low<br>□ Moderate<br>□ High<br>□ No included studies		**low**", 2/14 chose ".No included studies/无相关证据"
**5. Values: Is there important uncertainty about or variability in how much people value the main outcomes?**<br>Detailed judgments: How much do individuals value each of the main outcomes? Is uncertainty about how much they value each of the outcomes or variability in how much different individual value the outcomes large enough that it could lead to different decisions?<br>对 B 细胞耗竭期间复发率 1%的重视程度，是否因个体不同而存在不确定性和变化性？个体对 B 细胞耗竭期间复发率 1%的重视程度有多大？不确定性和变化性是否会导致不同的决策？不确定性体现在对上述推荐的理解程度；变化性体现在对上述推荐重视程度的差异。		
**JUDGEMENTS**	**RESEARCH EVIDENCE**	**ADDITIONAL CONSIDERATIONS**
□ Important uncertainty or variability<br>□ Possibly important uncertainty or variability<br>□ Probably no important uncertainty or variability<br>☑ **No Important uncertainty or variability**	/	Vote Results : 1/14 chose " Probably Important uncertainty or variability ", 1/14 chose " Probably no Important uncertainty or variability", **12/14 chose "No Important uncertainty or variability".**
**6. Balance of effects: Does the balance between desirable and undesirable effects favour the intervention or the comparison?**<br>Detailed judgments: What is the balance between the desirable and undesirable effects, taking into account how much individuals value the main outcome, how substantial the desirable and undesirable effects are, the certainty of those estimates, discount rates, risk aversion and risk seeking?<br>权衡 B 细胞耗竭期间复发率 1%的利弊。从以下方面权衡利弊：个体对 B 细胞耗竭期间复发率 1%的重视程度；利多大？；弊多大？；估计值的精确性；信心有多大？；风险多大？；可能规避风险么？		
**JUDGEMENTS**	**RESEARCH EVIDENCE**	**ADDITIONAL CONSIDERATIONS**
□ Favours the comparison<br>□ Probably favours the comparison（**弊 Large 于利**）<br>□ Does not favour either the intervention or the comparison（**弊利相当**）<br>□ Probably favours the intervention（**利 Large 于弊**）<br>☑ **Favours the intervention**	/	Vote Results : 2/14 chose " Probably favours the intervention ", **9/14 chose "Favours the intervention",** 1/14 chose " Varies ", 2/14 chose "Don' t know".

□ Varies<br>□ Uncertain		

7. Resources required: How large are the resource requirements (costs)?

Detailed judgments: How large is the cost of the difference in resource use between the intervention and comparision?

B 细胞耗竭期间复发率 1%成本支出有多大？支出成本有多大？

JUDGEMENTS	RESEARCH EVIDENCE	ADDITIONAL CONSIDERATIONS
□ Large costs<br>□ Moderate costs<br>□ Negligible costs or savings<br>□ Moderate savings<br>□ Large savings<br>□ Varies<br>☑ **Don' t know**	/	Vote Results : **6/14 chose "Negligible costs or savings"**, 2/14 chose "Moderate savings", **6/14 chose "Don' t know" .**<br>经考虑， choose "Don' t know"。

8. Certainty of evidence of required resources: What is the certainty of the evidence of resource requirements (costs)?

Detailed judgments: How certain is the evidence of a difference for each type of resource use (eg. drugs, hospitalizations) and the cost of resources?

成本支出的证据质量如何？对 B 细胞耗竭期间复发率 1%成本支出（包括药物、住院等费用）相关证据的确定性。

JUDGEMENTS	RESEARCH EVIDENCE	ADDITIONAL CONSIDERATIONS
□ Very low<br>□ Low<br>□ Moderate<br>□ High<br>☑ **No included studies**	/	Vote Results : **9/14 chose "Very low"**, 1/14 chose "Low", 4/14 chose "No included studies" .<br>实际未有文献纳入。

9. Cost-effectiveness: Does the cost-effectiveness of the intervention favour the intervention or the comparison?

Detailed judgments: Is the intervention cost-effective, taking into account uncertainty about or variability in the costs, uncertainty about or variability in the net benefit, sensitivity analyses, and the reliability and applicability of the economic evaluation?<br>对 B 细胞耗竭期间复发率 1%干预的成本效益分析。从以下方面分析预测的成本效益：对支出成本的不确定性或变化性；对净利润的不确定性或变化性；敏感性分析；经济评估的可靠性和适用性。		
**JUDGEMENTS**	**RESEARCH EVIDENCE**	**ADDITIONAL CONSIDERATIONS**
☐ Favours the comparison<br>☐ Probably favours the comparison<br>☐ Does not favour either the intervention or the comparison<br>☐ Probably favours the intervention<br>☑ **Favours the intervention**<br>☐ Varies<br>☐ No included studies	/	Vote Results : 4/14 chose " Probably favours the intervention " , **6/14 chose "Favours the intervention"**, 1/14 chose "Varies", 3/14 chose "No included studies" .

10. EQUITY: What would be the impact on health equity?

Detailed judgments: Are there plausible reasons for anticipating differences in the relative effectiveness of the intervention for disadvantaged subgroups or different baseline conditions across disadvantaged subgroups that affect the absolute effectiveness of the intervention or the importance of the problem?

对卫生公平性的影响？B 细胞耗竭期间复发率 1%的是否在弱势人群中有所降低，对此是否有合理的解释？弱势人群的不同基线水平会影响预测的绝对有效性或研究问题的重要性，对此是否有合理的解释？

**JUDGEMENTS**	**RESEARCH EVIDENCE**	**ADDITIONAL CONSIDERATIONS**
☐ Reduced<br>☑ **Probably Reduced**<br>☐ Probably no impact<br>☐ Probably increased<br>☐ Increased<br>☐ Varies<br>☐ Uncertain	/	Vote Results : **6/14 chose "Probably Reduced"**, 2/14 chose "Probably no impact", 1/14 chose " Varies " , 5/14 chose "Don' t know" .

11. ACCEPTABILITY: Is the intervention acceptable to key stakeholders?

Detailed judgments: Are key stakeholders likely not to accept the distribution of the benefits, harms and costs; or the costs or undesirable effects in the short term for desirable effects (benefits) in the future? Are they likely to disagree with the values attached to the desirable or undesirable effects, or not to accept the diagnostic intervention because of ethical concerns?

患儿及其家长是否接受 B 细胞耗竭期间复发率 1%。患儿及其家长是否会接受干预带来的获益、伤害及支出，或远期获益带来的短期内的不良反应；是否会对利弊判断背后的价值观念持反对意见；是否会出于伦理考虑拒绝诊断性治疗。

**JUDGEMENTS**	**RESEARCH EVIDENCE**	**ADDITIONAL CONSIDERATIONS**

☐ No<br>☐ Probably no<br>☐ Probably yes<br>☑ **Yes**<br>☐ Varies<br>☐ Uncertain	/	Vote Results : 2/14 chose " Probably Yes " , **9/14 chose " Yes " ,** 3/14 chose " Don' t know" .

**12. FEASABILITY: Is the option feasible to implement?**<br>Detailed judgments: Is it feasible to sustain use of the intervention and to address potential barriers to using it?		
**JUDGEMENTS**	**RESEARCH EVIDENCE**	**ADDITIONAL CONSIDERATIONS**
☐ No<br>☐ Probably no<br>☐ Probably yes<br>☑ **Yes**<br>☐ Varies<br>☐ Uncertain	/	Vote Results : 2/14 chose " Probably Yes " , **11/14 chose "Yes",** 1/14 chose "Varies" .

SUMMARY OF JUDGEMENTS

<table>
<tr><th>CRITERIA</th><th colspan="8">DECISION</th></tr>
<tr><td>1. PROBLEM</td><td colspan="2">No</td><td>Probably no</td><td colspan="2">Probably Yes</td><td>Yes</td><td>Varies</td><td>Don't know</td></tr>
<tr><td>2. BENEFITS</td><td colspan="2">Trivial</td><td>Small</td><td colspan="2">Moderate</td><td>Large</td><td>Varies</td><td>Don't know</td></tr>
<tr><td>3. HARMS</td><td colspan="2">Large</td><td>Moderate</td><td colspan="2">Small</td><td>Trivial</td><td>Varies</td><td>Don't know</td></tr>
<tr><td>4. QUALITY OF EVIDENCE</td><td colspan="2">Very low</td><td>Low</td><td colspan="2">Moderate</td><td>High</td><td colspan="2">No included studies</td></tr>
<tr><td>5. VALUES</td><td colspan="2">Important uncertainty or variability</td><td>Probably Important uncertainty or variability</td><td colspan="2">Probably no Important uncertainty or variability</td><td>No Important uncertainty or variability</td><td colspan="2">Varies</td></tr>
<tr><td>6. BALANCE OF EFFECTS</td><td>Favours the comparison</td><td>Probably favours the comparison</td><td colspan="2">Does not favour either the intervention or the comparison</td><td>Probably favours the intervention</td><td>Favours the intervention</td><td>Varies</td><td>Don't know</td></tr>
<tr><td>7. RESOURCES REQUIRED</td><td>Large costs</td><td>Moderate costs</td><td colspan="2">Negligible costs or savings</td><td>Large savings</td><td>Moderate savings</td><td>Varies</td><td>Don't know</td></tr>
<tr><td>8. CERTAINTY OF EVIDENCE OF REQUIRED RESOURCES</td><td>Very low</td><td>Low</td><td colspan="2">Moderate</td><td colspan="2">High</td><td colspan="2">No included studies</td></tr>
<tr><td>9. COST-EFFECTIVENESS</td><td>Favours the comparison</td><td>Probably favours the comparison</td><td colspan="2">Does not favour either the intervention or the comparison</td><td>Probably favours the intervention</td><td>Favours the intervention</td><td>Varies</td><td>Don't know</td></tr>
<tr><td>10. EQUITY</td><td>Reduced</td><td>Probably Reduced</td><td colspan="2">Probably no impact</td><td>Probably Increased</td><td>Increased</td><td>Varies</td><td>Don't know</td></tr>
<tr><td>11. ACCEPTABILITY</td><td>No</td><td>Probably no</td><td colspan="2">Probably Yes</td><td colspan="2">Yes</td><td>Varies</td><td>Don't know</td></tr>
<tr><td>12. FEASIBILITY</td><td>No</td><td>Probably no</td><td colspan="2">Probably Yes</td><td colspan="2">Yes</td><td>Varies</td><td>Don't know</td></tr>
</table>

TYPE OF RECOMMENDATION

Strong recommendation against the intervention	Conditional recommendation against the intervention	Conditional recommendation for either the intervention or the comparison	Conditional recommendation for the intervention	Strong recommendation for the intervention
○	○	○	○	○

CONCLUSIONS

Reason for recommendation

The relapse rate during B-cell depletion is an important observational indicator.

Recommendation(text)

The relapse rate during B-cell depletion is 1%. (1D)

Implementation considerations

Some hospitals may not be available to detect because of lacking corresponding equipment and technology.

Recommendation 15: RTX treatment does not increase the incidence of serious adverse events and infections in children with SSNS. (1D)

What is the incidence of adverse events in RTX treatment of children with SSNS?	
**STUDY TYPE**	Two-arm study
**POPULATION**	SSNS
**INTERVENTION**	RTX
**COMPARISON**	Placebo, conventional immunosuppressants
**MAIN OUTCOMES**	The incidence of serious adverse events
**PERSPECTIVE**	Clinicians, social workers and parents of children with FRNS/SDNS
**SETTING**	Hospital
**CONFLICT OF INTERESTS**	No

ASSESSMENT

1. PROBLEM: Is the problem a priority?

Detailed judgments: Detailed judgments: Detailed judgments: The more serious or urgent a problem is, the more likely it is that an option that addresses the problem will be a priority. RTX 治疗 SSNS 患儿严重不良事件和感染问题具有优先性么？根据问题的严重性和紧急性程度判断其是否具有优先性。严重性和紧急性程度越高，优先性越大

JUDGEMENTS	RESEARCH EVIDENCE	ADDITIONAL CONSIDERATIONS
□ No<br>□ Probably no<br>□ Probably yes<br>☑ **Yes**<br>□ Varies<br>□ Uncertain	/	Vote Results： 1/14 chose “Probably Yes”， **12/14 chose “Yes”，** 1/14 chose “Don’t know”．

2. Desirable effects: How substantial are the desirable anticipated effects?

Detailed judgments: How large are the desirable effects of the intervention taking into account the importance of the outcomes (how much they are valued), and the size of the effect (the

likelihood of experiencing a benefit or how much of an improvement individuals would be likely to experience)?

Detailed judgments: RTX 治疗 SSNS 患儿未 Increased 严重不良事件和感染发生率的有益影响有多大？根据结局重要性（受重视的程度）和效应值大小（获益的可能性和个体情况改善的程度）对干预措施的有益影响进行判断

JUDGEMENTS

- ☐ Trivial
- ☐ Small
- ☐ Moderate
- ☑ **Large**
- ☐ Varies
- ☐ Uncertain

RESEARCH EVIDENCE

结局；患儿数量；研究类型和数量	OR（95%CI）	绝对效应值（事件数/例数）		证据质量	升降级说明
		对照组	干预组		
不良事件					
干预:RTX；对照：安慰剂；48 例；1 项 RCT[193]					
不良事件发生率	OR=3.13(0.12-80.68)	23/24	24/24		
干预:RTX；对照：常规免疫抑制剂；313 例；4 项 RCT[30，54，275，340]，2 项 NRSI[226，378]					
不良事件发生率	OR=3.77(0.26-55.54)	84/153	106/160		
干预:RTX；对照：安慰剂/常规免疫抑制剂；361 例；5 项 RCT[30，54，193，275，340]，2 项 NRSI[226，378]					
不良事件发生率	OR=4.96(0.36-68.48)	107/177	130/184		
严重不良事件					
干预:RTX；对照：安慰剂；48 例；1 项 RCT[193]					
严重不良事件发生率	OR=2.14(0.63-7.33)	6/24	10/24		
干预:RTX；对照：常规免疫抑制剂；214 例；3 项 RCT[30,54,275]					
严重不良事件发生率	OR=0.90(0.18-4.35)	3/98	4/116		
干预:RTX；对照：安慰剂/常规免疫抑制剂；262 例；4 项 RCT[30,54,193,275]					
严重不良事件发生率	OR=1.56(0.59-4.17)	9/122	14/140		
中性粒细胞减少					
干预:RTX；对照：安慰剂；48 例；1 项 RCT[193]					
中性粒细胞减少发生率	OR=8.47(1.12-64.20)	0/24	4/24		
干预:RTX；对照：他克莫司；40 例；1 项 RCT[275]					
中性粒细胞减少发生率	OR=7.39(0.15-372.38)	0/20	1/20		
淋巴细胞减少					
干预:RTX；对照：安慰剂；48 例；1 项 RCT[193]					
淋巴细胞减少发生率	OR=1.00(0.22-4.49)	4/24	4/24		
干预:RTX；对照：他克莫司；40 例；1 项 RCT[275]					
淋巴细胞减少发生率	OR=0.14(0.00-6.82)	1/20	0/20		
感染					
干预:RTX；对照：安慰剂；48 例；1 项 RCT[193]					
感染发生率	OR=7.67(0.85-69.54)	18/24	23/24		
干预:RTX；对照：常规免疫抑制剂；94 例；2 项 RCT[30,275]					
感染发生率	OR=2.83(0.69-11.62)	23/38	33/56		

结局	研究数量和类型	事件数/总数	率（95%CI）	证据质量
中性粒细胞减少	11 项病例系列报告[66,141,142,159,215,273,336,339,393,398,408]	19/369	5%（3%~8%）	

Refer to Meta Figure 28, Figure 29, Figure 30, Figure 31

ADDITIONAL CONSIDERATIONS

Vote Results：1/14 chose “Trivial”, 2/14 chose “ Moderate ”, **11/14 chose “Yes”.**

3. Undesirable effects: How substantial are the undesirable anticipated effects?

Detailed judgments: How large are the desirable effects of the intervention taking into account the importance of the outcomes (how much they are valued), and the size of the effect (the likelihood of experiencing a benefit or how much of an improvement individuals would be likely to experience)?

RTX 治疗 SSNS 患儿未 Increased 严重不良事件和感染发生率的不良影响有多大？根据结局重要性（受重视的程度）和效应值大小（获益的可能性和个体情况改善的程度）对干预措施的不良影响进行判断

JUDGEMENTS | **RESEARCH EVIDENCE** | **ADDITIONAL CONSIDERATIONS**

□ Large<br>□ Moderate<br>□ Small<br>☑ **Trivial**<br>□ Varies<br>□ Uncertain	/	Vote Results : 1/14 chose "Moderate", 6/14 chose "Small", **7/14 chose "Trivial".**

4. Certainty of the evidence: What is the overall certainty of the evidence of effects?

Detailed judgments: How good an indication does the research provide of the likely effects across all of the critical outcomes; i.e. the likelihood that the effects will be different enough from what the research found that it might affect a decision about the intervention?

RTX 治疗 SSNS 患儿未 Increased 严重不良事件和感染发生率有益影响和不良影响相关证据的总体质量？基于 RTX 治疗 SSNS 患儿未 Increased 严重不良事件和感染发生率的可能影响，判断干预效果是否会对干预决策产生影响

JUDGEMENTS	RESEARCH EVIDENCE	ADDITIONAL CONSIDERATIONS
☑ **Very low**<br>□ Low<br>□ Moderate<br>□ High<br>□ No included studies	/	Vote Results : **13/14 chose "Very low",** 1/14 chose " No included studies".

5. Values: Is there important uncertainty about or variability in how much people value the main outcomes?

Detailed judgments: How much do individuals value each of the main outcomes? Is uncertainty about how much they value each of the outcomes or variability in how much different individual value the outcomes large enough that it could lead to different decisions?

对 RTX 治疗 SSNS 患儿未 Increased 严重不良事件和感染发生率的重视程度，是否因个体不同而存在不确定性和变化性？个体对 RTX 治疗 SSNS 患儿未 Increased 严重不良事件和感染发生率的重视程度有多大？不确定性和变化性是否会导致不同的决策？不确定性体现在对上述推荐的理解程度；变化性体现在对上述推荐重视程度的差异。

JUDGEMENTS	RESEARCH EVIDENCE	ADDITIONAL CONSIDERATIONS
□ Important uncertainty or variability<br>□ Possibly important uncertainty or variability<br>□ Probably no important uncertainty or variability<br>☑ **No Important uncertainty or variability**	/	Vote Results: 2/14 chose "Probably no Important uncertainty or variability", **12/14 chose " No Important uncertainty or variability".**

6. Balance of effects: Does the balance between desirable and undesirable effects favour the intervention or the comparison?

Detailed judgments: What is the balance between the desirable and undesirable effects, taking into account how much individuals value the main outcome, how substantial the desirable and undesirable effects are, the certainty of those estimates, discount rates, risk aversion and risk seeking?
权衡 RTX 治疗 SSNS 患儿未 Increased 严重不良事件和感染发生率利弊。从以下方面权衡利弊：个体对上述推荐的重视程度；利多大？；弊多大？；估计值的精确性；信心有多大？；风险多大？；可能规避风险么？

JUDGEMENTS	RESEARCH EVIDENCE	ADDITIONAL CONSIDERATIONS
□ Favours the comparison<br>□ Probably favours the comparison **(弊 Large 于利)**<br>□ Does not favour either the intervention or the comparison **(弊利相当)**<br>□ Probably favours the intervention **(利 Large 于弊)**<br>☑ **Favours the intervention**<br>□ Varies<br>□ Uncertain	/	Vote Results： 1/14 chose “Probably favours the intervention”， **13/14 chose “Favours the intervention”**.

7. Resources required: How large are the resource requirements (costs)?

Detailed judgments: How large is the cost of the difference in resource use between the intervention and comparision?
RTX 治疗 SSNS 患儿未 Increased 严重不良事件和感染发生率成本支出有多大？支出成本有多大？

JUDGEMENTS	RESEARCH EVIDENCE	ADDITIONAL CONSIDERATIONS
□ Large costs<br>□ Moderate costs<br>☑ **Negligible costs or savings**<br>□ Moderate savings<br>□ Large savings<br>□ Varies<br>□ Uncertain	Refer to recommendation 18	Vote Results : **9/14 chose “Negligible costs or savings”**, 1/14 chose “Moderate savings”, 4/14 chose “Don’t know”.

8. Certainty of evidence of required resources: What is the certainty of the evidence of resource requirements (costs)?

Detailed judgments: How certain is the evidence of a difference for each type of resource use (eg. drugs, hospitalizations) and the cost of resources?
成本支出的证据质量如何？对 RTX 治疗 SSNS 患儿未 Increased 严重不良事件和感染发生率成本支出（包括药物、住院等费用）相关证据的确定性。

JUDGEMENTS	RESEARCH EVIDENCE	ADDITIONAL CONSIDERATIONS
☑ **Trivial**<br>□ Low<br>□ Moderate<br>□ High<br>□ No included studies	Refer to recommendation 18	Vote Results： **7/14 chose “Very low”**，3/14 chose “Low”，1/14 chose “Moderate”，3/14 chose “No included studies”．

9. Cost-effectiveness: Does the cost-effectiveness of the intervention favour the intervention or the comparison?

Detailed judgments: Is the intervention cost-effective, taking into account uncertainty about or variability in the costs, uncertainty about or variability in the net benefit, sensitivity analyses, and the reliability and applicability of the economic evaluation?
RTX 治疗 SSNS 患儿未 Increased 严重不良事件和感染发生率干预的成本效益分析 从以下方面分析干预的成本效益：对支出成本的不确定性或变化性；对净利润的不确定性或变化性；敏感性分析；经济评估的可靠性和适用性。

JUDGEMENTS	RESEARCH EVIDENCE	ADDITIONAL CONSIDERATIONS
□ Favours the comparison<br>□ Probably favours the comparison<br>□ Does not favour either the intervention or the comparison<br>□ Probably favours the intervention<br>☑ **Favours the intervention**<br>□ Varies<br>□ No included studies	Refer to recommendation 18	Vote Results： **14/14 chose “Favours the intervention”．**

10. EQUITY: What would be the impact on health equity?

Detailed judgments: Are there plausible reasons for anticipating differences in the relative effectiveness of the intervention for disadvantaged subgroups or different baseline conditions across disadvantaged subgroups that affect the absolute effectiveness of the intervention or the importance of the problem?

对卫生公平性的影响？RTX 治疗 SSNS 患儿未 Increased 严重不良事件和感染发生率在弱势人群中是否有所降低，对此是否有合理的解释？弱势人群的不同基线水平会影响干预的绝对有效性或研究问题的重要性，对此是否有合理的解释？

JUDGEMENTS	RESEARCH EVIDENCE	ADDITIONAL CONSIDERATIONS
☐ Reduced<br>☑ **Probably Reduced**<br>☐ Probably no impact<br>☐ Probably increased<br>☐ Increased<br>☐ Varies<br>☐ Uncertain	Refer to recommendation 18	Vote Results: **8/14 chose "Probably Reduced"**, 4/14 chose "Probably no impact", 2/14 chose "Probably increased".

11. ACCEPTABILITY: Is the intervention acceptable to key stakeholders?

Detailed judgments: Are key stakeholders likely not to accept the distribution of the benefits, harms and costs; or the costs or undesirable effects in the short term for desirable effects (benefits) in the future? Are they likely to disagree with the values attached to the desirable or undesirable effects, or not to accept the diagnostic intervention because of ethical concerns? 患儿及其家长是否接受 RTX 治疗 SSNS 患儿未 Increased 严重不良事件和感染发生率？患儿及其家长是否会接受干预带来的获益、伤害及支出，或远期获益带来的短期内的不良反应；是否会对利弊判断背后的价值观念持反对意见；是否会出于伦理考虑拒绝诊断性治疗。

JUDGEMENTS	RESEARCH EVIDENCE	ADDITIONAL CONSIDERATIONS
☐ No<br>☐ Probably no<br>☐ Probably yes<br>☑ **Yes**<br>☐ Varies<br>☐ Uncertain	/	Vote Results: 4/14 chose "Probably Yes", **10/14 chose "Yes"**.

12. FEASABILITY: Is the option feasible to implement?

Detailed judgments: Is it feasible to sustain use of the intervention and to address potential barriers to using it?

JUDGEMENTS	RESEARCH EVIDENCE	ADDITIONAL CONSIDERATIONS
☐ No<br>☐ Probably no<br>☐ Probably yes<br>☑ **Yes**<br>☐ Varies<br>☐ Uncertain	/	Vote Results: 1/14 chose "Probably Yes", **13/14 chose "Yes"**.

SUMMARY OF JUDGEMENTS

CRITERIA	DECISION							
1. PROBLEM	No		Probably no	Probably Yes		**Yes**	Varies	Don’t know
2. BENEFITS	Trivial		Small	Moderate		**Large**	Varies	Don’t know
3. HARMS	Large		Moderate	Small		**Trivial**	Varies	Don’t know
4. QUALITY OF EVIDENCE	**Very low**		Low	Moderate		High	No included studies	
5. VALUES	Important uncertainty or variability		Probably Important uncertainty or variability	Probably no Important uncertainty or variability		**No Important uncertainty or variability**	Varies	
6. BALANCE OF EFFECTS	Favours the comparison	Probably favours the comparison	Does not favour either the intervention or the comparison		Probably favours the intervention	**Favours the intervention**	Varies	Don’t know
7. RESOURCES REQUIRED	Large costs	Moderate costs	**Negligible costs or savings**		Large savings	Moderate savings	Varies	Don’t know
8. CERTAINTY OF EVIDENCE OF REQUIRED RESOURCES	**Very low**	Low	Moderate		High		No included studies	
9. COST-EFFECTIVENESS	Favours the comparison	Probably favours the comparison	Does not favour either the intervention or the comparison		Probably favours the intervention	**Favours the intervention**	Varies	Don’t know
10. EQUITY	Reduced	**Probably Reduced**	Probably no impact		Probably Increased	Increased	Varies	Don’t know
11. ACCEPTABILITY	No	Probably no	Probably Yes		**Yes**		Varies	Don’t know
12. FEASIBILITY	No	Probably no	Probably Yes		**Yes**		Varies	Don’t know

TYPE OF RECOMMENDATION

Strong recommendation against the intervention	Conditional recommendation against the intervention	Conditional recommendation for either the intervention or the comparison	Conditional recommendation for the intervention	Strong recommendation for the intervention
○	○	○	○	○

CONCLUSIONS

Reason for recommendation

Serious adverse events with clear definition is an important factor to consider.

Recommendation(text)

RTX treatment does not increase the incidence of serious adverse events and infections in children with SSNS. (1D)

Recommendation 16 (deleted): In children with SSNS receiving RTX treatment, the incidence of hypogammaglobulinemia is 26%, and the incidence of persistent hypogammaglobulinemia is 13%.

What is the incidence of adverse events in RTX treatment of children with SSNS?	
STUDY TYPE	Single-arm study
POPULATION	SSNS
INTERVENTION	RTX
COMPARISON	No
MAIN OUTCOMES	The incidence of hypogammaglobulinemia and persistent hypogammaglobulinemia
PERSPECTIVE	Clinicians, social workers and parents of children with FRNS/SDNS
SETTING	Hospital
CONFLICT OF INTERESTS	No

ASSESSMENT

1. PROBLEM: Is the problem a priority?

Detailed judgments: Detailed judgments: Detailed judgments: The more serious or urgent a problem is, the more likely it is that an option that addresses the problem will be a priority. RTX 治疗 SSNS 患儿中低 IgG 血症和持续性低 IgG 血症问题具有优先性么？根据问题的严重性和紧急性程度判断其是否具有优先性。严重性和紧急性程度越高，优先性越大

JUDGEMENTS	RESEARCH EVIDENCE	ADDITIONAL CONSIDERATIONS
□ No<br>□ Probably no<br>□ Probably yes<br>□ Yes<br>□ Varies<br>□ Uncertain	/	

2. Desirable effects: How substantial are the desirable anticipated effects?

Detailed judgments: How large are the desirable effects of the intervention taking into account the importance of the outcomes (how much they are valued), and the size of the effect (the

likelihood of experiencing a benefit or how much of an improvement individuals would be likely to experience)?
RTX 治疗 SSNS 患儿中 Low IgG 血症 26%和持续性 Low IgG 血症 13%的有益影响有多大？根据结局重要性（受重视的程度）和效应值大小（获益的可能性和个体情况改善的程度）对干预措施的有益影响进行判断

JUDGEMENTS

- ☐ Trivial
- ☐ Small
- ☐ Moderate
- ☐ Large
- ☐ Varies
- ☐ Uncertain

RESEARCH EVIDENCE

结局	研究数量和类型	事件数/总数	率（95%CI）	证据质量
**低 IgG 血症**	11 项病例系列报告[13, 66, 135, 142, 159, 198, 215, 273, 316, 328, 418]	104/393	26%（22%~31%）	
针对性研究亚组	2 项病例系列报告[198, 273]	56/110	51%（42%~60%）	
非针对性研究亚组	9 项病例系列报告[13, 66, 135, 142, 159, 215, 316, 328, 418]	48/283	17%（13%～22%）	
**持续性低 IgG 血症**	3 项病例系列报告[198, 279, 316]	34/257	13%（10%~18%）	
**抗 RTX 抗体生成**	5 项病例系列报告[28, 66, 137, 234, 398]	15/96	16%（10%~24%）	
**血清病**	3 项病例系列报告[137, 336, 339]	6/129	5%（2%~10%）	

Refer to Meta Figure 32, Figure 33, Figure 34, Figure 35

ADDITIONAL CONSIDERATIONS

3. Undesirable effects: How substantial are the undesirable anticipated effects?

Detailed judgments: How large are the desirable effects of the intervention taking into account the importance of the outcomes (how much they are valued), and the size of the effect (the likelihood of experiencing a benefit or how much of an improvement individuals would be likely to experience)?
RTX 治疗 SSNS 患儿中 Low IgG 血症 26%和持续性 Low IgG 血症 13%的不良影响有多大？根据结局重要性（受重视的程度）和效应值大小（获益的可能性和个体情况改善的程度）对干预措施的不良影响进行判断

JUDGEMENTS

- ☐ Large
- ☐ Moderate
- ☐ Small
- ☐ Trivial
- ☐ Varies
- ☐ Uncertain

RESEARCH EVIDENCE

ADDITIONAL CONSIDERATIONS

4. Certainty of the evidence: What is the overall certainty of the evidence of effects?

Detailed judgments: How good an indication does the research provide of the likely effects across all of the critical outcomes; i.e. the likelihood that the effects will be different enough from what the research found that it might affect a decision about the intervention?
RTX 治疗 SSNS 患儿中 Low IgG 血症 26%和持续性 Low IgG 血症 13%有益影响和不良影响相关证据的总体质量？基于 RTX 治疗 SSNS 患儿中 Low IgG 血症 26%和持续性 Low IgG 血症 13%的可能影响，判断干预效果是否会对干预决策产生影响

JUDGEMENTS

- ☐ Very low
- ☐ Low
- ☐ Moderate

RESEARCH EVIDENCE

/

ADDITIONAL CONSIDERATIONS

□ High<br>□ No included studies		

5. Values: Is there important uncertainty about or variability in how much people value the main outcomes?

Detailed judgments: How much do individuals value each of the main outcomes? Is uncertainty about how much they value each of the outcomes or variability in how much different individual value the outcomes large enough that it could lead to different decisions?

对 RTX 治疗 SSNS 患儿中 Low IgG 血症 26%和持续性 Low IgG 血症 13%的重视程度，是否因个体不同而存在不确定性和变化性？个体对 RTX 治疗 SSNS 患儿中 Low IgG 血症 26%和持续性 Low IgG 血症 13%的重视程度有多大？不确定性和变化性是否会导致不同的决策？不确定性体现在对上述推荐的理解程度；变化性体现在对上述推荐重视程度的差异。

JUDGEMENTS	RESEARCH EVIDENCE	ADDITIONAL CONSIDERATIONS
□ Important uncertainty or variability<br>□ Possibly important uncertainty or variability<br>□ Probably no important uncertainty or variability<br>□ No important uncertainty or variability	/	

6. Balance of effects: Does the balance between desirable and undesirable effects favour the intervention or the comparison?

Detailed judgments: What is the balance between the desirable and undesirable effects, taking into account how much individuals value the main outcome, how substantial the desirable and undesirable effects are, the certainty of those estimates, discount rates, risk aversion and risk seeking?

权衡 RTX 治疗 SSNS 患儿中 Low IgG 血症 26%和持续性 Low IgG 血症 13%的利弊从以下方面权衡利弊：个体对上述推荐的重视程度；利多大？；弊多大？；估计值的精确性；信心有多大？；风险多大？；可能规避风险么？

JUDGEMENTS	RESEARCH EVIDENCE	ADDITIONAL CONSIDERATIONS
□ Favours the comparison<br>□ Probably favours the comparison（弊 Large 于利）<br>□ Does not favour either the intervention or the comparison（弊利相当）<br>□ Probably favours the intervention（利 Large 于弊）<br>□ Favours the intervention<br>□ Varies	/	

◻ Uncertain		

7. Resources required: How large are the resource requirements (costs)?

Detailed judgments: How large is the cost of the difference in resource use between the intervention and comparision?

RTX 治疗 SSNS 患儿中 Low IgG 血症 26%和持续性 Low IgG 血症 13%成本支出有多大？支出成本有多大？

JUDGEMENTS	RESEARCH EVIDENCE	ADDITIONAL CONSIDERATIONS
◻ Large costs<br>◻ Moderate costs<br>◻ Negligible costs or savings<br>◻ Moderate savings<br>◻ Large savings<br>◻ Varies<br>◻ Uncertain	Refer to recommendation 18	

8. Certainty of evidence of required resources: What is the certainty of the evidence of resource requirements (costs)?

Detailed judgments: How certain is the evidence of a difference for each type of resource use (eg. drugs, hospitalizations) and the cost of resources?

成本支出的证据质量如何？对 RTX 治疗 SSNS 患儿中 Low IgG 血症 26%和持续性 Low IgG 血症 13%成本支出（包括药物、住院等费用）相关证据的确定性。

JUDGEMENTS	RESEARCH EVIDENCE	ADDITIONAL CONSIDERATIONS
◻ Very low<br>◻ Low<br>◻ Moderate<br>◻ High<br>◻ No included studies	Refer to recommendation 18	

9. Cost-effectiveness: Does the cost-effectiveness of the intervention favour the intervention or the comparison?

Detailed judgments: Is the intervention cost-effective, taking into account uncertainty about or variability in the costs, uncertainty about or variability in the net benefit, sensitivity analyses, and the reliability and applicability of the economic evaluation?
RTX 治疗 SSNS 患儿中 Low IgG 血症 26%和持续性 Low IgG 血症 13%干预的成本效益分析。从以下方面分析干预的成本效益：对支出成本的不确定性或变化性；对净利润的不确定性或变化性；敏感性分析；经济评估的可靠性和适用性。

JUDGEMENTS	RESEARCH EVIDENCE	ADDITIONAL CONSIDERATIONS
□ Favours the comparison<br>□ Probably favours the comparison<br>□ Does not favour either the intervention or the comparison<br>□ Probably favours the intervention<br>□ Favours the intervention<br>□ Varies<br>□ No included studies	Refer to recommendation 18	

10. EQUITY: What would be the impact on health equity?

Detailed judgments: Are there plausible reasons for anticipating differences in the relative effectiveness of the intervention for disadvantaged subgroups or different baseline conditions across disadvantaged subgroups that affect the absolute effectiveness of the intervention or the importance of the problem?
对卫生公平性的影响？RTX 治疗 SSNS 患儿中 Low IgG 血症 26%和持续性 Low IgG 血症 13%在弱势人群中有所升 High，对此是否有合理的解释？弱势人群的不同基线水平会影响干预的绝对有效性或研究问题的重要性，对此是否有合理的解释？

JUDGEMENTS	RESEARCH EVIDENCE	ADDITIONAL CONSIDERATIONS
□ Reduced<br>□ Probably reduced<br>□ Probably no impact<br>□ Probably increased<br>□ Increased<br>□ Varies<br>□ Uncertain	/	

11. ACCEPTABILITY: Is the intervention acceptable to key stakeholders?

Detailed judgments: Are key stakeholders likely not to accept the distribution of the benefits, harms and costs; or the costs or undesirable effects in the short term for desirable effects (benefits) in the future? Are they likely to disagree with the values attached to the desirable or undesirable effects, or not to accept the diagnostic intervention because of ethical concerns?
患儿及其家长是否接受 RTX 治疗 SSNS 患儿中 Low IgG 血症 26%和持续性 Low IgG 血症 13%？患儿及其家长是否会接受干预带来的获益、伤害及支出，或远期获益带来的短期内的不良反应；是否会对利弊判断背后的价值观念持反对意见；是否会出于伦理考虑拒绝诊断性治疗。

JUDGEMENTS	RESEARCH EVIDENCE	ADDITIONAL CONSIDERATIONS
◻ No<br>◻ Probably no<br>◻ Probably yes<br>◻ Yes<br>◻ Varies<br>◻ Uncertain	/	

12. FEASABILITY: Is the option feasible to implement?

Detailed judgments: Is it feasible to sustain use of the intervention and to address potential barriers to using it?

JUDGEMENTS	RESEARCH EVIDENCE	ADDITIONAL CONSIDERATIONS
◻ No<br>◻ Probably no<br>◻ Probably yes<br>◻ Yes<br>◻ Varies<br>◻ Uncertain	/	

SUMMARY OF JUDGEMENTS

<table>
<tr><th>CRITERIA</th><th colspan="8">DECISION</th></tr>
<tr><td>1. PROBLEM</td><td colspan="2">No</td><td>Probably no</td><td colspan="2">Probably Yes</td><td>Yes</td><td>Varies</td><td>Don’ t know</td></tr>
<tr><td>2. BENEFITS</td><td colspan="2">Trivial</td><td>Small</td><td colspan="2">Moderate</td><td>Large</td><td>Varies</td><td>Don’ t know</td></tr>
<tr><td>3. HARMS</td><td colspan="2">Large</td><td>Moderate</td><td colspan="2">Small</td><td>Trivial</td><td>Varies</td><td>Don’ t know</td></tr>
<tr><td>4. QUALITY OF EVIDENCE</td><td colspan="2">Very low</td><td>Low</td><td colspan="2">Moderate</td><td>High</td><td colspan="2">No included studies</td></tr>
<tr><td>5. VALUES</td><td colspan="2">Important uncertainty or variability</td><td>Probably Important uncertainty or variability</td><td colspan="2">Probably no Important uncertainty or variability</td><td>No Important uncertainty or variability</td><td colspan="2">Varies</td></tr>
<tr><td>6. BALANCE OF EFFECTS</td><td>Favours the comparison</td><td>Probably favours the comparison</td><td colspan="2">Does not favour either the intervention or the comparison</td><td>Probably favours the intervention</td><td>Favours the intervention</td><td>Varies</td><td>Don’ t know</td></tr>
<tr><td>7. RESOURCES REQUIRED</td><td>Large costs</td><td>Moderate costs</td><td colspan="2">Negligible costs or savings</td><td>Large savings</td><td>Moderate savings</td><td>Varies</td><td>Don’ t know</td></tr>
<tr><td>8. CERTAINTY OF EVIDENCE OF REQUIRED RESOURCES</td><td>Very low</td><td>Low</td><td colspan="2">Moderate</td><td colspan="2">High</td><td colspan="2">No included studies</td></tr>
<tr><td>9. COST-EFFECTIVENESS</td><td>Favours the comparison</td><td>Probably favours the comparison</td><td colspan="2">Does not favour either the intervention or the comparison</td><td>Probably favours the intervention</td><td>Favours the intervention</td><td>Varies</td><td>Don’ t know</td></tr>
<tr><td>10. EQUITY</td><td>Reduced</td><td>Probably Reduced</td><td colspan="2">Probably no impact</td><td>Probably Increased</td><td>Increased</td><td>Varies</td><td>Don’ t know</td></tr>
<tr><td>11. ACCEPTABILITY</td><td>No</td><td>Probably no</td><td colspan="2">Probably Yes</td><td colspan="2">Yes</td><td>Varies</td><td>Don’ t know</td></tr>
<tr><td>12. FEASIBILITY</td><td>No</td><td>Probably no</td><td colspan="2">Probably Yes</td><td colspan="2">Yes</td><td>Varies</td><td>Don’ t know</td></tr>
</table>

TYPE OF RECOMMENDATION

Strong recommendation against the intervention	Conditional recommendation against the intervention	Conditional recommendation for either the intervention or the comparison	Conditional recommendation for the intervention	Strong recommendation for the intervention
○	○	○	○	○

CONCLUSIONS

Reason for recommendation

After discussion by experts, the evidence of this recommendation is not convincing, so it is deleted and the evidence is displayed in the recommendation description of recommendation 15 for reference.

Recommendation 17 (deleted): In the treatment of RTX in children with SSNS, steroid, antihistamines and acetaminophen are routinely used to prevent possible infusion reactions to RTX. The incidence of infusion reaction is 30%.

What measures should be taken to prevent RTX-induced adverse reactions?	
STUDY TYPE	Sing-arm study
POPULATION	SSNS
INTERVENTION	Steroid, paracetamol, antihistamines, etc.
COMPARISON	No
MAIN OUTCOMES	The incidence of infusion-related reactions
PERSPECTIVE	Clinicians, social workers and parents of children with FRNS/SDNS
SETTING	Hospital
CONFLICT OF INTERESTS	No

ASSESSMENT

1. PROBLEM: Is the problem a priority?

Detailed judgments: The more serious or urgent a problem is, the more likely it is that an option that addresses the problem will be a priority.

RTX 的可能输液反应问题具有优先性么？根据问题的严重性和紧急性程度判断其是否具有优先性。严重性和紧急性程度越高，优先性越大

JUDGEMENTS	RESEARCH EVIDENCE	ADDITIONAL CONSIDERATIONS
□ No<br>□ Probably no<br>□ Probably yes<br>□ Yes<br>□ Varies<br>□ Uncertain	/	

2. Desirable effects: How substantial are the desirable anticipated effects?

Detailed judgments: How large are the desirable effects of the intervention taking into account the importance of the outcomes (how much they are valued), and the size of the effect (the likelihood of experiencing a benefit or how much of an improvement individuals would be likely to experience)?

Detailed judgments: RTX 输液反应发生率达 30%的有益影响有多大？根据结局重要性（受重视的程度）和效应值大小（获益的可能性和个体情况改善的程度）对干预措施的有益影响进行判断

JUDGEMENTS	RESEARCH EVIDENCE	ADDITIONAL CONSIDERATIONS
□ Trivial<br>□ Small<br>□ Moderate<br>□ Large<br>□ Varies<br>□ Uncertain		

3. Undesirable effects: How substantial are the undesirable anticipated effects?

Detailed judgments: How large are the desirable effects of the intervention taking into account the importance of the outcomes (how much they are valued), and the size of the effect (the likelihood of experiencing a benefit or how much of an improvement individuals would be likely to experience)?

RTX 输液反应发生率达 30%的不良影响有多大？根据结局重要性（受重视的程度）和效应值大小（获益的可能性和个体情况改善的程度）对干预措施的不良影响进行判断

JUDGEMENTS	RESEARCH EVIDENCE	ADDITIONAL CONSIDERATIONS
□ Large<br>□ Moderate<br>□ Small<br>□ Trivial<br>□ Varies<br>□ Uncertain	/	

4. Certainty of the evidence: What is the overall certainty of the evidence of effects?

Detailed judgments: How good an indication does the research provide of the likely effects across all of the critical outcomes; i.e. the likelihood that the effects will be different enough from what the research found that it might affect a decision about the intervention?

RTX 输液反应发生率 30%有益影响和不良影响相关证据的总体质量？基于 RTX 输液反应发生率 30%的可能影响，判断干预效果是否会对干预决策产生影响

JUDGEMENTS	RESEARCH EVIDENCE	ADDITIONAL CONSIDERATIONS
□ Very low<br>□ Low<br>□ Moderate<br>□ High	/	

□ No included studies		

5. Values: Is there important uncertainty about or variability in how much people value the main outcomes?

Detailed judgments: How much do individuals value each of the main outcomes? Is uncertainty about how much they value each of the outcomes or variability in how much different individual value the outcomes large enough that it could lead to different decisions?

对 RTX 输液反应发生率 30%重视程度，是否因个体不同而存在不确定性和变化性？个体对 RTX 输液反应发生率 30%的重视程度有多大？不确定性和变化性是否会导致不同的决策？不确定性体现在对上述推荐的理解程度；变化性体现在对上述推荐重视程度的差异。

JUDGEMENTS	RESEARCH EVIDENCE	ADDITIONAL CONSIDERATIONS
□ Important uncertainty or variability<br>□ Possibly important uncertainty or variability<br>□ Probably no important uncertainty or variability<br>□ No important uncertainty or variability	/	

6. Balance of effects: Does the balance between desirable and undesirable effects favour the intervention or the comparison?

Detailed judgments: What is the balance between the desirable and undesirable effects, taking into account how much individuals value the main outcome, how substantial the desirable and undesirable effects are, the certainty of those estimates, discount rates, risk aversion and risk seeking?

权衡 RTX 输液反应发生率 30%利弊。从以下方面权衡利弊：个体对上述推荐的重视程度；利多大？；弊多大？；估计值的精确性；信心有多大？；风险多大？；可能规避风险么？

JUDGEMENTS	RESEARCH EVIDENCE	ADDITIONAL CONSIDERATIONS
□ Favours the comparison<br>□ Probably favours the comparison（弊 Large 于利）<br>□ Does not favour either the intervention or the comparison（弊利相当）<br>□ Probably favours the intervention（利 Large 于弊）<br>□ Favours the intervention<br>□ Varies<br>□ Uncertain	/	

7. Resources required: How large are the resource requirements (costs)?

Detailed judgments: How large is the cost of the difference in resource use between the intervention and comparision?

RTX 输液反应发生率 30%成本支出有多大？支出成本有多大？		
**JUDGEMENTS**	**RESEARCH EVIDENCE**	**ADDITIONAL CONSIDERATIONS**
□ Large costs<br>□ Moderate costs<br>□ Negligible costs or savings<br>□ Moderate savings<br>□ Large savings<br>□ Varies<br>□ Uncertain	/	

8. Certainty of evidence of required resources: What is the certainty of the evidence of resource requirements (costs)?

Detailed judgments: How certain is the evidence of a difference for each type of resource use (eg. drugs, hospitalizations) and the cost of resources?
成本支出的证据质量如何？对 RTX 输液反应发生率 30%成本支出（包括药物、住院等费用）相关证据的确定性。

JUDGEMENTS	RESEARCH EVIDENCE	ADDITIONAL CONSIDERATIONS
□ Very low<br>□ Low<br>□ Moderate<br>□ High<br>□ No included studies	/	

9. Cost-effectiveness: Does the cost-effectiveness of the intervention favour the intervention or the comparison?

Detailed judgments: Is the intervention cost-effective, taking into account uncertainty about or variability in the costs, uncertainty about or variability in the net benefit, sensitivity analyses, and the reliability and applicability of the economic evaluation?
对 RTX 输液反应发生率 30%干预的成本效益分析。从以下方面分析干预的成本效益：对支出成本的不确定性或变化性；对净利润的不确定性或变化性；敏感性分析；经济评估的可靠性和适用性。

JUDGEMENTS	RESEARCH EVIDENCE	ADDITIONAL CONSIDERATIONS

□ Favours the comparison<br>□ Probably favours the comparison<br>□ Does not favour either the intervention or the comparison<br>□ Probably favours the intervention<br>□ Favours the intervention<br>□ Varies<br>□ No included studies	/	

10. EQUITY: What would be the impact on health equity?

Detailed judgments: Are there plausible reasons for anticipating differences in the relative effectiveness of the intervention for disadvantaged subgroups or different baseline conditions across disadvantaged subgroups that affect the absolute effectiveness of the intervention or the importance of the problem?

对卫生公平性的影响？RTX 输液反应发生率 30%是否在弱势人群中有所升 High，对此是否有合理的解释？弱势人群的不同基线水平会影响干预的绝对有效性或研究问题的重要性，对此是否有合理的解释？

JUDGEMENTS	RESEARCH EVIDENCE	ADDITIONAL CONSIDERATIONS
□ Reduced<br>□ Probably reduced<br>□ Probably no impact<br>□ Probably increased<br>□ Increased<br>□ Varies<br>□ Uncertain	/	

11. ACCEPTABILITY: Is the intervention acceptable to key stakeholders?

Detailed judgments: Are key stakeholders likely not to accept the distribution of the benefits, harms and costs; or the costs or undesirable effects in the short term for desirable effects (benefits) in the future? Are they likely to disagree with the values attached to the desirable or undesirable effects, or not to accept the diagnostic intervention because of ethical concerns?

患儿及其家长是否接受 RTX 输液反应发生率 30%？患儿及其家长是否会接受干预带来的获益、伤害及支出，或远期获益带来的短期内的不良反应；是否会对利弊判断背后的价值观念持反对意见；是否会出于伦理考虑拒绝诊断性治疗。

JUDGEMENTS	RESEARCH EVIDENCE	ADDITIONAL CONSIDERATIONS
□ No<br>□ Probably no<br>□ Probably yes<br>□ Yes<br>□ Varies<br>□ Uncertain	/	

12. FEASABILITY: Is the option feasible to implement?

Detailed judgments: Is it feasible to sustain use of the intervention and to address potential barriers to using it?

JUDGEMENTS	RESEARCH EVIDENCE	ADDITIONAL CONSIDERATIONS
☐ No<br>☐ Probably no<br>☐ Probably yes<br>☐ Yes<br>☐ Varies<br>☐ Uncertain	/	

SUMMARY OF JUDGEMENTS

CRITERIA	DECISION							
1. PROBLEM	No		Probably no	Probably Yes		Yes	Varies	Don’ t know
2. BENEFITS	Trivial		Small	Moderate		Large	Varies	Don’ t know
3. HARMS	Large		Moderate	Small		Trivial	Varies	Don’ t know
4. QUALITY OF EVIDENCE	Very low		Low	Moderate		High	No included studies	
5. VALUES	Important uncertainty or variability		Probably Important uncertainty or variability	Probably no Important uncertainty or variability		No Important uncertainty or variability	Varies	
6. BALANCE OF EFFECTS	Favours the comparison	Probably favours the comparison	Does not favour either the intervention or the comparison		Probably favours the intervention	Favours the intervention	Varies	Don’ t know
7. RESOURCES REQUIRED	Large costs	Moderate costs	Negligible costs or savings		Large savings	Moderate savings	Varies	Don’ t know
8. CERTAINTY OF EVIDENCE OF REQUIRED RESOURCES	Very low	Low	Moderate		High		No included studies	
9. COST-EFFECTIVENESS	Favours the comparison	Probably favours the comparison	Does not favour either the intervention or the comparison		Probably favours the intervention	Favours the intervention	Varies	Don’ t know
10. EQUITY	Reduced	Probably Reduced	Probably no impact		Probably Increased	Increased	Varies	Don’ t know
11. ACCEPTABILITY	No	Probably no	Probably Yes		Yes		Varies	Don’ t know
12. FEASIBILITY	No	Probably no	Probably Yes		Yes		Varies	Don’ t know

TYPE OF RECOMMENDATION

Strong recommendation against the intervention	Conditional recommendation against the intervention	Conditional recommendation for either the intervention or the comparison	Conditional recommendation for the intervention	Strong recommendation for the intervention
○	○	○	○	○

CONCLUSIONS

Reason for recommendation

After experts discussion, the cause of infusion-related reactions is not clear, so this recommendation is deleted, and the evidence of this recommendation is displayed in the recommendation description of recommendation 15 for reference.

Recommendation 18: RTX treatment of children with SSNS does not increase the total medical expenditure. (1D)

What is the cost-effectiveness of RTX treatment in children aged 1-18 years with SSNS?	
STUDY TYPE	Cohort study and self-control study
POPULATION	SSNS
INTERVENTION	RTX
COMPARISON	CNI
MAIN OUTCOMES	Number of relapses
PERSPECTIVE	Clinicians, social workers and parents of children with FRNS/SDNS
SETTING	Hospital
CONFLICT OF INTERESTS	No

ASSESSMENT

1. PROBLEM: Is the problem a priority?

Detailed judgments: The more serious or urgent a problem is, the more likely it is that an option that addresses the problem will be a priority.

利妥昔单抗治疗的成本效果问题具有优先性么？根据问题的严重性和紧急性程度判断其是否具有优先性。严重性和紧急性程度越高，优先性越大

JUDGEMENTS	RESEARCH EVIDENCE	ADDITIONAL CONSIDERATIONS
□ No<br>□ Probably no<br>□ Probably yes<br>☑ **Yes**<br>□ Varies<br>□ Uncertain	/	Vote Results： 2/14 chose “Probably Yes”， **12/14 chose “Yes”．**

2. Desirable effects: How substantial are the desirable anticipated effects?

Detailed judgments: How large are the desirable effects of the intervention taking into account the importance of the outcomes (how much they are valued), and the size of the effect (the

likelihood of experiencing a benefit or how much of an improvement individuals would be likely to experience)?<br>RTX 药物治疗 SSNS 患儿在不 Increased 总医疗开支的情况下可 Reduced 复发次数的有益影响有多大？根据结局重要性（受重视的程度）和效应值大小（获益的可能性和个体情况改善的程度）对干预措施的有益影响进行判断		
**JUDGEMENTS**	**RESEARCH EVIDENCE**	**ADDITIONAL CONSIDERATIONS**
☐ Trivial<br>☐ Small<br>☐ Moderate<br>☑ **Large**<br>☐ Varies<br>☐ Uncertain	/	Vote Results : 4/14 chose "Moderate", **9/14 chose "Large"**, 1/14 chose "Don' t know".

3. Undesirable effects: How substantial are the undesirable anticipated effects?

Detailed judgments: How large are the desirable effects of the intervention taking into account the importance of the outcomes (how much they are valued), and the size of the effect (the likelihood of experiencing a benefit or how much of an improvement individuals would be likely to experience)?

RTX 药物治疗 SSNS 患儿在不 Increased 总医疗开支的情况下可 Reduced 复发次数的不良影响有多大？根据结局重要性（受重视的程度）和效应值大小（获益的可能性和个体情况改善的程度）对干预措施的不良影响进行判断

**JUDGEMENTS**	**RESEARCH EVIDENCE**	**ADDITIONAL CONSIDERATIONS**
☐ Large<br>☐ Moderate<br>☑ **Small**<br>☐ Trivial<br>☐ Varies<br>☐ Uncertain	/	Vote Results : 1/14 chose "Moderate", **7/14 chose "Small"**, 5/14 chose " Trivial ", 1/14 chose "Varies".

4. Certainty of the evidence: What is the overall certainty of the evidence of effects?

Detailed judgments: How good an indication does the research provide of the likely effects across all of the critical outcomes; i.e. the likelihood that the effects will be different enough from what the research found that it might affect a decision about the intervention?

RTX 药物治疗 SSNS 患儿在不 Increased 总医疗开支的情况下可 Reduced 复发次数有益影响和不良影响相关证据的总体质量？基于干预对所有关键结局的可能影响，判断干预效果是否会对干预决策产生影响

**JUDGEMENTS**	**RESEARCH EVIDENCE**	**ADDITIONAL CONSIDERATIONS**
☑ **Very low**<br>☐ Low<br>☐ Moderate<br>☐ High	/	Vote Results : **8/14 chose " Very low"**, 5/14 chose "Low", 1/14 chose "Moderate".

□ No included studies		

5. Values: Is there important uncertainty about or variability in how much people value the main outcomes?

Detailed judgments: How much do individuals value each of the main outcomes? Is uncertainty about how much they value each of the outcomes or variability in how much different individual value the outcomes large enough that it could lead to different decisions?

对 RTX 药物治疗 SSNS 患儿在不 Increased 总医疗开支的情况下可 Reduced 复发次数的重视程度，是否因个体不同而存在不确定性和变化性？个体对 RTX 药物治疗 SSNS 患儿在不 Increased 总医疗开支的情况下可 Reduced 复发次数的重视程度有多大？不确定性和变化性是否会导致不同的决策？不确定性体现在对上述推荐的理解程度；变化性体现在对上述推荐重视程度的差异。

JUDGEMENTS	RESEARCH EVIDENCE	ADDITIONAL CONSIDERATIONS
□ Important uncertainty or variability<br>☑ **Probably important uncertainty or variability**<br>□ Probably no important uncertainty or variability<br>□ No important uncertainty or variability	/	Vote Results：2/14 chose “Important uncertainty or variability”，**8/14 chose “Probably Important uncertainty or variability”**，2/14 chose “Probably no Important uncertainty or variability”，2/14 chose “No Important uncertainty or variability”.

6. Balance of effects: Does the balance between desirable and undesirable effects favour the intervention or the comparison?

Detailed judgments: What is the balance between the desirable and undesirable effects, taking into account how much individuals value the main outcome, how substantial the desirable and undesirable effects are, the certainty of those estimates, discount rates, risk aversion and risk seeking?

权衡 RTX 药物治疗 SSNS 患儿在不 Increased 总医疗开支的情况下可降低复发次数利弊后更倾向于 RTX 组还是对照组 CNI。从以下方面权衡利弊：个体对上述推荐的重视程度；利多大？；弊多大？；估计值的精确性；信心有多大？；风险多大？；可能规避风险么？

JUDGEMENTS	RESEARCH EVIDENCE	ADDITIONAL CONSIDERATIONS
□ Favours the comparison<br>□ Probably favours the comparison<br>□ Does not favour either the intervention or the comparison<br>□ Probably favours the intervention<br>☑ **Favours the intervention**<br>□ Varies<br>□ Uncertain	/	Vote Results：**13/14 chose “Favours the intervention”**，1/14 chose “Don’t know”.

7. Resources required: How large are the resource requirements (costs)?

Detailed judgments: How large is the cost of the difference in resource use between the intervention and comparision?

RTX 药物治疗 SSNS 患儿在不 Increased 总医疗开支的情况下可 Reduced 复发次数成本支出有多大？RTX 和对照组 CNI 的支出成本差异有多大？		
**JUDGEMENTS**	**RESEARCH EVIDENCE**	**ADDITIONAL CONSIDERATIONS**
□ Large costs<br>□ Moderate costs<br>☑ **Negligible costs or savings**<br>□ Moderate savings<br>□ Large savings<br>□ Varies<br>□ Uncertain	/	Vote Results： 1/14 chose "Moderate costs"， **8/14 chose "Negligible costs or savings"**， 5/14 chose "Moderate savings" .

8. Certainty of evidence of required resources: What is the certainty of the evidence of resource requirements (costs)?

Detailed judgments: How certain is the evidence of a difference for each type of resource use (eg. drugs, hospitalizations) and the cost of resources?

成本支出的证据质量如何？对 RTX 药物治疗 SSNS 患儿在不 Increased 总医疗开支的情况下可降低复发次数成本支出（包括药物、住院等费用）相关证据的确定性。

**JUDGEMENTS**	**RESEARCH EVIDENCE**	**ADDITIONAL CONSIDERATIONS**
□ Very low<br>☑ **Low**<br>□ Moderate<br>□ High<br>□ No included studies	/	Vote Results： 2/14 chose "Very low"， **9/14 chose "Low"**， 2/14 chose "Moderate"， 1/14 chose "High" .

9. Cost-effectiveness: Does the cost-effectiveness of the intervention favour the intervention or the comparison?

Detailed judgments: Is the intervention cost-effective, taking into account uncertainty about or variability in the costs, uncertainty about or variability in the net benefit, sensitivity analyses, and the reliability and applicability of the economic evaluation?

9. 成本效益：

Detailed judgments: 对 RTX 药物治疗 SSNS 患儿在不 Increased 总医疗开支的情况下可降低复发次数干预的成本效益分析更倾向于 RTX 还是对照组 CNI。从以下方面分析干预的成本效益：对支出成本的不确定性或变化性；对净利润的不确定性或变化性；敏感性分析；经济评估的可靠性和适用性。

**JUDGEMENTS**	**RESEARCH EVIDENCE**	**ADDITIONAL CONSIDERATIONS**

□ Favours the comparison<br>□ Probably favours the comparison<br>□ Does not favour either the intervention or the comparison<br>☑ **Probably favours the intervention**<br>☑ **Favours the intervention**<br>□ Varies<br>□ No included studies	/	Vote Results： 2/14 chose “Does not favour either the intervention or the comparison”， **6/14 chose “Probably favours the intervention”， 6/14 chose “Favours the intervention”.**

10. EQUITY: What would be the impact on health equity?

Detailed judgments: Are there plausible reasons for anticipating differences in the relative effectiveness of the intervention for disadvantaged subgroups or different baseline conditions across disadvantaged subgroups that affect the absolute effectiveness of the intervention or the importance of the problem?

对卫生公平性的影响？RTX 药物治疗 SSNS 患儿在不增加总医疗开支的情况下可降低复发次数的相对有效性在弱势人群中有所降低，对此是否有合理的解释？弱势人群的不同基线水平会影响干预的绝对有效性或研究问题的重要性，对此是否有合理的解释？

JUDGEMENTS	RESEARCH EVIDENCE	ADDITIONAL CONSIDERATIONS
□ Reduced<br>☑ **Probably Reduced**<br>□ Probably no impact<br>□ Probably increased<br>□ Increased<br>□ Varies<br>□ Uncertain	/	Vote Results： **5/14 chose “Probably Reduced”**， 1/14 chose “Probably no impact”， 2/14 chose “Probably increased”， 3/14 chose “Increased”， 3/14 chose “Varies”.<br>考虑同样情况下弱势群体 Probably 产生的问题

11. ACCEPTABILITY: Is the intervention acceptable to key stakeholders?

Detailed judgments: Are key stakeholders likely not to accept the distribution of the benefits, harms and costs; or the costs or undesirable effects in the short term for desirable effects (benefits) in the future? Are they likely to disagree with the values attached to the desirable or undesirable effects, or not to accept the diagnostic intervention because of ethical concerns?

患儿及其家长是否接受 RTX 药物治疗 SSNS 患儿在不 Increased 总医疗开支的情况下可 Reduced 复发次数。患儿及其家长是否会接受干预带来的获益、伤害及支出，或远期获益带来的短期内的不良反应；是否会对利弊判断背后的价值观念持反对意见；是否会出于伦理考虑拒绝诊断性治疗。

JUDGEMENTS	RESEARCH EVIDENCE	ADDITIONAL CONSIDERATIONS
□ No<br>□ Probably no<br>□ Probably yes<br>☑ **Yes**<br>□ Varies<br>□ Uncertain	/	Vote Results： 4/14 chose “Probably Yes”， **10/14 chose “Yes”.**

12. FEASABILITY: Is the option feasible to implement?

Detailed judgments: Is it feasible to sustain use of the intervention and to address potential barriers to using it?

JUDGEMENTS	RESEARCH EVIDENCE	ADDITIONAL CONSIDERATIONS
☐ No<br>☐ Probably no<br>☐ Probably yes<br>☑ **Yes**<br>☐ Varies<br>☐ Uncertain	/	Vote Results： 2/14 chose "Probably Yes", **12/14 chose "Yes"**.

SUMMARY OF JUDGEMENTS

<table>
<tr><th>CRITERIA</th><th colspan="8">DECISION</th></tr>
<tr><td>1. PROBLEM</td><td colspan="2">No</td><td>Probably no</td><td colspan="2">Probably Yes</td><td>Yes</td><td>Varies</td><td>Don’ t know</td></tr>
<tr><td>2. BENEFITS</td><td colspan="2">Trivial</td><td>Small</td><td colspan="2">Moderate</td><td>Large</td><td>Varies</td><td>Don’ t know</td></tr>
<tr><td>3. HARMS</td><td colspan="2">Large</td><td>Moderate</td><td colspan="2">Small</td><td>Trivial</td><td>Varies</td><td>Don’ t know</td></tr>
<tr><td>4. QUALITY OF EVIDENCE</td><td colspan="2">Very low</td><td>Low</td><td colspan="2">Moderate</td><td>High</td><td colspan="2">No included studies</td></tr>
<tr><td>5. VALUES</td><td colspan="2">Important uncertainty or variability</td><td>Probably Important uncertainty or variability</td><td colspan="2">Probably no Important uncertainty or variability</td><td>No Important uncertainty or variability</td><td colspan="2">Varies</td></tr>
<tr><td>6. BALANCE OF EFFECTS</td><td>Favours the comparison</td><td>Probably favours the comparison</td><td colspan="2">Does not favour either the intervention or the comparison</td><td>Probably favours the intervention</td><td>Favours the intervention</td><td>Varies</td><td>Don’ t know</td></tr>
<tr><td>7. RESOURCES REQUIRED</td><td>Large costs</td><td>Moderate costs</td><td colspan="2">Negligible costs or savings</td><td>Large savings</td><td>Moderate savings</td><td>Varies</td><td>Don’ t know</td></tr>
<tr><td>8. CERTAINTY OF EVIDENCE OF REQUIRED RESOURCES</td><td>Very low</td><td>Low</td><td colspan="2">Moderate</td><td colspan="2">High</td><td colspan="2">No included studies</td></tr>
<tr><td>9. COST-EFFECTIVENESS</td><td>Favours the comparison</td><td>Probably favours the comparison</td><td colspan="2">Does not favour either the intervention or the comparison</td><td>Probably favours the intervention</td><td>Favours the intervention</td><td>Varies</td><td>Don’ t know</td></tr>
<tr><td>10. EQUITY</td><td>Reduced</td><td>Probably Reduced</td><td colspan="2">Probably no impact</td><td>Probably Increased</td><td>Increased</td><td>Varies</td><td>Don’ t know</td></tr>
<tr><td>11. ACCEPTABILITY</td><td>No</td><td>Probably no</td><td colspan="2">Probably Yes</td><td colspan="2">Yes</td><td>Varies</td><td>Don’ t know</td></tr>
<tr><td>12. FEASIBILITY</td><td>No</td><td>Probably no</td><td colspan="2">Probably Yes</td><td colspan="2">Yes</td><td>Varies</td><td>Don’ t know</td></tr>
</table>

TYPE OF RECOMMENDATION

Strong recommendation against the intervention	Conditional recommendation against the intervention	Conditional recommendation for either the intervention or the comparison	Conditional recommendation for the intervention	Strong recommendation for the intervention
○	○	○	○	○

CONCLUSIONS

Reason for recommendation

Medical expenditure is an important factor to consider.

Recommendation(text)

RTX treatment of children with SSNS does not increase the total medical expenditure. (1D)

Implementation considerations

1. The two studies are not conducted in China (one is from the United States, only cost analysis and effect analysis; another one is from Japan, including cost analysis, effect analysis and cost-effectiveness analysis). Although it can be switched to US dollars for discussion, there may be national differences;
2. The included references are few and the quality of evidence is very low;
3. The possibility of entering medical insurance in the future needs to be considered;

4. It is necessary to take other expenses besides the cost of drugs into consideration, such as hospital expenses.

Research priorities

Cost-effectiveness analysis
